# Tool recommender system in Galaxy using deep learning

**DOI:** 10.1093/gigascience/giaa152

**Published:** 2021-01-06

**Authors:** Anup Kumar, Helena Rasche, Björn Grüning, Rolf Backofen

**Affiliations:** Bioinformatics Group, Department of Computer Science, University of Freiburg, Georges-Koehler-Allee 106, 79110 Freiburg, Germany; Bioinformatics Group, Department of Computer Science, University of Freiburg, Georges-Koehler-Allee 106, 79110 Freiburg, Germany; Bioinformatics Group, Department of Computer Science, University of Freiburg, Georges-Koehler-Allee 106, 79110 Freiburg, Germany; Bioinformatics Group, Department of Computer Science, University of Freiburg, Georges-Koehler-Allee 106, 79110 Freiburg, Germany; Signalling Research Centres BIOSS and CIBSS, University of Freiburg, Schaenzlestr. 18, 79104 Freiburg, Germany

**Keywords:** recommender system, Galaxy, workflows, deep learning, neural networks, gated recurrent units

## Abstract

**Background:**

Galaxy is a web-based and open-source scientific data-processing platform. Researchers compose pipelines in Galaxy to analyse scientific data. These pipelines, also known as workflows, can be complex and difficult to create from thousands of tools, especially for researchers new to Galaxy. To help researchers with creating workflows, a system is developed to recommend tools that can facilitate further data analysis.

**Findings:**

A model is developed to recommend tools using a deep learning approach by analysing workflows composed by researchers on the European Galaxy server. The higher-order dependencies in workflows, represented as directed acyclic graphs, are learned by training a gated recurrent units neural network, a variant of a recurrent neural network. In the neural network training, the weights of tools used are derived from their usage frequencies over time and the sequences of tools are uniformly sampled from training data. Hyperparameters of the neural network are optimized using Bayesian optimization. Mean accuracy of 98% in recommending tools is achieved for the top-1 metric.

**Conclusions:**

The model is accessed by a Galaxy API to provide researchers with recommended tools in an interactive manner using multiple user interface integrations on the European Galaxy server. High-quality and highly used tools are shown at the top of the recommendations. The scripts and data to create the recommendation system are available under MIT license at https://github.com/anuprulez/galaxy_tool_recommendation.

## Background

Life sciences depend increasingly on high-throughput data, turning them into data science to a large extent. However, raw high-throughput data have little value on their own without proper analysis and interpretation. To simplify the data analysis process and to ensure a reproducible analysis, several workflow systems such as Bcbio-nextgen, Omics Pipe, Nextflow, Luigi, Toil, and many others have emerged [1–3]. The main idea for workflow systems is based on the observation that any computational analysis of high-throughput data encompasses multiple steps such as quality control, preprocessing, quantification, and statistical analysis to transform raw data into scientific results. Collectively, these steps form a workflow where each step performs a definite transformation of the data, which can be performed using standardized tools. Using workflow for the analysis is simple and convenient and has several advantages. First, it is easy to replace individual tools by a newer version or to assess the influence of the associated step on the final result. Second, a workflow can be saved, shared, and reused, which ensures reproducible research. Therefore, workflows are becoming essential in the analysis of scientific data and there are multiple platforms where researchers can create workflows for their analyses. However, a critical question is how to assess whether a generated workflow is state-of-the-art or even valid at all. To give a concrete example, one can use several real-valued input vectors (such as fluorescence-based measurement stemming from arrays), transform them into integer-based values in the first step, and combine it with a tool that uses a count-based statistics (such as negative binomial distribution as used in DESeq2 [4]) to determine values that show high differential behaviour. While this workflow would run on a workflow system without problems and even produce some results, the generated results are not valid because the wrong statistical model was applied. Therefore, it is important to use a tool for each step in a workflow that can bring desired results. To make this possible, a system is needed that can recommend useful tools at each step while creating a workflow.

### Galaxy and workflows

Galaxy is an open-source data-processing platform that enables researchers to create and store their workflows for multiple scientific analyses [5]. A workflow in Galaxy is a directed acyclic graph and consists of 1 or many tool sequences to analyse scientific data such as DNA and RNA sequences. A tool consumes ≥1 data file as input, produces ≥1 data file as output, and supports a number of formats of these input and output files. In workflows, the tools are connected one after another following a constraint that the adjacent tools must have compatible data types. In other words, the data types of output files of a tool should match the data types of input files of the following tool. Galaxy has thousands of accessible tools, and acquiring familiarity and constructing workflows with these tools can be a complex and time-consuming task, especially for researchers new to Galaxy. To assist them in creating workflows and making them aware of the possible tools for further analyses, a recommender system is devised. The benefits of such a system are manifold. First, it will make researchers more efficient by saving the time they waste creating erroneous or less optimal workflows by choosing tools that may produce undesired results. Second, it will help researchers bypass the step of searching for tools separately, which will further reduce the time spent in creating workflows and at the same time increase the accessibility of tools. Third, it will promote high-quality tools that have been used more often in the past (last 1 year) to the top of the recommendations and downgrade those having lower usage frequencies. This is achieved by assigning each tool a weight derived from its usage frequency over a period of time. Finally, it can be extended to promote the newly added tools in Galaxy by showing them alongside the recommended tools predicted using the neural network approach.

### Recommender systems

The objective of having recommender systems in fields such as scientific literature search, online shopping, travel bookings, media-service providers, and many other fields is to help people discover suitable, interesting, and newly released products. These recommended products are recognized on the basis of the usage and purchasing patterns of people in the past. In the field of scientific literature search, the exponential increase in the number of published articles necessitates having a recommender system to help scientists explore relevant and recent papers quickly [6–8]. Recommender systems are important in the world of commercial applications too. Companies such as Amazon and Netflix have appropriately used them to learn the preferences of their respective customers in selecting products such as their favourite books or movies and to propose a few products out of a large catalogue. By enabling users and customers to discover reasonable and customized products, recommender systems have helped them grow as organizations [9, 10]. In short, recommender systems make it faster for users and customers to look through a few suggested products to find the most suitable ones. These successful implementations of recommender systems by organizations across the world working in diverse areas to assess the needs of their respective users in proposing relevant products motivated us to create a tool recommender system in Galaxy.

### Related work

**Figure 1:**
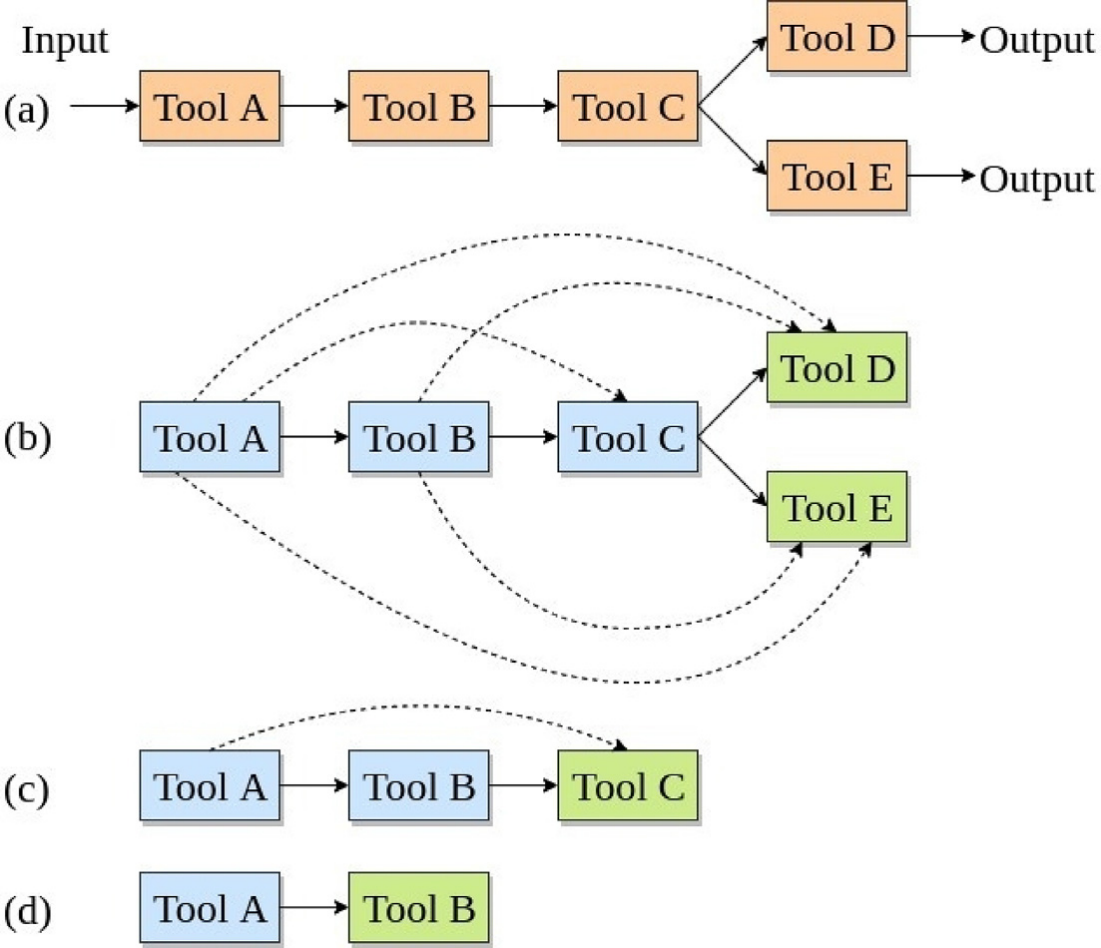
An example workflow consisting of 5 different tools (a) is decomposed into multiple tool sequences (b–d). Each tool sequence shows higher-order dependencies where a tool is dependent on all of its prior tools. These dependencies are indicated by the dashed arrows.

To simplify creating workflows for scientific analyses, a few approaches have been proposed that suggest alternative tools and workflows. EDAM and semantic annotations of tools are used to compose workflows automatically for mass spectrometry–based proteomics [11]. The annotations include the names, functionalities, and input and output data types of tools. The PROPHETS program generates suitable candidates of workflows that match the goal of the proposed workflow and its annotations [12]. WINGS offers multiple variations of a workflow created using different tools. It makes use of the input parameters, types of datasets, and functions of tools to build the variations [13, 14]. The approach used by DiBernardo et al. [15] uses data types to facilitate the automatic creation of workflows. All these approaches depend on either annotations or matching input and output data types of adjacent tools in workflows, and they pose challenges such as the addition and maintenance of the meaningful annotations of tools and extracting input and output data types of adjacent tools. Moreover, these approaches have their workflow generation restricted to a few specific bioinformatics analyses such as proteomics or proteogenomics. In addition, they do not discuss the presence of higher-order relationships [16] in tool sequences of workflows. Our approach to recommend tools in workflows aims to overcome these challenges in the following manner. First, it does not require collecting and storing information about tools. Second, it takes into account the higher-order relationships among tools (Fig. 1) in tool sequences. Finally, it incorporates workflows from multiple scientific analyses to produce the recommender system.

### Sequential learning on workflows

Workflows, created by many researchers in Galaxy for different scientific analyses, are decomposed into numerous tool sequences (Fig. 1). The sequential nature of these tool sequences where tools are connected one after another inspires us to apply similar learning techniques used for other sequential data such as text and speech. There are multiple studies in the fields of natural language processing, clinical research, and speech recognition that apply deep learning techniques on sequential data to obtain good accuracy in predicting future items. The approach used by Yin et al. [17] finds context in long sequences of words for sentiment analysis and part-of-speech tagging using recurrent neural network (RNN) and achieves 85% and 93% accuracy, respectively. For clinical data, learning on long sequences of health states proves to be beneficial [18]. The health states of patients recorded at different time points are analysed by accessing their electronic health records. The future health states of patients could be predicted by training RNN on the sequences of their past health states to achieve 85% accuracy. Moreover, variants of RNN are used to model speech and music signals [19, 20]. These successful studies benefit from sequential learning techniques using different variants of RNN. Therefore, in our work, a variant of RNN—gated recurrent units (GRU)—is used to create the tool recommender system in Galaxy.

A Bayesian network can also be used for modelling directed acyclic graphs (workflows) [21, 22]. It requires the computation of joint and conditional probabilities of nodes in graphs, and an increase in the number of nodes can lead to a higher cost to compute these probabilities. In addition, making predictions by learning a probabilistic network is a hard problem [23–25]. Because of these drawbacks of using a Bayesian network, it is not used in our approach to create the recommender system in Galaxy.

## Data Description

More than 18,000 workflows from different scientific analyses such as RNA-seq, variant-calling, Hi-C, assembly, single-cell, proteomics, and so on in the European Galaxy server [26] have been used to create the recommender system. A workflow consisting of 5 tools is shown in Fig. 1a. It is divided into smaller tool sequences as shown in Fig. 1b–d. The last tool, shown in green, of each tool sequence (of length n) is assigned as the label of the subsequence (of length n − 1) shown in blue in Fig. 1. A label is an output that is learned and predicted by the recommender system. In the neural network learning, a tool is a label. For example, in Fig. 1b, Tools D and E are the labels of the subsequence Tool A → Tool B → Tool C. They show higher-order dependencies in their connections, which implies that a tool is dependent not only on its immediate predecessor but also on all prior tools in the tool sequence. For example, in Fig. 1c, the Tool C is dependent on Tools B and A. By analysing multiple workflow fragments in this way, the neural network learns that the label of a tool sequence Tool A → Tool B is Tool C. It is expected that dividing a tool sequence into fragments with a minimum length of 2 tools, as shown in Fig. 1c and d, will improve the generalization performance of the neural network because it gets more tool sequences with a variety of lengths to learn from. The dependencies shown in Fig. 1b–d present in tool sequences are learned using the GRU neural network by modelling the conditional probability given by Equation 1 [27]. Using the approach explained above, >229,000 tool sequences are extracted.

## Usage pattern of tools

**Figure 2:**
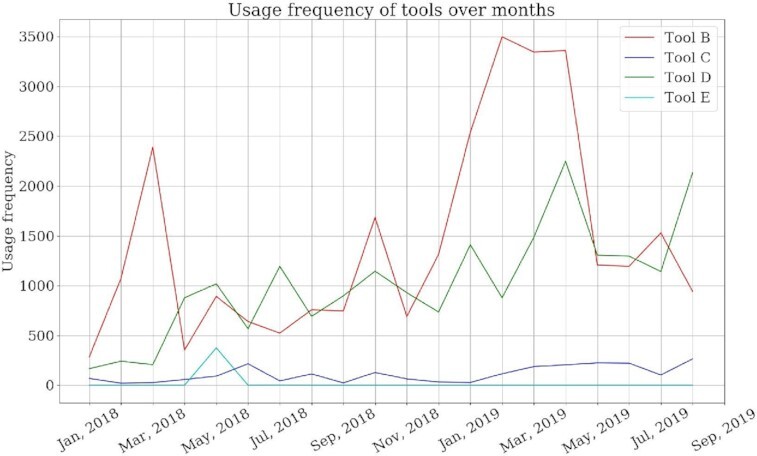
The usage frequencies of 4 tools collected over the past 1 year. Tools B and D have high usage frequencies almost every month, while Tools C and E have much lower usage frequencies. Tool A is absent from the plot because it is not the label of any tool for the workflow shown in Fig. 1.

Tools in Galaxy have different usage patterns. Some tools are used more often than other tools for multiple reasons such as differences in their functions and availability of similar but better tools. It is essential to analyse the usage patterns of tools because the recommender system proposes tools for researchers and these tools should have high relevance to their analyses. One of the key indicators of the relevance of tools can be their high usage frequencies. If a tool has been used often in the recent past, it implies that the tool is relevant. However, if a tool was used often a few years ago but has been used less often in the past 6 months, then the relevance of that tool has certainly declined. The usage frequencies of tools (shown as labels in Fig. 1) over the past year are shown in Fig. 2.

### Shared and non-shared workflows

The set of workflows may have low-quality workflows that have not been published or are deleted or may have errors in their tool connections. To distinguish between high- and low-quality tool connections, the labels for each tool sequence are divided into 2 categories—shared and non-shared labels. The shared labels come from the published, non-deleted, and non-erroneous workflows while the non-published labels come from other workflows. While recommending tools for a tool or tool sequence, the shared labels are promoted to the top of the recommendations if available, followed by the non-shared labels. This enables high-quality tools to be shown as the top recommendations.

### Imbalance in workflows

Tool sequences from these workflows may vary in number—some may occur more frequently and others may not. Therefore, the complete set of tool sequences may not be equally representative of all workflows coming from different scientific analyses. Learning on the imbalanced set of tool sequences can induce bias, which may have an undesired outcome—high accuracy in recommending tools coming from highly frequent tool sequences and low accuracy for tools coming from less frequent ones. To mitigate this bias, all tool sequences are chosen with uniform frequency while training the neural network, which allows it to attain comparable accuracy in recommending tools in different scientific analyses. This uniform sampling strategy is discussed in the “Implementation” section in detail.

## Results

**Figure 3:**
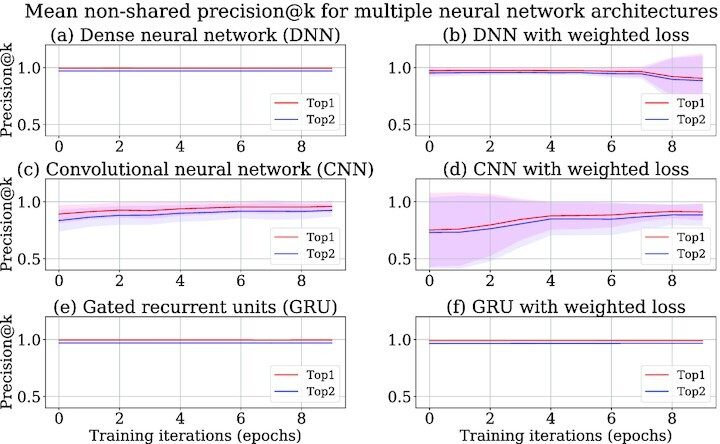
Top-k (precision@k) non-shared precision for DNN, CNN, and GRU neural networks with cross-entropy loss function in (a), (c), and (e) respectively . Top-k (precision@k) non-shared precision for DNN, CNN, and GRU neural networks with weighted cross-entropy loss function in (b), (d), and (f) respectively .

**Figure 4:**
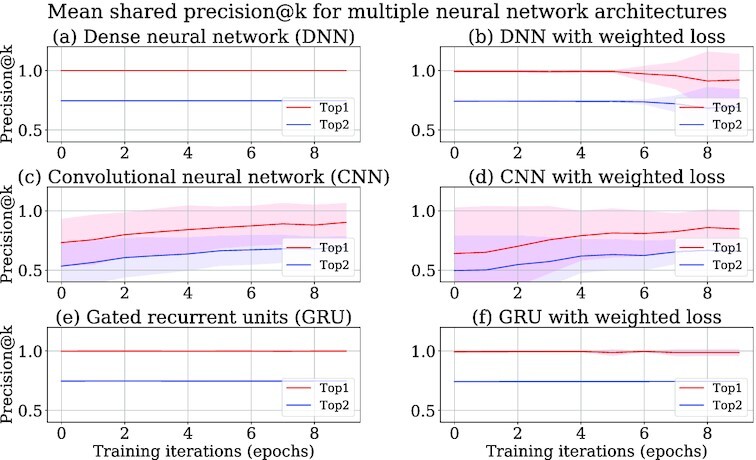
Top-k (precision@k) shared precision for DNN, CNN, and GRU neural networks with cross-entropy loss function in (a), (c), and (e) respectively. Top-k (precision@k) shared precision for DNN, CNN, and GRU neural networks with weighted cross-entropy loss function in (b), (d), and (f) respectively .

**Figure 5:**
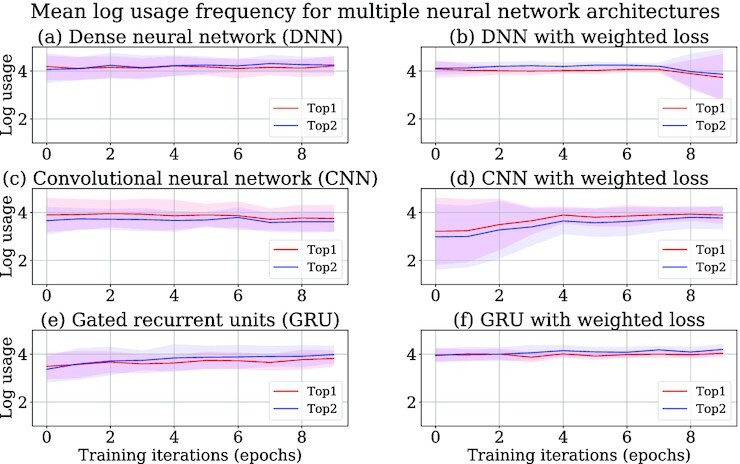
Usage frequencies of (top-k) predicted tools for DNN, CNN, and GRU neural networks with cross-entropy loss function in (a), (c), and (e) respectively. Usage frequencies of (top-k) predicted tools for DNN, CNN, and GRU neural networks with weighted cross-entropy loss function in (b), (d), and (f) respectively.

Three different neural network architectures—dense neural network (DNN), convolutional neural network (CNN), and gated recurrent units neural network (GRU)—are compared on their performances in predicting tools (Figs 3 and 4). The models obtained after training all the neural network architectures are used to predict tools for the tool sequences in the test data after every training iteration. Top-k precision (precision@k) is a popular metric for evaluating a recommender system [28–30]. Precision@k implies how many of the *k* predicted tools are correct. The correctness here refers to the compatibility of the predicted tools with the tool for which predictions have been made. For example, *k* = 2 implies that there are 2 predicted tools with the highest predicted scores. If only 1 of them is correct, then the precision@2 is $1/2 = 0.5$. In this way, precision@1 and precision@2 are computed for all the tool sequences in the test data and then averaged. Precision@1 and precision@2 metrics are used in this approach to evaluate the quality of the tool recommender system. The precisions of recommended tools are computed separately for the non-shared and shared recommendations and shown in different plots (Figs 3 and 4), but for usage frequencies, they are combined into 1 plot (Fig. 5). The precision and usage frequencies of the predicted tools for the precision@1 and precision@2 (top-1 and top-2) metrics are computed over 10 training iterations for each experiment run. They are averaged and their respective standard deviations are computed over 10 experiment runs. Mean precision and usage frequencies are shown by the respective line plots, and the shaded regions span the area between 1 standard deviation above and below the mean (Figs 3–5).

### Comparison of GRU neural network with other approaches

The GRU neural network with the weighted cross-entropy loss function shows superior performance to CNN (Figs 3d, and f, 4d and f) by achieving 98% top-1 non-shared and shared precision, which proves that the GRU layers in a neural network are better for learning on tool sequences than the convolutional layer. Moreover, it shows a lower divergence in non-shared and shared precision and usage frequencies (Figs 3d and f, 4d and f, 5d and f), establishing that its predictive strength is more stable than CNN over multiple experiment runs. Surprisingly, the weighted cross-entropy loss function does not have any beneficial effect on the CNN architecture as its non-shared and shared precision and usage frequencies show higher divergence over multiple experiment runs (Figs 3c and d, 4c and d, 5c and d). Therefore, CNN is not used in our approach. In contrast to CNN, DNN achieves a similar non-shared and shared precision to the GRU neural network with a small divergence (Figs 3a, b, and f and 4a, b, and f). However, due to higher divergence in accumulated usage frequencies, it is not used in our approach (Fig. 5a, b, and f). Weighted cross-entropy loss function in the GRU neural network (Fig. 5f) drives it to classify tools more robustly with higher usage frequencies. In other words, it predicts tools with higher precision than CNN and lower divergence in usage frequencies than DNN. Therefore, it is used in our approach to learn on tool sequences and recommend tools.

To compare the performance of the GRU neural network with approaches that do not use any neural network, 2 ideas are explored. The first approach simply stores all the sequences of tools [31] formed using the technique shown in Fig. 1 to create a model. To recommend tools using this model, all the tool sequences are searched for a given tool or a sequence of tools. The second approach uses the ExtraTrees classifier [32] to recommend tools. These two approaches are discussed in section S1 of the supplementary document in detail.

### Benefit of regularization

Using regularization minimizes overfitting by assisting the GRU neural network to make better recommendations by predicting tools that have low usage frequencies but are useful in addition to tools with high usage frequencies. For example, the recommendations of “UMI-tools count" [33] tool with the regularized model include the “Seurat" [34] tool, which is absent from recommendations by the non-regularized model. Another example is for “RaceID, Lineage computation using StemID" tool sequence, which gets “Lineage Branch Analysis using StemID" [35] tool as one of the recommendations by the regularized model while the non-regularized model makes no recommendation at all. The recommendations for a popular mapper, RNA-STAR [36], are featureCounts [37], MultiQC [38], Infer Experiment [39], and a few others by both the models. But, in addition to these recommendations, the regularized model recommends the Read Distribution [39] tool, which is not predicted by the non-regularized model. More details are provided in Supplementary Table 1 in section S4 of the supplementary document.

### Examples of tool recommendations

**Figure 6:**
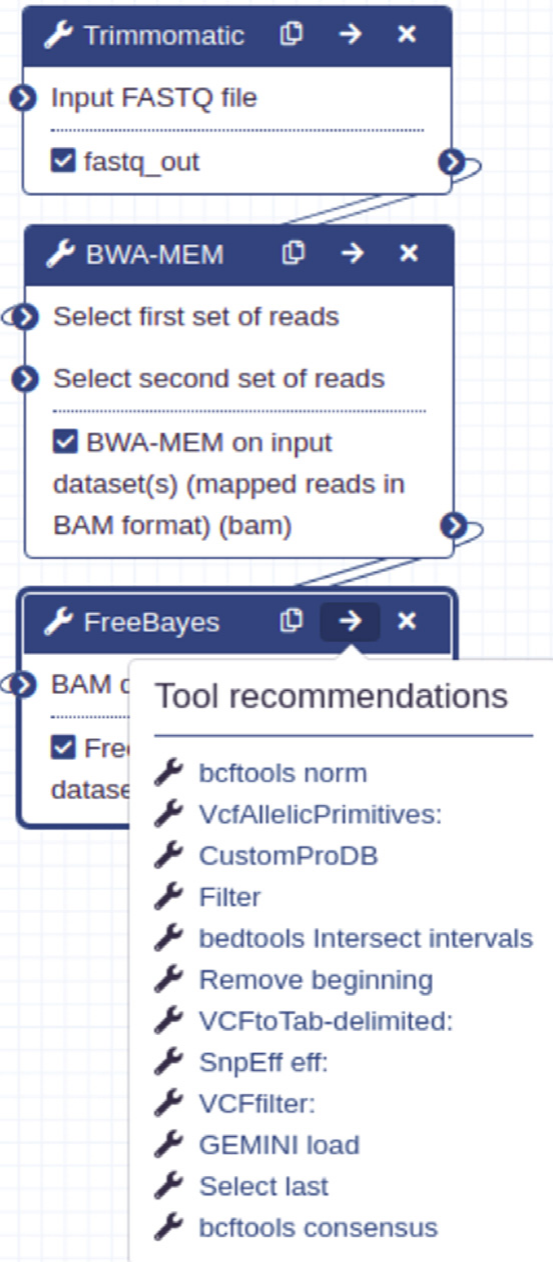
Recommended tools, listed in the “Tool recommendations" dropdown, in the workflow editor of the European Galaxy server for the Trimmomatic → BWA-MEM → FreeBayes tool sequence. The recommended tools for the tool sequence can be seen in a dropdown while hovering on the right arrow button visible in the top right corner of the “FreeBayes" tool. Clicking on any recommended tool such as “bcftools norm" in the dropdown opens a new block for the chosen tool that can be connected to the tool sequence.

**Figure 7:**
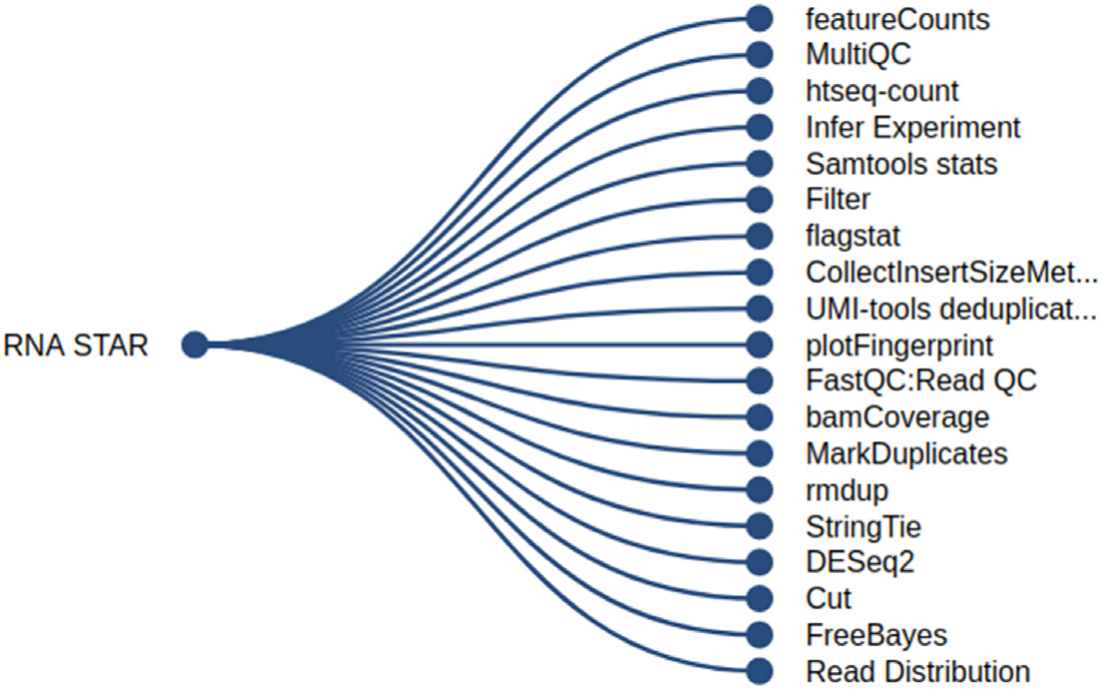
The figure shows recommended tools as leaves (on the right) of the tree after executing the RNA-STAR tool. Clicking on any recommended tool opens its definition in Galaxy and can be used for further analysis with the data files produced by the previous tool (RNA-STAR).

To illustrate the real-time use of the recommender system in the European Galaxy server, 2 examples are provided. The first shows recommended tools for a tool sequence with 3 tools, Trimmomatic [40] → BWA-MEM [41] → FreeBayes [42], in the workflow editor of the European Galaxy server (Fig. 6). Trimmomatic is used to trim sequencing data such as DNA and RNA sequences. One of the useful analyses after trimming the sequences is to map them on a reference genome using a mapper. Several mappers such as BWA-MEM [41], Bowtie2 [43], and RNA-STAR [36] are predicted. BWA-MEM is chosen from the predicted mappers and connected to Trimmomatic. After mapping, for further analysis of mapped sequences, many tools are predicted such as MultiQC for summarizing the quality of mapping, featureCounts for counting the reads mapped to different regions on the genome, or FreeBayes for detecting variants, and a few others. FreeBayes is chosen and a list of recommendations is shown as a dropdown containing tools such as bcftools norm [44], VcfAllelicPrimitives [45], and many others for the Trimmomatic → BWA-MEM → FreeBayes tool sequence. Another example of tool recommendations after using RNA-STAR is shown in Fig. 7. It shows follow-up tools such as bamCoverage [46] for calculating read coverage, MultiQC, featureCounts, and a few others. In summary, the tool recommendations provide useful knowledge about tools to Galaxy users and researchers to continue multiple scientific analyses.

Table 1 lists a few shared and non-shared recommended tools for multiple tool sequences in different scientific analyses such as computational chemistry, epigenetics, machine learning, proteomics, RNA sequencing, and a few others. The recommended tools shown in this table are frequently used for standard scientific analyses as highlighted in multiple Galaxy Training Network tutorials [60].

**Table 1.** Shared and non-shared recommendations for tool sequences in different scientific analyses

Scientific analysis	Tool/Tool sequences	Recommended tools	
		Shared	Non-shared
Computational chemistry	Molecule to fingerprint [47]	Taylor-Butina clustering, NxN Clustering [48], Similarity Search	
Epigenetics	hicBuildMatrix [49]	hicSumMatrices	hicMergeMatrixBins, hicPlotMatrix [50], hicPCA, hicTransform
	multiBamSummary [46]	plotCorrelation [51]	plotPCA
	Bowtie2	MASC2 CallPeak [51]	bamCoverage, FreeBayes
Machine learning	Create a deep learning model architecture		Create deep learning model [52], Build Deep learning Batch Training Models
Proteomics	Msconvert [53]	Search GUI, FlashLFQ	PeakPickerHiRes [54]
RNA Sequencing	Cutadapt [55]	FastQC, RNA-STAR, MultiQC [56]	Bowtie2, Hisat2, BWA-MEM
	Cutadapt [55], RNA-STAR	featureCounts, MultiQC, Infer Experiment [56]	bamCoverage, RmDup
Single-cell	UMI-tools extract [33]	RNA-STAR [57]	Bowtie2, Hisat2, BWA-MEM, UMI-tools group
	Initial processing using RaceID [35]	Clustering using RaceID [58]	
	Initial processing using RaceID, Clustering using RaceID [35]	Cluster Inspection using RaceID [58], Lineage computation using StemID	
Variant-calling	FreeBayes	VcfAllelicPrimitives [59]	Gemini load
	FreeBayes,VcfAllelicPrimitives	SnpSift Filter, VT normalize	SnpEff eff [59]

## Implementation

To create a tool recommender system in Galaxy, workflows are collected from the European Galaxy server. A workflow may have 1 or many tool sequences where tools are connected one after another. Tool sequences are transformed into matrices and produced as input to a GRU neural network to learn patterns in the connections of tools.

(1) \begin{equation*} p(x_T|x_1,x_2,....,x_{T-1}) \end{equation*}

The probability of a tool (*x_T_*) is estimated given all other prior tools (*x*_1_, ..., *x*_*T* − 1_) for a tool sequence (*x*_1_, ..., *x*_*T* − 1_, *x_T_*). Neural network learning is classification because there are labels for tool sequences, which are learned and then predicted. Moreover, the classification is multi-class (multiple tools as labels) and multi-label (multiple tools as labels for a tool sequence) [61]. To ensure unbiased learning and evaluation by the neural network, the set of tool sequences is divided into 2 parts—training and test. The training data are used for learning a model and the test data are used for evaluating the model.

### Uniform sampling

Workflows in Galaxy come from different scientific analyses. It may happen that the numbers of workflows from these analyses are not comparable—some analyses may have a large number of workflows while some may have only a small number of workflows. This can cause some tools to be present very frequently in workflows while other tools are less frequent. Learning on these workflows and recommending tools may exhibit bias by showing better recommendations for the frequently occurring tools and poorer recommendations for the less frequent ones. To showcase this imbalance, the frequencies of the last tool in each tool sequence in training data are calculated and it is found that only a few tools have large frequencies and most of the tools are present in low frequencies (Supplementary Fig. 3). For example, the tools with very high frequencies (>10,000) are “Concatenate datasets", “Cut", “Grouping", and “Join" while the tools with very low frequencies (<5) are “Cluster inspection using RaceID", “rDock cavity definition" [62], and “ChiRA collapse". Therefore, to overcome this drawback, the training data created after extracting tool sequences should be balanced to make the neural network learn on a similar number of tool sequences from different scientific analyses in each training iteration. To implement this strategy, a set of last tools in all tool sequences from the training data is collected. Furthermore, for each tool in this set, a list of indices of tool sequences in the training data are stored for which it is the last tool (Supplementary Table 3). Only the last tools are considered for implementing this strategy because of 2 reasons. First, the smallest tool sequences contain only 2 tools, and second, all tools become the last tool in ≥1 tool sequence and the computed frequencies of these last tools suggest their overall frequencies in the training data.

In the neural network training, for each iteration (which consumes all tool sequences in the training data), small batches containing an equal number of tool sequences are created. For example, if the batch size is 100 and the size of training data is 2,000, then 20 (2,000/100 = 20) batches are created, each containing 100 tool sequences. In each batch, 100 tools from the set of last tools are uniformly selected (Column 2 in Supplementary Table 3) and for each selected tool, a tool sequence is chosen uniformly from its respective list of tool indices (Column 3 in Supplementary Table 3). After selecting tool sequences for many batches for each iteration of training (epoch), it is expected that all the tools from the set of last tools and their respective tool sequences are chosen. Performing this uniform selection of different tool sequences for each iteration, the training data become balanced. Supplementary Fig. 4 shows that each last tool is present ∼1,670 times on average in each iteration (epoch). The order of tools in Supplementary Figs 3 and 4 is the same.

### Data transformation

**Figure 8:**
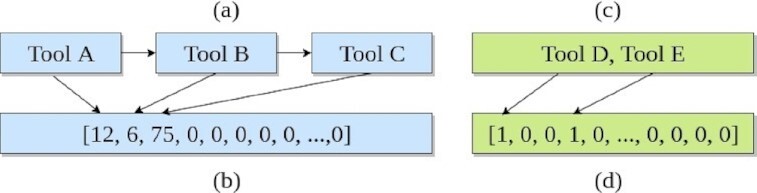
The figure shows how a tool sequence and its labels are transformed into vectors.

Tool sequences extracted from workflows are transformed into vectors because neural networks require input data to be represented as vectors and matrices. Each tool sequence has 1 or more labels (Fig. 1), and they are transformed into different vectors—a tool sequence vector (Fig. 8b) and a label vector (Fig. 8d). To form these vectors, a dictionary of tools is needed, which stores an index for each tool. Using the indices of tools, a tool sequence vector is created preserving the original order of tools as in the tool sequence. For example, Tool A has an index of “12" in the dictionary; therefore it is replaced by “12" in the vector (Fig. 8b). The vector is padded with trailing zeros to keep the length of the vector the same across the varying lengths of tool sequences. The size of this vector is 25, which means that a tool sequence can have a maximum of 25 tools. The tool sequences larger than this size are discarded. The labels (Fig. 8c) are transformed into a bit vector (Fig. 8d) in which the positions, stored as indices in the dictionary of tools, of the labels (tools) are turned “on" (set to 1), specifying that these tools are the labels of the tool sequence and others are not (set to 0). The bit vector has the same size as the dictionary of tools. In the machine learning field, it is also known as a multi hot-encoded vector. Together, these 2 vectors form a training sample for the neural network. A pair of vectors are created in this manner for each tool sequence, and for all the tool sequences, they are combined to form 2 matrices, one for tool sequences and another for their respective labels. Internally, the label vectors have 2 subsets, one for shared labels (from the published, non-deleted and non-erroneous workflows) and another for non-shared labels (from the rest of the workflows). These matrices form input data to the neural network.

### Neural network architecture

**Figure 9:**
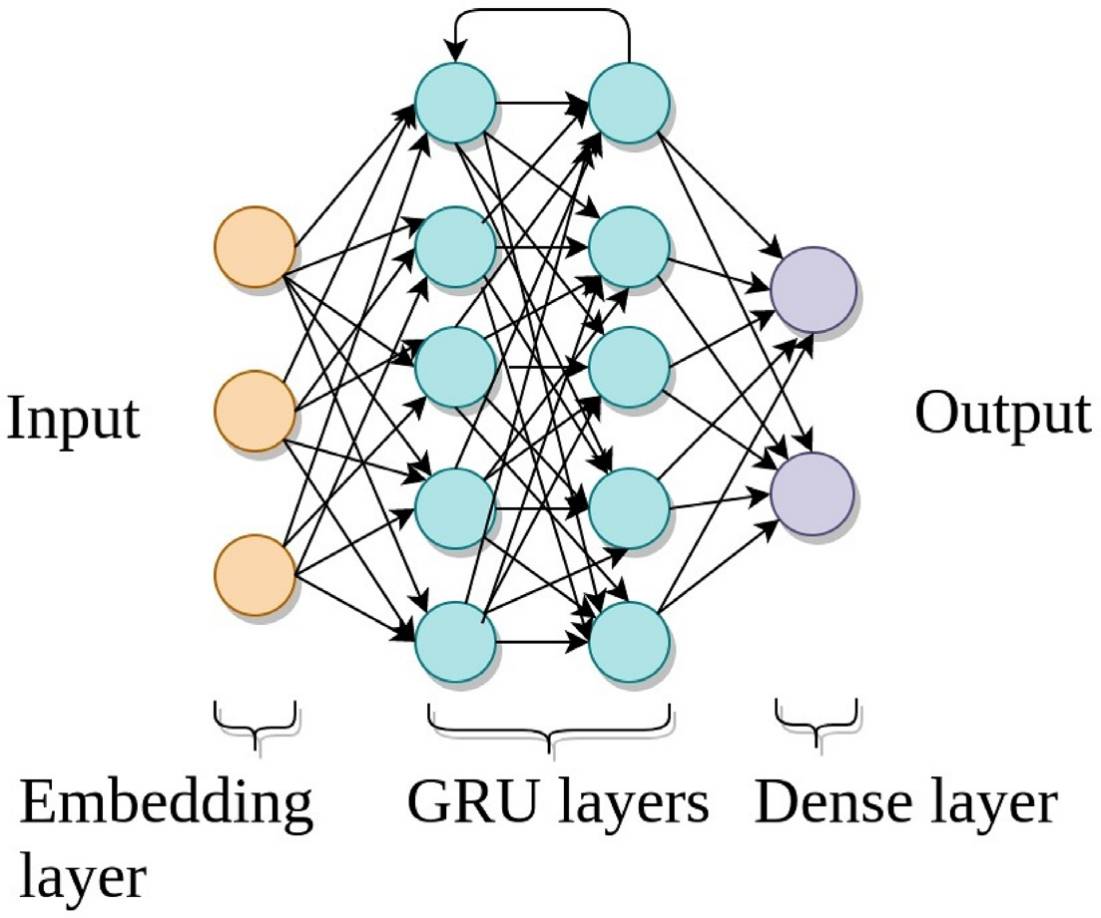
The figure shows the architecture of the GRU neural network. It has 4 components as layers. The first layer is the input layer (yellow), followed by 2 stacked layers of GRU (cyan), and the last layer is the output layer (lavender). The dropout layers are added between the embedding and GRU layers, between the 2 GRU layers, and between the second GRU and dense layers.

GRU neural network, a variant of RNN, is used for creating a model to recommend tools. The neural network architecture has multiple components such as different layers (Fig. 9), activation functions, class weights, loss function, and hyperparameter tuning technique, which are discussed in detail in the following paragraphs.

#### Embedding layer

The first component of the neural network architecture is an input layer (Fig. 9), which learns an embedding, a fixed-size vector, for each tool. This vector is used by the neural network as an internal representation of a tool. The embedding vector replaces the indices of tools in each tool sequence. The size of the embedding vector is fixed for all tools. For example, the vector of a tool sequence [12, 6, 75, 0, 0, ..., 0] is transformed into [[0.3, 0.01, 0.003, ..., 0.23], [0.5, 0.1, 0.005, ..., 0.9], [...], 0, 0, ...,0] by the embedding layer. The same embedding vector represents a tool in all tool sequences in which the tool is present.

#### GRU layer

The stacked layers of GRU learn deeper structures in the tool sequences by modelling the conditional probabilities of tools (labels) given all other prior tools (Fig. 9). GRU has certain advantages that help it to learn on sequential data. First, it avoids the problems of vanishing and exploding gradients that commonly occur in traditional RNN [63]. This is important because learning higher-order dependencies depends on the gradients of errors concerning the parameters (recurrent and input weight matrices) of GRU layers. Second, GRU has slightly fewer parameters than the long short-term memory network (LSTM), another variant of RNN, which makes using GRU simpler than LSTM. Finally, it achieves accuracy similar to that of LSTM [19].

#### Output layer

The last component of the neural network architecture is a dense layer that computes predictions (Fig. 9). The dimension of this layer is equal to the number of unique tools because it predicts a score for each tool (label). The predicted score of each tool is considered as its probability of being the label of an input tool sequence. The closer the predicted score of a tool is to 1, the more probable it is to be the recommended tool and the closer it is to 0, the less probable it is to be the recommended tool.

#### Dropout layer

Overfitting happens when a neural network performs exceptionally well on the training data but its performance on test (unseen) data remains poor. To minimize the effect of overfitting, a dropout layer is used between 2 layers of the neural network. It sets a few randomly chosen connections to 0 in the neural network to introduce some randomness to minimize overfitting [64, 65]. Three dropout layers are used in our approach, one between the embedding and the first GRU layers, one between 2 GRU layers, and the last between the second GRU and dense layers.

#### Activations

These are mathematical functions that are used in neural networks to transform inputs to a layer into its outputs. Two activations are used in our approach—one is exponential linear units (ELU) [66] and another is sigmoid (Equation 2). ELU is used for both the GRU layers and has a special feature of being negative when the input is negative, which allows mean activation (output) to get closer to 0 compared to other activation functions such as ReLU [67], which is always positive. As mean activations approach 0, the approximated and actual gradients get closer to each other. Therefore, using ELU in our neural network as an activation can be useful to achieve faster training, an improved reduction in loss, and better accuracy. Sigmoid is used in the output layer, which normalizes any real number to lie between 0 and 1, and this resulting quantity is considered the probability of each tool:

(2) \begin{equation*} f(x) = \frac{1}{1 + e^{-x}}. \end{equation*}

#### Usage frequencies of tools as weights

To incorporate the usage frequency of tools in the recommender system, the usage frequencies of all the tools used in the past 1 year have been collected and are used in the neural network training as the weights of tools. A tool that has been used often (e.g., Tool B in Fig. 2) in the past 1 year is assigned a higher weight than a tool (e.g., Tool C in Fig. 2) that has been used less often in the past 1 year. When tools are recommended a score is assigned to each tool by the neural network. It is expected that a tool with a higher weight gets a higher score and a tool with a lower weight gets a lower score. To summarize, the relevance of a tool to be used in a workflow decays if its usage decreases in Galaxy over time. This weighting scheme filters out tools from the list of recommendations that have not been used in the past 1 year irrespective of their origin, either shared or non-shared workflows.

Alternatively, the relevance of tools can also be ascertained by counting the occurrence of each tool in all workflows and these occurrences can be used as their weights in the neural network training. But, it may happen that some tools that were used often in the past to create workflows are not used anymore. Therefore, assigning weights to these tools in the neural network training based on their occurrences in workflows may not be a good indicator of their relevance and, overall, may not be optimal.

A curve is fitted through the usage frequencies of each tool using support vector regression (SVR) to display a trend of the usage of the tool over time. Using this trend, the usage of the tool for the next month is predicted and its logarithm is used as the weight for this tool in the neural network training. The logarithm of usage frequencies is computed to normalize them because only a few tools have a significantly large magnitude of usage compared to that of the remaining tools, which may lead the neural network to learn and predict only tools with a very large magnitude of usage and ignore other tools. Learning a trend for each tool involves 5-fold cross-validation and optimizing 2 hyperparameters of SVR, kernel and degree, using grid search. The values used for the kernel are “rbf," “poly," and “linear" and the values of degree used are 2 and 3. By following the grid search, there are 3 (kernels) × 2 (degrees) = 6 different combinations of hyperparameters to be verified to find the best curve for each tool [68].

#### Loss function

A neural network learns patterns from data by minimizing a loss function. Cross-entropy is a popular choice for a loss function in classification problems [69]. In our approach, the cross-entropy function is used in the GRU neural network to compute the loss between the true and predicted label and is weighted by the label’s weight. The loss is summed up over all labels of a tool sequence and then averaged (Equation 3). The term *T* is the total number of labels (size of the label bit vector). The term *w_i_* is the weight of the *i*^th^ label. The terms *p*^*a*^ and *p*^*b*^ refer to the true and predicted label vectors for a tool sequence, respectively. In general, the loss is large when *p*^*a*^ and *p*^*b*^ are far away from each other, which means that the learning by the neural network is not good. If they are close, the loss is low and the predictions are better. When an unweighted cross-entropy is used as the loss function for any classification problem [70], then it is assumed that all the predictions have the same weight and it does not differentiate between the more and less dominant labels. In our approach when it is used as a loss function in the neural network, then even though the predicted labels are correct they may not necessarily have large weights and thereby may be less relevant. Therefore, to reduce the possibility of less relevant labels appearing in recommendations, loss is weighted by the weights of labels. This means that if a label with a larger weight is misclassified, which means that the true and predicted values are different, then the overall loss is higher. In this way, the wrong classification of a label with a larger weight is penalized more than the wrong classification of a label with a smaller weight.

(3) \begin{equation*} \mathrm{loss} = - \frac{1}{T} \sum \nolimits_{i=1}^{T} \left[p_{i}^a \cdot \log (p^{b}_{i}) + (1 - p_{i}^a) \cdot \log (1 - p^{b}_{i})\right] * w_i
\end{equation*}

The loss in Equation 3 is computed for all tool sequences in training data and is minimized using a root mean square propagation (RMSProp) optimizer. It follows an adaptive approach to estimate the learning rate by keeping knowledge of gradients in prior iterations. The learning rate is updated by dividing it with an average of the square of the prior gradients [71].

#### Hyperparameter tuning

The hyperparameters in our approach are optimized using Bayesian (sequential model-based) optimization [72]. It learns from the previously evaluated configurations, which ensures faster convergence. Reasonable ranges of all the hyperparameters to be optimized are given and the best configuration is found after 20 evaluations. More details are given in section S7 of the supplementary document.

### Learning and predictions

More than 229,000 tool sequences collected from >18,000 workflows are divided into training and test data. A neural network learns patterns in the tool sequences from the training data and creates a model. The ability of the model to recommend tools is evaluated on the test data, which are unseen by the neural network during training. The training data form 80% (∼185,000) of all tool sequences and are iterated over 10 epochs of neural network training. The remaining 20% (∼45,000) is used as the test data. The running time of the training is ∼50 hours on a high-performance compute cluster provided by bwCloud [73] with multiple cores. After learning on the training data, the model is used to predict tools. Each predicted tool gets a probability score of being the recommended tool of a tool or tool sequence. Two sets of predictions are made—shared and non-shared. Each set is sorted in descending order of their probabilities and the top ones in both sets are combined to show them as recommendations.

## Summary and Future Work

A system to recommend tools in Galaxy is built by analysing workflows using a variant of RNN (GRU) and a weighted cross-entropy loss function. The recommended tools are relevant for multiple scientific analyses with high accuracy as shown by the high similarities between the tools used in Galaxy Training Network tutorials and the recommended tools for similar analyses (Table 1). Moreover, they are easily accessible through simple UI integrations in Galaxy (Figs 6 and 7). Collectively, they improve user experience by helping researchers to easily create correct workflows. In addition, the approach does not store any information about tools and the recommendations are made by learning only the patterns of tool connections in workflows. The model [74] created using this approach and an API [75] are integrated into the European Galaxy server. The API resides with other Galaxy APIs and accesses a tool or a tool sequence specified by researchers to show its recommendations in real time using the model. The API is used at 2 different user interfaces in Galaxy—one shows recommendations in the workflow editor (Fig. 6) and another shows them after each tool execution (Fig. 7). The recommendation system should be potentially helpful for researchers who are new to the Galaxy platform. It shows them a few follow-up tools from a big collection of >3,000 tools and enables them to perform multiple exploratory data analyses.

Different Galaxy servers maintain different sets of tools and workflows. The present approach can be used to create different recommendation models for different Galaxy servers. Alternatively, all the workflows can be collected from multiple Galaxy servers and using the present approach, 1 recommendation model can be created by learning on the complete set of workflows and the model can be distributed to different Galaxy servers. To improve the quality of recommendations, the annotations of tools can be incorporated in the learning mechanism by assigning higher weights to the annotated tools in comparison to tools that are not annotated. Tools containing similar annotations may have similar functionalities, and using these similarities, tool recommendations can be further enhanced by showing similar tools for each recommended tool. In addition to learning tool connections to recommend tools, the knowledge of tools connecting to different tools based on their respective parameters can also be incorporated.

## Methods

### Library, model, and code repositories

The Keras deep learning library is used for producing the neural network architectures [76]. The trained model [74] is saved as an H5 file to simplify its distribution to different Galaxy instances (Galaxy, RRID:SCR_006281). The file is an HDF5 store containing the weights of different layers of the neural network and their configurations, a dictionary of tools and their indices, and the weights of tools. The weights and configuration of the neural network are needed to recreate the trained model. The dictionary is used to replace IDs of the predicted tools by their indices in a tool sequence. All data and python scripts used in our approach are stored at GitHub for all approaches—GRU [77], CNN [78], and DNN [79]. In each of these repositories, the process to create a tool recommendation model is explained. All these repositories are provided with a script (“extract_data.sh”) for collecting raw input datasets from a Galaxy instance. These datasets are workflows and usage frequencies of tools and are also provided in each repository. The values of multiple hyperparameters of neural networks, number of training iterations, and sizes of training and test data can be altered using a bash script (“train.sh"). To execute the scripts on a GPU-enabled machine, the “tensorflow-gpu" package should be installed instead of “tensorflow" as mentioned in the conda package dependencies file (“environment.yml"). To see recommended tools, an ipython script (“tool_recommendation_gru_wc.ipynb" for GRU repository) is also provided that loads and recreates a trained model to predict tools for a tool or a tool sequence. The result files storing precision, training, and validation losses and usage frequencies, which are used for generating line plots (Figs 3–5), for all approaches are also available at GitHub [80]. The code repositories of 2 other approaches that do not use neural networks are available at simple approach [31] and ExtraTrees [32].

### New recommendation model

On a usual Galaxy server, tools and workflows are dynamic as they are added and updated regularly. Therefore, it is important to train the GRU neural network on the complete set of workflows periodically to keep the tool recommendation model updated with the latest tools and workflows. Using a Galaxy tool [81], a new recommendation model can be created after collecting workflows and tool usage data from a Galaxy server. The tool runs for several hours (>24 hours) and creates a model, which is pushed to an online repository [74]. From this repository, Galaxy downloads it using an API [75] to recommend tools. The recommendation model is created periodically every 3–4 months to accommodate new workflows and tools. Galaxy administrators can decide upon the frequency of creating a new model. It can be created every month or every 6 months.

### New tools as recommendations

Galaxy administrators can overwrite the recommended tools predicted using the trained model by a different set of tools using the configuration option described by Kumar [82]. In addition, to highlight the newly added tools, which are not part of the model, they can be appended to the recommendations using this additional configuration option.

## Availability of Supporting Source Code and Requirements

Project name: Tool recommender in Galaxy using deep learning

Project home page: https://github.com/anuprulez/galaxy_tool_recommendation

Operating system: Linux

Programming languages: Python, XML, JavaScript

Other requirements: Tensorflow, Keras, Scikit-learn, Numpy, H5py, Csvkit, Hyperopt

License: MIT License

RRID:SCR_018491

Biotools ID: tool_recommender_system_in_galaxy

## Data Availability

A snapshot of the source code is available in the *GigaScience* GigaDB repository [83].

## Additional Files

Supplementary Table 1. Comparison of recommendations between the GRU neural network, a simple model, and ExtraTrees classifier.

Supplementary Table 2. Comparison of recommendations between the regularized and non-regularized GRU neural network.

Supplementary Table 3. The strategy of uniform sampling of training data.

Supplementary Figure 1. Architecture of convolutional neural network (CNN) used in the article.

Supplementary Figure 2. Architecture of dense neural network (DNN) used in the article.

Supplementary Figure 3. Original frequencies of last tools in training data.

Supplementary Figure 4. The frequencies of last tools in training data after uniform sampling.

Supplementary Figure 5. Top-1 non-shared precision for less frequent tools in test data.

Supplementary Figure 6. Top-1 shared precision for less frequent tools in test data.

Supplementary Figure 7. Top-1 and top-2 precision (non-shared and shared recommendations) of the ExtraTrees classifier for test data.

## Abbreviations

API: application programming interface; BWA: Burrows-Wheeler Aligner; CNN: convolutional neural network; DNN: dense neural network; EDAM: EMBRACE Data And Methods; ELU: exponential linear units; GPU: graphics processing unit; GRU: gated recurrent units; LSTM: long short-term memory network; PROPHETS: Process Realization and Optimization Platform Using Human-Readable Expression of Temporal-Logic Synthesis; RNN: recurrent neural network; SVR: support vector regression; UI: user interface; WINGS: workflow instance generation and specialization.

## Competing Interests

The authors declare that they have no competing interests.

## Funding

This work was supported by the German Research Foundation (DFG) under Germany’s Excellence Strategy (CIBSS - EXC-2189 - Project ID 390939984) and German Federal Ministry of Education and Research (BMBF grant 031A538A de.NBI). The article processing charge was funded by the University of Freiburg in the funding programme Open Access Publishing.

## Authors’ Contributions

A.K. implemented the project and wrote the manuscript. H.R. wrote scripts for data collection, contributed to the manuscript, and deployed the project on the European Galaxy server. B.G. provided the idea of the project, validated results, and contributed to the manuscript. R.B. contributed to the manuscript. All authors approved the manuscript.

## Supplementary Material

giaa152_GIGA-D-20-00053_Original_Submission

giaa152_GIGA-D-20-00053_Revision_1

giaa152_GIGA-D-20-00053_Revision_2

giaa152_GIGA-D-20-00053_Revision_3

giaa152_Response_to_Reviewer_Comments_Original_Submission

giaa152_Response_to_Reviewer_Comments_Revision_1

giaa152_Response_to_Reviewer_Comments_Revision_2

giaa152_Reviewer_1_Report_Original_Submission Bernie Pope, Ph.D. -- 3/9/2020 Reviewed

giaa152_Reviewer_1_Report_Revision_1 Bernie Pope, Ph.D. -- 8/19/2020 Reviewed

giaa152_Reviewer_2_Report_Original_Submission Jeremy Leipzig -- 3/31/2020 Reviewed

giaa152_Reviewer_3_Report_Original_Submission Katy Wolstencroft -- 4/2/2020 Reviewed

giaa152_Reviewer_3_Report_Revision_1 Katy Wolstencroft -- 9/7/2020 Reviewed

giaa152_Reviewer_4_Report_Original_Submission MatÃoÅ¡ KalaÅ¡ -- 4/20/2020 Reviewed

giaa152_Supplemental_Files
