# Supplementary material for: Tool recommender system in Galaxy using deep learning
Source: Gigascience. 2021 Jan 6;10(1):giaa152. doi: 10.1093/gigascience/giaa152 (PMC7786169; doi:10.1093/gigascience/giaa152)
Supplement: giaa152_GIGA-D-20-00053_Revision_1 [file giaa152_giga-d-20-00053_revision_1.pdf]

GigaScience

Tool recommender system in Galaxy using deep learning

--Manuscript Draft--

**Manuscript Number:**	GIGA-D-20-00053R1
**Full Title:**	Tool recommender system in Galaxy using deep learning
**Article Type:**	Technical Note
**Funding Information:**	DFG (German Research Foundation) (CIBSS - EXC-2189 - Project ID 390939984) — Dr. Rolf Backofen<br>BMBF grant (de.NBI) (031A538A) — Dr. Björn Grüning
**Abstract:**	Background Galaxy is a web-based and open-source scientific data-processing platform. Researchers compose pipelines in Galaxy to analyse scientific data. These pipelines, also known as workflows, can be complex and difficult to create from thousands of tools, especially for researchers new to Galaxy. To help researchers with creating workflows, a system is developed to recommend tools that can facilitate further data analysis. Findings A model is developed to recommend tools using a deep learning approach by analysing workflows composed by researchers on the European Galaxy server.The higher-order dependencies in workflows, represented as directed acyclic graphs, are learned by training a gated recurrent units (GRU) neural network, a variant of a recurrent neural network (RNN). In the neural network training, the weights of tools used are derived from their usage frequencies over time and the sequences of tools are uniformly sampled from training data. Hyperparameters of the neural network are optimised using Bayesian optimisation. Mean accuracy of 98% in recommending tools is achieved for the top-1 metric. Conclusions The model is accessed by a Galaxy API to provide researchers with recommended tools in an interactive manner using multiple user interface (UI) integrations on the European Galaxy server. Good quality and highly-used tools are shown at the top of the recommendations. The scripts and data to create the recommendation system are available under MIT license at https://github.com/anuprulez/galaxy_tool_recommendation.
**Corresponding Author:**	Anup Kumar<br>Albert-Ludwigs-Universitat Freiburg<br>Freiburg, GERMANY
**Corresponding Author Secondary Information:**	
**Corresponding Author's Institution:**	Albert-Ludwigs-Universitat Freiburg
**Corresponding Author's Secondary Institution:**	
**First Author:**	Anup Kumar
**First Author Secondary Information:**	
**Order of Authors:**	Anup Kumar<br>Helena Rasche<br>Björn Grüning<br>Rolf Backofen
**Order of Authors Secondary Information:**	
**Response to Reviewers:**	GIGA-D-20-00053<br>Tool recommender system in Galaxy using deep learning<br>Anup Kumar; Helena Rasche; Björn Grüning; Rolf Backofen<br>GigaScience<br><br>Dear Anup,

Powered by Editorial Manager® and ProduXion Manager® from Aries Systems Corporation

Your manuscript "Tool recommender system in Galaxy using deep learning" (GIGA-D-20-00053) has been assessed by our reviewers. While it is of interest, we are unable to consider it for publication without some additional work. The reviewers have raised a number of points which we believe would improve the manuscript and would allow a revised version to be published in GigaScience. Particularly regarding the need for some real-world examples.

Author response:

We highly appreciate the suggestions provided by the editor and all the reviewers and have addressed all the review comments. A few sections have been added and modified in the paper and they are accordingly referenced in the responses. Moreover, we have provided real-world examples of tool recommendations for many tools and tool sequences from multiple scientific analyses in the paper (Table 1) and in the supplementary document (Supplementary table 1 and 2) in addition to figures 6 and 7.

Their reports, together with any other comments, are below. Please also take a moment to check our website at https://www.editorialmanager.com/giga/ for any additional comments that were saved as attachments.

In addition, please register any new software application in the bio.tools and SciCrunch.org databases to receive RRID (Research Resource Identification Initiative ID) and biotoolsID identifiers, and include these in your manuscript. This will facilitate tracking, reproducibility and re-use of your tool.

Author response:

We have received RRID (SCR_018491) for the project and also registered it on bio.tools (URL: https://bio.tools/tool_recommender_system_in_galaxy).

If you are able to fully address these points, we would encourage you to submit a revised manuscript to GigaScience. Once you have made the necessary corrections, please submit online at:

https://www.editorialmanager.com/giga/

If you have forgotten your username or password please use the "Send Login Details" link to get your login information. For security reasons, your password will be reset.

Please include a point-by-point within the 'Response to Reviewers' box in the submission system. Please ensure you describe additional experiments that were carried out and include a detailed rebuttal of any criticisms or requested revisions that you disagreed with. Please also ensure that your revised manuscript conforms to the journal style, which can be found in the Instructions for Authors on the journal homepage. If the data and code has been modified in the revision process please be sure to update the public versions of this too.

The due date for submitting the revised version of your article is 20 Jul 2020.

I look forward to receiving your revised manuscript soon.

Best wishes,

Scott Edmunds, Ph.D.
GigaScience
www.gigasciencejournal.com

Reviewer reports:

Powered by Editorial Manager® and ProduXion Manager® from Aries Systems Corporation

Reviewer #1: This paper describes a tool recommendation system for Galaxy workflows that employs a GRU neural network to train a classifier system. The classifier is trained on existing workflows that are recorded in the Galaxy system, and usage frequency of tools is additionally used to weight their relevance to the result.

Author response:

Thank you for your review comments. Please find their responses below.

This paper was submitted under the "Research" manuscript type, but I wonder whether it would have been better as a Technical note, given that the paper describes an implemented system, but there is no tested hypotheses or research question being answered.

Author response:

The manuscript has been reformatted as a technical note.

There are some interesting ideas explored in this paper but I have some major concerns about its current presentation:
- The solution seems like it might be overkill for the problem that is being solved. The authors have not implemented or compared to any alternative approaches to solving the same problem, so we have no basis to understand whether a NN is needed for this problem, or whether a simpler statistical model would suffice.

Author response:

We have compared the proposed approach with a simple model and ExtraTrees classifier and added the following text to the section S1 of the supplementary document which compares the performance of the proposed approach with these two other approaches.

“To compare the performance of the GRU neural network with approaches without any use of the neural networks, two ideas are explored - a simple approach to store all the indices of sequences of tools (https://github.com/anuprulez/galaxy_tool_recommendation/tree/statistical_model) and the other one uses ExtraTrees classifier (https://github.com/anuprulez/galaxy_tool_recommendation/tree/sklearn_rf).
The simple approach to recommend tools is implemented by storing the indices of tool sequences extracted from over 18,000 workflows on the European Galaxy server. The size of the resulting model (simple model) is 46 MB while its size created using the GRU neural network is only 6 MB. As the number of workflows grows in future, the model size will grow too, posing limitations in storing and sharing it over online platforms such as Galaxy. Therefore, to limit the size of the model, machine learning and deep learning methods are more suitable where it is not required to store any data. Instead, it is sufficient only to store the weights of features in data. Moreover, the GRU neural network recommends tools similar to the ones recommended by the simple model (Table 1 in supplementary section S2) for multiple scientific analyses. In addition, ExtraTrees, an ensemble based classifier, is also trained on tool sequences to recommend tools. The optimisation of hyperparameters such as the number of estimators, depth of trees and so on and the uniform sampling of the training data were done following the same approach as used for training the GRU neural network. The model trained with the ExtraTrees classifier achieves a precision of less than 0.5 for the top-1 metric (Supplementary Figure 7) which is very low compared to the accuracy of the GRU neural network (0.98 top-1 precision). It shows that the ExtraTrees classifier is unsuitable for learning on sequential data as used in this project. Moreover, the peak memory usage for the ExtraTrees classifier is approximately 75 GB while for the GRU neural network it is approximately 8 GB. In summary, large model size of the simple model and low precision and high memory usage for the ExtraTrees classifier

Powered by Editorial Manager® and ProduXion Manager® from Aries Systems Corporation

do not qualify them as good approaches to create the tool recommendation system. On the other hand, the GRU neural network has a smaller model size and memory usage and higher precision which collectively make it a better approach to create the tool recommendation system."

- The paper does not address the issue of whether the tool is really useful in practice. For example it says "they [recommendations] improve user experience by helping researchers to easily create correct workflows". This claim is not tested in this paper. You would need to test user behaviour to find out, or in the very least, survey user experience with the tool.

Author response:

Table 1 in the paper lists a few standard (tool connections in published and non-deleted are marked as standard and they are of good quality) and normal (tool connections from other workflows) recommended tools for multiple tool sequences in different scientific analyses such as Computational chemistry, Epigenetics, Machine learning, Proteomics, RNA sequencing analysis and a few others. The recommended tools shown in this table are highly used for standard scientific analyses as highlighted in multiple GTN training materials (https://training.galaxyproject.org/) which shows that the recommended tools are of good quality and highly used. Moreover, this project has been deployed on the European Galaxy server for the last 12 months and has received very positive user-feedback since then. It was presented as a live demonstration at the Galaxy community conference (GCC) 2019 and at the Automatic workflow composition workshop (March 2020). At both events, positive feedback was received. In addition, the code has been accepted upstream by the Galaxy community and is already part of the main Galaxy codebase (release 20.05) because it was considered to be an important addition for the Galaxy users. A couple of reviewers of this paper seem to have tested it and found it to be good and intuitive. However, there is no easy way to monitor user's behavior regarding the usefulness of recommendations. We are in contact with the cognitive science department of our university, who are supporting us with the user experience (UX) questions. In summary, we are confident that such a recommendation system can assist Galaxy users in choosing good quality tools for different analyses.

- Such a recommendation system us potentially dangerous if it is giving poor/biased/incorrect suggestions to users, and is highly sensitive to the training data. It is not clear from the paper how biases in the training data are dealt with. For example, suppose the Galaxy server is used by students undergoing training. It could be the case that for training purposes students are taught initially to use older, out of date, tools and techniques, for the sake of simplicity. There could be very many of these students on the system. A large number of students using out-of-date techniques could seriously bias the results. The paper suggests re-training the system periodically, which is reasonable, however, how does the system protect against circular dependencies in the data, where it starts training on workflows that have used the recommendation system themselves?

Author response:

We have divided the tool connections into standard and normal connections. Standard connections are tool connections coming from the published and non-deleted workflows which are of good quality while the normal ones are from other workflows. Recommendations are also divided into standard and normal ones and the standard ones are shown at the top of the list of recommendations. We achieve high accuracy (top-1 precision = 0.98) for the standard and normal recommendations. Table 1 in the paper suggests that the system predicts useful tools which appear in diverse standard scientific pipelines mentioned in Galaxy training materials. These materials use the latest tools which are used for training purposes around the world and they are regularly maintained. To avoid old tools appearing in the list of recommendations, we have implemented a whitelist (overwrite.yml) where Galaxy admins can overwrite

Powered by Editorial Manager® and ProduXion Manager® from Aries Systems Corporation

deprecated tools. Training data (workflows) can get biased, for example, when lots of students are using old tools to create workflows for multiple scientific analyses as asked in the review. Therefore, to minimise the problem occuring due to the biases, we performed uniform sampling of the data before feeding it to the neural network. “Uniform sampling” section has been added to the paper. Please find the corresponding text from the paper below.

“Workflows in Galaxy come from different scientific analyses. It may happen that the number of workflows from these analyses are not comparable - some analyses may have a large number of workflows while some may have only a small number of workflows. This can cause some tools to be present very frequently in workflows while other tools are less frequent. Learning on these workflows and recommending tools may exhibit bias by showing better recommendations for the frequently occurring tools and poorer recommendations for the less frequent ones. To showcase this imbalance, the frequencies of the last tool in each tool sequence in training data are calculated and it is found that only a few tools have large frequencies and most of the tools are present in low frequencies (Supplementary Figure 3). For example, the tools with very high frequencies (> 10,000) are “Concatenate datasets”, “Cut”, “Grouping” and “Join” while the tools having very low frequencies (< 5) are “Cluster inspection using RaceID”, “rDock cavity definition” and “ChiRA collapse”. Therefore, to overcome this drawback, the training data created after extracting tool sequences should be balanced in order to make the neural network learn on a similar number of tool sequences from different scientific analyses in each training iteration. To implement this strategy, a set of last tools in all tool sequences from the training data is collected. Further, for each tool in this set, a list of indices of tool sequences in the training data are stored for which it is the last tool (Supplementary Table 3). Only the last tools are considered for implementing this strategy because of two reasons. First, the smallest tool sequences contain only two tools and second, all tools become the last tool in at least one tool sequence and the computed frequencies of these last tools suggest their overall frequencies in the training data. In the neural network training, for each iteration (which consumes all tool sequences in the training data), small batches containing equal number of tool sequences are created. For example if batch size is 100 and the size of training data is 2000, then 20 (2000/100=20) batches are created, each containing 100 tool sequences. In each batch, 100 tools from the set of last tools are uniformly selected (Column 2 in Supplementary Table 3) and for each selected tool, a tool sequence is chosen uniformly from its respective list of tool indices (Column 3 in Supplementary Table 3). After selecting tool sequences for many batches for each iteration of training (epoch), it is expected that all the tools from the set of last tools and their respective tool sequences are chosen. Performing this uniform selection of different tool sequences for each iteration, the training data becomes balanced. Supplementary Figure 4 shows that each last tool is present approximately 1670 times on an average in each iteration (epoch). The order of tools in the Supplementary Figures 3 and 4 are same.”

Due to the uniform sampling, good precision has been achieved for the tool sequences with low frequencies too. In summary, the recommendation of good quality and highly used tools should mitigate the problem of circular dependency. Please see section S5 of the supplementary document for more details.

- The system considers tools, but apparently not data types. In the example discussed on page 4, how would the system know to recommend RNA-STAR compared to BWA or Bowtie if it doesn't know whether the inputs are DNA or RNA?

Author response:

Currently, Galaxy does not differentiate between RNA and DNA as both are FASTA files. This behaviour is in Galaxy and does not arise from the tool recommendation system. If there are tools such as RNA-FASTA or DNA-FASTA in Galaxy, this issue would be solved. However, the recommendation system uses the knowledge of data types of tools to filter out the incompatible recommendations as a post-processing step (after recommending tools using the trained model). The recommendations shown in the Galaxy UI for a tool or a sequence of tools have compatible data types with the last

Powered by Editorial Manager® and ProduXion Manager® from Aries Systems Corporation

tool it connects to. The recommendations with incompatible data types are not shown.

Beyond those concerns above, I feel that the paper is imbalanced in its structure. Too much space is used to discuss general features of NNs, whereas too little attention is paid to the actual methods used in the project. For example, the actual size and nature of the training data is only very briefly mentioned near the end of the paper "The number of tool sequences extracted from workflows is approximately 200,000..." This is a very important detail that deserves more attention. We don't know the proportions of the training workflows for different kinds of analyses, and therefore it is hard to say anything about selection bias in the data. What if 90% of the training data is for DNA sequencing, how would this affect the ability of the system to recommend proteomics tools for example?

Author response:

The “Data description” section in the paper has been extended to include information about workflows. In addition, we have added a “Uniform sampling” section in the paper that explains how the imbalance in the data is solved. More details about uniform sampling are given in section S5 of the supplementary document. Uniform sampling makes it possible that the neural network trains on tool sequences from workflows in multiple scientific analyses in roughly equal number of times. Therefore, even if 90% of training data is for DNA sequencing and other analyses form only 10% of all data, the neural network will train on different sequences of tools almost equally. Supplementary Figures 5 and 6 show that the precision (standard and normal) achieved for the less frequent tool sequences are also good (90%). In addition, every Galaxy instance will have its own set of tools and workflows. Therefore, it has been made possible to create different recommendation models ideal for different Galaxy instances by using a Galaxy tool - “Create a model to recommend tools”.

Reviewer #2: The authors present a Galaxy tool recommendation system based on a training set of over 200,000 tool sequences from existing workflows. The final version of this system uses a GRU neural network. Although extensive testing was done to compare and benchmark this model with other neural networks, it should be noted there is no existing conventional approach (Markov Model/market basket/Bayesian) but I don't feel that it is necessary to create a straw man for this purpose. The neural network offers a high degree of accuracy without the need to manually annotate each tool.

The description of the GRU implementation, testing, and optimization is very good and will inform readers about how to implement similar solutions for other applications. The Github repository is also well organized. This paper is well suited to this journal and should be accepted with minor revisions.

Author response::

Thank you for the appreciation and for your review comments. Please find their responses below.

I have three minor revisions to suggest:
1. I think most readers would understand what overfitting would look like in a typical machine learning, but maybe not in a recommendation engine for tools. What noise or error that is propagated by an overfit recommendation engine? For instance, would it resemble obscure tools that some edge case user chose? More importantly, what does the regularization step actually do in this case - recommend a repertoire of more common tools, or simply remain agnostic?

Author response:

Powered by Editorial Manager® and ProduXion Manager® from Aries Systems Corporation

The following text has been added to section S4 of the supplementary document.

The use of dropout in the GRU neural network for regularisation allowed the model to have smaller weights compared to the non-regularised GRU neural network model. Overall mean of weights (weights of all layers) for the regularised model is 0.0755 while for the non-regularised model, it is 0.0962. Having larger weights in a neural network model may lead to overfitting as it is an indication of a more complex network. In an overfit model, the neurons (neural network units) in a layer try to fix the errors made in the previous layers to make the model robust (on the training data) and may not learn more general features. One of the advantages of using regularisation in a recommendation engine can be to avoid the following situation - an overfit model may try to learn the most common tools with higher usage frequency to minimise the error (due to the weighted cross-entropy loss function) and may ignore tools with lower usage, but important ones. For example, the regularised model recommends the following tools for the “UMI-tools count” tool. The (log) usage frequency has been shown beside each tool name.

-Text transformation with sed (3.98) [Standard recommendation]
-Column Join on Collections (4.78) [Normal recommendation]
-Initial processing using RaceID (3.68) [Normal recommendation]
-Transpose rows/columns (3.57) [Normal recommendation]
-Seurat (2.67) [Normal recommendation]

The non-regularised model also recommends the same tools except “Seurat” tool which has the lowest usage frequency out of all recommended ones.

Provide a real-world example, something like…

"An example of overfitting in our recommendation system may involve tools with few use cases (e.g. tools for dealing with organisms with no reference assembly). The GRU would learn only from a limited training set and which could recommend tools that would not be appropriate for most users."

Author response:

We have added a table comparing the recommendations made by the regularised and non-regularised models to the section S4 of the supplementary document. Please find the relevant text from section “Benefit of regularisation” in the paper below.

“Using regularisation minimises overfitting by assisting the GRU neural network to make better recommendations by predicting tools having low usage frequencies but useful in addition to tools with high usage frequencies. For example, the recommendations of "UMI-tools count" tool with the regularised model include "Seurat" tool which is absent from recommendations by the non-regularised model. Another example is for "RaceID, Lineage computation using StemID" tool sequence which gets "Lineage Branch Analysis using StemID" tool as one of the recommendations by the regularised model while there is no recommendation at all by the non-regularised model. The recommendations for a popular mapper, RNA-STAR, are "featureCounts", "MultiQC", "Infer Experiment" and few others by both the models. But, in addition to these recommendations, the regularised model recommends the "Read Distribution" tool which is not predicted by the non-regularised model. More details have been provided in section S4 of the supplementary document.”

2. It appears the recommendation engine cannot recommend entirely new tools without completely rebuilding the model. Please explain how often the model is rebuilt in practice. Also explain how new tools would ever be recommended if they don't appear in existing workflows. Is the design that entirely new tools would be adopted into workflows by "power users" and therefore trickle down to more casual Galaxy users?

Author response:

Powered by Editorial Manager® and ProduXion Manager® from Aries Systems Corporation

The recommendation model for usegalaxy.eu is currently created periodically every 3-4 months to accommodate new workflows and tools. Galaxy admins can decide upon the frequency of creating a new model. It can be created every month or every 6 months. In addition, there is a provision to add and overwrite tool recommendations in the Galaxy API by Galaxy admins to promote newly added tools and deprecate old tools, respectively for all Galaxy users including power and casual ones. We created a tool to make it easier to recreate the training model with the latest tools and workflows by creating a Galaxy tool - “Create a model to recommend tools”. Using this tool, a new recommendation model can be created after collecting workflows and tool usage data from any Galaxy server. The tool runs for several hours (> 24 hours) and creates the recommendation model. It can be downloaded from Galaxy and uploaded to Galaxy’s test data repository (https://github.com/galaxyproject/galaxy-test-data) by Galaxy admins. From this repository, Galaxy downloads it using the API to recommend tools.

3. DESeq2 is miscapitalized as DeSeq2 on pg2

Author response:

Thank you for spotting this mistake. We have corrected it in the paper.

Reviewer #3: This paper describes an interesting and novel approach for learning from the large numbers of existing workflows in Galaxy. Using a neural network approach to learn which tools are used most frequently, and which tools are used in sequence, is a good basis for building a recommender system. The number of available Galaxy workflows provides a large test set to work with and spans a wide range of bioinformatics analyses. The recommender has already been embedded into the Galaxy.eu server. This increases the significance of the work as it means that a large user community can already benefit from the recommendations.

Author response:

We appreciate your positive feedback and thank you for your review comments. Please find their responses below.

Major Points

1. The paper would benefit from a deeper discussion about the quality and validity of the workflows in the test set. Are all workflow used, or only those that run without generating an error? There are many workflows created during exploration that are never used for an eventual analysis, either because they are considered unsuitable by their creator, or because they simply did not run. How are these 'test' workflows separated from 'production' workflows that are used to generate actual results?

Author response:

We have collected over 18,000 workflows from the European Galaxy server and extracted over 229,000 paths (sequences of tools) from them which are used for training a neural network (Gated recurrent units). All these paths are divided into training and test sets. The training set is used for training and the trained recommendation model is evaluated on the test set. To address the quality of the workflows, the connection of tools within workflows are separated based on their quality - the connections coming from the non-deleted and published workflows are marked as “standard” or “good” connections and the rest of all other tool connections are marked as “normal” connections. The recommendations are also separated as standard and normal. Standard recommendations (good quality) are promoted to the top of the list of recommendations followed by normal recommendations. In addition,

the recommendation model can be created ideal for different Galaxy instances by using respective tools and workflows by Galaxy admins. Moreover, a whitelist of tools can be provided by Galaxy admins using the config option to overwrite the recommendations made by the neural network if needed.

2. Related to the point above - is there an attempt to collect and learn from tool parameter information in addition to the tool function? Some tools perform multiple functions and therefore behave very differently depending upon their configuration.

Author response:

Currently, we are recommending tools based on tool connections in workflows. In addition to learning from tool connections, recommending tools based on the configurations of tools would be in our future enhancement of the tool recommendation system and it has been mentioned in the “Summary and future work” section of the paper too.

3. The recommender system is based on suggesting the next tool, but could the recommender system also be used to suggest whole configured workflows as the next step? If a collection of tools are always used together, suggesting one at a time (without parameter information) would be less accurate overall than suggesting a fully configured workflow for all the subsequent steps.

Author response:

Recommending a set of tools at each step provides more flexibility to users to diverge into multiple analyses using different tools enabling users to explore many different tools. However, predicting complete workflows may lose many different variations in which a tool can be used. Moreover, there is already a provision for sharing workflows in Galaxy. Also, many workflows are already available for multiple analyses over Galaxy Training Network (https://training.galaxyproject.org/). It may be possible to recommend complete workflows, but currently we do not aim to predict complete workflows in this work.

4. Recommendations are based on tool use frequency data and previous workflows. If a new tool is developed, there will be a lag before this tool is recommended to users, even if it is 'better' than similar tools. How could this problem be addressed? Similarly, using recommendations may lead to less innovation in workflow creation. Could the authors discuss the implications of this (i.e. who is the recommender for? Is it for the workflow expert, or for users who need more bioinformatics support?)

Author response:

Galaxy admins can overwrite the recommended tools predicted using the trained model by a different set of tools using a configuration option by adding different tools. In addition, newly added tools, which are not part of the model, can be appended to the recommendations using this additional configuration option. Moreover, every 3-4 months, a new recommendation model trained on the latest data will be made available on the EU Galaxy server. Galaxy admins can decide upon the frequency of creating a new model. It can be created every month or every 6 months. Admins of each Galaxy instance can create a recommendation model built using the tools and workflows on that instance. The frequency of model creation can also be managed by Galaxy admins. The question of implication of the recommendation system is briefly discussed in the “Summary and future work” section of the paper. Please find the relevant text from the paper below.

“The recommendation system should be potentially helpful for those researchers who are new to the Galaxy platform. It shows them a few follow-up tools from a big

Powered by Editorial Manager® and ProduXion Manager® from Aries Systems Corporation

collection of more than 3,000 tools and enables them to perform multiple exploratory data analyses."

6. Have the authors performed a user evaluation on their recommender system? It is already part of Galaxy.eu and the manuscript claims it "improves user experience by helping researchers
to easily create correct workflows". However, there are no details of current usage or of how this has been assessed to date. As it is available, I have used it, and it is very intuitive, but the paper would be strengthened by adding a more formal evaluation of usage to date.

Author response:

This project has been deployed on the European Galaxy server for the last 12 months and has received very positive user-feedback since then. It was presented as a live demonstration at the Galaxy community conference (GCC) 2019 and at the Automatic workflow composition workshop (March 2020). At both events, positive feedback was received. In addition, the code has been accepted upstream by the Galaxy community and is already part of the main Galaxy codebase (https://github.com/galaxyproject/galaxy/tree/release_20.05) because it was considered to be an important addition for the Galaxy users. Table 1 in the paper suggests that the recommendation system predicts useful tools which appear in diverse standard scientific pipelines mentioned in Galaxy training materials (https://training.galaxyproject.org/). These materials use the latest tools which are used for training purposes around the world and they are regularly maintained. However, there is no easy way to monitor user's behavior regarding the usefulness of recommendations. We are in contact with the cognitive science department of our university, who are supporting us with user experience (UX) questions. In summary, we are confident that such a recommendation system can assist Galaxy users in choosing good quality tools for different analyses.

Minor point

In the results section, the example workflow is based on NGS sequence analysis (Trimmomatic --> Bowtie2 --> FreeBayes). This is a good example, however, in the text, the authors suggest that after bowtie2, FastQC might be recommended. Usually, FastQC would be used before Trimmomatic.

Author response:

Thank you for spotting it. We have updated the text in the paper.

Reviewer #4: Dear authors and editor(s),

After seeing a conference presentation and the tool recommender in usegalaxy.eu last summer, and noting the bioRxiv preprint of this work last November (https://doi.org/10.1101/838599), I am happy seeing that authors submitted the manuscript to GigaScience. Compared to the preprint, the text has been polished and better structured into sections.

I find the manuscript very nicely written and relatively easy to read (with only minor opportunities for editorial polishing, some mentioned below). As a reader, I highly appreciate the effort of the authors towards making the text reader-friendly. I find it also clear from the text, how the data was collected and curated, and where it is openly published. The manuscript also points to the used source code (scripts), and all is available together under a permissive open source software license.

I did not have the capacity to test the various scripts - for extracting data and all of the models training - but I repeatedly tested the user interface on the usegalaxy.eu server, useful from the user's point of view.

I'm very happy with the content and the results of this work, and the manuscript convinces me about the choices taken in the authors' approach. I don't recommend a revision of the work. My following suggestions are only about the code repository and some minute, mostly editorial improvements of the text, plus a couple of brief comments.

Author response:

We appreciate your positive review and thank you for your review comments.

Repositories and branches:

This manuscript links to a repository containing all the used code and data (https://github.com/anuprulez/galaxy_tool_recommendation), created in February 2020 by a mass upload of most of the code and data. I am really happy to see that some updates have been pushed to this repo in the recent weeks of April 2020.

The history of the work is recorded in another repo (https://github.com/anuprulez/similar_galaxy_workflow), with various branches active last year, a separate master branch subsequently active in Sep and Oct, and a 'tool_recommendation_release_19_09' branch active aftewards between Oct 2019 and Feb 2020. It would be nice to note the history and the relations between these repos and branches somewhere in the beginning of all the involved README.md files, especially so that the community sees where to look for the active work and where not (and eventually fork, experiment, contribute, start issue threads, etc.).

Author response:

We have added a note in the README.md file explaining the old and new repositories. Initial work to create a tool recommendation model is stored at https://github.com/anuprulez/similar_galaxy_workflow. This repository storing the history of work until October 2019 will not be used in future. The current repository (https://github.com/anuprulez/galaxy_tool_recommendation) will be used for current and future developments. Further, the information about different branches has been added to the readme file.

It would be a nice bonus to have the version used in the article tagged as a release on GitHub, ideally with a Zenodo archive DOI (https://zenodo.org/oauth/login/github/?next=%2Fdeposit followed by https://zenodo.org/account/settings/github/), which could be permanently linked from the published article.

Author response:

We have added the zenodo link (https://zenodo.org/record/3885595#.Xt5uuzczY5k) to the code repository (https://github.com/anuprulez/galaxy_tool_recommendation).

Notably, the README.md currently contains useful information on how to reproduce the work, and use it on another Galaxy server. I would also welcome in the near future some additional brief information about this project, and explicitly separate "how to-s" for various types of usage/users: Galaxy end users, Galaxy admins, researchers who want to reproduce the work, existing contributors to the project, and potential new contributors. CONTRIBUTING.md might be a good option for the latter or latter 2; and/or README.md can include the overview of all the information, with further links to potential other files with details.

Powered by Editorial Manager® and ProduXion Manager® from Aries Systems Corporation

Author response:

We have added a CONTRIBUTING.md (https://github.com/anuprulez/galaxy_tool_recommendation) file explaining how to contribute to this repository. We have also updated the README.md file which contains information about how to reproduce the work, how to enable this feature in Galaxy for admins and how to use/see recommendations for Galaxy end users.

Abstract:

The abstract of the manuscript is nicely concise and gives a good summary of the work. I suggest it could possibly be made even easier to read and understand (especially for unacquainted readers), with a couple of small changes.

"To make creating workflows easier, faster and less error-prone, a predictive system is developed to recommend tools facilitating further analysis." could be simplified into something in the sense of "To help (users) with creating workflows, we developed a system to recommend tools that (would) facilitate further analysis."

Author response:

We have changed the sentence to:
“To help researchers with creating workflows, a system is developed to recommend tools that can facilitate further data analysis.”

"A model is developed to recommend tools by analysing workflows, composed by researchers" - without comma, or comma after 'tools' would read smoother.

Author response:

We have changed the sentence to:
“A model is developed to recommend tools using a deep learning approach by analysing workflows composed by researchers on the European Galaxy server.”

"precision@1, precision@2 and precision@3 metrics" - would it be clearer with e.g. "top-one, top-two, and top-three metrics", or not?

Author response:

We have updated the names of metrics to top-1 and top-2. Now, the sentence reads as:
“Mean accuracy of 98% in recommending tools is achieved for the top-1 metric.”

The Conclusion(s) part is hard to read. The preprint included a sentence similar to "The model is accessed by the Galaxy API to recommend tools in real time.", which could perhaps fit in. Subsequent "Multiple user interface (UI) integrations on the European Galaxy server communicate with an API, which accesses the model, to apprise researchers of recommended tools in an interactive manner." could be simplified - especially if the previous sentence from the preprint is included in some form - into e.g.: "Multiple user-interface integrations on the European Galaxy server communicate with the(?) API, which accesses the model, to provide researchers with recommended (next?) tools, in an interactive manner." The last sentence would be clearer with something like "The scripts to create the recommendation model, and the (sample) (training) data (we used), are available under MIT License at ..."

Author response:

We have updated the text as follows.

“The model is accessed by a Galaxy API to provide researchers with recommended tools in an interactive manner using multiple user interface (UI) integrations on the European Galaxy server. Good quality and highly-used tools are shown at the top of the recommendations. The scripts and data to create the recommendation system are available under MIT license at https://github.com/anuprulez/galaxy_tool_recommendation”

Main text:

The manuscript is excellently written, however, including some opportunities for minor improvements to smoothen some of the sentences. These include unusual or ambiguous words ('stemming', 'appropriately', the latter can be omitted); some punctuation (I find some missing commas, but appreciate nicely and correctly used hyphenation); and minor grammar nuances, e.g. "Galaxy is a(n) open-source data-processing platform...", or "time spent (in)".

Author response:

Thank you for the appreciation.

I also included a couple of positive comments among the following notes.

[Introduction]

"... to ensure it, a system is needed which can recommend correct tools while creating a workflow." - both 'ensure' and 'correct' are too strong (restrictive) words in this context, claiming way too ambitious goals.

Author response:

We have updated the text as follows:
“To make it possible, a system is needed which can recommend useful tools at each step while creating a workflow.”

"has a defined number of data types for these input and output files" - clearer would be something like "supports a number of formats of these input and output files", in case it is only the format that is the point here. (The use of 'data types' in what follows after is fine, unless the authors would want to be more precise (but verbose) with types of data and data formats. However, I don't suggest that it is necessary here. Still, one may want to take into account that outside of Galaxy, 'data type' relates to types in (typed) programming languages for programmers, and to acquisition technologies and techniques in the jargon of many biologists and medical researchers.)

Author response:

We have updated the text as follows:
“A tool consumes one or more data files as input and produces one or more data files as output and supports a number of formats of these input and output files.”

"Second, it will help them bypass the step of searching for tools separately which shows potential to further reduce the time spent in creating workflows and increase the accessibility of tools." - For me personally, this is where I see the biggest benefit of the recommender system (bigger than the "First", 'correctness' use case). Maybe some

smoothening of the sentence, e.g. "Second, it will help users bypass the step of searching for tools separately, what will further reduce the time spent in creating workflows and (at the same time) increase the accessibility of tools."

Author response:

We have updated the text as follows:
“Second, it will help researchers bypass the step of searching for tools separately, which will further reduce the time spent in creating workflows and at the same time increase the accessibility of tools.”

"Finally, it can also be used to promote the newly added tools in Galaxy by showing them alongside the recommended tools predicted using the deep learning approach." - This is really important and great!

Author response:

Thank you for your appreciation.

"metadata of tools" might be clearer with "information about tools". Also in Discussion.

Author response:

We have updated the text as follows:
“First, it does not require collecting and storing information about tools.”

"higher-order relationships among tools" are explained in Figure 1. The explanation is sufficient, and aided by the figure, but perhaps it would then be nice to add a link to the figure where they are mentioned the first time (except for the abstract).

Author response:

We have updated the text as follows:
“Second, it takes into account the higher-order relationships among tools (Figure 1) in tool sequences.”

"A Bayesian network can also be used..." perhaps a new paragraph?

Author response:

We have addressed it in the paper.

[Results]

"the next generation sequencing (NGS) data" could just be "the sequencing data".

Author response:

We have addressed it in the paper.

The recommended tools mentioned in the text following right after do not match with the recommended tools in Figure 5. It would be nice to explain what those in Figure 5 are, or match them with the text. Perhaps an easy UX fix: Display which step the recommended tools are for.

Author response:

We have updated the caption of the image to describe its contents. Moreover, we have

updated the text in the “Examples of tool recommendations” section describing this image.

Figure 6 description: RNA-STAR is usually spelled with all capitals, as in the main text.

Author response:

We have addressed it in the paper.

[Discussion] (Reading the content, it could also be titled "Summary and final remarks".)

Author response:

We have addressed it in the paper and updated the name of the section to “Summary and future work”.

"A recommender system to predict tools in Galaxy..." - Isn't it a "prediction system to recommend tools in Galaxy" instead? What about simply "A system to recommend tools in Galaxy..."?

Author response:

We have addressed it in the paper.

"in real time."

Author response:

We have addressed it in the paper.

The last 2 sentences could be part of a separate paragraph on "future work"/"future options", perhaps slightly more elaborate. For example, any UI ideas on how to suggest both highly-used and new tools transparently, and how to administer these (automatically?)? Or would it be worth experimenting with the inclusion of tools similarity, especially if these are in the future better annotated with semantic information?

Author response:

We have addressed it in the “Summary and future work” section. Please find the relevant text from the paper below.

“Different Galaxy servers maintain different sets of tools and workflows. The current approach can be used to create different recommendation models for different Galaxy servers. Alternatively, all the workflows can be collected from multiple Galaxy servers and using the current approach, one recommendation model can be created by learning on the complete set of workflows and the model can be distributed to different Galaxy servers. To improve the quality of recommendations, the annotations of tools can be incorporated in the learning mechanism by assigning higher weights to the annotated tools in comparison to tools which are not annotated. Tools containing similar annotations may have similar functionalities and using these similarities, tool recommendations can be further enhanced by showing similar tools for each recommended tool.”

The inclusion of new tools is discussed in the “New tools as recommendations” subsection of “Methods” section.

Powered by Editorial Manager® and ProduXion Manager® from Aries Systems Corporation

	[Methods]<br><br>"usage based relevance" could be "usage-based relevance" or just "usage statistics" or even just "usage frequency" itself.<br><br>Author response:<br><br>We have addressed it in the paper.<br><br><br>GRU layer: I like the concise explanation! Similarly for the activation functions, and the prediction of usage frequency.<br><br>Author response:<br><br>Thank you for the appreciation.<br><br><br>"time complexity", no hyphen.<br><br>Author response:<br><br>We have addressed it in the paper.<br><br><br>"parameters (of) these architectures"<br><br>Author response:<br><br>We have addressed it in the paper.<br><br>Space permitting, it would be a nice extra to have figures of the used CNN and "DNN" architectures included as well, like the Figure 8 for the GRU RNN, near the "Multiple neural network architectures" subsection.<br><br>Author response:<br><br>We have added different neural network architecture images to the section S3 of the supplementary document.<br><br>This was all from my side, I hope to see this article published in GigaScience!<br><br>Sincerely yours,<br>Matúš Kalaš
**Additional Information:**	
**Question**	**Response**
Are you submitting this manuscript to a special series or article collection?	No
**Experimental design and statistics**<br><br><br>Full details of the experimental design and statistical methods used should be given in the Methods section, as detailed in our Minimum Standards Reporting Checklist. Information essential to interpreting the data presented should be made available in the figure legends.	Yes

Have you included all the information requested in your manuscript?	
**Resources**<br><br>A description of all resources used, including antibodies, cell lines, animals and software tools, with enough information to allow them to be uniquely identified, should be included in the Methods section. Authors are strongly encouraged to cite Research Resource Identifiers (RRIDs) for antibodies, model organisms and tools, where possible.<br><br>Have you included the information requested as detailed in our Minimum Standards Reporting Checklist?	Yes
**Availability of data and materials**<br><br>All datasets and code on which the conclusions of the paper rely must be either included in your submission or deposited in publicly available repositories (where available and ethically appropriate), referencing such data using a unique identifier in the references and in the "Availability of Data and Materials" section of your manuscript.<br><br>Have you have met the above requirement as detailed in our Minimum Standards Reporting Checklist?	Yes

Powered by Editorial Manager® and ProduXion Manager® from Aries Systems Corporation

LaTeX - Main Document (TeX file)
Click here to access/download;LaTeX - Main Document (TeX file);main.tex

GigaScience, 0000, 1–13

doi: xx.xxxx/xxxx
Manuscript in Preparation
Technical Note

TECHNICAL NOTE

Tool recommender system in Galaxy using deep learning

Anup Kumar[1,*,†], Helena Rasche[1, ‡, †], Björn Grüning[1, §, †] and Rolf Backofen[1,2, ¶, †]

[1]Bioinformatics Group, Department of Computer Science, University of Freiburg, Georges-Koehler-Allee 106, 79110 Freiburg, Germany and [2]Signalling Research Centres BIOSS and CIBSS, University of Freiburg, Schaenzlestr. 18, 79104 Freiburg, Germany

[*]kumara@informatik.uni-freiburg.de
[‡]helena.rasche@gmail.com
[§]gruening@informatik.uni-freiburg.de
[¶]backofen@informatik.uni-freiburg.de
[†]Contributions follow the order of the names of authors

Abstract

Background Galaxy is a web-based and open-source scientific data-processing platform. Researchers compose pipelines in Galaxy to analyse scientific data. These pipelines, also known as workflows, can be complex and difficult to create from thousands of tools, especially for researchers new to Galaxy. To help researchers with creating workflows, a system is developed to recommend tools that can facilitate further data analysis. **Findings** A model is developed to recommend tools using a deep learning approach by analysing workflows composed by researchers on the European Galaxy server. The higher-order dependencies in workflows, represented as directed acyclic graphs, are learned by training a gated recurrent units (GRU) neural network, a variant of a recurrent neural network (RNN). In the neural network training, the weights of tools used are derived from their usage frequencies over time and the sequences of tools are uniformly sampled from training data. Hyperparameters of the neural network are optimised using Bayesian optimisation. Mean accuracy of 98% in recommending tools is achieved for the top-1 metric. **Conclusions** The model is accessed by a Galaxy API to provide researchers with recommended tools in an interactive manner using multiple user interface (UI) integrations on the European Galaxy server. Good quality and highly-used tools are shown at the top of the recommendations. The scripts and data to create the recommendation system are available under MIT license at https://github.com/anuprulez/galaxy_tool_recommendation.

Key words: Recommender system; Galaxy; Workflows; Deep learning; Neural networks; Gated recurrent units

Findings

Background

Life sciences depend increasingly on high-throughput data, turning them into data science to a large extent. However, raw high-throughput data does not have much value on its own without proper analysis and interpretation of the data. To simplify the data analysis process and to ensure a reproducible analysis, several workflow systems such as Bcbio-nextgen, Omics Pipe, Nextflow, Luigi, Toil and many others have emerged [1, 2, 3]. The main idea for workflow systems is based on the observation that any computational analysis of high-throughput data encompasses multiple steps such as quality control, preprocessing, quantification and statistical analysis to transform raw data into scientific results. Collec-

Compiled on: August 2, 2020.
Draft manuscript prepared by the author.

tively, these steps form a workflow where each step performs a definite transformation of the data, which can be performed using standardised tools. Using workflow for the analysis is simple and convenient and has several advantages. First, it is easy to replace individual tools by a newer version or to assess the influence of the associated step on the final result. Second, a workflow can be saved, shared and reused, which ensures reproducible research. Therefore, workflows are becoming essential in the analysis of scientific data and there are multiple platforms where researchers can create workflows for their analyses. However, a critical question is how to assess whether a generated workflow is state-of-art or even valid at all. To give a concrete example, one can use several real-valued input vectors (such as fluorescence-based measurement stemming from arrays), transform them into integer-based values in the first step and combine it with a tool that uses a count-based statistics (such as negative binomial distribution as used in DESeq2 [4]) to determine values that show high differential behaviour. While this workflow would run on a workflow system without problems and even produce some results, the generated results are not valid because of the wrong statistical model. Therefore, it is important to use a tool for each step in a workflow which can bring desired results. To make it possible, a system is needed which can recommend useful tools at each step while creating a workflow.

Galaxy and workflows

Galaxy is an open-source data processing platform which enables researchers to create and store their workflows for multiple scientific analyses [5]. A workflow in Galaxy is a directed acyclic graph and consists of one or many tool sequences to analyse scientific data such as DNA and RNA sequences. A tool consumes one or more data files as input and produces one or more data files as output and supports a number of formats of these input and output files. In workflows, the tools are connected one after another following a constraint that the adjacent tools must have compatible data types. In other words, the data types of output files of a tool should match the data types of input files of the following tool. Galaxy has thousands of accessible tools and acquiring familiarity and constructing workflows with these tools can be a complex and time-consuming task, especially for researchers new to Galaxy. To assist them in creating workflows and making them aware of the possible tools for further analyses, a recommender system is devised. The benefits of having such a system are manifold. First, it will avoid the loss of time spent in creating erroneous or less optimal workflows by choosing tools which may produce undesired results and thereby making researchers more efficient. Second, it will help researchers bypass the step of searching for tools separately, which will further reduce the time spent in creating workflows and at the same time increase the accessibility of tools. Third, it will promote good quality tools having higher usage frequencies in the past (last one year) to the top of the recommendations and downgrade those having lower usage frequencies to the bottom of the recommendations. It is achieved by assigning weights to tools which are derived from their usage frequencies over a period of time. Finally, it can be extended to promote the newly added tools in Galaxy by showing them alongside the recommended tools predicted using the neural network approach.

Recommender systems

The objective of having recommender systems in fields such as scientific literature search, online shopping, travel bookings, media-service providers and many other fields is to help people discover suitable, interesting and newly-released products. These recommended products are recognised based on the usage and purchasing patterns of people in the past. In the field of scientific literature search, the exponential increase in the number of published papers necessitates having a recommender system to help scientists explore relevant and recent papers quickly [6, 7, 8]. Recommender systems are significant in the world of commercial applications too. Companies such as Amazon and Netflix have appropriately used them to learn preferences of their respective customers in selecting products such as their favourite books or movies and to propose a few products out of a large catalogue. By enabling users and customers to discover reasonable and customised products, recommender systems have helped them grow as organisations [9, 10]. In short, recommender systems make it faster for users and customers to look through a few recommended products to find the most suitable ones. These successful implementations of recommender systems by organisations across the world working in diverse areas to assess the needs of their respective users in proposing relevant products motivated us to create a tool recommender system in Galaxy.

Related work

To simplify creating workflows for scientific analyses, a few approaches have been proposed which suggest alternative tools and workflows. EDAM (EMBRACE Data And Methods) and semantic annotations of tools are used to compose workflows automatically for mass-spectrometry based proteomics [11]. The annotations include the names, functionalities, input and output data types of tools. The PROPHETS (Process Realisation and Optimisation Platform using Human-readable Expression of Temporal-logic Synthesis) program generates suitable candidates of workflows which match the goal of the proposed workflow and its annotations [12]. WINGS (Workflow INstance Generation and Specialization) offers multiple variations of a workflow created using different tools. It makes use of the input parameters, types of datasets and functions of tools to build the variations [13, 14]. The approach used in [15] utilises data types to facilitate the automatic creation of workflows. All these approaches depend either on annotations or matching input and output data types of adjacent tools in workflows and they pose challenges such as the addition and maintenance of the meaningful annotations of tools and extracting input and output data types of adjacent tools. Moreover, these approaches have their workflow generation restricted to a few specific bioinformatics analyses such as proteomics or proteogenomics. In addition, they do not discuss the presence of higher-order relationships [16] in tool sequences of workflows. Our approach to recommend tools in workflows aims to overcome these challenges in the following manner. First, it does not require collecting and storing information about tools. Second, it takes into account the higher-order relationships among tools (Figure 1) in tool sequences. Finally, it incorporates workflows from multiple scientific analyses to produce the recommender system.

Sequential learning on workflows

Workflows, created by many researchers in Galaxy for different scientific analyses, are decomposed into numerous tool sequences (Figure 1). The sequential nature of these tool sequences where tools are connected one after another inspires us to apply similar learning techniques used for other sequential data such as text and speech. There are multiple studies in the fields of natural language processing, clinical research and speech recognition which apply deep learning techniques on se-

quential data to obtain good accuracy in predicting future items. The approach used in [17] finds context in long sequences of words for sentiment analysis and part-of-speech tagging using recurrent neural network (RNN) and achieves 85% and 93% accuracy, respectively. For clinical data, learning on long sequences of health states proves to be beneficial [18]. The health states of patients recorded at different time points are analysed by accessing their electronic health records. The future health states of patients could be predicted by training RNN on the sequences of their past health states to achieve 85% accuracy. Moreover, the variants of RNN are used to model speech and music signals [19, 20]. These successful studies benefit from the sequential learning techniques using different variants of RNN. Therefore, in our work, a variant of RNN - gated recurrent units (GRU) - is used to create the tool recommender system in Galaxy.

A Bayesian network can also be used for modelling directed acyclic graphs (workflows) [21, 22]. It requires computing joint and conditional probabilities of nodes in graphs and an increase in the number of nodes can lead to a higher cost to compute these probabilities. In addition, making predictions by learning a probabilistic network is a hard problem [23, 24, 25]. Because of these drawbacks of using a Bayesian network, it is not used in our approach to create the recommender system in Galaxy.

Data description

Over 18,000 workflows from different scientific analyses such as RNA-seq, Variant-calling, Hi-C, Assembly, Single-cell, Proteomics and so on in the European Galaxy server [26] have been used to create the recommender system. A workflow consisting of 5 tools is shown in Figure 1a. It is divided into smaller tool sequences as shown in Figure 1b, 1c and 1d. The last tool, shown in green, of each tool sequence (of length n) is assigned as the label of the sub-sequence (of length n-1) shown in blue in Figure 1. A label is an output which is learned and predicted by the recommender system. In the neural network learning, a tool is a label. For example, in Figure 1b, Tools D and E are the labels of the sub-sequence Tool A → Tool B → Tool C. They show higher-order dependencies in their connections which implies that a tool is not only dependent on its immediate predecessor but also on all prior tools in the tool sequence. For example, in Figure 1c, the Tool C is dependent on Tools B and A. By analysing multiple workflow fragments in this way, the neural network should learn that the label of a tool sequence Tool A → Tool B is Tool C. It is expected that dividing a tool sequence into fragments with a minimum length of two tools, as shown in Figure 1c and 1d, will improve the generalisation performance of the neural network because it gets more tool sequences with a variety of lengths to learn from. The dependencies shown in Figure 1b, 1c and 1d present in tool sequences are learned using the GRU neural network by modelling the conditional probability given by Equation 1 [27]. Using the approach explained above, more than 229,000 tool sequences are extracted.

Tools in Galaxy have different usage patterns. Some tools are used more often than other tools for multiple reasons such as differences in their functions and availability of similar but better tools. It is essential to analyse the usage patterns of tools because the recommender system proposes tools for researchers and these tools should have high relevance to their analyses. One of the key indicators of relevance of tools can be their high usage frequencies. If a tool has been used often in the recent past, it implies that the tool is relevant. However, if a tool was used often a few years ago but has been used less often in the last six months then the relevance of that tool has certainly declined. The usage frequencies of tools (shown as labels in Figure 1) over the past year are shown in Figure 2.

The set of workflows may have poor quality workflows which are non-published or deleted or may have errors in their tool connections. To distinguish between good and poor quality tool connections, the labels for each tool sequence are divided into two parts - standard and normal labels. The standard labels come from the published, non-deleted and non-erroneous workflows while the normal labels come from other workflows. While recommending tools for a tool or tool sequence, the standard labels are promoted to the top of the recommendations if available followed by the normal labels. This should allow good quality tools to be shown as the top recommendations.

Tool sequences from these workflows may vary in number - some may occur more frequently and others may not. Therefore, the complete set of tool sequences may not be equally representative of all workflows coming from different scientific analyses. Learning on the imbalanced set of tool sequences can induce bias which may have an undesired outcome - good accuracy in recommending tools coming from highly frequent tool sequences and poor accuracy for tools coming from less frequent ones. To mitigate this bias, all tool sequences are chosen with uniform frequency while training the neural network which should allow it to have a comparable accuracy in recommending tools in different scientific analyses. This uniform sampling strategy is discussed in the "Implementation" section in detail.

Results

Three different neural network architectures - dense neural network (DNN), convolutional neural network (CNN) and gated recurrent units neural network (GRU) - are compared on their performances in predicting tools (Figures 3 and 4). The models obtained after training all the neural network architectures are used to predict tools for the tool sequences in the test data after every training iteration. Top-k precision (precision@k) is a popular metric for evaluating a recommender system [28, 29, 30]. Precision@k implies how many in the k predicted tools are correct. The correctness here refers to the compatibility of the predicted tools with the tool for which predictions have been made. For example, $k = 2$ implies that the number of predicted tools are 2 with the highest predicted scores. If only 1 of them is correct, then the precision@2 is $\frac{1}{2} = 0.5$. In this way, precision@1 and precision@2 are computed for all the tool sequences in the test data and then averaged. Precision@1 and precision@2 metrics are used in this approach to evaluate the quality of the tool recommender system. Precision of recommended tools are computed separately for the standard and normal recommendations and shown in different plots (Figures 3 and 4) but for usage frequencies, they are combined into one plot (Figure 5). The precision and usage frequencies of the predicted tools for the precision@1 and precision@2 (top-1 and top-2) metrics are computed over 10 training iterations for each experiment run. They are averaged and their respective standard deviations are computed over 10 experiment runs. Mean precision and usage frequencies are shown by the respective line plots and the shaded regions span the area between one standard deviation above and below the mean (Figures 3, 4 and 5).

Comparison of GRU neural network with other approaches

The GRU neural network with the weighted cross-entropy loss function shows superior performance to CNN (Figures 3d, 3f, 4d and 4f) by achieving 98% top-1 standard and normal precision which proves that the GRU layers in a neural network are better for learning on tool sequences than the convolutional layer. Moreover, it shows a lower divergence in standard and

normal precision and usage frequencies (Figures 3d, 3f, 4d, 4f, 5d and 5f) establishing that its predictive strength is more stable than CNN over multiple experiment runs. Surprisingly, the weighted cross-entropy loss function does not have any beneficial effect on the CNN architecture as its standard and normal precision and usage frequencies show higher divergence over multiple experiment runs (Figures 3c, 3d, 4c, 4d, 5c and 5d). Therefore, CNN is not used in our approach. In contrast to CNN, DNN achieves a similar standard and normal precision to the GRU neural network with a small divergence (Figures 3a, 3b, 3f, 4a, 4b and 4f). However, due to higher divergence in accumulated usage frequencies, it is not used in our approach (Figures 5a, 5b and 5f). Weighted cross-entropy loss function in the GRU neural network (Figure 5f) drives it to classify tools more robustly with higher usage frequencies. In other words, it predicts tools with higher precision than CNN and lower divergence in usage frequencies than DNN. Therefore, it is used in our approach to learn on tool sequences and recommend tools.

To compare the performance of the GRU neural network with approaches which do not use any neural network, two ideas are explored. First, using a simple approach to store all the indices of sequences of tools [31] and second, using Extra-Trees classifier [32]. These approaches are discussed in section S1 of the supplementary document in detail.

Benefit of regularisation

Using regularisation minimises overfitting by assisting the GRU neural network to make better recommendations by predicting tools having low usage frequencies but useful in addition to tools with high usage frequencies. For example, the recommendations of "UMI-tools count" [33] tool with the regularised model include "Seurat" [34] tool which is absent from recommendations by the non-regularised model. Another example is for "RaceID, Lineage computation using StemID" tool sequence which gets "Lineage Branch Analysis using StemID" [35] tool as one of the recommendations by the regularised model while there is no recommendation at all by the non-regularised model. The recommendations for a popular mapper, RNA-STAR [36], are "featureCounts" [37], "MultiQC" [38], "Infer Experiment" [39] and few others by both the models. But, in addition to these recommendations, the regularised model recommends the "Read Distribution" [39] tool which is not predicted by the non-regularised model. More details have been provided in section S4 of the supplementary document.

Examples of tool recommendations

To illustrate the real time usage of the recommender system in the European Galaxy server, two examples have been provided. The first one shows recommended tools for a tool sequence with 3 tools, Trimmomatic [40] → BWA-MEM [41] → FreeBayes [42], in the workflow editor of the European Galaxy server (Figure 6). Trimmomatic is used to trim the sequencing data such as DNA and RNA sequences. One of the useful analyses after trimming the sequences is to map them on a reference genome using a mapper. Several mappers such as BWA-MEM [41], Bowtie2 [43] and RNA-STAR [36] are predicted. BWA-MEM is chosen from the predicted mappers and connected to Trimmomatic. After mapping, for further analysis of mapped sequences, many tools are predicted such as MultiQC for summarising the quality of mapping, featureCounts for counting the reads mapped to different regions on the genome or FreeBayes for detecting variants and a few others. FreeBayes is chosen and a list of recommendations is shown as a dropdown containing tools such as "bcftools norm" [44], VcfAllelicPrimitives [45] and many others for Trimmomatic → BWA-MEM → FreeBayes tool sequence. Another example of tool recommendations after using RNA-STAR is shown in Figure 7. It shows follow-up tools such as bamCoverage [46] for calculating read coverage, MultiQC, featureCounts and a few others. In summary, the tool recommendations provide useful knowledge about tools to Galaxy users and researchers to continue multiple scientific analyses.

Table 1 lists a few standard and normal recommended tools for multiple tool sequences in different scientific analyses such as Computational chemistry, Epigenetics, Machine learning, Proteomics, RNA sequencing and a few others. The recommended tools shown in this table are highly used for standard scientific analyses as highlighted in multiple GTN training materials [60].

Implementation

To create a tool recommender system in Galaxy, workflows are collected from the European Galaxy server. A workflow may have one or many tool sequences where tools are connected one after another. Tool sequences are transformed into matrices and produced as input to a GRU neural network to learn patterns in the connections of tools.

$$p(x_T | x_1, x_2, ..., x_{T-1}) \tag{1}$$

The probability of a tool (x_T) is estimated given all other prior tools (x_1, ..., x_{T-1}) for a tool sequence (x_1, ..., x_{T-1}, x_T). Neural network learning is classification because there are labels for tool sequences which are learned and then predicted. Moreover, the classification is multi-class (multiple tools as labels) and multi-label (multiple tools as labels for a tool sequence) [61]. To ensure an unbiased learning and evaluation by the neural network, the set of tool sequences is divided into two parts - training and test. The training data is used for learning a model and the test data is used for evaluating the model.

Relevance of tools

To incorporate the usage frequency of tools in the recommender system, the usage frequencies of all the tools used in the last one year have been collected and are used in the neural network training as the weights (logarithm of usage frequencies) of tools. A tool which has been used often (for example Tool B in Figure 2) in the past one year is assigned a higher weight than a tool (for example Tool C in Figure 2) which has been used less often in the past one year. When tools are recommended a score is assigned to each tool by the neural network. It is expected that a tool with a higher weight gets a higher score and a tool with a lower weight gets a lower score. To summarise, the relevance of a tool to be used in a workflow decays if its usage drops over time in Galaxy. Alternatively, the relevance of tools can also be ascertained by counting the occurrence of each tool in all workflows and these occurrences can be used as their weights in the neural network training. But, it may happen that some tools which were used often in the past to create workflows are not used anymore. Therefore, assigning weights to these tools in the neural network training based on their occurrences in workflows may not be a good indicator of their relevance and overall, may not be optimal.

Uniform sampling

Workflows in Galaxy come from different scientific analyses. It may happen that the number of workflows from these analyses are not comparable - some analyses may have a large number of workflows while some may have only a small number of workflows. This can cause some tools to be present very frequently in workflows while other tools are less frequent. Learning on these workflows and recommending tools may exhibit

Table 1. Standard and normal recommended tools for tool sequences in different scientific analyses

Scientific analyses	Tool/Tool sequences	Recommended tools (standard)	Recommended tools (normal)
Computational chemistry	Molecule to fingerprint [47]	Taylor-Butina clustering, NxN Clustering [48], Similarity Search	–
Epigenetics	hicBuildMatrix [49]	hicSumMatrices	hicMergeMatrixBins, hicPlotMatrix [50], hicPCA, hicTransform
Epigenetics	multiBamSummary [46]	plotCorrelation [51]	plotPCA
Epigenetics	Bowtie2	MASC2 CallPeak [51]	bamCoverage, FreeBayes
Machine learning	Create a deep learning model architecture	–	Create deep learning model [52], Build Deep learning Batch Training Models
Proteomics	Msconvert [53]	Search GUI, FlashLFQ	PeakPickerHiRes [54]
RNA Sequencing	Cutadapt [55]	FastQC, RNA-STAR, MultiQC [56]	Bowtie2, Hisat2, BWA-MEM
RNA Sequencing	Cutadapt [55], RNA-STAR	featureCounts, MultiQC, Infer Experiment [56]	bamCoverage, RmDup
Single-cell	UMI-tools extract [33]	RNA-STAR [57]	Bowtie2, Hisat2, BWA-MEM, UMI-tools group
Single-cell	Initial processing using RaceID [35]	Clustering using RaceID [58]	–
Single-cell	Initial processing using RaceID, Clustering using RaceID [35]	Cluster Inspection using RaceID [58], Lineage computation using StemID	–
Variant-calling	FreeBayes	VcfAllelicPrimitives [59]	Gemini load
Variant-calling	FreeBayes,VcfAllelicPrimitives	SnpSift Filter, VT normalize	SnpEff eff [59]

bias by showing better recommendations for the frequently occurring tools and poorer recommendations for the less frequent ones. To showcase this imbalance, the frequencies of the last tool in each tool sequence in training data are calculated and it is found that only a few tools have large frequencies and most of the tools are present in low frequencies (Supplementary Figure 3). For example, the tools with very high frequencies (> 10,000) are "Concatenate datasets", "Cut", "Grouping" and "Join" while the tools having very low frequencies (< 5) are "Cluster inspection using RaceID", "rDock cavity definition" [62] and "ChiRA collapse". Therefore, to overcome this drawback, the training data created after extracting tool sequences should be balanced in order to make the neural network learn on a similar number of tool sequences from different scientific analyses in each training iteration. To implement this strategy, a set of last tools in all tool sequences from the training data is collected. Further, for each tool in this set, a list of indices of tool sequences in the training data are stored for which it is the last tool (Supplementary Table 3). Only the last tools are considered for implementing this strategy because of two reasons. First, the smallest tool sequences contain only two tools and second, all tools become the last tool in at least one tool sequence and the computed frequencies of these last tools suggest their overall frequencies in the training data.

In the neural network training, for each iteration (which consumes all tool sequences in the training data), small batches containing equal number of tool sequences are created. For example if batch size is 100 and the size of training data is 2000, then 20 (2000/100=20) batches are created, each containing 100 tool sequences. In each batch, 100 tools from the set of last tools are uniformly selected (Column 2 in Supplementary Table 3) and for each selected tool, a tool sequence is chosen uniformly from its respective list of tool indices (Column 3 in Supplementary Table 3). After selecting tool sequences for many batches for each iteration of training (epoch), it is expected that all the tools from the set of last tools and their respective tool sequences are chosen. Performing this uniform selection of different tool sequences for each iteration, the training data becomes balanced. Supplementary Figure 4 shows that each last tool is present approximately 1670 times on an average in each iteration (epoch). The order of tools in the Supplementary Figures 3 and 4 are same.

Data transformation

Tool sequences extracted from workflows are transformed into vectors because neural networks require input data to be represented as vectors and matrices. Each tool sequence has one or more labels (Figure 1) and they are transformed into different vectors - a tool sequence vector (Figure 8b) and a label vector (Figure 8d). To form these vectors, a dictionary of tools is needed which stores an index for each tool. Using the indices of tools, a tool sequence vector is created preserving the

original order of tools as in the tool sequence. For example, Tool A has an index of "12" in the dictionary, therefore it is replaced by "12" in the vector (Figure 8b). The vector is padded with trailing zeros to keep the length of the vector same across the varying lengths of tool sequences. The size of this vector is 25 which means that a tool sequence can have a maximum of 25 tools. The tool sequences larger than this size are discarded. The labels (Figure 8c) are transformed into a bit vector (Figure 8d) in which the positions, stored as indices in the dictionary of tools, of the labels (tools) are turned "on" (set to 1) specifying that these tools are the labels of the tool sequence and others are not (set to 0). It has the same size as the dictionary of tools. In the machine learning field, it is also known as a multi hot-encoded vector. Together, these two vectors form a training sample for the neural network. A pair of vectors are created in this manner for each tool sequence and for all the tool sequences, they are combined to form two matrices - one for tool sequences and another for their respective labels. Internally, the label vectors have two subsets, one for standard labels (from the published, non-deleted and non-erroneous workflows) and another for normal labels (from the rest of the workflows). These matrices form input data to the neural network.

Neural network architecture

GRU neural network, a variant of RNN, is used for creating a model to recommend tools. The neural network architecture has multiple components such as different layers (Figure 9), activation functions, class weights, loss function, hyperparameter tuning technique which are discussed in detail in the following paragraphs.

Embedding layer. The first component of the neural network architecture is an input layer (Figure 9) which learns an embedding, a fixed-size vector, for each tool. This vector is used by the neural network as an internal representation of a tool. The embedding vector replaces the indices of tools in each tool sequence. The size of the embedding vector is fixed for all tools. For example, the vector of a tool sequence [12, 6, 75, 0, 0, ..., 0] is transformed into [[0.3, 0.01, 0.003, ..., 0.23], [0.5, 0.1, 0.005, ..., 0.9], [...], 0, 0, ...,0] by the embedding layer. The same embedding vector represents a tool in all tool sequences in which the tool is present.

GRU layer. The stacked layers of GRU learn deeper structures in the tool sequences by modelling the conditional probabilities of tools (labels) given all other prior tools (Figure 9). GRU has certain advantages which helps it to learn on sequential data. First, it avoids the problems of vanishing and exploding gradients which commonly occur in traditional RNN [63]. It is important because learning higher-order dependencies depends on the gradients of errors concerning the parameters (recurrent and input weight matrices) of GRU layers. Second, GRU has slightly fewer parameters than the long short-term memory network (LSTM), another variant of RNN, which makes using GRU simpler than LSTM. Finally, it achieves similar accuracy as the LSTM [19].

Output layer. The last component of the neural network architecture is a dense layer which computes predictions (Figure 9). The dimension of this layer is equal to the number of unique tools because it predicts a score for each tool (label). The predicted score of each tool is considered as its probability of being the label of an input tool sequence. The closer the predicted score of a tool is to 1, the more probable it is to be the recommended tool and the closer it is to 0, the less probable it is to be the recommended tool.

Dropout layer. Overfitting happens when a neural network performs exceptionally well on the training data but its performance on test (unseen) data remains poor. To minimise the effect of overfitting, a dropout layer is used between two layers of the neural network. It sets a few randomly chosen connections to 0 in the neural network to introduce some randomness to minimise overfitting [64, 65]. 3 dropout layers are used in our approach - one between the embedding and the first GRU layers, one between 2 GRU layers and the last one between the second GRU and dense layers.

Activations. These are mathematical functions which are used in neural networks to transform inputs to a layer into its outputs. Two activations are used in our approach - one is exponential linear units (ELU) [66] and another is sigmoid (Equation 2). ELU is used for both the GRU layers and has a special feature of being negative when the input is negative which allows mean activation (output) to get closer to 0 compared to other activation functions such as ReLU [67] which is always positive. As mean activations get closer to 0, the approximated and actual gradients get closer to each other. Therefore, using ELU in our neural network as an activation can be useful to achieve faster training, an increased drop in loss and better accuracy. Sigmoid is used in the output layer which normalises any real number to lie between 0 and 1 and it is considered as a probability of each tool.

$$f(x) = \frac{1}{1 + e^{-x}} \tag{2}$$

Usage frequencies of tools as weights. To ensure that the relevance of tools decays with time if they have not been used regularly in the recent past, their usage frequencies are used as their respective weights in the neural network training. The usage frequencies of tools over the past year (Figure 2) have been collected from the European Galaxy server. A curve is fit through the usage frequencies of each tool using support vector regression (SVR) to display a trend of the usage of tool over time. Using this trend, the usage of the tool for the next month is predicted and its logarithm is used as the weight for this tool. The logarithm of usage frequencies is computed to normalise them because only a few tools have significantly large magnitude of usage compared to that of the remaining tools which may lead the neural network to learn and predict only tools with very large magnitude of usage and ignore other tools. Learning a trend for each tool involves 5-fold cross-validation and optimising two hyperparameters of SVR, kernel and degree, using grid search. The values used for the kernel are - "rbf", "poly" and "linear" and the values of degree used are 2 and 3. By following the grid search, there are 3 (kernels) x 2 (degrees) = 6 different combinations of hyperparameters to be verified to find the best curve for each tool [68].

Loss function. A neural network learns patterns from data by minimising a loss function. Cross-entropy is a popular choice for a loss function in classification problems [69]. In our approach, cross-entropy function is used in the GRU neural network to compute the loss between the true and predicted label and is weighted by the label's weight. The loss is summed up over all labels of a tool sequence and then averaged (Equation 3). The term T is the total number of labels (size of the label bit vector). The term w_i is the weight of the ith label. The terms p^a and p^b refer to the true and predicted label vectors for a tool sequence, respectively. In general, the loss is large when p^a and p^b are far away from each other which means that the learning by the neural network is not good. If they are close, the loss is low and the predictions are better. When an unweighted

cross-entropy is used as the loss function for any classification problem [70] then it is assumed that all the predictions have the same weight and it does not differentiate between the more and less dominant labels. In our approach when it is used as a loss function in the neural network, then even though the predicted labels are correct they may not necessarily have large weights and thereby maybe less relevant. Therefore, to reduce the possibility of less relevant labels appearing in recommendations, loss is weighted by the weights of labels. It means that if a label with a larger weight is misclassified, which means that the true and predicted values are different, then the overall loss is higher. In this way, the wrong classification of a label with a larger weight is penalised more than the wrong classification of a label with a smaller weight.

$$loss = -\frac{1}{T}\sum_{i=1}^{T}(p_i^a \cdot \log(p_i^b) + (1 - p_i^a) \cdot \log(1 - p_i^b)) * w_i \quad (3)$$

The loss in Equation 3 is computed for all tool sequences in training data and is minimised using a root mean square propagation (RMSProp) optimiser. It follows an adaptive approach to estimate the learning rate by keeping knowledge of gradients in prior iterations. The learning rate is updated by dividing it with an average of the square of the prior gradients [71].

Hyperparameter tuning. A neural network has multiple hyperparameters. In our approach, they are the number of dimensions of the embedding layer, learning and dropout rates, number of units for the GRU layer and size of batches. They should be optimised to find the best configuration (a combination of hyperparameters) for training on tool sequences as a different configuration may give a different performance on the same training data. The grid and random searches are popular techniques to optimise hyperparameters. One limitation of these approaches is that they evaluate each configuration independently and have a high time complexity to find the best configuration. Therefore, the hyperparameters in our approach are optimised using a Bayesian (sequential model-based) optimisation [72]. It learns from the previously evaluated configurations which ensures faster convergence. Reasonable ranges of all the hyperparameters to be optimised are given and the best configuration is found after 20 evaluations.

Learning and predictions

Over 229,000 tool sequences collected from more than 18,000 workflows are divided into training and test data. A neural network learns patterns in the tool sequences from the training data and creates a model. The ability of the model to recommend tools is evaluated on the test data which is unseen by the neural network during training. The training data forms 80% (approximately 185,000) of all tool sequences and it is iterated over 10 epochs of neural network training. The remaining 20% (approximately 45,000) is used as the test data. The running time of the training is approximately 50 hours on a high performance compute cluster provided by bwCloud [73] with multiple cores. After learning on the training data, the model is used to predict tools. Each predicted tool gets a probability score of being the recommended tool of a tool or tool sequence. Two sets of predictions are made - standard and normal. Each set is sorted in the descending order of their probabilities and the top ones in both sets are combined to show them as recommendations.

Summary and future work

A system to recommend tools in Galaxy is built by analysing workflows using a variant of RNN (GRU) and a weighted cross-entropy loss function. The recommended tools are relevant for multiple scientific analyses with high accuracy as shown by the high similarities between the tools used in GTN tutorials and the recommended tools for similar analyses (Table 1). Moreover, they are easily accessible through simple UI integrations in Galaxy (Figures 6 and 7). Collectively, they improve user experience by helping researchers to easily create correct workflows. In addition, the approach does not store any information about tools and the recommendations are made by learning only the patterns of tool connections in workflows. The model [74] created using this approach and an API [75] are integrated into the European Galaxy server. The API resides with other Galaxy APIs and accesses a tool or a tool sequence specified by researchers to show its recommendations in real time using the model. The API is used at two different user interfaces in Galaxy - one shows recommendations in the workflow editor (Figure 6) and another shows them after each tool execution (Figure 7). The recommendation system should be potentially helpful for those researchers who are new to the Galaxy platform. It shows them a few follow-up tools from a big collection of more than 3,000 tools and enables them to perform multiple exploratory data analyses.

Different Galaxy servers maintain different sets of tools and workflows. The current approach can be used to create different recommendation models for different Galaxy servers. Alternatively, all the workflows can be collected from multiple Galaxy servers and using the current approach, one recommendation model can be created by learning on the complete set of workflows and the model can be distributed to different Galaxy servers. To improve the quality of recommendations, the annotations of tools can be incorporated in the learning mechanism by assigning higher weights to the annotated tools in comparison to tools which are not annotated. Tools containing similar annotations may have similar functionalities and using these similarities, tool recommendations can be further enhanced by showing similar tools for each recommended tool. In addition to learning tool connections to recommend tools, the knowledge of tools connecting to different tools based on their respective parameters can also be incorporated.

Methods

Multiple neural network architectures

Multiple neural network architectures such as convolutional neural network (CNN) and dense neural network (DNN) with only dense layers, are used to compare their respective predictive strengths with the GRU neural network (Figures 3, 4 and 5). In these architectures too, the embedding layer is used as the first (input) layer and a dense layer is used as an output layer having the same dimensions as the number of tools. Additionally, in CNN, convolutional and max-pooling layers are used to learn spatial patterns in tool sequences and downsample the dimensionality of input, respectively. Moreover, two dense layers are also used and the last one serves as an output layer. DNN uses two dense layers as hidden layers. The cross-entropy, with and without weights, is used as the loss function and RMSProp is used as an optimiser. Bayesian optimisation is used to optimise the hyperparameters of these architectures. More details about the architectures of DNN and CNN are given in section S3 of the supplementary document.

Library, model and code repositories

The Keras deep learning library is used for producing the neural network architectures [76]. The trained model [74] is saved as an H5 file to simplify its distribution to different Galaxy instances. The file is an HDF5 store containing the weights of different layers of the neural network and their configurations, a dictionary of tools and their indices and the weights of tools. The weights and configuration of the neural network are needed to recreate the trained model. The dictionary is used to replace IDs of the predicted tools by their indices in a tool sequence. All data and python scripts used in our approach are stored at Github for all approaches - GRU [77], CNN [78] and DNN [79]. In each of these repositories, the process to create a tool recommendation model is explained. All these repositories are provided with a script ("extract_data.sh") for collecting raw input datasets from a Galaxy instance. These datasets are workflows and usage frequencies of tools and are also provided in each repository. The values of multiple hyperparameters of neural networks, number of training iterations and sizes of training and test data can be altered using a bash script ("train.sh"). To execute the scripts on a GPU enabled machine, the "tensorflow-gpu" package should be installed instead of "tensorflow" as mentioned in the conda package dependencies file ("environment.yml"). To see recommended tools, an ipython script ("tool_recommendation_gru_wc.ipynb" for GRU repository) is also provided which loads and recreates a trained model to predict tools for a tool or a tool sequence. The result files storing precision, training and validation losses and usage frequencies, which are used for generating line plots (Figures 3, 4 and 5), for all approaches are also available at Github [80]. The code repositories of two other approaches which do not use neural networks are available at - simple approach [31] and ExtraTrees [32].

New recommendation model

On a usual Galaxy server, tools and workflows are dynamic as they are added and updated regularly. Therefore, it is important to train the GRU neural network on the complete set of workflows periodically to keep the tool recommendation model updated with the latest tools and workflows. Using a Galaxy tool [81], a new recommendation model can be created after collecting workflows and tool usage data from a Galaxy server. The tool runs for several hours (> 24 hours) and creates a model which is pushed to an online repository [74]. From this repository, Galaxy downloads it using an API [75] to recommend tools. The recommendation model is created periodically every 3-4 months to accommodate new workflows and tools. Galaxy admins can decide upon the frequency of creating a new model. It can be created every month or every 6 months

New tools as recommendations

Galaxy admins can overwrite the recommended tools predicted using the trained model by a different set of tools using the configuration option described in [82]. In addition, to highlight the newly added tools, which are not part of the model, they can be appended to the recommendations using this additional configuration option.

Availability of supporting source code and requirements

Project name: Tool recommender in Galaxy using deep learning
Project home page: https://github.com/anuprulez/galaxy_tool_recommendation
Operating system: Linux
Programming languages: Python, XML, JavaScript
Other requirements: Tensorflow, Keras, Scikit-learn, Numpy, H5py, Csvkit, Hyperopt
License: MIT License
RRID: SCR_018491
Biotools ID: tool_recommender_system_in_galaxy

Additional files

File name: Tool_recommendations_supplementary_file.pdf
Title: Supplementary Material: Tool recommender system in Galaxy using deep learning Description: There 3 tables - Table 1 shows a comparison of recommendations between the GRU neural network, a simple model and ExtraTrees classifier, Table 2 shows a comparison of recommendations between the regularised and non-regularised GRU neural network and Table 3 shows the strategy of uniform sampling of training data. There are 7 figures - Figures 1 and 2 show architectures of convolutional neural network (CNN) and dense neural network (DNN) used in the paper, Figure 3 shows original frequencies of last tools in training data, Figure 4 shows the frequencies of last tools in training data after uniform sampling, Figures 5 and 6 show top-1 normal and standard precision respectively for less frequent tools and Figure 7 shows the top-1 and top-2 precision (normal and standard recommendations) of the ExtraTrees classifier.

Declarations

List of abbreviations

API: Application programming interface; CNN: Convolutional neural network; DNN: Dense neural network; ELU: Exponential linear units; GRU: Gated recurrent units; PROPHETS: Process realisation and optimisation platform using human-readable expression of temporal-logic synthesis; RNN: Recurrent neural network; SVR: Support vector regression; UI: User interface; WINGS: Workflow instance generation and specialization;

Ethics approval and consent to participate

Not applicable

Consent for publication

Not applicable

Competing interests

The authors declare that they have no competing interests.

Funding

This work was supported by the German Research Foundation (DFG) under Germany's Excellence Strategy (CIBSS - EXC-2189 - Project ID 390939984) and German Federal Ministry of Education and Research (BMBF grant 031A538A de.NBI).

Authors' contributions

First author (A.K.) implemented the project and wrote the manuscript. The second author (H.R.) wrote scripts for data collection, contributed to the manuscript and deployed the project on European Galaxy server. The third author (B.G.) provided the idea of the project, validated results, and contributed to the manuscript. The last author (R.B.) contributed to the manuscript. All authors approved the manuscript.

Acknowledgements

We thank Simon Bray and Joachim Wolff for proofreading the manuscript, Dr. Wolfgang Maier for providing feedback and Gianmauro Cuccuru for deploying it on the European Galaxy server.

References

1. Ewels P, Krueger F, Käller M, Andrews S. Cluster Flow: A user-friendly bioinformatics workflow tool. F1000Research 2017;5:2824, doi:10.12688/f1000research.10335.2.
2. Leipzig J. A review of bioinformatic pipeline frameworks. Brief Bioinform 2017;1;18(3):530-536, doi:10.1093/bib/bbw020.
3. Baichoo S, Souilmi Y, Panji S, Botha G, et al. Developing reproducible bioinformatics analysis workflows for heterogeneous computing environments to support African genomics. BMC Bioinformatics 2018;19, 457 (2018), doi:10.1186/s12859-018-2446-1.
4. Love MI, Huber W, Anders S. Moderated estimation of fold change and dispersion for RNA-seq data with DESeq2. Genome Biol 2014;550. doi:10.1186/s13059-014-0550-8.
5. Afgan E, Baker D, Batut B, et al. The Galaxy platform for accessible, reproducible and collaborative biomedical analyses: 2018 update. Nucleic Acids Research 2018;46(W1):W537-W544. doi:10.1093/nar/gky379.
6. Bela G, Beel J, Hentschel C. Scienstein : A Research Paper Recommender System. In: Proceedings of the International Conference on Emerging Trends in Computing; 2009. p. 309-315. http://www.sciplore.org/wp-content/papercite-data/pdf/gipp09.pdf.
7. Achakulvisut T, Acuna DE, Ruangrong T, Kording K. Science Concierge: A Fast Content-Based Recommendation System for Scientific Publications. PLoS ONE 2016;11(7): e0158423, doi:10.1371/journal.pone.0158423.
8. Wang D, Liang Y, Xu D, et al. A content-based recommender system for computer science publications. Knowledge-Based Systems 2018;157:1-9. doi:10.1016/j.knosys.2018.05.001.
9. SGomez-Uribe CA, Hunt N. The Netflix Recommender System: Algorithms, Business Value, and Innovation. ACM Transactions on Management Information Systems TMIS 2016;6(4).
10. Smith B, Linden G. Two Decades of Recommender Systems at Amazon.com. IEEE Internet Computing 2017;21(3):12-18. doi:10.1109/MIC.2017.72.
11. Palmblad M, L LA, Ison J, Schwämmle V. Automated workflow composition in mass spectrometry-based proteomics. Bioinformatics 2019;35(4):656-664. doi:10.1093/bioinformatics/bty646.
12. Naujokat S, Lamprecht AL, Steffen B. Loose Programming with PROPHETS, Fundamental Approaches to Software Engineering, vol. 7212. J L, A Z, editors, Springer, Berlin, Heidelberg; 1996. Online ISBN 978-3-642-28872-2, doi: 10.1007/978-3-642-28872-2_7.
13. Gil Y, Ratnakar V, Kim J, et al. Wings Intelligent Workflow-Based Design of Computational Experiments. IEEE Intelligent Systems 2011;26(1):62-72. doi:10.1109/MIS.2010.9.
14. Srivastava A, Adusumilli R, Boyce H, et al. Semantic workflows for benchmark challenges: Enhancing comparability, reusability and reproducibility. PSB 2018;doi:10.1142/9789813279827_0019.
15. DiBernardo M, Pottinger R, Wilkinson M. Semi-automatic web service composition for the life sciences using the biomoby semantic web framework. Journal of Biomedical Informatics 2008;41(5):837-847. doi:10.1016/j.jbi.2008.02.005.
16. Michalski V, Memisevic R, Konda KR. Modeling sequential data using higher-order relational features and predictive training. CoRR 2014;abs/1402.2333. http://arxiv.org/abs/1402.2333.
17. Yin W, Kann K, Yu M, Schütze H. Comparative Study of CNN and RNN for Natural Language Processing. ArXiv 2017;abs/1702.01923. http://arxiv.org/abs/1702.01923.
18. Lipton ZC, Kale DC, Elkan C, Wetzel R. Learning to diagnose with LSTM recurrent neural networks. CoRR 2015;abs/1511.03677.
19. Chung J, Gulcehre C, Cho K, Bengio Y. Empirical evaluation of gated recurrent neural networks on sequence modeling. In: NIPS 2014 Workshop on Deep Learning, December 2014; 2014. .
20. Boulanger-Lewandowski N, Bengio Y, Vincent P. Modeling Temporal Dependencies in High-Dimensional Sequences: Application to Polyphonic Music Generation and Transcription. Proceedings of the 29th International Conference on Machine Learning, ICML 2012;2.
21. Karan S, Zola J. Exact structure learning of Bayesian networks by optimal path extension. IEEE International Conference on Big Data 2016;p. 48-55. doi:10.1109/BigData.2016.7840588.
22. Spirtes P, Glymour C, Scheines R, et al. Constructing Bayesian Network Models of Gene Expression Networks from Microarray Data, Research Showcase @ CMU. Semantic Scholar 2018;doi:10.1184/R1/6491291.v1.
23. Chickering DM. Learning Bayesian Networks is NP-Complete, vol. 112. D F, HJ L, editors, New York, NY: Springer; 1996. ISBN: 978-1-4612-2404-4, doi:10.1007/978-1-4612-2404-4_12.
24. Chickering DM, Heckerman D, Meek C. Large-Sample Learning of Bayesian Networks is NP-Hard. Journal of Machine Learning Research 2004;5:1287-1330.
25. Cooper GF. The computational complexity of probabilistic inference using bayesian belief networks. Artificial Intelligence 1990;42:393-405. doi:10.1016/0004-3702(90)90060-D.
26. European Galaxy Server. https://usegalaxy.eu/ (2020);. Accessed 22 July 2020.
27. Jian X, Wickramarathne TL, Chawla NV. Representing higher-order dependencies in networks. Science Advances 2016;2(5). doi:10.1126/sciadv.1600028.
28. Said A, Bellogín Kouki A, de Vries AP. A Top-N Recommender System Evaluation Protocol Inspired by Deployed Systems. In: In Proceedings of the 2013 ACM RecSys Workshop on Large-Scale Recommender Systems. LSRS; 2013. .
29. Kang Z, Peng C, Cheng Q. Top-N Recommender System via Matrix Completion. In: Proceedings of the Thirtieth AAAI Conference on Artificial Intelligence (AAAI-16); 2016. .
30. Deshpande M, Karypis G. Item-Based Top-N recommender Algorithms. ACM Transactions on Information Systems 2004;22(1):143-177. doi:10.1145/963770.963776.
31. Kumar A, Tool Recommender in Galaxy using stored tool

sequences. 2020;. https://github.com/anuprulez/galaxy_tool_recommendation/tree/statistical_model. Accessed 22 July 2020.

32. Kumar A, Tool recommender system in Galaxy using extra trees classifier. 2020;. https://github.com/anuprulez/galaxy_tool_recommendation/tree/sklearn_rf. Accessed 22 July 2020.
33. Smith T, Heger A, Sudbery I. UMI-tools: modeling sequencing errors in Unique Molecular Identifiers to improve quantification accuracy. Genome Research 2017;27(3):491–499. doi:10.1101/gr.209601.116.
34. Butler A, Hoffman P, Smibert P, Papalexi E, Satija R. Integrating single-cell transcriptomic data across different conditions, technologies, and species. Nature Biotechnology 2018;36(5):411–420. doi:10.1038/nbt.4096.
35. D G, A L, L K, K W, O B, N S, et al. Single-cell messenger RNA sequencing reveals rare intestinal cell types. Nature 2015;525(7568):251–255. doi:10.1038/nature14966.
36. A D, CA D, F S, et al. STAR: ultrafast universal RNA-seq aligner. Bioinformatics 2013;29(1):15–21. doi:10.1093/bioinformatics/bts635.
37. Liao Y, Smyth GK, Shi W. featureCounts: an efficient general purpose program for assigning sequence reads to genomic features. Bioinformatics 2013;30 (7):923–930. doi:10.1093/bioinformatics/btt656.
38. P E, M M, S L, M K. MultiQC: summarize analysis results for multiple tools and samples in a single report. Bioinformatics 2016;32(19):3047–3048. doi:10.1093/bioinformatics/btw354.
39. Wang L, Wang S, Li W. RSeQC: quality control of RNA-seq experiments. Bioinformatics 2012;28(16):2184–2185. doi:10.1093/bioinformatics/bts356.
40. Bolger AM, Lohse M, Usadel B. Trimmomatic: a flexible trimmer for Illumina sequence data. Bioinformatics 2014;30:2114–2120.
41. Li H. Aligning sequence reads, clone sequences and assembly contigs with BWA-MEM. ArXiv 2013 03;1303.
42. Garrison E, Marth G, Haplotype-based variant detection from short-read sequencing; 2012. https://arxiv.org/abs/1207.3907.
43. Langmead B, Trapnell C, Pop M, et al. Ultrafast and memory-efficient alignment of short DNA sequences to the human genome. Genome Biol 2009;10. https://doi.org/10.1186/gb-2009-10-3-r25.
44. Li H, Handsaker B, Wysoker A, Fennell T, Ruan J, Homer N, et al. The Sequence Alignment/Map format and SAMtools. Bioinformatics 2009;25 (16):2078–2079. doi:10.1093/bioinformatics/btp352.
45. Garrison E, A C++ library for parsing and manipulating VCF files.;. https://github.com/vcflib/vcflib. 2015. Accessed 02 August 2020.
46. Ramirez F, Ryan DP, Gruening B, Bhardwaj V, Kilpert F, Richter AS, et al. deepTools2: a next generation web server for deep-sequencing data analysis. Nucleic Acids Research 2016;44 (W1:W160–W165. doi:10.1093/nar/gkw257.
47. O'Boyle NM, Banck M, James CA, Morley C, Vandermeersch T, Hutchison GR. Open Babel: An open chemical toolbox. Journal of Cheminformatics 2011;3 (1). doi:10.1186/1758-2946-3-33.
48. Bray S, Protein-ligand docking (Galaxy Training Materials); 2020. https://training.galaxyproject.org/training-material/topics/computational-chemistry/tutorials/cheminformatics/tutorial.html, [Online; accessed 22 July 2020].
49. Ramirez F, Wolff J, Gruening B, Bhardwaj V, Ryan D, Duendar F, Deeptools/Hicexplorer: Winter Release;. https://zenodo.org/record/1133705. 2017. Accessed 02 August 2020.
50. Wolff J, Ramirez F, Bhardwaj V, Polkh E, Hi-C analysis of Drosophila melanogaster cells using HiCExplorer (Galaxy Training Materials); 2020. https://training.galaxyproject.org/training-material/topics/epigenetics/tutorials/hicexplorer/tutorial.html, [Online; accessed 22 July 2020].
51. Dündar F, Erxleben A, Batut B, Bhardwaj V, Ramirez F, Rabbani L, Formation of the Super-Structures on the Inactive X (Galaxy Training Materials); 2019. https://training.galaxyproject.org/training-material/topics/epigenetics/tutorials/formation_of_super-structures_on_xi/tutorial.html, [Online; accessed 22 July 2020].
52. Kumar A, Khanteymoori A, Introduction to deep learning (Galaxy Training Materials); 2020. https://training.galaxyproject.org/training-material/topics/statistics/tutorials/intro_deep_learning/tutorial.html, [Online; accessed 22 July 2020].
53. Kessner D, Chambers M, Burke R, Agus D, Mallick P. ProteoWizard: open source software for rapid proteomics tools development. Bioinformatics 2008;24 (21):2534–2536. doi:10.1093/bioinformatics/btn323.
54. Sigloch FC, Grüning B, Peptide and Protein ID using OpenMS tools (Galaxy Training Materials); 2020. https://training.galaxyproject.org/training-material/topics/proteomics/tutorials/protein-id-oms/tutorial.html, [Online; accessed 22 July 2020].
55. Marcel M. Cutadapt removes adapter sequences from high-throughput sequencing reads. EMBnetjournal 2011;17 (1). doi:10.14806/ej.17.1.200.
56. Batut B, Freeberg M, Heydarian M, Erxleben A, Videm P, Blank C, et al., Reference-based RNA-Seq data analysis (Galaxy Training Materials); 2020. https://training.galaxyproject.org/training-material/topics/transcriptomics/tutorials/ref-based/tutorial.html, [Online; accessed 22 July 2020].
57. Tekman M, Batut B, Erxleben A, Maier W, Pre-processing of Single-Cell RNA Data (Galaxy Training Materials); 2020. https://training.galaxyproject.org/training-material/topics/transcriptomics/tutorials/scrna-preprocessing/tutorial.html, [Online; accessed 22 July 2020].
58. Tekman M, Ostrovsky A, Downstream Single-cell RNA analysis with RaceID (Galaxy Training Materials); 2020. https://training.galaxyproject.org/training-material/topics/transcriptomics/tutorials/scrna-raceid/tutorial.html, [Online; accessed 22 July 2020].
59. Nekrutenko A, Soranzo N, Calling variants in diploid systems (Galaxy Training Materials); 2019. https://training.galaxyproject.org/training-material/topics/variant-analysis/tutorials/dip/tutorial.html, [Online; accessed 22 July 2020].
60. Batut B, et al. Community-Driven Data Analysis Training for Biology. Cell Systems 2018;6:752–758.E1. https://training.galaxyproject.org/, doi:10.1016/j.cels.2018.05.012.
61. Tsoumakas G, Katakis I. Multi-label classification: An overview. International Journal of Data Warehousing and Mining 2009;3:1–13. doi:10.4018/jdwm.2007070101.
62. Ruiz-Carmona S, Alvarez-Garcia D, Foloppe N, Garmendia-Doval AB, Juhos S, Schmidtke P, et al. rDock: A Fast, Versatile and Open Source Program for Docking Ligands to Proteins and Nucleic Acids. PLoS Computational Biology 2014;10 (4). doi:10.1371/journal.pcbi.1003571.
63. Pascanu R, Mikolov T, Bengio Y. Understanding the exploding gradient problem. ArXiv 2012;abs/1211.5063.
64. Zaremba W, Sutskever I, Vinyals O. Recurrent Neural Network Regularization. ArXiv 2014;abs/1409.2329.
65. Gal Y, Ghahramani Z. A Theoretically Grounded Application of Dropout in Recurrent Neural Networks. In: Pro-

ceedings of the 30th International Conference on Neural Information Processing Systems; 2016. p. 1027–1035.
66. Clevert D, Unterthiner T, Hochreiter S. Fast and Accurate Deep Network Learning by Exponential Linear Units (ELUs). CoRR 2015;abs/1511.07289.
67. Nair V, Hinton GE. Rectified Linear Units Improve Restricted Boltzmann Machines. In: ICML'10: Proceedings of the 27th International Conference on International Conference on Machine Learning, June 2010; 2010. p. 807–814.
68. Pedregosa F, Varoquaux G, Gramfort A, et al. Scikit-learn: Machine Learning in Python. Journal of Machine Learning Research 2011;12:2825–2830. doi:10.5555/1953048.2078195.
69. Janocha K, Czarnecki W. On Loss Functions for Deep Neural Networks in Classification. ArXiv 2017;abs/1702.05659. doi:10.4467/20838476SI.16.004.6185.
70. Sadowski P, Notes on Backpropagation. https://www.ics.uci.edu/~pjsadows/notes.pdf (2016). Accessed 22 July 2020;.
71. Ruder S. An overview of gradient descent optimization algorithms. ArXiv 2016;abs/1609.04747.
72. Bergstra J, Yamins D, Cox DD. Hyperopt: A Python Library for Optimizing the Hyperparameters of Machine Learning Algorithms. 12th Python in science conf (SCIPY 2013) 2013;.
73. BwCluster. https://portal.bw-cloud.org/project/ (2020);. Accessed 22 July 2020.
74. Kumar A, Tool Recommender model;. https://github.com/galaxyproject/galaxy-test-data/blob/master/tool_recommendation_model.hdf5. 2020. Accessed 29 July 2020.
75. Kumar A, Get tool predictions. 2020;. https//github.com/usegalaxy-eu/galaxy/blob/release_20.05_europe/lib/galaxy/webapps/galaxy/api/workflows.py#L638. Accessed 22 July 2020.
76. Chollet F, et al. Keras 2015;.
77. Kumar A, Tool Recommender in Galaxy using GRU neural network. 2020;. https://github.com/anuprulez/galaxy_tool_recommendation. Accessed 22 July 2020.
78. Kumar A, Tool Recommender in Galaxy using CNN neural network. 2020;. https://github.com/anuprulez/galaxy_tool_recommendation/tree/cnn_wc. Accessed 22 July 2020.
79. Kumar A, Tool Recommender in Galaxy using DNN neural network. 2020;. https://github.com/anuprulez/galaxy_tool_recommendation/tree/dnn_wc. Accessed 22 July 2020.
80. Kumar A, Output results files. 2020;. https://github.com/anuprulez/galaxy_tool_recommendation/tree/master/output_files/data_20_05. Accessed 22 July 2020.
81. Kumar A, Tool Recommender model creator;. https://usegalaxy.eu/root?tool_id=toolshed.g2.bx.psu.edu/repos/bgruening/create_tool_recommendation_model/create_tool_recommendation_model/0.0.3. 2020. Accessed 22 July 2020.
82. Kumar A, Tool Recommender overwrite;. https://github.com/usegalaxy-eu/galaxy/blob/release_20.05_europe/config/tool_recommendations_overwrite.yml.sample. 2020. Accessed 30 July 2020.

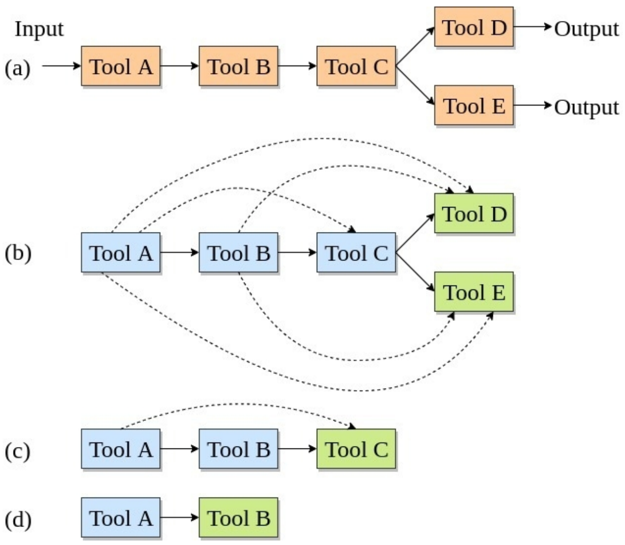

Figure 1. An example workflow (a) is shown consisting of 5 different tools which is decomposed into multiple tool sequences shown in (b), (c) and (d). Each tool sequence shows higher-order dependencies where a tool is dependent on all of its prior tools. These dependencies are shown by the dashed arrows.

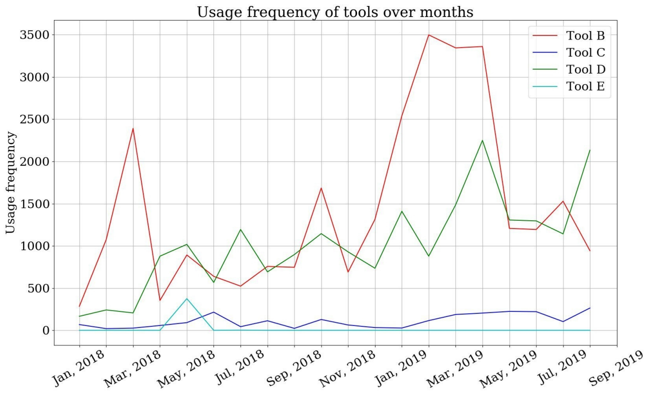

Figure 2. The plot shows the usage frequencies of 4 tools collected over the past one year. The Tools B and D have high usage frequencies almost every month while the Tools C and E have much lower usage frequencies compared to Tools B and D. The tool A is absent from the plot because it is not the label of any tool for the workflow shown in Figure 1.

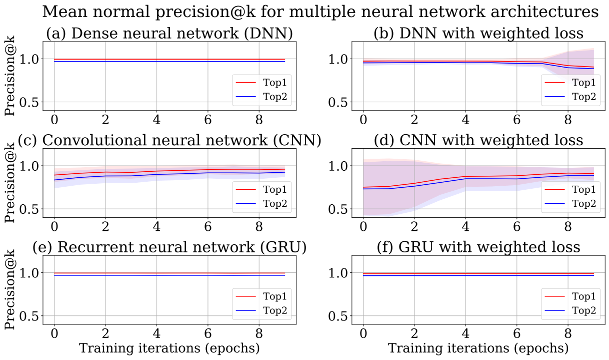

Figure 3. The subplots (a), (c) and (e) show top-k (precision@k) normal precision for DNN, CNN and GRU neural networks with cross-entropy loss function, respectively. The subplots (b), (d) and (f) show top-k (precision@k) normal precision for DNN, CNN and GRU neural networks with weighted cross-entropy loss function, respectively.

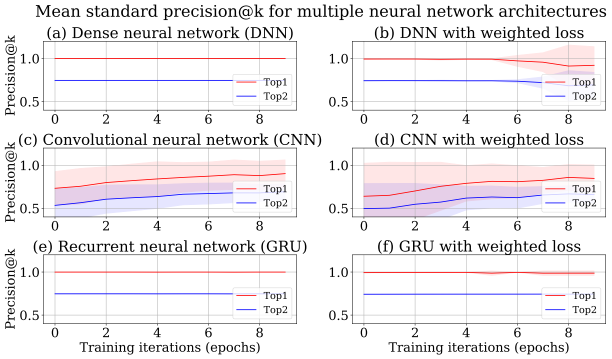

Figure 4. The subplots (a), (c) and (e) show top-k (precision@k) standard precision for DNN, CNN and GRU neural networks with cross-entropy loss function, respectively. The subplots (b), (d) and (f) show top-k (precision@k) standard precision for DNN, CNN and GRU neural networks with weighted cross-entropy loss function, respectively.

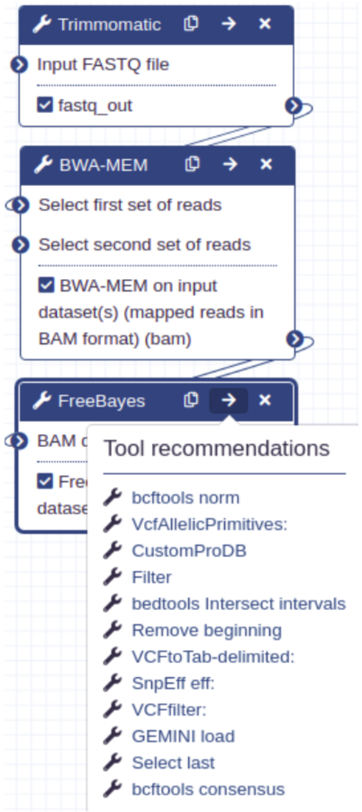

Figure 6. The figure shows recommended tools, listed in a dropdown with "Tool recommendations" as its header, in the workflow editor of the European Galaxy server for "Trimmomatic → BWA-MEM → FreeBayes" tool sequence. The recommended tools for the tool sequence can be seen in a dropdown while hovering on the right arrow button placed in the top-right corner of the "FreeBayes" tool. Clicking on any recommended tool such as "bcftools norm" in the dropdown opens a new block for the chosen tool which can be connected to the tool sequence.

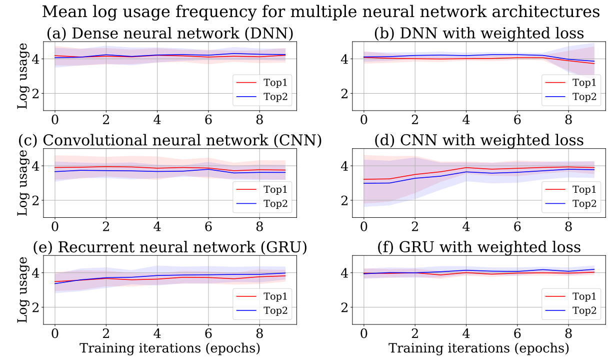

.

Figure 5. The subplots (a), (c) and (e) show usage frequencies of (top-k) predicted tools for DNN, CNN and GRU neural networks with cross-entropy loss function, respectively. The subplots (b), (d) and (f) show usage frequencies of (top-k) predicted tools for DNN, CNN and GRU neural networks with weighted cross-entropy loss function, respectively.

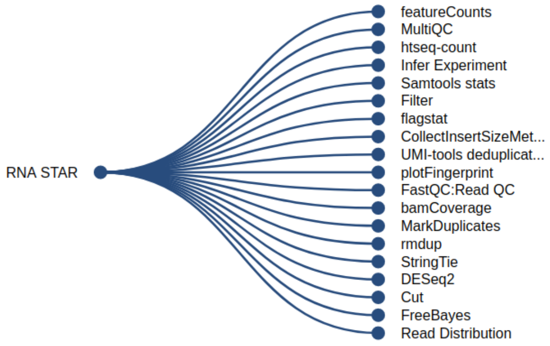

Figure 7. The figure shows recommended tools as leaves (on the right) of the tree after executing RNA-STAR tool. Clicking on any recommended tool opens its definition in Galaxy and can be used for further analysis with the data files produced by the previous tool (RNA-STAR).

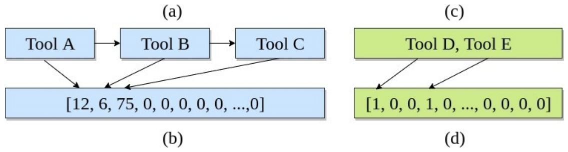

Figure 8. The figure shows how a tool sequence and its labels are transformed into vectors.

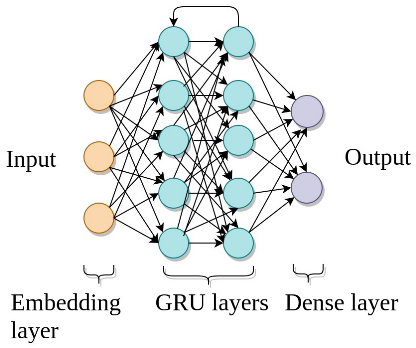

Figure 9. The figure shows the architecture of the GRU neural network. It has four components as layers. The first layer is the input layer (yellow), two stacked layers of GRU (cyan) and the last layer is the output layer (violet). The dropout layers are added between embedding and GRU layers, between two GRU layers and between the second GRU and dense layers.

Reference manuscript
Click here to access/download;Manuscript;GigaScience_Tool_Recommend

GigaScience, 0000, 1–12

doi: xx.xxxx/xxxx
Manuscript in Preparation
Technical Note

TECHNICAL NOTE

Tool recommender system in Galaxy using deep learning

Anup Kumar[1,*,†], Helena Rasche[1, ‡, †], Björn Grüning[1, §, †] and Rolf Backofen[1,2, ¶, †]

[1]Bioinformatics Group, Department of Computer Science, University of Freiburg, Georges-Koehler-Allee 106, 79110 Freiburg, Germany and [2]Signalling Research Centres BIOSS and CIBSS, University of Freiburg, Schaenzlestr. 18, 79104 Freiburg, Germany

[*]kumara@informatik.uni-freiburg.de
[‡]helena.rasche@gmail.com
[§]gruening@informatik.uni-freiburg.de
[¶]backofen@informatik.uni-freiburg.de
[†]Contributions follow the order of the names of authors

Abstract

Background Galaxy is a web-based and open-source scientific data-processing platform. Researchers compose pipelines in Galaxy to analyse scientific data. These pipelines, also known as workflows, can be complex and difficult to create from thousands of tools, especially for researchers new to Galaxy. To help researchers with creating workflows, a system is developed to recommend tools that can facilitate further data analysis. **Findings** A model is developed to recommend tools using a deep learning approach by analysing workflows composed by researchers on the European Galaxy server. The higher-order dependencies in workflows, represented as directed acyclic graphs, are learned by training a gated recurrent units (GRU) neural network, a variant of a recurrent neural network (RNN). In the neural network training, the weights of tools used are derived from their usage frequencies over time and the sequences of tools are uniformly sampled from training data. Hyperparameters of the neural network are optimised using Bayesian optimisation. Mean accuracy of 98% in recommending tools is achieved for the top-1 metric. **Conclusions** The model is accessed by a Galaxy API to provide researchers with recommended tools in an interactive manner using multiple user interface (UI) integrations on the European Galaxy server. Good quality and highly-used tools are shown at the top of the recommendations. The scripts and data to create the recommendation system are available under MIT license at https://github.com/anuprulez/galaxy_tool_recommendation.

Key words: Recommender system; Galaxy; Workflows; Deep learning; Neural networks; Gated recurrent units

Findings

Background

Life sciences depend increasingly on high-throughput data, turning them into data science to a large extent. However, raw high-throughput data does not have much value on its own without proper analysis and interpretation of the data. To simplify the data analysis process and to ensure a reproducible analysis, several workflow systems such as Bcbio-nextgen, Omics Pipe, Nextflow, Luigi, Toil and many others have emerged [1, 2, 3]. The main idea for workflow systems is based on the observation that any computational analysis of high-throughput data encompasses multiple steps such as quality control, preprocessing, quantification and statistical analysis to transform raw data into scientific results. Collec-

Compiled on: August 2, 2020.
Draft manuscript prepared by the author.

tively, these steps form a workflow where each step performs a definite transformation of the data, which can be performed using standardised tools. Using workflow for the analysis is simple and convenient and has several advantages. First, it is easy to replace individual tools by a newer version or to assess the influence of the associated step on the final result. Second, a workflow can be saved, shared and reused, which ensures reproducible research. Therefore, workflows are becoming essential in the analysis of scientific data and there are multiple platforms where researchers can create workflows for their analyses. However, a critical question is how to assess whether a generated workflow is state-of-art or even valid at all. To give a concrete example, one can use several real-valued input vectors (such as fluorescence-based measurement stemming from arrays), transform them into integer-based values in the first step and combine it with a tool that uses a count-based statistics (such as negative binomial distribution as used in DESeq2 [4]) to determine values that show high differential behaviour. While this workflow would run on a workflow system without problems and even produce some results, the generated results are not valid because of the wrong statistical model. Therefore, it is important to use a tool for each step in a workflow which can bring desired results. To make it possible, a system is needed which can recommend useful tools at each step while creating a workflow.

Galaxy and workflows

Galaxy is an open-source data processing platform which enables researchers to create and store their workflows for multiple scientific analyses [5]. A workflow in Galaxy is a directed acyclic graph and consists of one or many tool sequences to analyse scientific data such as DNA and RNA sequences. A tool consumes one or more data files as input and produces one or more data files as output and supports a number of formats of these input and output files. In workflows, the tools are connected one after another following a constraint that the adjacent tools must have compatible data types. In other words, the data types of output files of a tool should match the data types of input files of the following tool. Galaxy has thousands of accessible tools and acquiring familiarity and constructing workflows with these tools can be a complex and time-consuming task, especially for researchers new to Galaxy. To assist them in creating workflows and making them aware of the possible tools for further analyses, a recommender system is devised. The benefits of having such a system are manifold. First, it will avoid the loss of time spent in creating erroneous or less optimal workflows by choosing tools which may produce undesired results and thereby making researchers more efficient. Second, it will help researchers bypass the step of searching for tools separately, which will further reduce the time spent in creating workflows and at the same time increase the accessibility of tools. Third, it will promote good quality tools having higher usage frequencies in the past (last one year) to the top of the recommendations and downgrade those having lower usage frequencies to the bottom of the recommendations. It is achieved by assigning weights to tools which are derived from their usage frequencies over a period of time. Finally, it can be extended to promote the newly added tools in Galaxy by showing them alongside the recommended tools predicted using the neural network approach.

Recommender systems

The objective of having recommender systems in fields such as scientific literature search, online shopping, travel bookings, media-service providers and many other fields is to help people discover suitable, interesting and newly-released products. These recommended products are recognised based on the usage and purchasing patterns of people in the past. In the field of scientific literature search, the exponential increase in the number of published papers necessitates having a recommender system to help scientists explore relevant and recent papers quickly [6, 7, 8]. Recommender systems are significant in the world of commercial applications too. Companies such as Amazon and Netflix have appropriately used them to learn preferences of their respective customers in selecting products such as their favourite books or movies and to propose a few products out of a large catalogue. By enabling users and customers to discover reasonable and customised products, recommender systems have helped them grow as organisations [9, 10]. In short, recommender systems make it faster for users and customers to look through a few recommended products to find the most suitable ones. These successful implementations of recommender systems by organisations across the world working in diverse areas to assess the needs of their respective users in proposing relevant products motivated us to create a tool recommender system in Galaxy.

Related work

To simplify creating workflows for scientific analyses, a few approaches have been proposed which suggest alternative tools and workflows. EDAM (EMBRACE Data And Methods) and semantic annotations of tools are used to compose workflows automatically for mass-spectrometry based proteomics [11]. The annotations include the names, functionalities, input and output data types of tools. The PROPHETS (Process Realisation and Optimisation Platform using Human-readable Expression of Temporal-logic Synthesis) program generates suitable candidates of workflows which match the goal of the proposed workflow and its annotations [12]. WINGS (Workflow INstance Generation and Specialization) offers multiple variations of a workflow created using different tools. It makes use of the input parameters, types of datasets and functions of tools to build the variations [13, 14]. The approach used in [15] utilises data types to facilitate the automatic creation of workflows. All these approaches depend either on annotations or matching input and output data types of adjacent tools in workflows and they pose challenges such as the addition and maintenance of the meaningful annotations of tools and extracting input and output data types of adjacent tools. Moreover, these approaches have their workflow generation restricted to a few specific bioinformatics analyses such as proteomics or proteogenomics. In addition, they do not discuss the presence of higher-order relationships [16] in tool sequences of workflows. Our approach to recommend tools in workflows aims to overcome these challenges in the following manner. First, it does not require collecting and storing information about tools. Second, it takes into account the higher-order relationships among tools (Figure 1) in tool sequences. Finally, it incorporates workflows from multiple scientific analyses to produce the recommender system.

Sequential learning on workflows

Workflows, created by many researchers in Galaxy for different scientific analyses, are decomposed into numerous tool sequences (Figure 1). The sequential nature of these tool sequences where tools are connected one after another inspires us to apply similar learning techniques used for other sequential data such as text and speech. There are multiple studies in the fields of natural language processing, clinical research and speech recognition which apply deep learning techniques on se-

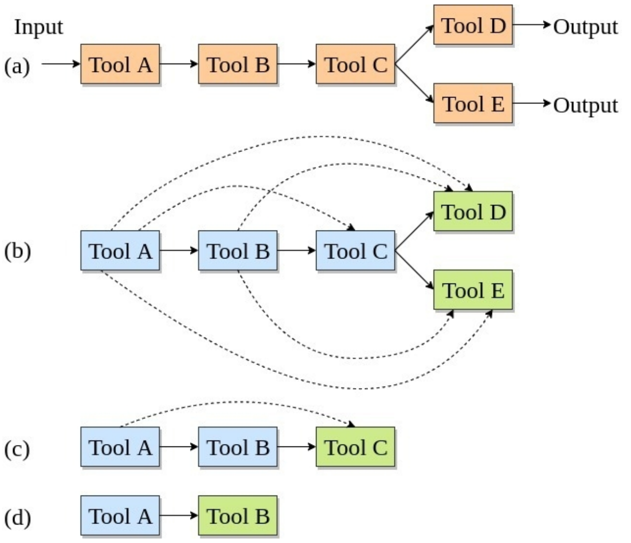

Figure 1. An example workflow (a) is shown consisting of 5 different tools which is decomposed into multiple tool sequences shown in (b), (c) and (d). Each tool sequence shows higher-order dependencies where a tool is dependent on all of its prior tools. These dependencies are shown by the dashed arrows.

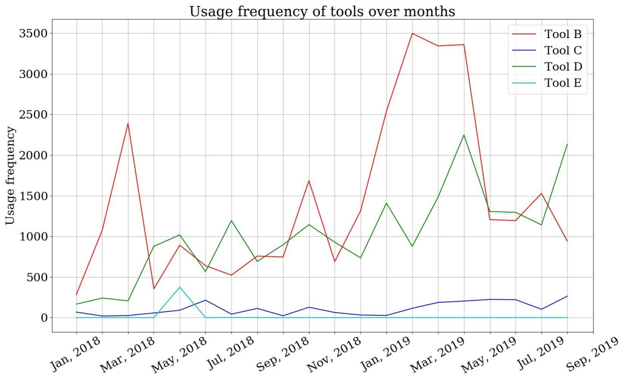

Figure 2. The plot shows the usage frequencies of 4 tools collected over the past one year. The Tools B and D have high usage frequencies almost every month while the Tools C and E have much lower usage frequencies compared to Tools B and D. The tool A is absent from the plot because it is not the label of any tool for the workflow shown in Figure 1.

quential data to obtain good accuracy in predicting future items. The approach used in [17] finds context in long sequences of words for sentiment analysis and part-of-speech tagging using recurrent neural network (RNN) and achieves 85% and 93% accuracy, respectively. For clinical data, learning on long sequences of health states proves to be beneficial [18]. The health states of patients recorded at different time points are analysed by accessing their electronic health records. The future health states of patients could be predicted by training RNN on the sequences of their past health states to achieve 85% accuracy. Moreover, the variants of RNN are used to model speech and music signals [19, 20]. These successful studies benefit from the sequential learning techniques using different variants of RNN. Therefore, in our work, a variant of RNN - gated recurrent units (GRU) - is used to create the tool recommender system in Galaxy.

A Bayesian network can also be used for modelling directed acyclic graphs (workflows) [21, 22]. It requires computing joint and conditional probabilities of nodes in graphs and an increase in the number of nodes can lead to a higher cost to compute these probabilities. In addition, making predictions by learning a probabilistic network is a hard problem [23, 24, 25]. Because of these drawbacks of using a Bayesian network, it is not used in our approach to create the recommender system in Galaxy.

Data description

Over 18,000 workflows from different scientific analyses such as RNA-seq, Variant-calling, Hi-C, Assembly, Single-cell, Proteomics and so on in the European Galaxy server [26] have been used to create the recommender system. A workflow consisting of 5 tools is shown in Figure 1a. It is divided into smaller tool sequences as shown in Figure 1b, 1c and 1d. The last tool, shown in green, of each tool sequence (of length n) is assigned as the label of the sub-sequence (of length n-1) shown in blue in Figure 1. A label is an output which is learned and predicted by the recommender system. In the neural network learning, a tool is a label. For example, in Figure 1b, Tools D and E are the labels of the sub-sequence Tool A $\rightarrow$ Tool B $\rightarrow$ Tool C. They show higher-order dependencies in their connections which implies that a tool is not only dependent on its immediate predecessor but also on all prior tools in the tool sequence. For example, in Figure 1c, the Tool C is dependent on Tools B and A. By analysing multiple workflow fragments in this way, the neural network should learn that the label of a tool sequence Tool A $\rightarrow$ Tool B is Tool C. It is expected that dividing a tool sequence into fragments with a minimum length of two tools, as shown in Figure 1c and 1d, will improve the generalisation performance of the neural network because it gets more tool sequences with a variety of lengths to learn from. The dependencies shown in Figure 1b, 1c and 1d present in tool sequences are learned using the GRU neural network by modelling the conditional probability given by Equation 1 [27]. Using the approach explained above, more than 229,000 tool sequences are extracted.

Tools in Galaxy have different usage patterns. Some tools are used more often than other tools for multiple reasons such as differences in their functions and availability of similar but better tools. It is essential to analyse the usage patterns of tools because the recommender system proposes tools for researchers and these tools should have high relevance to their analyses. One of the key indicators of relevance of tools can be their high usage frequencies. If a tool has been used often in the recent past, it implies that the tool is relevant. However, if a tool was used often a few years ago but has been used less often in the last six months then the relevance of that tool has certainly declined. The usage frequencies of tools (shown as labels in Figure 1) over the past year are shown in Figure 2.

The set of workflows may have poor quality workflows which are non-published or deleted or may have errors in their tool connections. To distinguish between good and poor quality tool connections, the labels for each tool sequence are divided into two parts - standard and normal labels. The standard labels come from the published, non-deleted and non-erroneous workflows while the normal labels come from other workflows. While recommending tools for a tool or tool sequence, the standard labels are promoted to the top of the recommendations if available followed by the normal labels. This should allow good quality tools to be shown as the top recommendations.

Tool sequences from these workflows may vary in number - some may occur more frequently and others may not. Therefore, the complete set of tool sequences may not be equally representative of all workflows coming from different scientific analyses. Learning on the imbalanced set of tool sequences can induce bias which may have an undesired outcome - good accuracy in recommending tools coming from highly frequent tool sequences and poor accuracy for tools coming from less

frequent ones. To mitigate this bias, all tool sequences are chosen with uniform frequency while training the neural network which should allow it to have a comparable accuracy in recommending tools in different scientific analyses. This uniform sampling strategy is discussed in the "Implementation" section in detail.

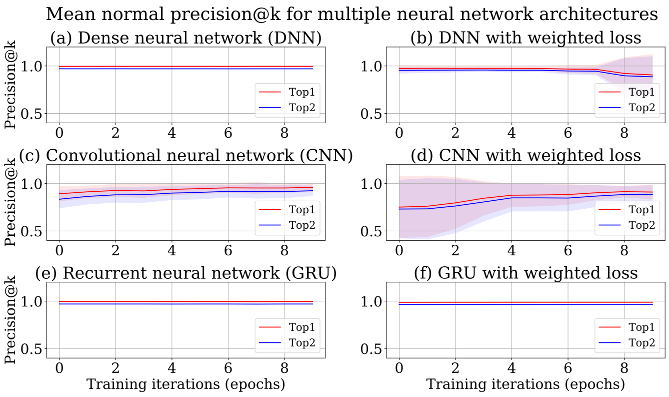

Figure 3. The subplots (a), (c) and (e) show top-k (precision@k) normal precision for DNN, CNN and GRU neural networks with cross-entropy loss function, respectively. The subplots (b), (d) and (f) show top-k (precision@k) normal precision for DNN, CNN and GRU neural networks with weighted cross-entropy loss function, respectively.

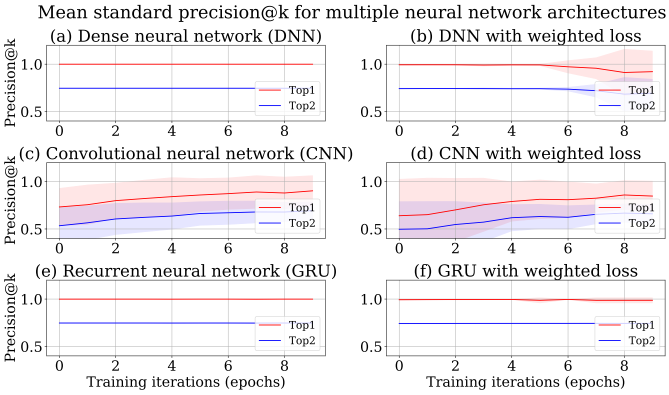

Figure 4. The subplots (a), (c) and (e) show top-k (precision@k) standard precision for DNN, CNN and GRU neural networks with cross-entropy loss function, respectively. The subplots (b), (d) and (f) show top-k (precision@k) standard precision for DNN, CNN and GRU neural networks with weighted cross-entropy loss function, respectively.

Results

Three different neural network architectures - dense neural network (DNN), convolutional neural network (CNN) and gated recurrent units neural network (GRU) - are compared on their performances in predicting tools (Figures 3 and 4). The models obtained after training all the neural network architectures are used to predict tools for the tool sequences in the test data after every training iteration. Top-k precision (precision@k) is a popular metric for evaluating a recommender system [28, 29, 30]. Precision@k implies how many in the k predicted tools are correct. The correctness here refers to the compatibility of the predicted tools with the tool for which predictions have been made. For example, $k = 2$ implies that the number of predicted tools are 2 with the highest predicted scores. If only 1 of them is correct, then the precision@2 is $\frac{1}{2} = 0.5$. In this way, precision@1 and precision@2 are computed for all the tool sequences in the test data and then averaged. Precision@1 and precision@2 metrics are used in this approach to evaluate the quality of the tool recommender system. Precision of recommended tools are computed separately for the standard and normal recommendations and shown in different plots (Figures 3 and 4) but for usage frequencies, they are combined into one plot (Figure 5). The precision and usage frequencies of the predicted tools for the precision@1 and precision@2 (top-1 and top-2) metrics are computed over 10 training iterations for each experiment run. They are averaged and their respective standard deviations are computed over 10 experiment runs. Mean precision and usage frequencies are shown by the respective line plots and the shaded regions span the area between one standard deviation above and below the mean (Figures 3, 4 and 5).

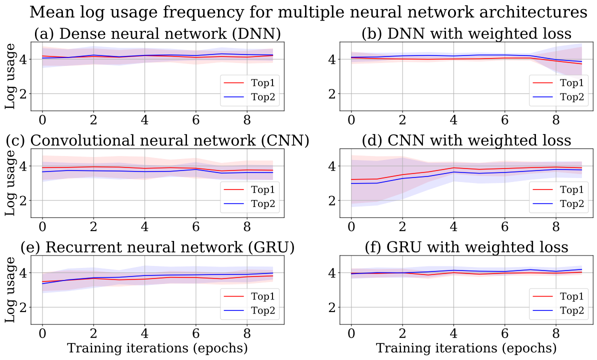

.

Figure 5. The subplots (a), (c) and (e) show usage frequencies of (top-k) predicted tools for DNN, CNN and GRU neural networks with cross-entropy loss function, respectively. The subplots (b), (d) and (f) show usage frequencies of (top-k) predicted tools for DNN, CNN and GRU neural networks with weighted cross-entropy loss function, respectively.

Comparison of GRU neural network with other approaches

The GRU neural network with the weighted cross-entropy loss function shows superior performance to CNN (Figures 3d, 3f, 4d and 4f) by achieving 98% top-1 standard and normal precision which proves that the GRU layers in a neural network are better for learning on tool sequences than the convolutional layer. Moreover, it shows a lower divergence in standard and normal precision and usage frequencies (Figures 3d, 3f, 4d, 4f, 5d and 5f) establishing that its predictive strength is more stable than CNN over multiple experiment runs. Surprisingly, the weighted cross-entropy loss function does not have any beneficial effect on the CNN architecture as its standard and normal precision and usage frequencies show higher divergence over multiple experiment runs (Figures 3c, 3d, 4c, 4d, 5c and 5d). Therefore, CNN is not used in our approach. In contrast to CNN, DNN achieves a similar standard and normal precision to the GRU neural network with a small divergence (Figures 3a, 3b, 3f, 4a, 4b and 4f). However, due to higher divergence in accumulated usage frequencies, it is not used in our approach (Figures 5a, 5b and 5f). Weighted cross-entropy loss function in the GRU neural network (Figure 5f) drives it to classify tools more robustly with higher usage frequencies. In other words, it predicts tools with higher precision than CNN and lower divergence in usage frequencies than DNN. Therefore, it is used in our approach to learn on tool sequences and recommend tools.

To compare the performance of the GRU neural network

with approaches which do not use any neural network, two ideas are explored. First, using a simple approach to store all the indices of sequences of tools [31] and second, using Extra-Trees classifier [32]. These approaches are discussed in section S1 of the supplementary document in detail.

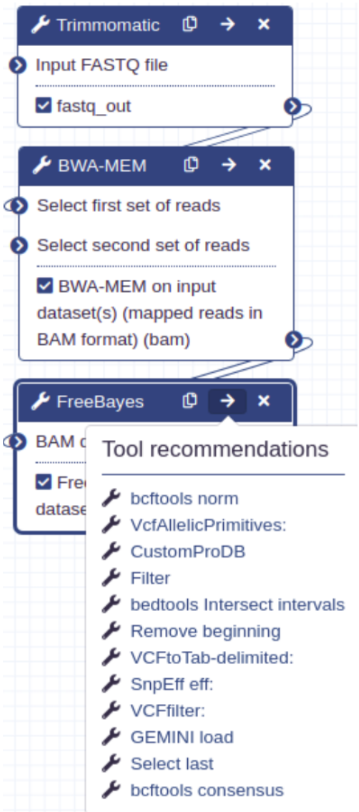

Figure 6. The figure shows recommended tools, listed in a dropdown with "Tool recommendations" as its header, in the workflow editor of the European Galaxy server for "Trimmomatic → BWA-MEM → FreeBayes" tool sequence. The recommended tools for the tool sequence can be seen in a dropdown while hovering on the right arrow button placed in the top-right corner of the "FreeBayes" tool. Clicking on any recommended tool such as "bcftools norm" in the dropdown opens a new block for the chosen tool which can be connected to the tool sequence.

Benefit of regularisation

Using regularisation minimises overfitting by assisting the GRU neural network to make better recommendations by predicting tools having low usage frequencies but useful in addition to tools with high usage frequencies. For example, the recommendations of "UMI-tools count" [33] tool with the regularised model include "Seurat" [34] tool which is absent from recommendations by the non-regularised model. Another example is for "RaceID, Lineage computation using StemID" tool sequence which gets "Lineage Branch Analysis using StemID" [35] tool as one of the recommendations by the regularised model while there is no recommendation at all by the non-regularised model. The recommendations for a popular mapper, RNA-STAR [36], are "featureCounts" [37], "MultiQC" [38], "Infer Experiment" [39] and few others by both the models. But, in addition to these recommendations, the regularised model recommends the "Read Distribution" [39] tool which is not predicted by the non-regularised model. More details have been provided in section S4 of the supplementary document.

Examples of tool recommendations

To illustrate the real time usage of the recommender system in the European Galaxy server, two examples have been provided. The first one shows recommended tools for a tool sequence with 3 tools, Trimmomatic [40] → BWA-MEM [41] → FreeBayes [42], in the workflow editor of the European Galaxy server (Figure 6). Trimmomatic is used to trim the sequencing data such as DNA and RNA sequences. One of the useful analyses after trimming the sequences is to map them on a reference genome using a mapper. Several mappers such as BWA-MEM [41], Bowtie2 [43] and RNA-STAR [36] are predicted. BWA-MEM is chosen from the predicted mappers and connected to Trimmomatic. After mapping, for further analysis of mapped sequences, many tools are predicted such as MultiQC for summarising the quality of mapping, featureCounts for counting the reads mapped to different regions on the genome or FreeBayes for detecting variants and a few others. FreeBayes is chosen and a list of recommendations is shown as a dropdown containing tools such as "bcftools norm" [44], VcfAllelicPrimitives [45] and many others for Trimmomatic → BWA-MEM → FreeBayes tool sequence. Another example of tool recommendations after using RNA-STAR is shown in Figure 7. It shows follow-up tools such as bamCoverage [46] for calculating read coverage, MultiQC, featureCounts and a few others. In summary, the tool recommendations provide useful knowledge about tools to Galaxy users and researchers to continue multiple scientific analyses.

Table 1 lists a few standard and normal recommended tools for multiple tool sequences in different scientific analyses such as Computational chemistry, Epigenetics, Machine learning, Proteomics, RNA sequencing and a few others. The recommended tools shown in this table are highly used for standard scientific analyses as highlighted in multiple GTN training materials [60].

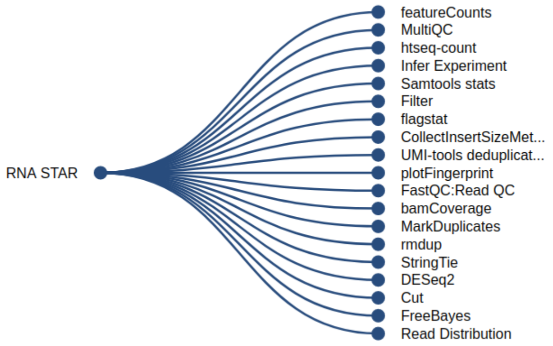

Figure 7. The figure shows recommended tools as leaves (on the right) of the tree after executing RNA-STAR tool. Clicking on any recommended tool opens its definition in Galaxy and can be used for further analysis with the data files produced by the previous tool (RNA-STAR).

Implementation

To create a tool recommender system in Galaxy, workflows are collected from the European Galaxy server. A workflow may have one or many tool sequences where tools are connected one after another. Tool sequences are transformed into matrices and produced as input to a GRU neural network to learn patterns in the connections of tools.

$$p(x_T | x_1, x_2, ..., x_{T-1}) \quad (1)$$

Table 1. Standard and normal recommended tools for tool sequences in different scientific analyses

Scientific analyses	Tool/Tool sequences	Recommended tools (standard)	Recommended tools (normal)
Computational chemistry	Molecule to fingerprint [47]	Taylor-Butina clustering, NxN Clustering [48], Similarity Search	–
Epigenetics	hicBuildMatrix [49]	hicSumMatrices	hicMergeMatrixBins, hicPlotMatrix [50], hicPCA, hicTransform
Epigenetics	multiBamSummary [46]	plotCorrelation [51]	plotPCA
Epigenetics	Bowtie2	MASC2 CallPeak [51]	bamCoverage, FreeBayes
Machine learning	Create a deep learning model architecture	–	Create deep learning model [52], Build Deep learning Batch Training Models
Proteomics	Msconvert [53]	Search GUI, FlashLFQ	PeakPickerHiRes [54]
RNA Sequencing	Cutadapt [55]	FastQC, RNA-STAR, MultiQC [56]	Bowtie2, Hisat2, BWA-MEM
RNA Sequencing	Cutadapt [55], RNA-STAR	featureCounts, MultiQC, Infer Experiment [56]	bamCoverage, RmDup
Single-cell	UMI-tools extract [33]	RNA-STAR [57]	Bowtie2, Hisat2, BWA-MEM, UMI-tools group
Single-cell	Initial processing using RaceID [35]	Clustering using RaceID [58]	–
Single-cell	Initial processing using RaceID, Clustering using RaceID [35]	Cluster Inspection using RaceID [58], Lineage computation using StemID	–
Variant-calling	FreeBayes	VcfAllelicPrimitives [59]	Gemini load
Variant-calling	FreeBayes,VcfAllelicPrimitives	SnpSift Filter, VT normalize	SnpEff eff [59]

The probability of a tool (x_T) is estimated given all other prior tools (x_1, ..., x_{T-1}) for a tool sequence (x_1, ..., x_{T-1}, x_T). Neural network learning is classification because there are labels for tool sequences which are learned and then predicted. Moreover, the classification is multi-class (multiple tools as labels) and multi-label (multiple tools as labels for a tool sequence) [61]. To ensure an unbiased learning and evaluation by the neural network, the set of tool sequences is divided into two parts - training and test. The training data is used for learning a model and the test data is used for evaluating the model.

Relevance of tools

To incorporate the usage frequency of tools in the recommender system, the usage frequencies of all the tools used in the last one year have been collected and are used in the neural network training as the weights (logarithm of usage frequencies) of tools. A tool which has been used often (for example Tool B in Figure 2) in the past one year is assigned a higher weight than a tool (for example Tool C in Figure 2) which has been used less often in the past one year. When tools are recommended a score is assigned to each tool by the neural network. It is expected that a tool with a higher weight gets a higher score and a tool with a lower weight gets a lower score. To summarise, the relevance of a tool to be used in a workflow decays if its usage drops over time in Galaxy. Alternatively, the relevance of tools can also be ascertained by counting the occurrence of each tool in all workflows and these occurrences can be used as their weights in the neural network training. But, it may happen that some tools which were used often in the past to create workflows are not used anymore. Therefore, assigning weights to these tools in the neural network training based on their occurrences in workflows may not be a good indicator of their relevance and overall, may not be optimal.

Uniform sampling

Workflows in Galaxy come from different scientific analyses. It may happen that the number of workflows from these analyses are not comparable - some analyses may have a large number of workflows while some may have only a small number of workflows. This can cause some tools to be present very frequently in workflows while other tools are less frequent. Learning on these workflows and recommending tools may exhibit bias by showing better recommendations for the frequently occurring tools and poorer recommendations for the less frequent ones. To showcase this imbalance, the frequencies of the last tool in each tool sequence in training data are calculated and it is found that only a few tools have large frequencies and most of the tools are present in low frequencies (Supplementary Figure 3). For example, the tools with very high frequencies (> 10,000) are “Concatenate datasets”, “Cut”, “Grouping” and “Join” while the tools having very low frequencies (< 5) are

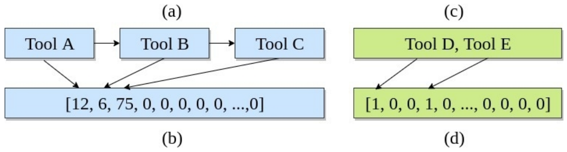

Figure 8. The figure shows how a tool sequence and its labels are transformed into vectors.

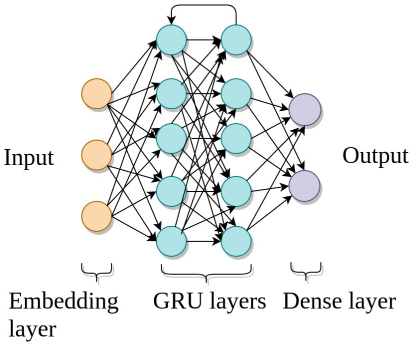

Figure 9. The figure shows the architecture of the GRU neural network. It has four components as layers. The first layer is the input layer (yellow), two stacked layers of GRU (cyan) and the last layer is the output layer (violet). The dropout layers are added between embedding and GRU layers, between two GRU layers and between the second GRU and dense layers.

"Cluster inspection using RaceID", "rDock cavity definition" [62] and "ChiRA collapse". Therefore, to overcome this drawback, the training data created after extracting tool sequences should be balanced in order to make the neural network learn on a similar number of tool sequences from different scientific analyses in each training iteration. To implement this strategy, a set of last tools in all tool sequences from the training data is collected. Further, for each tool in this set, a list of indices of tool sequences in the training data are stored for which it is the last tool (Supplementary Table 3). Only the last tools are considered for implementing this strategy because of two reasons. First, the smallest tool sequences contain only two tools and second, all tools become the last tool in at least one tool sequence and the computed frequencies of these last tools suggest their overall frequencies in the training data.

In the neural network training, for each iteration (which consumes all tool sequences in the training data), small batches containing equal number of tool sequences are created. For example if batch size is 100 and the size of training data is 2000, then 20 (2000/100=20) batches are created, each containing 100 tool sequences. In each batch, 100 tools from the set of last tools are uniformly selected (Column 2 in Supplementary Table 3) and for each selected tool, a tool sequence is chosen uniformly from its respective list of tool indices (Column 3 in Supplementary Table 3). After selecting tool sequences for many batches for each iteration of training (epoch), it is expected that all the tools from the set of last tools and their respective tool sequences are chosen. Performing this uniform selection of different tool sequences for each iteration, the training data becomes balanced. Supplementary Figure 4 shows that each last tool is present approximately 1670 times on an average in each iteration (epoch). The order of tools in the Supplementary Figures 3 and 4 are same.

Data transformation

Tool sequences extracted from workflows are transformed into vectors because neural networks require input data to be represented as vectors and matrices. Each tool sequence has one or more labels (Figure 1) and they are transformed into different vectors - a tool sequence vector (Figure 8b) and a label vector (Figure 8d). To form these vectors, a dictionary of tools is needed which stores an index for each tool. Using the indices of tools, a tool sequence vector is created preserving the original order of tools as in the tool sequence. For example, Tool A has an index of "12" in the dictionary, therefore it is replaced by "12" in the vector (Figure 8b). The vector is padded with trailing zeros to keep the length of the vector same across the varying lengths of tool sequences. The size of this vector is 25 which means that a tool sequence can have a maximum of 25 tools. The tool sequences larger than this size are discarded. The labels (Figure 8c) are transformed into a bit vector (Figure 8d) in which the positions, stored as indices in the dictionary of tools, of the labels (tools) are turned "on" (set to 1) specifying that these tools are the labels of the tool sequence and others are not (set to 0). It has the same size as the dictionary of tools. In the machine learning field, it is also known as a multi hot-encoded vector. Together, these two vectors form a training sample for the neural network. A pair of vectors are created in this manner for each tool sequence and for all the tool sequences, they are combined to form two matrices - one for tool sequences and another for their respective labels. Internally, the label vectors have two subsets, one for standard labels (from the published, non-deleted and non-erroneous workflows) and another for normal labels (from the rest of the workflows). These matrices form input data to the neural network.

Neural network architecture

GRU neural network, a variant of RNN, is used for creating a model to recommend tools. The neural network architecture has multiple components such as different layers (Figure 9), activation functions, class weights, loss function, hyperparameter tuning technique which are discussed in detail in the following paragraphs.

Embedding layer. The first component of the neural network architecture is an input layer (Figure 9) which learns an embedding, a fixed-size vector, for each tool. This vector is used by the neural network as an internal representation of a tool. The embedding vector replaces the indices of tools in each tool sequence. The size of the embedding vector is fixed for all tools. For example, the vector of a tool sequence [12, 6, 75, 0, 0, ..., 0] is transformed into [[0.3, 0.01, 0.003, ..., 0.23], [0.5, 0.1, 0.005, ..., 0.9], [...], 0, 0, ...,0] by the embedding layer. The same embedding vector represents a tool in all tool sequences in which the tool is present.

GRU layer. The stacked layers of GRU learn deeper structures in the tool sequences by modelling the conditional probabilities of tools (labels) given all other prior tools (Figure 9). GRU has certain advantages which helps it to learn on sequential data. First, it avoids the problems of vanishing and exploding gradients which commonly occur in traditional RNN [63]. It is important because learning higher-order dependencies depends on the gradients of errors concerning the parameters (recurrent and input weight matrices) of GRU layers. Second, GRU has slightly fewer parameters than the long short-term memory network (LSTM), another variant of RNN, which makes using GRU simpler than LSTM. Finally, it achieves similar accuracy as the LSTM [19].

Output layer. The last component of the neural network architecture is a dense layer which computes predictions (Figure 9). The dimension of this layer is equal to the number of unique tools because it predicts a score for each tool (label). The predicted score of each tool is considered as its probability of being

the label of an input tool sequence. The closer the predicted score of a tool is to 1, the more probable it is to be the recommended tool and the closer it is to 0, the less probable it is to be the recommended tool.

Dropout layer. Overfitting happens when a neural network performs exceptionally well on the training data but its performance on test (unseen) data remains poor. To minimise the effect of overfitting, a dropout layer is used between two layers of the neural network. It sets a few randomly chosen connections to 0 in the neural network to introduce some randomness to minimise overfitting [64, 65]. 3 dropout layers are used in our approach - one between the embedding and the first GRU layers, one between 2 GRU layers and the last one between the second GRU and dense layers.

Activations. These are mathematical functions which are used in neural networks to transform inputs to a layer into its outputs. Two activations are used in our approach - one is exponential linear units (ELU) [66] and another is sigmoid (Equation 2). ELU is used for both the GRU layers and has a special feature of being negative when the input is negative which allows mean activation (output) to get closer to 0 compared to other activation functions such as ReLU [67] which is always positive. As mean activations get closer to 0, the approximated and actual gradients get closer to each other. Therefore, using ELU in our neural network as an activation can be useful to achieve faster training, an increased drop in loss and better accuracy. Sigmoid is used in the output layer which normalises any real number to lie between 0 and 1 and it is considered as a probability of each tool.

$$f(x) = \frac{1}{1 + e^{-x}} \tag{2}$$

Usage frequencies of tools as weights. To ensure that the relevance of tools decays with time if they have not been used regularly in the recent past, their usage frequencies are used as their respective weights in the neural network training. The usage frequencies of tools over the past year (Figure 2) have been collected from the European Galaxy server. A curve is fit through the usage frequencies of each tool using support vector regression (SVR) to display a trend of the usage of tool over time. Using this trend, the usage of the tool for the next month is predicted and its logarithm is used as the weight for this tool. The logarithm of usage frequencies is computed to normalise them because only a few tools have significantly large magnitude of usage compared to that of the remaining tools which may lead the neural network to learn and predict only tools with very large magnitude of usage and ignore other tools. Learning a trend for each tool involves 5-fold cross-validation and optimising two hyperparameters of SVR, kernel and degree, using grid search. The values used for the kernel are - "rbf", "poly" and "linear" and the values of degree used are 2 and 3. By following the grid search, there are 3 (kernels) x 2 (degrees) = 6 different combinations of hyperparameters to be verified to find the best curve for each tool [68].

Loss function. A neural network learns patterns from data by minimising a loss function. Cross-entropy is a popular choice for a loss function in classification problems [69]. In our approach, cross-entropy function is used in the GRU neural network to compute the loss between the true and predicted label and is weighted by the label's weight. The loss is summed up over all labels of a tool sequence and then averaged (Equation 3). The term T is the total number of labels (size of the label bit vector). The term w_i is the weight of the ith label. The terms p^a and p^b refer to the true and predicted label vectors for a tool sequence, respectively. In general, the loss is large when p^a and p^b are far away from each other which means that the learning by the neural network is not good. If they are close, the loss is low and the predictions are better. When an unweighted cross-entropy is used as the loss function for any classification problem [70] then it is assumed that all the predictions have the same weight and it does not differentiate between the more and less dominant labels. In our approach when it is used as a loss function in the neural network, then even though the predicted labels are correct they may not necessarily have large weights and thereby maybe less relevant. Therefore, to reduce the possibility of less relevant labels appearing in recommendations, loss is weighted by the weights of labels. It means that if a label with a larger weight is misclassified, which means that the true and predicted values are different, then the overall loss is higher. In this way, the wrong classification of a label with a larger weight is penalised more than the wrong classification of a label with a smaller weight.

$$loss = -\frac{1}{T}\sum_{i=1}^{T}(p_i^a \cdot \log(p_i^b) + (1 - p_i^a) \cdot \log(1 - p_i^b)) * w_i \tag{3}$$

The loss in Equation 3 is computed for all tool sequences in training data and is minimised using a root mean square propagation (RMSProp) optimiser. It follows an adaptive approach to estimate the learning rate by keeping knowledge of gradients in prior iterations. The learning rate is updated by dividing it with an average of the square of the prior gradients [71].

Hyperparameter tuning. A neural network has multiple hyperparameters. In our approach, they are the number of dimensions of the embedding layer, learning and dropout rates, number of units for the GRU layer and size of batches. They should be optimised to find the best configuration (a combination of hyperparameters) for training on tool sequences as a different configuration may give a different performance on the same training data. The grid and random searches are popular techniques to optimise hyperparameters. One limitation of these approaches is that they evaluate each configuration independently and have a high time complexity to find the best configuration. Therefore, the hyperparameters in our approach are optimised using a Bayesian (sequential model-based) optimisation [72]. It learns from the previously evaluated configurations which ensures faster convergence. Reasonable ranges of all the hyperparameters to be optimised are given and the best configuration is found after 20 evaluations.

Learning and predictions

Over 229,000 tool sequences collected from more than 18,000 workflows are divided into training and test data. A neural network learns patterns in the tool sequences from the training data and creates a model. The ability of the model to recommend tools is evaluated on the test data which is unseen by the neural network during training. The training data forms 80% (approximately 185,000) of all tool sequences and it is iterated over 10 epochs of neural network training. The remaining 20% (approximately 45,000) is used as the test data. The running time of the training is approximately 50 hours on a high performance compute cluster provided by bwCloud [73] with multiple cores. After learning on the training data, the model is used to predict tools. Each predicted tool gets a probability score of being the recommended tool of a tool or tool sequence. Two sets of predictions are made - standard and normal. Each set is sorted in the descending order of their probabilities and the top ones in both sets are combined to show them as recommen-

dations.

Summary and future work

A system to recommend tools in Galaxy is built by analysing workflows using a variant of RNN (GRU) and a weighted cross-entropy loss function. The recommended tools are relevant for multiple scientific analyses with high accuracy as shown by the high similarities between the tools used in GTN tutorials and the recommended tools for similar analyses (Table 1). Moreover, they are easily accessible through simple UI integrations in Galaxy (Figures 6 and 7). Collectively, they improve user experience by helping researchers to easily create correct workflows. In addition, the approach does not store any information about tools and the recommendations are made by learning only the patterns of tool connections in workflows. The model [74] created using this approach and an API [75] are integrated into the European Galaxy server. The API resides with other Galaxy APIs and accesses a tool or a tool sequence specified by researchers to show its recommendations in real time using the model. The API is used at two different user interfaces in Galaxy - one shows recommendations in the workflow editor (Figure 6) and another shows them after each tool execution (Figure 7). The recommendation system should be potentially helpful for those researchers who are new to the Galaxy platform. It shows them a few follow-up tools from a big collection of more than 3,000 tools and enables them to perform multiple exploratory data analyses.

Different Galaxy servers maintain different sets of tools and workflows. The current approach can be used to create different recommendation models for different Galaxy servers. Alternatively, all the workflows can be collected from multiple Galaxy servers and using the current approach, one recommendation model can be created by learning on the complete set of workflows and the model can be distributed to different Galaxy servers. To improve the quality of recommendations, the annotations of tools can be incorporated in the learning mechanism by assigning higher weights to the annotated tools in comparison to tools which are not annotated. Tools containing similar annotations may have similar functionalities and using these similarities, tool recommendations can be further enhanced by showing similar tools for each recommended tool. In addition to learning tool connections to recommend tools, the knowledge of tools connecting to different tools based on their respective parameters can also be incorporated.

Methods

Multiple neural network architectures

Multiple neural network architectures such as convolutional neural network (CNN) and dense neural network (DNN) with only dense layers, are used to compare their respective predictive strengths with the GRU neural network (Figures 3, 4 and 5). In these architectures too, the embedding layer is used as the first (input) layer and a dense layer is used as an output layer having the same dimensions as the number of tools. Additionally, in CNN, convolutional and max-pooling layers are used to learn spatial patterns in tool sequences and downsample the dimensionality of input, respectively. Moreover, two dense layers are also used and the last one serves as an output layer. DNN uses two dense layers as hidden layers. The cross-entropy, with and without weights, is used as the loss function and RMSProp is used as an optimiser. Bayesian optimisation is used to optimise the hyperparameters of these architectures. More details about the architectures of DNN and CNN are given in section S3 of the supplementary document.

Library, model and code repositories

The Keras deep learning library is used for producing the neural network architectures [76]. The trained model [74] is saved as an H5 file to simplify its distribution to different Galaxy instances. The file is an HDF5 store containing the weights of different layers of the neural network and their configurations, a dictionary of tools and their indices and the weights of tools. The weights and configuration of the neural network are needed to recreate the trained model. The dictionary is used to replace IDs of the predicted tools by their indices in a tool sequence. All data and python scripts used in our approach are stored at Github for all approaches - GRU [77], CNN [78] and DNN [79]. In each of these repositories, the process to create a tool recommendation model is explained. All these repositories are provided with a script ("extract_data.sh") for collecting raw input datasets from a Galaxy instance. These datasets are workflows and usage frequencies of tools and are also provided in each repository. The values of multiple hyperparameters of neural networks, number of training iterations and sizes of training and test data can be altered using a bash script ("train.sh"). To execute the scripts on a GPU enabled machine, the "tensorflow-gpu" package should be installed instead of "tensorflow" as mentioned in the conda package dependencies file ("environment.yml"). To see recommended tools, an ipython script ("tool_recommendation_gru_wc.ipynb" for GRU repository) is also provided which loads and recreates a trained model to predict tools for a tool or a tool sequence. The result files storing precision, training and validation losses and usage frequencies, which are used for generating line plots (Figures 3, 4 and 5), for all approaches are also available at Github [80]. The code repositories of two other approaches which do not use neural networks are available at - simple approach [31] and ExtraTrees [32].

New recommendation model

On a usual Galaxy server, tools and workflows are dynamic as they are added and updated regularly. Therefore, it is important to train the GRU neural network on the complete set of workflows periodically to keep the tool recommendation model updated with the latest tools and workflows. Using a Galaxy tool [81], a new recommendation model can be created after collecting workflows and tool usage data from a Galaxy server. The tool runs for several hours (> 24 hours) and creates a model which is pushed to an online repository [74]. From this repository, Galaxy downloads it using an API [75] to recommend tools. The recommendation model is created periodically every 3-4 months to accommodate new workflows and tools. Galaxy admins can decide upon the frequency of creating a new model. It can be created every month or every 6 months

New tools as recommendations

Galaxy admins can overwrite the recommended tools predicted using the trained model by a different set of tools using the configuration option described in [82]. In addition, to highlight the newly added tools, which are not part of the model, they can be appended to the recommendations using this additional configuration option.

Availability of supporting source code and requirements

Project name: Tool recommender in Galaxy using deep learning
Project home page: https://github.com/anuprulez/galaxy_tool_recommendation
Operating system: Linux
Programming languages: Python, XML, JavaScript
Other requirements: Tensorflow, Keras, Scikit-learn, Numpy, H5py, Csvkit, Hyperopt
License: MIT License
RRID: SCR_018491
Biotools ID: tool_recommender_system_in_galaxy

Additional files

File name: Tool_recommendations_supplementary_file.pdf Title: Supplementary Material: Tool recommender system in Galaxy using deep learning Description: There 3 tables - Table 1 shows a comparison of recommendations between the GRU neural network, a simple model and ExtraTrees classifier, Table 2 shows a comparison of recommendations between the regularised and non-regularised GRU neural network and Table 3 shows the strategy of uniform sampling of training data. There are 7 figures - Figures 1 and 2 show architectures of convolutional neural network (CNN) and dense neural network (DNN) used in the paper, Figure 3 shows original frequencies of last tools in training data, Figure 4 shows the frequencies of last tools in training data after uniform sampling, Figures 5 and 6 show top-1 normal and standard precision respectively for less frequent tools and Figure 7 shows the top-1 and top-2 precision (normal and standard recommendations) of the ExtraTrees classifier.

Declarations

List of abbreviations

API: Application programming interface; CNN: Convolutional neural network; DNN: Dense neural network; ELU: Exponential linear units; GRU: Gated recurrent units; PROPHETS: Process realisation and optimisation platform using human-readable expression of temporal-logic synthesis; RNN: Recurrent neural network; SVR: Support vector regression; UI: User interface; WINGS: Workflow instance generation and specialization;

Ethics approval and consent to participate

Not applicable

Consent for publication

Not applicable

Competing interests

The authors declare that they have no competing interests.

Funding

This work was supported by the German Research Foundation (DFG) under Germany's Excellence Strategy (CIBSS - EXC-2189 - Project ID 390939984) and German Federal Ministry of Education and Research (BMBF grant 031A538A de.NBI).

Authors' contributions

First author (A.K.) implemented the project and wrote the manuscript. The second author (H.R.) wrote scripts for data collection, contributed to the manuscript and deployed the project on European Galaxy server. The third author (B.G.) provided the idea of the project, validated results, and contributed to the manuscript. The last author (R.B.) contributed to the manuscript. All authors approved the manuscript.

Acknowledgements

We thank Simon Bray and Joachim Wolff for proofreading the manuscript, Dr. Wolfgang Maier for providing feedback and Gianmauro Cuccuru for deploying it on the European Galaxy server.

References

1. Ewels P, Krueger F, Käller M, Andrews S. Cluster Flow: A user-friendly bioinformatics workflow tool. F1000Research 2017;5:2824, doi:10.12688/f1000research.10335.2.
2. Leipzig J. A review of bioinformatic pipeline frameworks. Brief Bioinform 2017;1;18(3):530–536, doi:10.1093/bib/bbw020.
3. Baichoo S, Souilmi Y, Panji S, Botha G, et al. Developing reproducible bioinformatics analysis workflows for heterogeneous computing environments to support African genomics. BMC Bioinformatics 2018;19, 457 (2018), doi:10.1186/s12859-018-2446-1.
4. Love MI, Huber W, Anders S. Moderated estimation of fold change and dispersion for RNA-seq data with DESeq2. Genome Biol 2014;550. doi:10.1186/s13059-014-0550-8.
5. Afgan E, Baker D, Batut B, et al. The Galaxy platform for accessible, reproducible and collaborative biomedical analyses: 2018 update. Nucleic Acids Research 2018;46(W1):W537–W544. doi:10.1093/nar/gky379.
6. Bela G, Beel J, Hentschel C. Scienstein : A Research Paper Recommender System. In: Proceedings of the International Conference on Emerging Trends in Computing; 2009. p. 309–315. http://www.sciplore.org/wp-content/papercite-data/pdf/gipp09.pdf.
7. Achakulvisut T, Acuna DE, Ruangrong T, Kording K. Science Concierge: A Fast Content-Based Recommendation System for Scientific Publications. PLoS ONE 2016;11(7): e0158423, doi:10.1371/journal.pone.0158423.
8. Wang D, Liang Y, Xu D, et al. A content-based recommender system for computer science publications. Knowledge-Based Systems 2018;157:1–9. doi:10.1016/j.knosys.2018.05.001.
9. SGomez-Uribe CA, Hunt N. The Netflix Recommender System: Algorithms, Business Value, and Innovation. ACM Transactions on Management Information Systems TMIS 2016;6(4).
10. Smith B, Linden G. Two Decades of Recommender Systems at Amazon.com. IEEE Internet Computing 2017;21(3):12–18. doi:10.1109/MIC.2017.72.
11. Palmblad M, L LA, Ison J, Schwämmle V. Automated workflow composition in mass spectrometry-based proteomics. Bioinformatics 2019;35(4):656–664. doi:10.1093/bioinformatics/bty646.
12. Naujokat S, Lamprecht AL, Steffen B. Loose Programming

with PROPHETS, Fundamental Approaches to Software Engineering, vol. 7212. J L, A Z, editors, Springer, Berlin, Heidelberg; 1996. Online ISBN 978-3-642-28872-2, doi: 10.1007/978-3-642-28872-2_7.
13. Gil Y, Ratnakar V, Kim J, et al. Wings Intelligent Workflow-Based Design of Computational Experiments. IEEE Intelligent Systems 2011;26(1):62–72. doi:10.1109/MIS.2010.9.
14. Srivastava A, Adusumilli R, Boyce H, et al. Semantic workflows for benchmark challenges: Enhancing comparability, reusability and reproducibility. PSB 2018;doi:10.1142/9789813279827_0019.
15. DiBernardo M, Pottinger R, Wilkinson M. Semi-automatic web service composition for the life sciences using the biomoby semantic web framework. Journal of Biomedical Informatics 2008;41(5):837–847. doi:10.1016/j.jbi.2008.02.005.
16. Michalski V, Memisevic R, Konda KR. Modeling sequential data using higher-order relational features and predictive training. CoRR 2014;abs/1402.2333. http://arxiv.org/abs/1402.2333.
17. Yin W, Kann K, Yu M, Schütze H. Comparative Study of CNN and RNN for Natural Language Processing. ArXiv 2017;abs/1702.01923. http://arxiv.org/abs/1702.01923.
18. Lipton ZC, Kale DC, Elkan C, Wetzel R. Learning to diagnose with LSTM recurrent neural networks. CoRR 2015;abs/1511.03677.
19. Chung J, Gulcehre C, Cho K, Bengio Y. Empirical evaluation of gated recurrent neural networks on sequence modeling. In: NIPS 2014 Workshop on Deep Learning, December 2014; 2014. .
20. Boulanger-Lewandowski N, Bengio Y, Vincent P. Modeling Temporal Dependencies in High-Dimensional Sequences: Application to Polyphonic Music Generation and Transcription. Proceedings of the 29th International Conference on Machine Learning, ICML 2012;2.
21. Karan S, Zola J. Exact structure learning of Bayesian networks by optimal path extension. IEEE International Conference on Big Data 2016;p. 48–55. doi:10.1109/BigData.2016.7840588.
22. Spirtes P, Glymour C, Scheines R, et al. Constructing Bayesian Network Models of Gene Expression Networks from Microarray Data, Research Showcase @ CMU. Semantic Scholar 2018;doi:10.1184/R1/6491291.v1.
23. Chickering DM. Learning Bayesian Networks is NP-Complete, vol. 112. D F, HJ L, editors, New York, NY: Springer; 1996. ISBN: 978-1-4612-2404-4, doi:10.1007/978-1-4612-2404-4_12.
24. Chickering DM, Heckerman D, Meek C. Large-Sample Learning of Bayesian Networks is NP-Hard. Journal of Machine Learning Research 2004;5:1287–1330.
25. Cooper GF. The computational complexity of probabilistic inference using bayesian belief networks. Artificial Intelligence 1990;42:393–405. doi:10.1016/0004-3702(90)90060-D.
26. European Galaxy Server. https://usegalaxy.eu/ (2020);. Accessed 22 July 2020.
27. Jian X, Wickramarathne TL, Chawla NV. Representing higher-order dependencies in networks. Science Advances 2016;2(5). doi:10.1126/sciadv.1600028.
28. Said A, Bellogín Kouki A, de Vries AP. A Top-N Recommender System Evaluation Protocol Inspired by Deployed Systems. In: In Proceedings of the 2013 ACM RecSys Workshop on Large-Scale Recommender Systems. LSRS; 2013. .
29. Kang Z, Peng C, Cheng Q. Top-N Recommender System via Matrix Completion. In: Proceedings of the Thirtieth AAAI Conference on Artificial Intelligence (AAAI-16); 2016. .
30. Deshpande M, Karypis G. Item-Based Top-N recommender Algorithms. ACM Transactions on Information Systems 2004;22(1):143–177. doi:10.1145/963770.963776.
31. Kumar A, Tool Recommender in Galaxy using stored tool sequences. 2020;. https://github.com/anuprulez/galaxy_tool_recommendation/tree/statistical_model. Accessed 22 July 2020.
32. Kumar A, Tool recommender system in Galaxy using extra trees classifier. 2020;. https://github.com/anuprulez/galaxy_tool_recommendation/tree/sklearn_rf. Accessed 22 July 2020.
33. Smith T, Heger A, Sudbery I. UMI-tools: modeling sequencing errors in Unique Molecular Identifiers to improve quantification accuracy. Genome Research 2017;27(3):491–499. doi:10.1101/gr.209601.116.
34. Butler A, Hoffman P, Smibert P, Papalexi E, Satija R. Integrating single-cell transcriptomic data across different conditions, technologies, and species. Nature Biotechnology 2018;36(5):411–420. doi:10.1038/nbt.4096.
35. D G, A L, L K, K W, O B, N S, et al. Single-cell messenger RNA sequencing reveals rare intestinal cell types. Nature 2015;525(7568):251–255. doi:10.1038/nature14966.
36. A D, CA D, F S, et al. STAR: ultrafast universal RNA-seq aligner. Bioinformatics 2013;29(1):15–21. doi:10.1093/bioinformatics/bts635.
37. Liao Y, Smyth GK, Shi W. featureCounts: an efficient general purpose program for assigning sequence reads to genomic features. Bioinformatics 2013;30 (7):923–930. doi:10.1093/bioinformatics/btt656.
38. P E, M M, S L, M K. MultiQC: summarize analysis results for multiple tools and samples in a single report. Bioinformatics 2016;32(19):3047–3048. doi:10.1093/bioinformatics/btw354.
39. Wang L, Wang S, Li W. RSeQC: quality control of RNA-seq experiments. Bioinformatics 2012;28(16):2184–2185. doi:10.1093/bioinformatics/bts356.
40. Bolger AM, Lohse M, Usadel B. Trimmomatic: a flexible trimmer for Illumina sequence data. Bioinformatics 2014;30:2114–2120.
41. Li H. Aligning sequence reads, clone sequences and assembly contigs with BWA-MEM. ArXiv 2013 03;1303.
42. Garrison E, Marth G, Haplotype-based variant detection from short-read sequencing; 2012. https://arxiv.org/abs/1207.3907.
43. Langmead B, Trapnell C, Pop M, et al. Ultrafast and memory-efficient alignment of short DNA sequences to the human genome. Genome Biol 2009;10. https://doi.org/10.1186/gb-2009-10-3-r25.
44. Li H, Handsaker B, Wysoker A, Fennell T, Ruan J, Homer N, et al. The Sequence Alignment/Map format and SAMtools. Bioinformatics 2009;25 (16):2078–2079. doi:10.1093/bioinformatics/btp352.
45. Garrison E, A C++ library for parsing and manipulating VCF files.;. https://github.com/vcflib/vcflib. 2015. Accessed 02 August 2020.
46. Ramirez F, Ryan DP, Gruening B, Bhardwaj V, Kilpert F, Richter AS, et al. deepTools2: a next generation web server for deep-sequencing data analysis. Nucleic Acids Research 2016;44 (W1:W160–W165. doi:10.1093/nar/gkw257.
47. O'Boyle NM, Banck M, James CA, Morley C, Vandermeersch T, Hutchison GR. Open Babel: An open chemical toolbox. Journal of Cheminformatics 2011;3 (1). doi:10.1186/1758-2946-3-33.
48. Bray S, Protein-ligand docking (Galaxy Training Materials); 2020. https://training.galaxyproject.org/training-material/topics/computational-chemistry/tutorials/cheminformatics/tutorial.html, [Online; accessed 22 July 2020].
49. Ramirez F, Wolff J, Gruening B, Bhardwaj V, Ryan D,

Duendar F, Deeptools/Hicexplorer: Winter Release;. https://zenodo.org/record/1133705. 2017. Accessed 02 August 2020.
50. Wolff J, Ramirez F, Bhardwaj V, Polkh E, Hi-C analysis of Drosophila melanogaster cells using HiCExplorer (Galaxy Training Materials); 2020. https://training.galaxyproject.org/training-material/topics/epigenetics/tutorials/hicexplorer/tutorial.html, [Online; accessed 22 July 2020].
51. Dündar F, Erxleben A, Batut B, Bhardwaj V, Ramirez F, Rabbani L, Formation of the Super-Structures on the Inactive X (Galaxy Training Materials); 2019. https://training.galaxyproject.org/training-material/topics/epigenetics/tutorials/formation_of_super-structures_on_xi/tutorial.html, [Online; accessed 22 July 2020].
52. Kumar A, Khanteymoori A, Introduction to deep learning (Galaxy Training Materials); 2020. https://training.galaxyproject.org/training-material/topics/statistics/tutorials/intro_deep_learning/tutorial.html, [Online; accessed 22 July 2020].
53. Kessner D, Chambers M, Burke R, Agus D, Mallick P. ProteoWizard: open source software for rapid proteomics tools development. Bioinformatics 2008;24 (21):2534–2536. doi:10.1093/bioinformatics/btn323.
54. Sigloch FC, Grüning B, Peptide and Protein ID using OpenMS tools (Galaxy Training Materials); 2020. https://training.galaxyproject.org/training-material/topics/proteomics/tutorials/protein-id-oms/tutorial.html, [Online; accessed 22 July 2020].
55. Marcel M. Cutadapt removes adapter sequences from high-throughput sequencing reads. EMBnetjournal 2011;17 (1). doi:10.14806/ej.17.1.200.
56. Batut B, Freeberg M, Heydarian M, Erxleben A, Videm P, Blank C, et al., Reference-based RNA-Seq data analysis (Galaxy Training Materials); 2020. https://training.galaxyproject.org/training-material/topics/transcriptomics/tutorials/ref-based/tutorial.html, [Online; accessed 22 July 2020].
57. Tekman M, Batut B, Erxleben A, Maier W, Pre-processing of Single-Cell RNA Data (Galaxy Training Materials); 2020. https://training.galaxyproject.org/training-material/topics/transcriptomics/tutorials/scrna-preprocessing/tutorial.html, [Online; accessed 22 July 2020].
58. Tekman M, Ostrovsky A, Downstream Single-cell RNA analysis with RaceID (Galaxy Training Materials); 2020. https://training.galaxyproject.org/training-material/topics/transcriptomics/tutorials/scrna-raceid/tutorial.html, [Online; accessed 22 July 2020].
59. Nekrutenko A, Soranzo N, Calling variants in diploid systems (Galaxy Training Materials); 2019. https://training.galaxyproject.org/training-material/topics/variant-analysis/tutorials/dip/tutorial.html, [Online; accessed 22 July 2020].
60. Batut B, et al. Community-Driven Data Analysis Training for Biology. Cell Systems 2018;6:752–758.E1. https://training.galaxyproject.org/, doi:10.1016/j.cels.2018.05.012.
61. Tsoumakas G, Katakis I. Multi-label classification: An overview. International Journal of Data Warehousing and Mining 2009;3:1–13. doi:10.4018/jdwm.2007070101.
62. Ruiz-Carmona S, Alvarez-Garcia D, Foloppe N, Garmendia-Doval AB, Juhos S, Schmidtke P, et al. rDock: A Fast, Versatile and Open Source Program for Docking Ligands to Proteins and Nucleic Acids. PLoS Computational Biology 2014;10 (4). doi:10.1371/journal.pcbi.1003571.
63. Pascanu R, Mikolov T, Bengio Y. Understanding the exploding gradient problem. ArXiv 2012;abs/1211.5063.
64. Zaremba W, Sutskever I, Vinyals O. Recurrent Neural Network Regularization. ArXiv 2014;abs/1409.2329.
65. Gal Y, Ghahramani Z. A Theoretically Grounded Application of Dropout in Recurrent Neural Networks. In: Proceedings of the 30th International Conference on Neural Information Processing Systems; 2016. p. 1027–1035.
66. Clevert D, Unterthiner T, Hochreiter S. Fast and Accurate Deep Network Learning by Exponential Linear Units (ELUs). CoRR 2015;abs/1511.07289.
67. Nair V, Hinton GE. Rectified Linear Units Improve Restricted Boltzmann Machines. In: ICML'10: Proceedings of the 27th International Conference on International Conference on Machine Learning, June 2010; 2010. p. 807–814.
68. Pedregosa F, Varoquaux G, Gramfort A, et al. Scikit-learn: Machine Learning in Python. Journal of Machine Learning Research 2011;12:2825–2830. doi:10.5555/1953048.2078195.
69. Janocha K, Czarnecki W. On Loss Functions for Deep Neural Networks in Classification. ArXiv 2017;abs/1702.05659. doi:10.4467/20838476SI.16.004.6185.
70. Sadowski P, Notes on Backpropagation. https://www.ics.uci.edu/~pjsadows/notes.pdf (2016). Accessed 22 July 2020;.
71. Ruder S. An overview of gradient descent optimization algorithms. ArXiv 2016;abs/1609.04747.
72. Bergstra J, Yamins D, Cox DD. Hyperopt: A Python Library for Optimizing the Hyperparameters of Machine Learning Algorithms. 12th Python in science conf (SCIPY 2013) 2013;.
73. BwCluster. https://portal.bw-cloud.org/project/ (2020);. Accessed 22 July 2020.
74. Kumar A, Tool Recommender model;. https://github.com/galaxyproject/galaxy-test-data/blob/master/tool_recommendation_model.hdf5. 2020. Accessed 29 July 2020.
75. Kumar A, Get tool predictions. 2020;. https://github.com/usegalaxy-eu/galaxy/blob/release_20.05_europe/lib/galaxy/webapps/galaxy/api/workflows.py#L638. Accessed 22 July 2020.
76. Chollet F, et al. Keras 2015;.
77. Kumar A, Tool Recommender in Galaxy using GRU neural network. 2020;. https://github.com/anuprulez/galaxy_tool_recommendation. Accessed 22 July 2020.
78. Kumar A, Tool Recommender in Galaxy using CNN neural network. 2020;. https://github.com/anuprulez/galaxy_tool_recommendation/tree/cnn_wc. Accessed 22 July 2020.
79. Kumar A, Tool Recommender in Galaxy using DNN neural network. 2020;. https://github.com/anuprulez/galaxy_tool_recommendation/tree/dnn_wc. Accessed 22 July 2020.
80. Kumar A, Output results files. 2020;. https://github.com/anuprulez/galaxy_tool_recommendation/tree/master/output_files/data_20_05. Accessed 22 July 2020.
81. Kumar A, Tool Recommender model creator;. https://usegalaxy.eu/root?tool_id=toolshed.g2.bx.psu.edu/repos/bgruening/create_tool_recommendation_model/create_tool_recommendation_model/0.0.3. 2020. Accessed 22 July 2020.
82. Kumar A, Tool Recommender overwrite;. https://github.com/usegalaxy-eu/galaxy/blob/release_20.05_europe/config/tool_recommendations_overwrite.yml.sample. 2020. Accessed 30 July 2020.

Supplementary figure 1
Click here to access/download;Figure;supplementary_fi

Architecture of Convolutional neural network (CNN)

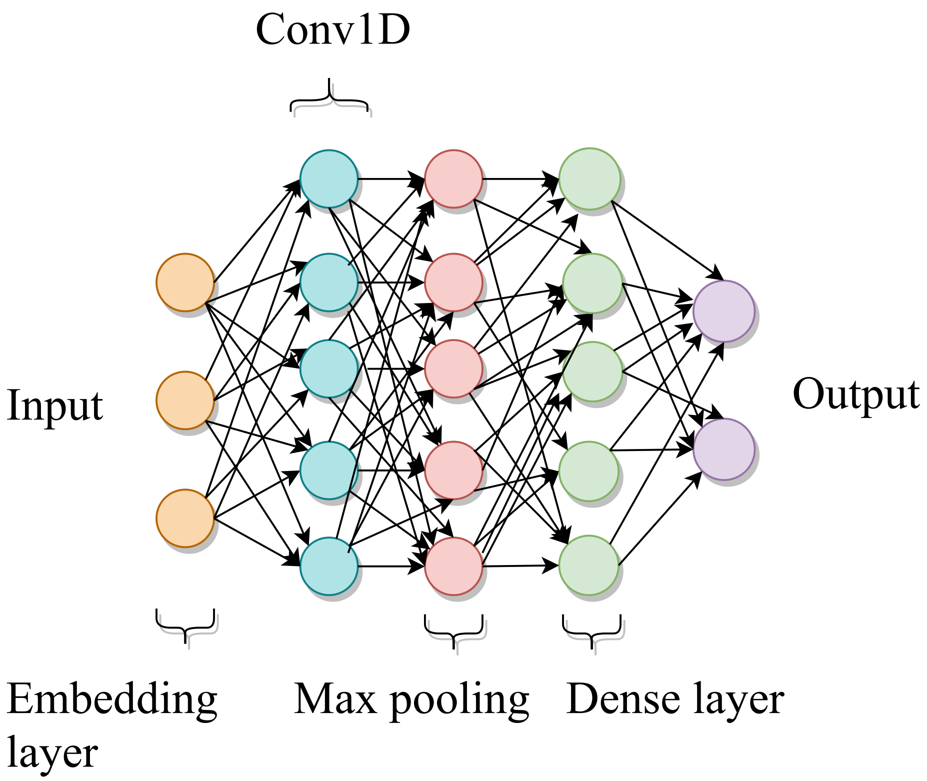

Supplementary figure 2

Click here to access/download;Figure;suppleme

Architecture of Dense neural network (DNN)

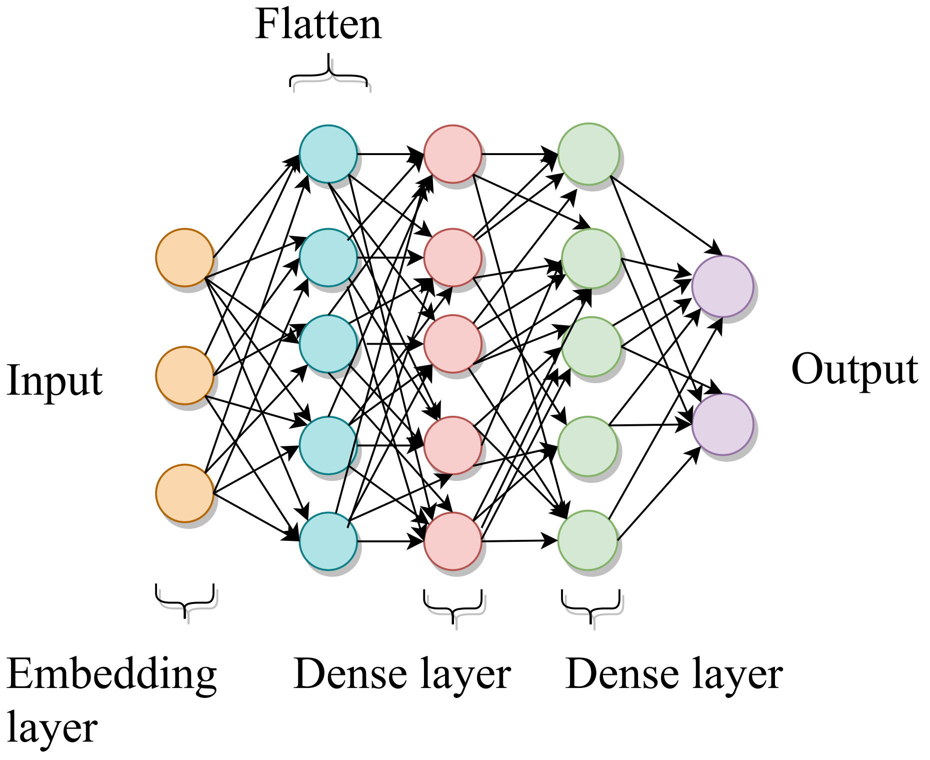

Click here to access/download;Figure;supplementary_figure_3.pdf

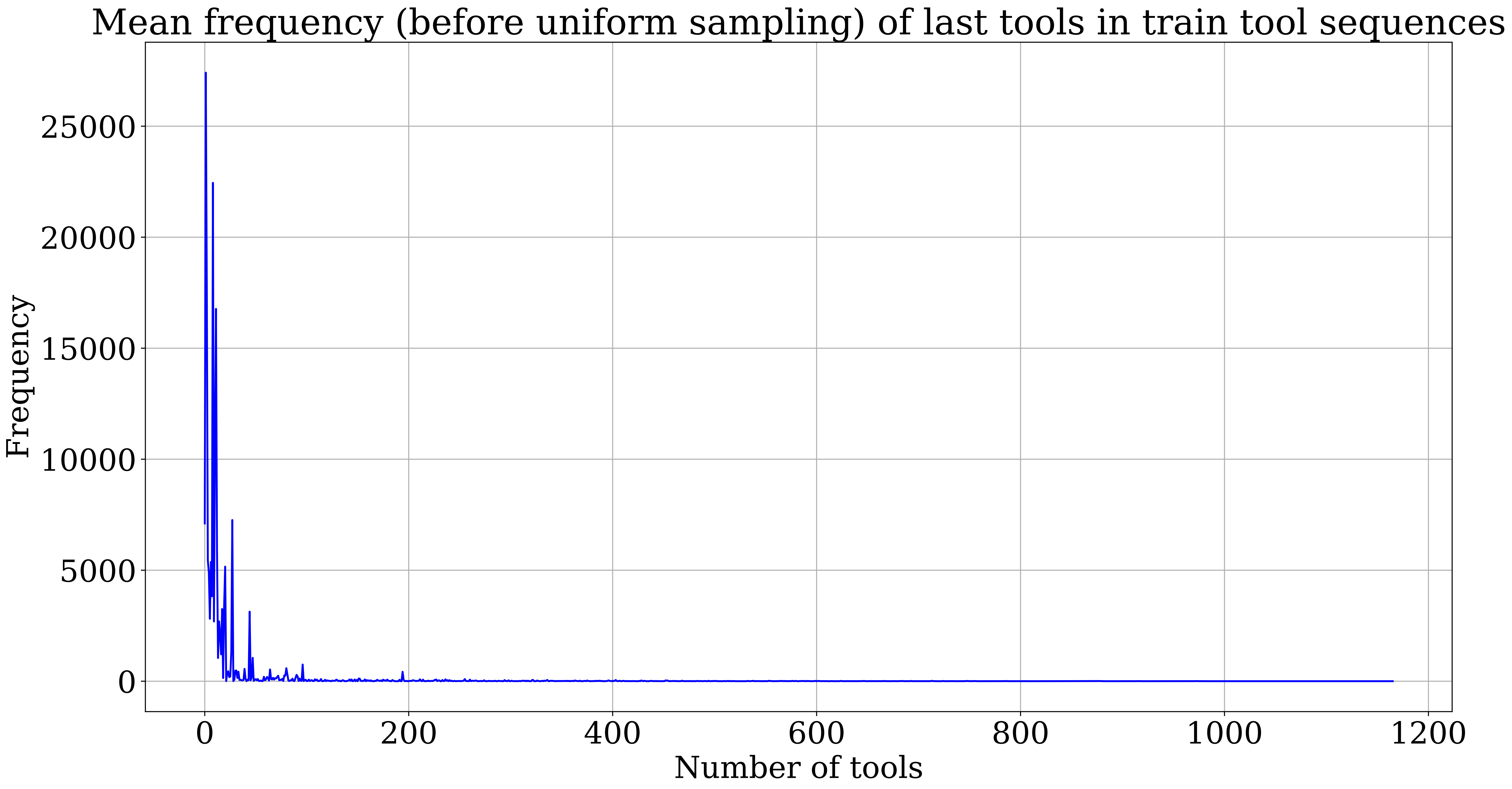

Click here to access/download;Figure;supplementary_figure_4.pdf

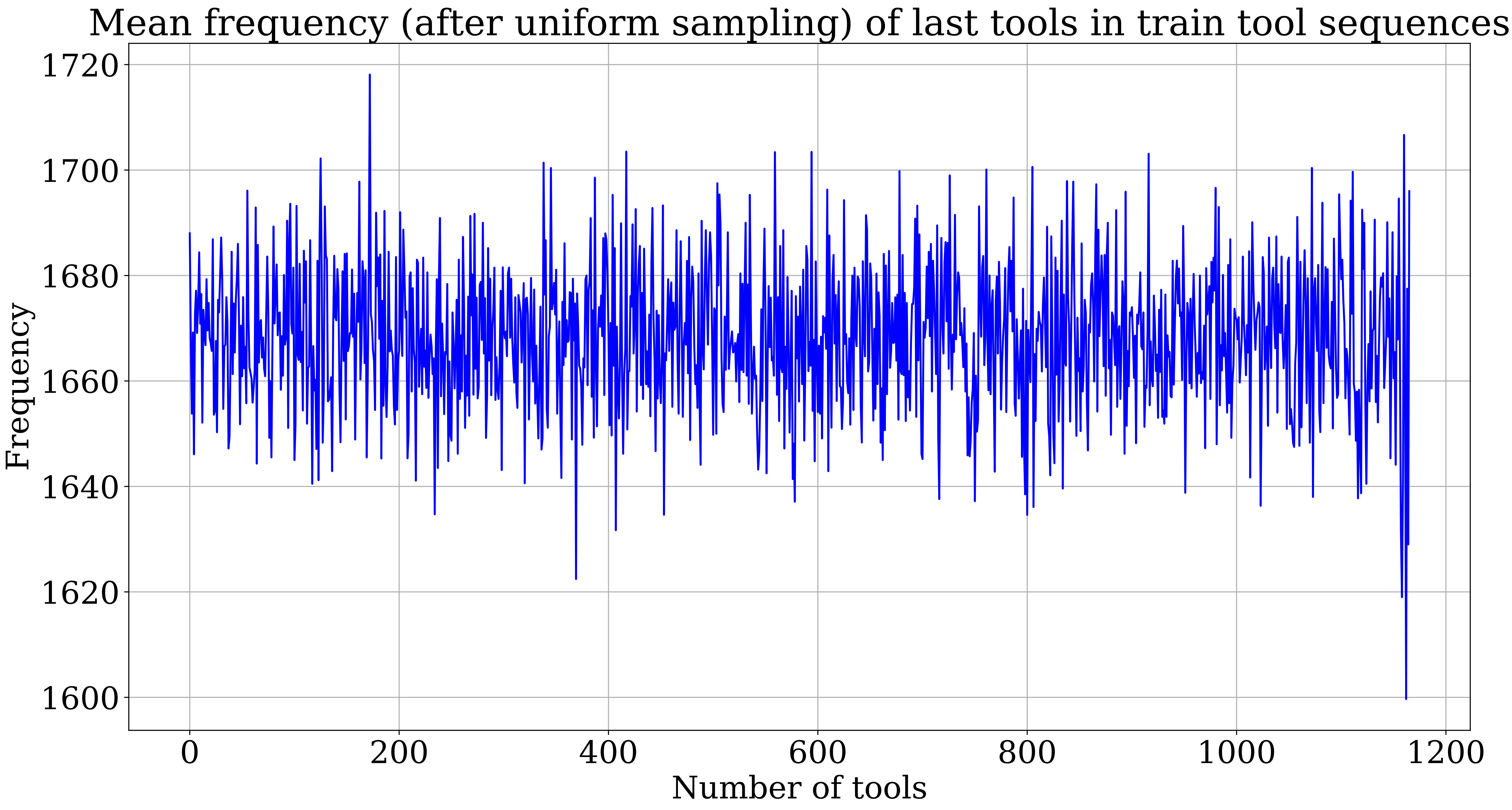

Click here to access/download;Figure;supplementary_figure_5.pdf

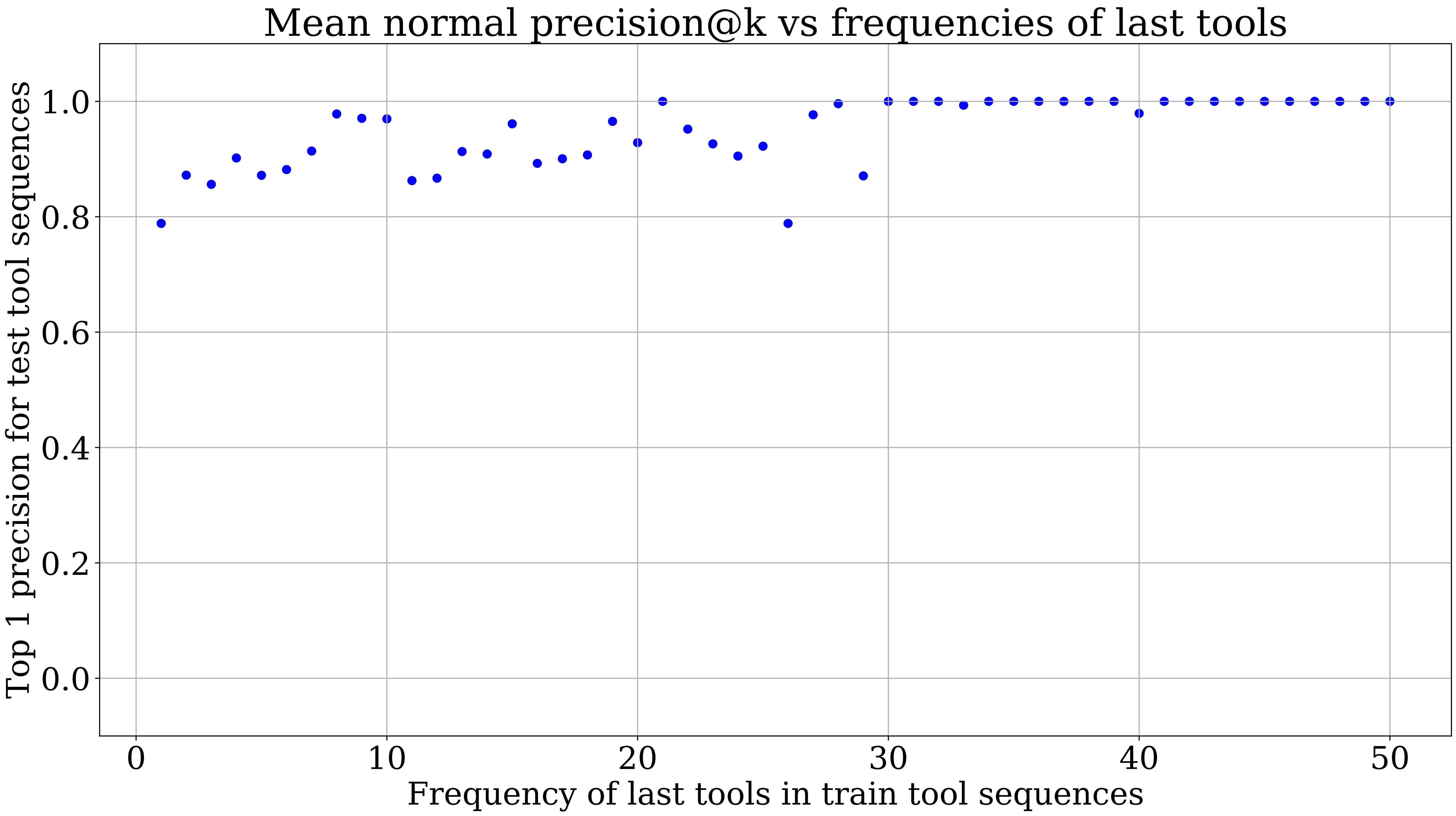

Click here to access/download;Figure;supplementary_figure_6.pdf

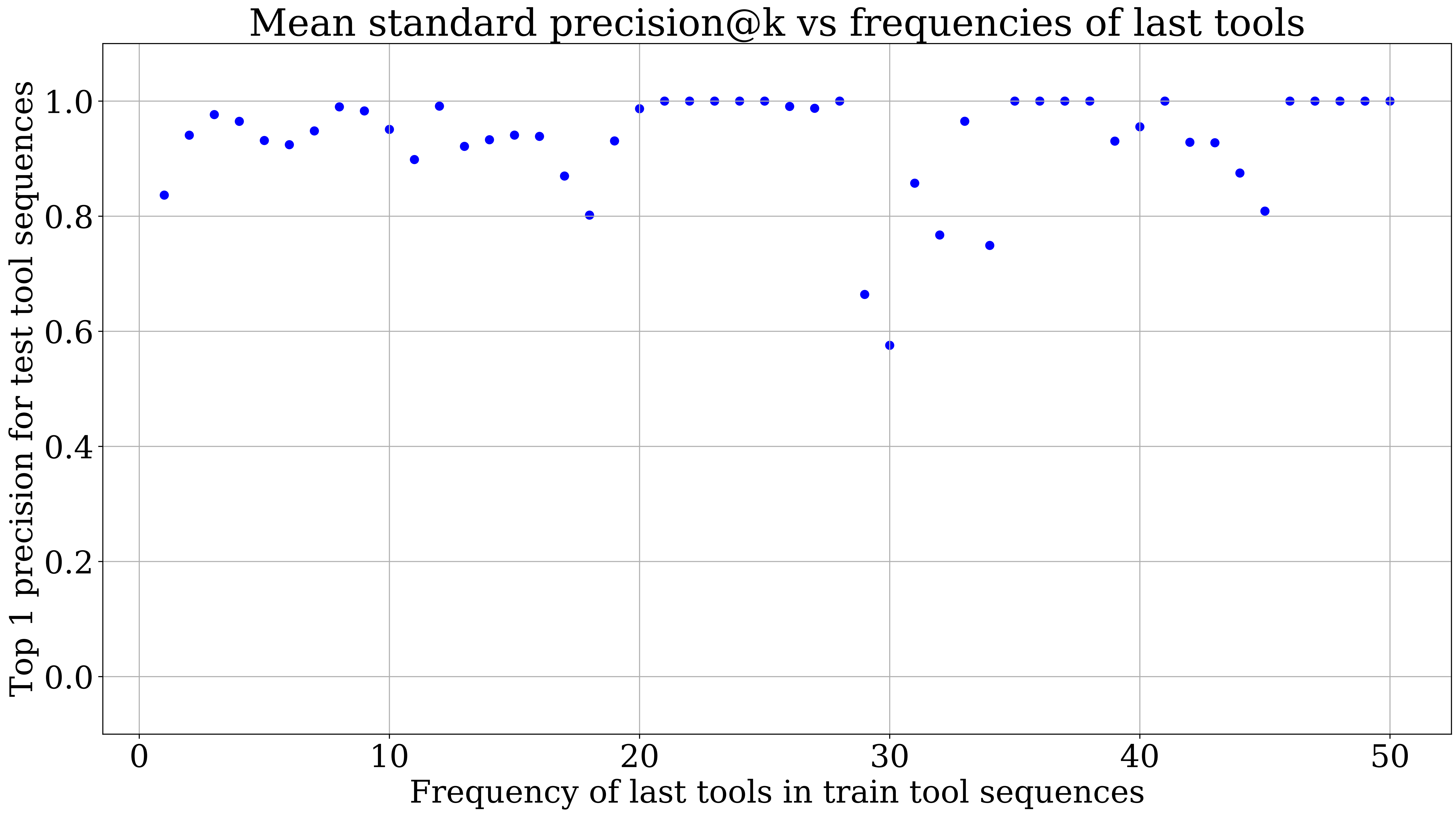

Click here to access/download;Figure;supplementary_figure_7.pdf

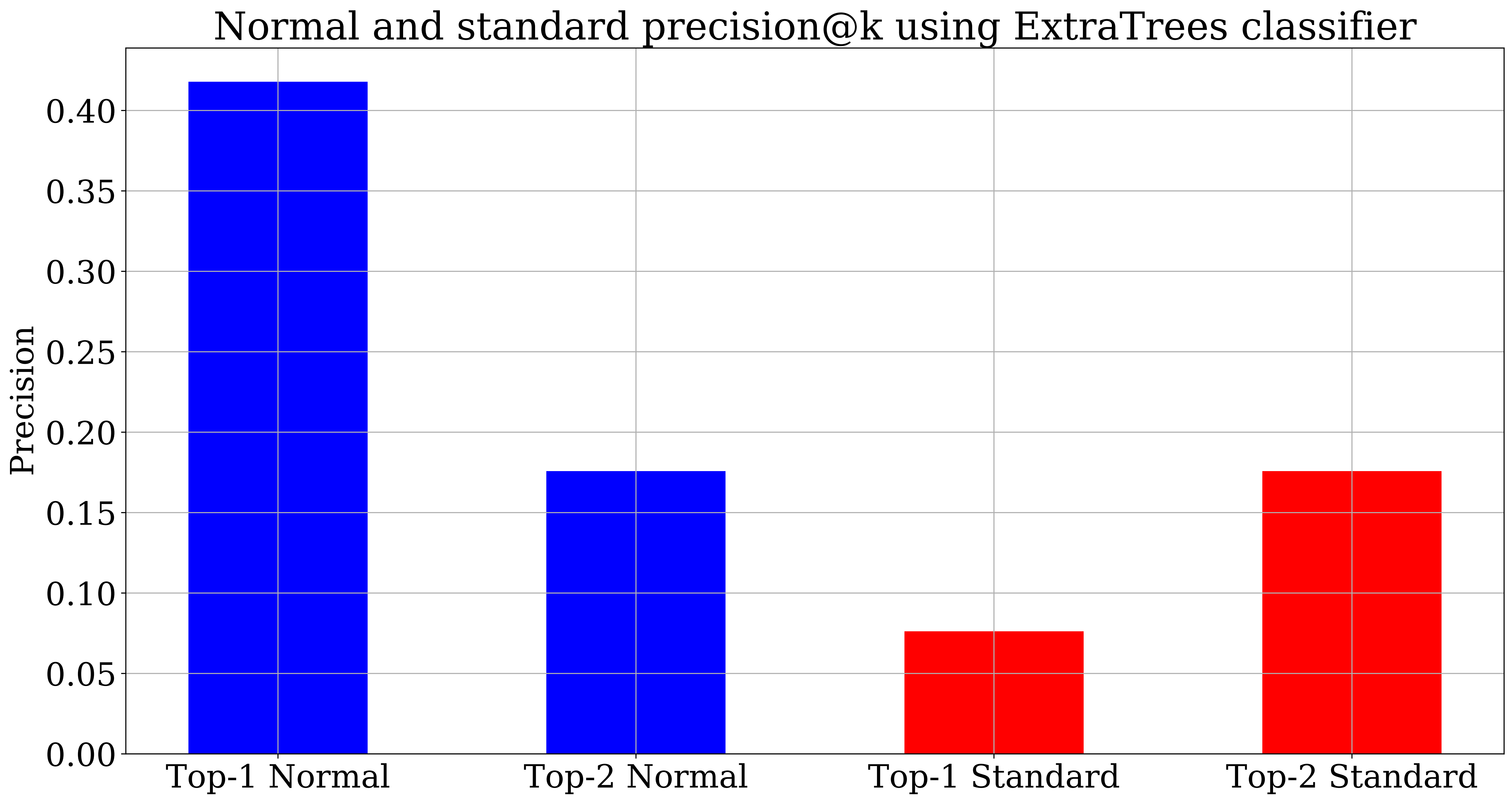

Click here to access/download
Supplementary Material
Tool_recommendations_supplementary_file.pdf
