# Supplementary material for: Tool recommender system in Galaxy using deep learning
Source: Gigascience. 2021 Jan 6;10(1):giaa152. doi: 10.1093/gigascience/giaa152 (PMC7786169; doi:10.1093/gigascience/giaa152)
Supplement: giaa152_GIGA-D-20-00053_Revision_3 [file giaa152_giga-d-20-00053_revision_3.pdf]

GigaScience
Tool recommender system in Galaxy using deep learning
--Manuscript Draft--

**Manuscript Number:**	GIGA-D-20-00053R3
**Full Title:**	Tool recommender system in Galaxy using deep learning
**Article Type:**	Technical Note
**Funding Information:**	DFG (German Research Foundation) (CIBSS - EXC-2189 - Project ID 390939984) — Dr. Rolf Backofen<br>BMBF grant (de.NBI) (031A538A) — Dr. Björn Grüning
**Abstract:**	Background Galaxy is a web-based and open-source scientific data-processing platform. Researchers compose pipelines in Galaxy to analyse scientific data. These pipelines, also known as workflows, can be complex and difficult to create from thousands of tools, especially for researchers new to Galaxy. To help researchers with creating workflows, a system is developed to recommend tools that can facilitate further data analysis. Findings A model is developed to recommend tools using a deep learning approach by analysing workflows composed by researchers on the European Galaxy server.The higher-order dependencies in workflows, represented as directed acyclic graphs, are learned by training a gated recurrent units (GRU) neural network, a variant of a recurrent neural network (RNN). In the neural network training, the weights of tools used are derived from their usage frequencies over time and the sequences of tools are uniformly sampled from training data. Hyperparameters of the neural network are optimised using Bayesian optimisation. Mean accuracy of 98% in recommending tools is achieved for the top-1 metric. Conclusions The model is accessed by a Galaxy API to provide researchers with recommended tools in an interactive manner using multiple user interface (UI) integrations on the European Galaxy server. Good quality and highly-used tools are shown at the top of the recommendations. The scripts and data to create the recommendation system are available under MIT license at https://github.com/anuprulez/galaxy_tool_recommendation .
**Corresponding Author:**	Anup Kumar<br>Albert-Ludwigs-Universitat Freiburg<br>Freiburg, GERMANY
**Corresponding Author Secondary Information:**	
**Corresponding Author's Institution:**	Albert-Ludwigs-Universitat Freiburg
**Corresponding Author's Secondary Institution:**	
**First Author:**	Anup Kumar
**First Author Secondary Information:**	
**Order of Authors:**	Anup Kumar<br>Helena Rasche<br>Björn Grüning<br>Rolf Backofen
**Order of Authors Secondary Information:**	
**Response to Reviewers:**	Dear Anup<br><br>Your manuscript "Tool recommender system in Galaxy using deep learning" (GIGA-D-20-00053R2) has been assessed by our curators and editors for a final time. Based on the changes can you cite the GigaDB citation in the Available of Supporting Data Section at the end of the paper. The reference you should use is:

Powered by Editorial Manager® and ProduXion Manager® from Aries Systems Corporation

	Kumar A; Rasche H; Gruening B; Backofen R (2020): Supporting data for "Tool recommender system in Galaxy using deep learning" GigaScience Database. http://dx.doi.org/10.5524/100838<br><br>I've sent you an email with an annotated paper explaining the changes in more detail.<br><br>Author comment:<br><br>Thank you for your feedback. We have made the following changes to the manuscript according to the feedback received as annotations in the manuscript.<br><br>1. We have added ORCIDs of all the authors beside their respective email ids.<br><br>2. We have included the Galaxy's RRID (RRID:SCR_006281) in "Library, model and code repositories" section of the manuscript.<br><br>3. We have added a short section "Availability of supporting data" just below the section "Availability of supporting source code and requirements" in the manuscript and cited the GigaScience GigaDB DOI.<br><br>Thank you!
**Additional Information:**	
**Question**	**Response**
Are you submitting this manuscript to a special series or article collection?	No
**Experimental design and statistics**<br><br>Full details of the experimental design and statistical methods used should be given in the Methods section, as detailed in our Minimum Standards Reporting Checklist. Information essential to interpreting the data presented should be made available in the figure legends.<br><br>Have you included all the information requested in your manuscript?	Yes
**Resources**<br><br>A description of all resources used, including antibodies, cell lines, animals and software tools, with enough information to allow them to be uniquely identified, should be included in the Methods section. Authors are strongly encouraged to cite Research Resource Identifiers (RRIDs) for antibodies, model organisms and tools, where possible.	Yes

Powered by Editorial Manager® and ProduXion Manager® from Aries Systems Corporation

Have you included the information requested as detailed in our Minimum Standards Reporting Checklist?	
**Availability of data and materials**<br><br>All datasets and code on which the conclusions of the paper rely must be either included in your submission or deposited in publicly available repositories (where available and ethically appropriate), referencing such data using a unique identifier in the references and in the “Availability of Data and Materials” section of your manuscript.<br><br>Have you have met the above requirement as detailed in our Minimum Standards Reporting Checklist?	Yes

Powered by Editorial Manager® and ProduXion Manager® from Aries Systems Corporation

Main tex file
Click here to access/download;LaTeX - Main Document (TeX file);main.tex

OXFORD
(GIGA)^n SCIENCE

GigaScience, 0000, 1–12

doi: xx.xxxx/xxxx
Manuscript in Preparation
Technical Note

TECHNICAL NOTE

Tool recommender system in Galaxy using deep learning

Anup Kumar[1,*,†], Helena Rasche[1, ‡, †], Björn Grüning[1, §, †] and Rolf Backofen[1,2, ¶, †]

[1]Bioinformatics Group, Department of Computer Science, University of Freiburg, Georges-Koehler-Allee 106, 79110 Freiburg, Germany and [2]Signalling Research Centres BIOSS and CIBSS, University of Freiburg, Schaenzlestr. 18, 79104 Freiburg, Germany

*kumara@informatik.uni-freiburg.de https://orcid.org/0000-0002-2068-4695
‡helena.rasche@gmail.com https://orcid.org/0000-0001-9760-8992
§gruening@informatik.uni-freiburg.de https://orcid.org/0000-0002-3079-6586
¶backofen@informatik.uni-freiburg.de https://orcid.org/0000-0001-8231-3323
†Contributions follow the order of the names of authors

Abstract

Background Galaxy is a web-based and open-source scientific data-processing platform. Researchers compose pipelines in Galaxy to analyse scientific data. These pipelines, also known as workflows, can be complex and difficult to create from thousands of tools, especially for researchers new to Galaxy. To help researchers with creating workflows, a system is developed to recommend tools that can facilitate further data analysis. **Findings** A model is developed to recommend tools using a deep learning approach by analysing workflows composed by researchers on the European Galaxy server. The higher-order dependencies in workflows, represented as directed acyclic graphs, are learned by training a gated recurrent units (GRU) neural network, a variant of a recurrent neural network (RNN). In the neural network training, the weights of tools used are derived from their usage frequencies over time and the sequences of tools are uniformly sampled from training data. Hyperparameters of the neural network are optimised using Bayesian optimisation. Mean accuracy of 98% in recommending tools is achieved for the top-1 metric. **Conclusions** The model is accessed by a Galaxy API to provide researchers with recommended tools in an interactive manner using multiple user interface (UI) integrations on the European Galaxy server. Good quality and highly-used tools are shown at the top of the recommendations. The scripts and data to create the recommendation system are available under MIT license at https://github.com/anuprulez/galaxy_tool_recommendation.

Key words: Recommender system; Galaxy; Workflows; Deep learning; Neural networks; Gated recurrent units

Findings

Background

Life sciences depend increasingly on high-throughput data, turning them into data science to a large extent. However, raw high-throughput data does not have much value on its own without proper analysis and interpretation of the data. To simplify the data analysis process and to ensure a reproducible analysis, several workflow systems such as Bcbio-nextgen, Omics Pipe, Nextflow, Luigi, Toil and many others have emerged [1, 2, 3]. The main idea for workflow systems is based on the observation that any computational analysis of high-throughput data encompasses multiple steps such as quality control, preprocessing, quantification and statistical analysis to transform raw data into scientific results. Collec-

Compiled on: November 25, 2020.
Draft manuscript prepared by the author.

tively, these steps form a workflow where each step performs a definite transformation of the data, which can be performed using standardised tools. Using workflow for the analysis is simple and convenient and has several advantages. First, it is easy to replace individual tools by a newer version or to assess the influence of the associated step on the final result. Second, a workflow can be saved, shared and reused, which ensures reproducible research. Therefore, workflows are becoming essential in the analysis of scientific data and there are multiple platforms where researchers can create workflows for their analyses. However, a critical question is how to assess whether a generated workflow is state-of-art or even valid at all. To give a concrete example, one can use several real-valued input vectors (such as fluorescence-based measurement stemming from arrays), transform them into integer-based values in the first step and combine it with a tool that uses a count-based statistics (such as negative binomial distribution as used in DESeq2 [4]) to determine values that show high differential behaviour. While this workflow would run on a workflow system without problems and even produce some results, the generated results are not valid because of the wrong statistical model. Therefore, it is important to use a tool for each step in a workflow which can bring desired results. To make it possible, a system is needed which can recommend useful tools at each step while creating a workflow.

Galaxy and workflows

Galaxy is an open-source data processing platform which enables researchers to create and store their workflows for multiple scientific analyses [5]. A workflow in Galaxy is a directed acyclic graph and consists of one or many tool sequences to analyse scientific data such as DNA and RNA sequences. A tool consumes one or more data files as input and produces one or more data files as output and supports a number of formats of these input and output files. In workflows, the tools are connected one after another following a constraint that the adjacent tools must have compatible data types. In other words, the data types of output files of a tool should match the data types of input files of the following tool. Galaxy has thousands of accessible tools and acquiring familiarity and constructing workflows with these tools can be a complex and time-consuming task, especially for researchers new to Galaxy. To assist them in creating workflows and making them aware of the possible tools for further analyses, a recommender system is devised. The benefits of having such a system are manifold. First, it will avoid the loss of time spent in creating erroneous or less optimal workflows by choosing tools which may produce undesired results and thereby making researchers more efficient. Second, it will help researchers bypass the step of searching for tools separately, which will further reduce the time spent in creating workflows and at the same time increase the accessibility of tools. Third, it will promote good quality tools having higher usage frequencies in the past (last one year) to the top of the recommendations and downgrade those having lower usage frequencies to the bottom of the recommendations. It is achieved by assigning weights to tools which are derived from their usage frequencies over a period of time. Finally, it can be extended to promote the newly added tools in Galaxy by showing them alongside the recommended tools predicted using the neural network approach.

Recommender systems

The objective of having recommender systems in fields such as scientific literature search, online shopping, travel bookings, media-service providers and many other fields is to help people discover suitable, interesting and newly-released products. These recommended products are recognised based on the usage and purchasing patterns of people in the past. In the field of scientific literature search, the exponential increase in the number of published papers necessitates having a recommender system to help scientists explore relevant and recent papers quickly [6, 7, 8]. Recommender systems are significant in the world of commercial applications too. Companies such as Amazon and Netflix have appropriately used them to learn preferences of their respective customers in selecting products such as their favourite books or movies and to propose a few products out of a large catalogue. By enabling users and customers to discover reasonable and customised products, recommender systems have helped them grow as organisations [9, 10]. In short, recommender systems make it faster for users and customers to look through a few recommended products to find the most suitable ones. These successful implementations of recommender systems by organisations across the world working in diverse areas to assess the needs of their respective users in proposing relevant products motivated us to create a tool recommender system in Galaxy.

Related work

To simplify creating workflows for scientific analyses, a few approaches have been proposed which suggest alternative tools and workflows. EDAM (EMBRACE Data And Methods) and semantic annotations of tools are used to compose workflows automatically for mass-spectrometry based proteomics [11]. The annotations include the names, functionalities, input and output data types of tools. The PROPHETS (Process Realisation and Optimisation Platform using Human-readable Expression of Temporal-logic Synthesis) program generates suitable candidates of workflows which match the goal of the proposed workflow and its annotations [12]. WINGS (Workflow INstance Generation and Specialization) offers multiple variations of a workflow created using different tools. It makes use of the input parameters, types of datasets and functions of tools to build the variations [13, 14]. The approach used in [15] utilises data types to facilitate the automatic creation of workflows. All these approaches depend either on annotations or matching input and output data types of adjacent tools in workflows and they pose challenges such as the addition and maintenance of the meaningful annotations of tools and extracting input and output data types of adjacent tools. Moreover, these approaches have their workflow generation restricted to a few specific bioinformatics analyses such as proteomics or proteogenomics. In addition, they do not discuss the presence of higher-order relationships [16] in tool sequences of workflows. Our approach to recommend tools in workflows aims to overcome these challenges in the following manner. First, it does not require collecting and storing information about tools. Second, it takes into account the higher-order relationships among tools (Figure 1) in tool sequences. Finally, it incorporates workflows from multiple scientific analyses to produce the recommender system.

Sequential learning on workflows

Workflows, created by many researchers in Galaxy for different scientific analyses, are decomposed into numerous tool sequences (Figure 1). The sequential nature of these tool sequences where tools are connected one after another inspires us to apply similar learning techniques used for other sequential data such as text and speech. There are multiple studies in the fields of natural language processing, clinical research and speech recognition which apply deep learning techniques on se-

quential data to obtain good accuracy in predicting future items. The approach used in [17] finds context in long sequences of words for sentiment analysis and part-of-speech tagging using recurrent neural network (RNN) and achieves 85% and 93% accuracy, respectively. For clinical data, learning on long sequences of health states proves to be beneficial [18]. The health states of patients recorded at different time points are analysed by accessing their electronic health records. The future health states of patients could be predicted by training RNN on the sequences of their past health states to achieve 85% accuracy. Moreover, the variants of RNN are used to model speech and music signals [19, 20]. These successful studies benefit from sequential learning techniques using different variants of RNN. Therefore, in our work, a variant of RNN - gated recurrent units (GRU) - is used to create the tool recommender system in Galaxy.

A Bayesian network can also be used for modelling directed acyclic graphs (workflows) [21, 22]. It requires computing joint and conditional probabilities of nodes in graphs and an increase in the number of nodes can lead to a higher cost to compute these probabilities. In addition, making predictions by learning a probabilistic network is a hard problem [23, 24, 25]. Because of these drawbacks of using a Bayesian network, it is not used in our approach to create the recommender system in Galaxy.

Data description

Over 18,000 workflows from different scientific analyses such as RNA-seq, Variant-calling, Hi-C, Assembly, Single-cell, Proteomics and so on in the European Galaxy server [26] have been used to create the recommender system. A workflow consisting of 5 tools is shown in Figure 1a. It is divided into smaller tool sequences as shown in Figure 1b, 1c and 1d. The last tool, shown in green, of each tool sequence (of length n) is assigned as the label of the sub-sequence (of length n-1) shown in blue in Figure 1. A label is an output which is learned and predicted by the recommender system. In the neural network learning, a tool is a label. For example, in Figure 1b, Tools D and E are the labels of the sub-sequence Tool A → Tool B → Tool C. They show higher-order dependencies in their connections which implies that a tool is not only dependent on its immediate predecessor but also on all prior tools in the tool sequence. For example, in Figure 1c, the Tool C is dependent on Tools B and A. By analysing multiple workflow fragments in this way, the neural network learns that the label of a tool sequence Tool A → Tool B is Tool C. It is expected that dividing a tool sequence into fragments with a minimum length of two tools, as shown in Figure 1c and 1d, will improve the generalisation performance of the neural network because it gets more tool sequences with a variety of lengths to learn from. The dependencies shown in Figure 1b, 1c and 1d present in tool sequences are learned using the GRU neural network by modelling the conditional probability given by Equation 1 [27]. Using the approach explained above, more than 229,000 tool sequences are extracted.

Usage pattern of tools

Tools in Galaxy have different usage patterns. Some tools are used more often than other tools for multiple reasons such as differences in their functions and availability of similar but better tools. It is essential to analyse the usage patterns of tools because the recommender system proposes tools for researchers and these tools should have high relevance to their analyses. One of the key indicators of the relevance of tools can be their high usage frequencies. If a tool has been used often in the recent past, it implies that the tool is relevant. However, if a tool was used often a few years ago but has been used less often in the last six months then the relevance of that tool has certainly declined. The usage frequencies of tools (shown as labels in Figure 1) over the past year are shown in Figure 2.

Shared and non-shared workflows

The set of workflows may have poor quality workflows which are non-published or deleted or may have errors in their tool connections. To distinguish between good and poor quality tool connections, the labels for each tool sequence are divided into two parts - shared and non-shared labels. The shared labels come from the published, non-deleted and non-erroneous workflows while the non-published labels come from other workflows. While recommending tools for a tool or tool sequence, the shared labels are promoted to the top of the recommendations if available followed by the non-shared labels. This enables good quality tools to be shown as the top recommendations.

Imbalance in workflows

Tool sequences from these workflows may vary in number - some may occur more frequently and others may not. Therefore, the complete set of tool sequences may not be equally representative of all workflows coming from different scientific analyses. Learning on the imbalanced set of tool sequences can induce bias which may have an undesired outcome - good accuracy in recommending tools coming from highly frequent tool sequences and poor accuracy for tools coming from less frequent ones. To mitigate this bias, all tool sequences are chosen with uniform frequency while training the neural network which allows it to have a comparable accuracy in recommending tools in different scientific analyses. This uniform sampling strategy is discussed in the "Implementation" section in detail.

Results

Three different neural network architectures - dense neural network (DNN), convolutional neural network (CNN) and gated recurrent units neural network (GRU) - are compared on their performances in predicting tools (Figures 3 and 4). The models obtained after training all the neural network architectures are used to predict tools for the tool sequences in the test data after every training iteration. Top-k precision (precision@k) is a popular metric for evaluating a recommender system [28, 29, 30]. Precision@k implies how many in the k predicted tools are correct. The correctness here refers to the compatibility of the predicted tools with the tool for which predictions have been made. For example, $k = 2$ implies that the number of predicted tools are 2 with the highest predicted scores. If only 1 of them is correct, then the precision@2 is $\frac{1}{2} = 0.5$. In this way, precision@1 and precision@2 are computed for all the tool sequences in the test data and then averaged. Precision@1 and precision@2 metrics are used in this approach to evaluate the quality of the tool recommender system. The precision of recommended tools are computed separately for the non-shared and shared recommendations and shown in different plots (Figures 3 and 4) but for usage frequencies, they are combined into one plot (Figure 5). The precision and usage frequencies of the predicted tools for the precision@1 and precision@2 (top-1 and top-2) metrics are computed over 10 training iterations for each experiment run. They are averaged and their respective standard deviations are computed over 10 experiment runs. Mean precision and usage frequencies are shown by the respective line plots and the shaded regions span the area between one standard deviation above and below the mean (Figures 3, 4 and 5).

Comparison of GRU neural network with other approaches
The GRU neural network with the weighted cross-entropy loss function shows superior performance to CNN (Figures 3d, 3f, 4d and 4f) by achieving 98% top-1 non-shared and shared precision which proves that the GRU layers in a neural network are better for learning on tool sequences than the convolutional layer. Moreover, it shows a lower divergence in non-shared and shared precision and usage frequencies (Figures 3d, 3f, 4d, 4f, 5d and 5f) establishing that its predictive strength is more stable than CNN over multiple experiment runs. Surprisingly, the weighted cross-entropy loss function does not have any beneficial effect on the CNN architecture as its non-shared and shared precision and usage frequencies show higher divergence over multiple experiment runs (Figures 3c, 3d, 4c, 4d, 5c and 5d). Therefore, CNN is not used in our approach. In contrast to CNN, DNN achieves a similar non-shared and shared precision to the GRU neural network with a small divergence (Figures 3a, 3b, 3f, 4a, 4b and 4f). However, due to higher divergence in accumulated usage frequencies, it is not used in our approach (Figures 5a, 5b and 5f). Weighted cross-entropy loss function in the GRU neural network (Figure 5f) drives it to classify tools more robustly with higher usage frequencies. In other words, it predicts tools with higher precision than CNN and lower divergence in usage frequencies than DNN. Therefore, it is used in our approach to learn on tool sequences and recommend tools.

To compare the performance of the GRU neural network with approaches which do not use any neural network, two ideas are explored. The first approach simply stores all the sequences of tools [31] formed using the technique shown in Figure 1 to create a model. To recommend tools using this model, all the tool sequences are searched for a given tool or a sequence of tools. The second approach uses ExtraTrees classifier [32] to recommend tools. These approaches are discussed in section S1 of the supplementary document in detail.

Benefit of regularisation
Using regularisation minimises overfitting by assisting the GRU neural network to make better recommendations by predicting tools having low usage frequencies but useful in addition to tools with high usage frequencies. For example, the recommendations of "UMI-tools count" [33] tool with the regularised model include "Seurat" [34] tool which is absent from recommendations by the non-regularised model. Another example is for "RaceID, Lineage computation using StemID" tool sequence which gets "Lineage Branch Analysis using StemID" [35] tool as one of the recommendations by the regularised model while there is no recommendation at all by the non-regularised model. The recommendations for a popular mapper, RNA-STAR [36], are "featureCounts" [37], "MultiQC" [38], "Infer Experiment" [39] and few others by both the models. But, in addition to these recommendations, the regularised model recommends the "Read Distribution" [39] tool which is not predicted by the non-regularised model. More details have been provided in section S4 of the supplementary document.

Examples of tool recommendations
To illustrate the real time usage of the recommender system in the European Galaxy server, two examples have been provided. The first one shows recommended tools for a tool sequence with 3 tools, Trimmomatic [40] → BWA-MEM [41] → FreeBayes [42], in the workflow editor of the European Galaxy server (Figure 6). Trimmomatic is used to trim the sequencing data such as DNA and RNA sequences. One of the useful analyses after trimming the sequences is to map them on a reference genome using a mapper. Several mappers such as BWA-MEM [41], Bowtie2 [43] and RNA-STAR [36] are predicted. BWA-MEM is chosen from the predicted mappers and connected to Trimmomatic. After mapping, for further analysis of mapped sequences, many tools are predicted such as MultiQC for summarising the quality of mapping, featureCounts for counting the reads mapped to different regions on the genome or FreeBayes for detecting variants and a few others. FreeBayes is chosen and a list of recommendations is shown as a dropdown containing tools such as "bcftools norm" [44], VcfAllelicPrimitives [45] and many others for Trimmomatic → BWA-MEM → FreeBayes tool sequence. Another example of tool recommendations after using RNA-STAR is shown in Figure 7. It shows follow-up tools such as bamCoverage [46] for calculating read coverage, MultiQC, featureCounts and a few others. In summary, the tool recommendations provide useful knowledge about tools to Galaxy users and researchers to continue multiple scientific analyses.

Table 1 lists a few shared and non-shared recommended tools for multiple tool sequences in different scientific analyses such as Computational chemistry, Epigenetics, Machine learning, Proteomics, RNA sequencing and a few others. The recommended tools shown in this table are highly used for standard scientific analyses as highlighted in multiple GTN training materials [60].

Implementation

To create a tool recommender system in Galaxy, workflows are collected from the European Galaxy server. A workflow may have one or many tool sequences where tools are connected one after another. Tool sequences are transformed into matrices and produced as input to a GRU neural network to learn patterns in the connections of tools.

$$p(x_T | x_1, x_2, ..., x_{T-1}) \tag{1}$$

The probability of a tool (x_T) is estimated given all other prior tools (x_1, ..., x_{T-1}) for a tool sequence (x_1, ..., x_{T-1}, x_T). Neural network learning is classification because there are labels for tool sequences which are learned and then predicted. Moreover, the classification is multi-class (multiple tools as labels) and multi-label (multiple tools as labels for a tool sequence) [61]. To ensure an unbiased learning and evaluation by the neural network, the set of tool sequences is divided into two parts - training and test. The training data is used for learning a model and the test data is used for evaluating the model.

Uniform sampling
Workflows in Galaxy come from different scientific analyses. It may happen that the number of workflows from these analyses are not comparable - some analyses may have a large number of workflows while some may have only a small number of workflows. This can cause some tools to be present very frequently in workflows while other tools are less frequent. Learning on these workflows and recommending tools may exhibit bias by showing better recommendations for the frequently occurring tools and poorer recommendations for the less frequent ones. To showcase this imbalance, the frequencies of the last tool in each tool sequence in training data are calculated and it is found that only a few tools have large frequencies and most of the tools are present in low frequencies (Supplementary Figure 3). For example, the tools with very high frequencies (> 10,000) are “Concatenate datasets”, “Cut”, “Grouping” and “Join” while the tools having very low frequencies (< 5) are “Cluster inspection using RaceID”, “rDock cavity definition” [62] and “ChiRA collapse”. Therefore, to overcome this drawback, the training data created after extracting tool sequences should be balanced to make the neural network learn

Table 1. Shared and non-shared recommendations for tool sequences in different scientific analyses

Scientific analyses	Tool/Tool sequences	Recommended tools (shared)	Recommended tools (non-shared)
Computational chemistry	Molecule to fingerprint [47]	Taylor-Butina clustering, NxN Clustering [48], Similarity Search	-
Epigenetics	hicBuildMatrix [49]	hicSumMatrices	hicMergeMatrixBins, hicPlotMatrix [50], hicPCA, hicTransform
Epigenetics	multiBamSummary [46]	plotCorrelation [51]	plotPCA
Epigenetics	Bowtie2	MASC2 CallPeak [51]	bamCoverage, FreeBayes
Machine learning	Create a deep learning model architecture	-	Create deep learning model [52], Build Deep learning Batch Training Models
Proteomics	Msconvert [53]	Search GUI, FlashLFQ	PeakPickerHiRes [54]
RNA Sequencing	Cutadapt [55]	FastQC, RNA-STAR, MultiQC [56]	Bowtie2, Hisat2, BWA-MEM
RNA Sequencing	Cutadapt [55], RNA-STAR	featureCounts, MultiQC, Infer Experiment [56]	bamCoverage, RmDup
Single-cell	UMI-tools extract [33]	RNA-STAR [57]	Bowtie2, Hisat2, BWA-MEM, UMI-tools group
Single-cell	Initial processing using RaceID [35]	Clustering using RaceID [58]	-
Single-cell	Initial processing using RaceID, Clustering using RaceID [35]	Cluster Inspection using RaceID [58], Lineage computation using StemID	-
Variant-calling	FreeBayes	VcfAllelicPrimitives [59]	Gemini load
Variant-calling	FreeBayes,VcfAllelicPrimitives	SnpSift Filter, VT normalize	SnpEff eff [59]

on a similar number of tool sequences from different scientific analyses in each training iteration. To implement this strategy, a set of last tools in all tool sequences from the training data is collected. Further, for each tool in this set, a list of indices of tool sequences in the training data are stored for which it is the last tool (Supplementary Table 3). Only the last tools are considered for implementing this strategy because of two reasons. First, the smallest tool sequences contain only two tools and second, all tools become the last tool in at least one tool sequence and the computed frequencies of these last tools suggest their overall frequencies in the training data.

In the neural network training, for each iteration (which consumes all tool sequences in the training data), small batches containing an equal number of tool sequences are created. For example, if the batch size is 100 and the size of training data is 2000, then 20 (2000/100=20) batches are created, each containing 100 tool sequences. In each batch, 100 tools from the set of last tools are uniformly selected (Column 2 in Supplementary Table 3) and for each selected tool, a tool sequence is chosen uniformly from its respective list of tool indices (Column 3 in Supplementary Table 3). After selecting tool sequences for many batches for each iteration of training (epoch), it is expected that all the tools from the set of last tools and their respective tool sequences are chosen. Performing this uniform selection of different tool sequences for each iteration, the training data becomes balanced. Supplementary Figure 4 shows that each last tool is present approximately 1670 times on an average in each iteration (epoch). The order of tools in the Supplementary Figures 3 and 4 are same.

Data transformation

Tool sequences extracted from workflows are transformed into vectors because neural networks require input data to be represented as vectors and matrices. Each tool sequence has one or more labels (Figure 1) and they are transformed into different vectors - a tool sequence vector (Figure 8b) and a label vector (Figure 8d). To form these vectors, a dictionary of tools is needed which stores an index for each tool. Using the indices of tools, a tool sequence vector is created preserving the original order of tools as in the tool sequence. For example, Tool A has an index of "12" in the dictionary, therefore it is replaced by "12" in the vector (Figure 8b). The vector is padded with trailing zeros to keep the length of the vector same across the varying lengths of tool sequences. The size of this vector is 25 which means that a tool sequence can have a maximum of 25 tools. The tool sequences larger than this size are discarded. The labels (Figure 8c) are transformed into a bit vector (Figure 8d) in which the positions, stored as indices in the dictionary of tools, of the labels (tools) are turned "on" (set to 1) specifying that these tools are the labels of the tool sequence and

others are not (set to 0). It has the same size as the dictionary of tools. In the machine learning field, it is also known as a multi hot-encoded vector. Together, these two vectors form a training sample for the neural network. A pair of vectors are created in this manner for each tool sequence and for all the tool sequences, they are combined to form two matrices - one for tool sequences and another for their respective labels. Internally, the label vectors have two subsets, one for shared labels (from the published, non-deleted and non-erroneous workflows) and another for non-shared labels (from the rest of the workflows). These matrices form input data to the neural network.

Neural network architecture

GRU neural network, a variant of RNN, is used for creating a model to recommend tools. The neural network architecture has multiple components such as different layers (Figure 9), activation functions, class weights, loss function, hyperparameter tuning technique which are discussed in detail in the following paragraphs.

Embedding layer. The first component of the neural network architecture is an input layer (Figure 9) which learns an embedding, a fixed-size vector, for each tool. This vector is used by the neural network as an internal representation of a tool. The embedding vector replaces the indices of tools in each tool sequence. The size of the embedding vector is fixed for all tools. For example, the vector of a tool sequence [12, 6, 75, 0, 0, ..., 0] is transformed into [[0.3, 0.01, 0.003, ..., 0.23], [0.5, 0.1, 0.005, ..., 0.9], [...], 0, 0, ...,0] by the embedding layer. The same embedding vector represents a tool in all tool sequences in which the tool is present.

GRU layer. The stacked layers of GRU learn deeper structures in the tool sequences by modelling the conditional probabilities of tools (labels) given all other prior tools (Figure 9). GRU has certain advantages which help it to learn on sequential data. First, it avoids the problems of vanishing and exploding gradients which commonly occur in traditional RNN [63]. It is important because learning higher-order dependencies depends on the gradients of errors concerning the parameters (recurrent and input weight matrices) of GRU layers. Second, GRU has slightly fewer parameters than the long short-term memory network (LSTM), another variant of RNN, which makes using GRU simpler than LSTM. Finally, it achieves similar accuracy as the LSTM [19].

Output layer. The last component of the neural network architecture is a dense layer which computes predictions (Figure 9). The dimension of this layer is equal to the number of unique tools because it predicts a score for each tool (label). The predicted score of each tool is considered as its probability of being the label of an input tool sequence. The closer the predicted score of a tool is to 1, the more probable it is to be the recommended tool and the closer it is to 0, the less probable it is to be the recommended tool.

Dropout layer. Overfitting happens when a neural network performs exceptionally well on the training data but its performance on test (unseen) data remains poor. To minimise the effect of overfitting, a dropout layer is used between two layers of the neural network. It sets a few randomly chosen connections to 0 in the neural network to introduce some randomness to minimise overfitting [64, 65]. 3 dropout layers are used in our approach - one between the embedding and the first GRU layers, one between 2 GRU layers and the last one between the second GRU and dense layers.

Activations. These are mathematical functions which are used in neural networks to transform inputs to a layer into its outputs. Two activations are used in our approach - one is exponential linear units (ELU) [66] and another is sigmoid (Equation 2). ELU is used for both the GRU layers and has a special feature of being negative when the input is negative which allows mean activation (output) to get closer to 0 compared to other activation functions such as ReLU [67] which is always positive. As mean activations get closer to 0, the approximated and actual gradients get closer to each other. Therefore, using ELU in our neural network as an activation can be useful to achieve faster training, an increased drop in loss and better accuracy. Sigmoid is used in the output layer which normalises any real number to lie between 0 and 1 and it is considered as a probability of each tool.

$$f(x) = \frac{1}{1 + e^{-x}} \tag{2}$$

Usage frequencies of tools as weights. To incorporate the usage frequency of tools in the recommender system, the usage frequencies of all the tools used in the last one year have been collected and are used in the neural network training as the weights of tools. A tool which has been used often (for example Tool B in Figure 2) in the past one year is assigned a higher weight than a tool (for example Tool C in Figure 2) which has been used less often in the past one year. When tools are recommended a score is assigned to each tool by the neural network. It is expected that a tool with a higher weight gets a higher score and a tool with a lower weight gets a lower score. To summarise, the relevance of a tool to be used in a workflow decays if its usage drops in Galaxy over time. This weighing scheme filters out tools from the list of recommendations which have not been used in the last one year irrespective of their origin, either shared or non-shared workflows.

Alternatively, the relevance of tools can also be ascertained by counting the occurrence of each tool in all workflows and these occurrences can be used as their weights in the neural network training. But, it may happen that some tools which were used often in the past to create workflows are not used anymore. Therefore, assigning weights to these tools in the neural network training based on their occurrences in workflows may not be a good indicator of their relevance and overall, may not be optimal.

A curve is fit through the usage frequencies of each tool using support vector regression (SVR) to display a trend of the usage of tool over time. Using this trend, the usage of the tool for the next month is predicted and its logarithm is used as the weight for this tool in the neural network training. The logarithm of usage frequencies is computed to normalise them because only a few tools have a significantly large magnitude of usage compared to that of the remaining tools which may lead the neural network to learn and predict only tools with a very large magnitude of usage and ignore other tools. Learning a trend for each tool involves 5-fold cross-validation and optimising two hyperparameters of SVR, kernel and degree, using grid search. The values used for the kernel are - "rbf", "poly" and "linear" and the values of degree used are 2 and 3. By following the grid search, there are 3 (kernels) x 2 (degrees) = 6 different combinations of hyperparameters to be verified to find the best curve for each tool [68].

Loss function. A neural network learns patterns from data by minimising a loss function. Cross-entropy is a popular choice for a loss function in classification problems [69]. In our approach, cross-entropy function is used in the GRU neural network to compute the loss between the true and predicted label

and is weighted by the label's weight. The loss is summed up over all labels of a tool sequence and then averaged (Equation 3). The term T is the total number of labels (size of the label bit vector). The term w_i is the weight of the ith label. The terms p^a and p^b refer to the true and predicted label vectors for a tool sequence, respectively. In general, the loss is large when p^a and p^b are far away from each other which means that the learning by the neural network is not good. If they are close, the loss is low and the predictions are better. When an unweighted cross-entropy is used as the loss function for any classification problem [70] then it is assumed that all the predictions have the same weight and it does not differentiate between the more and less dominant labels. In our approach when it is used as a loss function in the neural network, then even though the predicted labels are correct they may not necessarily have large weights and thereby may be less relevant. Therefore, to reduce the possibility of less relevant labels appearing in recommendations, loss is weighted by the weights of labels. It means that if a label with a larger weight is misclassified, which means that the true and predicted values are different, then the overall loss is higher. In this way, the wrong classification of a label with a larger weight is penalised more than the wrong classification of a label with a smaller weight.

$$loss = -\frac{1}{T}\sum_{i=1}^{T}(p_i^a \cdot \log(p_i^b) + (1 - p_i^a) \cdot \log(1 - p_i^b)) * w_i \quad (3)$$

The loss in Equation 3 is computed for all tool sequences in training data and is minimised using a root mean square propagation (RMSProp) optimiser. It follows an adaptive approach to estimate the learning rate by keeping knowledge of gradients in prior iterations. The learning rate is updated by dividing it with an average of the square of the prior gradients [71].

Hyperparameter tuning. The hyperparameters in our approach are optimised using Bayesian (sequential model-based) optimisation [72]. It learns from the previously evaluated configurations which ensure faster convergence. Reasonable ranges of all the hyperparameters to be optimised are given and the best configuration is found after 20 evaluations. More details are given in section S7 of the supplementary document.

Learning and predictions

Over 229,000 tool sequences collected from more than 18,000 workflows are divided into training and test data. A neural network learns patterns in the tool sequences from the training data and creates a model. The ability of the model to recommend tools is evaluated on the test data which is unseen by the neural network during training. The training data forms 80% (approximately 185,000) of all tool sequences and it is iterated over 10 epochs of neural network training. The remaining 20% (approximately 45,000) is used as the test data. The running time of the training is approximately 50 hours on a high performance compute cluster provided by bwCloud [73] with multiple cores. After learning on the training data, the model is used to predict tools. Each predicted tool gets a probability score of being the recommended tool of a tool or tool sequence. Two sets of predictions are made - shared and non-shared. Each set is sorted in the descending order of their probabilities and the top ones in both sets are combined to show them as recommendations.

Summary and future work

A system to recommend tools in Galaxy is built by analysing workflows using a variant of RNN (GRU) and a weighted cross-entropy loss function. The recommended tools are relevant for multiple scientific analyses with high accuracy as shown by the high similarities between the tools used in GTN tutorials and the recommended tools for similar analyses (Table 1). Moreover, they are easily accessible through simple UI integrations in Galaxy (Figures 6 and 7). Collectively, they improve user experience by helping researchers to easily create correct workflows. In addition, the approach does not store any information about tools and the recommendations are made by learning only the patterns of tool connections in workflows. The model [74] created using this approach and an API [75] are integrated into the European Galaxy server. The API resides with other Galaxy APIs and accesses a tool or a tool sequence specified by researchers to show its recommendations in real time using the model. The API is used at two different user interfaces in Galaxy - one shows recommendations in the workflow editor (Figure 6) and another shows them after each tool execution (Figure 7). The recommendation system should be potentially helpful for those researchers who are new to the Galaxy platform. It shows them a few follow-up tools from a big collection of more than 3,000 tools and enables them to perform multiple exploratory data analyses.

Different Galaxy servers maintain different sets of tools and workflows. The current approach can be used to create different recommendation models for different Galaxy servers. Alternatively, all the workflows can be collected from multiple Galaxy servers and using the current approach, one recommendation model can be created by learning on the complete set of workflows and the model can be distributed to different Galaxy servers. To improve the quality of recommendations, the annotations of tools can be incorporated in the learning mechanism by assigning higher weights to the annotated tools in comparison to tools which are not annotated. Tools containing similar annotations may have similar functionalities and using these similarities, tool recommendations can be further enhanced by showing similar tools for each recommended tool. In addition to learning tool connections to recommend tools, the knowledge of tools connecting to different tools based on their respective parameters can also be incorporated.

Methods

Library, model and code repositories

The Keras deep learning library is used for producing the neural network architectures [76]. The trained model [74] is saved as an H5 file to simplify its distribution to different Galaxy instances (RRID:SCR_006281). The file is an HDF5 store containing the weights of different layers of the neural network and their configurations, a dictionary of tools and their indices and the weights of tools. The weights and configuration of the neural network are needed to recreate the trained model. The dictionary is used to replace IDs of the predicted tools by their indices in a tool sequence. All data and python scripts used in our approach are stored at Github for all approaches - GRU [77], CNN [78] and DNN [79]. In each of these repositories, the process to create a tool recommendation model is explained. All these repositories are provided with a script ("extract_data.sh") for collecting raw input datasets from a Galaxy instance. These datasets are workflows and usage frequencies of tools and are also provided in each repository. The values of multiple hyperparameters of neural networks, number of training iterations and sizes of training and

test data can be altered using a bash script ("train.sh"). To execute the scripts on a GPU enabled machine, the "tensorflow-gpu" package should be installed instead of "tensorflow" as mentioned in the conda package dependencies file ("environment.yml"). To see recommended tools, an ipython script ("tool_recommendation_gru_wc.ipynb" for GRU repository) is also provided which loads and recreates a trained model to predict tools for a tool or a tool sequence. The result files storing precision, training and validation losses and usage frequencies, which are used for generating line plots (Figures 3, 4 and 5), for all approaches are also available at Github [80]. The code repositories of two other approaches which do not use neural networks are available at - simple approach [31] and ExtraTrees [32].

New recommendation model

On a usual Galaxy server, tools and workflows are dynamic as they are added and updated regularly. Therefore, it is important to train the GRU neural network on the complete set of workflows periodically to keep the tool recommendation model updated with the latest tools and workflows. Using a Galaxy tool [81], a new recommendation model can be created after collecting workflows and tool usage data from a Galaxy server. The tool runs for several hours (> 24 hours) and creates a model which is pushed to an online repository [74]. From this repository, Galaxy downloads it using an API [75] to recommend tools. The recommendation model is created periodically every 3-4 months to accommodate new workflows and tools. Galaxy admins can decide upon the frequency of creating a new model. It can be created every month or every 6 months

New tools as recommendations

Galaxy admins can overwrite the recommended tools predicted using the trained model by a different set of tools using the configuration option described in [82]. In addition, to highlight the newly added tools, which are not part of the model, they can be appended to the recommendations using this additional configuration option.

Availability of supporting source code and requirements

Project name: Tool recommender in Galaxy using deep learning
Project home page: https://github.com/anuprulez/galaxy_tool_recommendation
Operating system: Linux
Programming languages: Python, XML, JavaScript
Other requirements: Tensorflow, Keras, Scikit-learn, Numpy, H5py, Csvkit, Hyperopt
License: MIT License
RRID: SCR_018491
Biotools ID: tool_recommender_system_in_galaxy

Availability of supporting data

A snapshot of the source code is available in the GigaScience GigaDB repository [83].

Additional files

File name: Tool_recommendations_supplementary_file.pdf Title: Supplementary Material: Tool recommender system in Galaxy using deep learning Description: There 3 tables - Table 1 shows a comparison of recommendations between the GRU neural network, a simple model and ExtraTrees classifier, Table 2 shows a comparison of recommendations between the regularised and non-regularised GRU neural network and Table 3 shows the strategy of uniform sampling of training data. There are 7 figures - Figures 1 and 2 show architectures of convolutional neural network (CNN) and dense neural network (DNN) used in the paper, Figure 3 shows original frequencies of last tools in training data, Figure 4 shows the frequencies of last tools in training data after uniform sampling, Figures 5 and 6 show top-1 non-shared and shared precision respectively for less frequent tools and Figure 7 shows the top-1 and top-2 precision (non-shared and shared recommendations) of the ExtraTrees classifier.

Declarations

List of abbreviations

API: Application programming interface; CNN: Convolutional neural network; DNN: Dense neural network; ELU: Exponential linear units; GRU: Gated recurrent units; PROPHETS: Process realisation and optimisation platform using human-readable expression of temporal-logic synthesis; RNN: Recurrent neural network; SVR: Support vector regression; UI: User interface; WINGS: Workflow instance generation and specialization;

Ethics approval and consent to participate

Not applicable

Consent for publication

Not applicable

Competing interests

The authors declare that they have no competing interests.

Funding

This work was supported by the German Research Foundation (DFG) under Germany's Excellence Strategy (CIBSS - EXC-2189 - Project ID 390939984) and German Federal Ministry of Education and Research (BMBF grant 031A538A de.NBI).

Authors' contributions

First author (A.K.) implemented the project and wrote the manuscript. The second author (H.R.) wrote scripts for data collection, contributed to the manuscript and deployed the project on European Galaxy server. The third author (B.G.) provided the idea of the project, validated results, and contributed to the manuscript. The last author (R.B.) contributed to the manuscript. All authors approved the manuscript.

Acknowledgements

We thank Simon Bray and Joachim Wolff for proofreading the manuscript, Dr. Wolfgang Maier for providing feedback and Gianmauro Cuccuru for deploying it on the European Galaxy server.

References

1. Ewels P, Krueger F, Käller M, Andrews S. Cluster Flow: A user-friendly bioinformatics workflow tool. F1000Research 2017;5:2824, doi:10.12688/f1000research.10335.2.
2. Leipzig J. A review of bioinformatic pipeline frameworks. Brief Bioinform 2017;1;18(3):530–536, doi:10.1093/bib/bbw020.
3. Baichoo S, Souilmi Y, Panji S, Botha G, et al. Developing reproducible bioinformatics analysis workflows for heterogeneous computing environments to support African genomics. BMC Bioinformatics 2018;19, 457 (2018), doi:10.1186/s12859-018-2446-1.
4. Love MI, Huber W, Anders S. Moderated estimation of fold change and dispersion for RNA-seq data with DESeq2. Genome Biol 2014;550. doi:10.1186/s13059-014-0550-8.
5. Afgan E, Baker D, Batut B, et al. The Galaxy platform for accessible, reproducible and collaborative biomedical analyses: 2018 update. Nucleic Acids Research 2018;46(W1):W537–W544. doi:10.1093/nar/gky379.
6. Bela G, Beel J, Hentschel C. Scienstein : A Research Paper Recommender System. In: Proceedings of the International Conference on Emerging Trends in Computing; 2009. p. 309–315. http://www.sciplore.org/wp-content/papercite-data/pdf/gipp09.pdf.
7. Achakulvisut T, Acuna DE, Ruangrong T, Kording K. Science Concierge: A Fast Content-Based Recommendation System for Scientific Publications. PLoS ONE 2016;11(7): e0158423, doi:10.1371/journal.pone.0158423.
8. Wang D, Liang Y, Xu D, et al. A content-based recommender system for computer science publications. Knowledge-Based Systems 2018;157:1–9. doi:10.1016/j.knosys.2018.05.001.
9. SGomez-Uribe CA, Hunt N. The Netflix Recommender System: Algorithms, Business Value, and Innovation. ACM Transactions on Management Information Systems TMIS 2016;6(4).
10. Smith B, Linden G. Two Decades of Recommender Systems at Amazon.com. IEEE Internet Computing 2017;21(3):12–18. doi:10.1109/MIC.2017.72.
11. Palmblad M, L LA, Ison J, Schwämmle V. Automated workflow composition in mass spectrometry-based proteomics. Bioinformatics 2019;35(4):656–664. doi:10.1093/bioinformatics/bty646.
12. Naujokat S, Lamprecht AL, Steffen B. Loose Programming with PROPHETS, Fundamental Approaches to Software Engineering, vol. 7212. J L, A Z, editors, Springer, Berlin, Heidelberg; 1996. Online ISBN 978-3-642-28872-2, doi: 10.1007/978-3-642-28872-2_7.
13. Gil Y, Ratnakar V, Kim J, et al. Wings Intelligent Workflow-Based Design of Computational Experiments. IEEE Intelligent Systems 2011;26(1):62–72. doi:10.1109/MIS.2010.9.
14. Srivastava A, Adusumilli R, Boyce H, et al. Semantic workflows for benchmark challenges: Enhancing comparability, reusability and reproducibility. PSB 2018;doi:10.1142/9789813279827_0019.
15. DiBernardo M, Pottinger R, Wilkinson M. Semi-automatic web service composition for the life sciences using the biomoby semantic web framework. Journal of Biomedical Informatics 2008;41(5):837–847. doi:10.1016/j.jbi.2008.02.005.
16. Michalski V, Memisevic R, Konda KR. Modeling sequential data using higher-order relational features and predictive training. CoRR 2014;abs/1402.2333. http://arxiv.org/abs/1402.2333.
17. Yin W, Kann K, Yu M, Schütze H. Comparative Study of CNN and RNN for Natural Language Processing. ArXiv 2017;abs/1702.01923. http://arxiv.org/abs/1702.01923.
18. Lipton ZC, Kale DC, Elkan C, Wetzel R. Learning to diagnose with LSTM recurrent neural networks. CoRR 2015;abs/1511.03677.
19. Chung J, Gulcehre C, Cho K, Bengio Y. Empirical evaluation of gated recurrent neural networks on sequence modeling. In: NIPS 2014 Workshop on Deep Learning, December 2014; 2014. .
20. Boulanger-Lewandowski N, Bengio Y, Vincent P. Modeling Temporal Dependencies in High-Dimensional Sequences: Application to Polyphonic Music Generation and Transcription. Proceedings of the 29th International Conference on Machine Learning, ICML 2012;2.
21. Karan S, Zola J. Exact structure learning of Bayesian networks by optimal path extension. IEEE International Conference on Big Data 2016;p. 48–55. doi:10.1109/BigData.2016.7840588.
22. Spirtes P, Glymour C, Scheines R, et al. Constructing Bayesian Network Models of Gene Expression Networks from Microarray Data, Research Showcase @ CMU. Semantic Scholar 2018;doi:10.1184/R1/6491291.v1.
23. Chickering DM. Learning Bayesian Networks is NP-Complete, vol. 112. D F, HJ L, editors, New York, NY: Springer; 1996. ISBN: 978-1-4612-2404-4, doi:10.1007/978-1-4612-2404-4_12.
24. Chickering DM, Heckerman D, Meek C. Large-Sample Learning of Bayesian Networks is NP-Hard. Journal of Machine Learning Research 2004;5:1287–1330.
25. Cooper GF. The computational complexity of probabilistic inference using bayesian belief networks. Artificial Intelligence 1990;42:393–405. doi:10.1016/0004-3702(90)90060-D.
26. European Galaxy Server. https://usegalaxy.eu/ (2020);. Accessed 22 July 2020.
27. Jian X, Wickramarathne TL, Chawla NV. Representing higher-order dependencies in networks. Science Advances 2016;2(5). doi:10.1126/sciadv.1600028.
28. Said A, Bellogín Kouki A, de Vries AP. A Top-N Recommender System Evaluation Protocol Inspired by Deployed Systems. In: In Proceedings of the 2013 ACM RecSys Workshop on Large-Scale Recommender Systems. LSRS; 2013. .
29. Kang Z, Peng C, Cheng Q. Top-N Recommender System via Matrix Completion. In: Proceedings of the Thirtieth AAAI Conference on Artificial Intelligence (AAAI-16); 2016. .
30. Deshpande M, Karypis G. Item-Based Top-N recommender Algorithms. ACM Transactions on Information Systems 2004;22(1):143–177. doi:10.1145/963770.963776.
31. Kumar A, Tool Recommender in Galaxy using stored tool sequences. 2020;. https://github.com/anuprulez/galaxy_tool_recommendation/tree/statistical_model. Accessed 22 July 2020.
32. Kumar A, Tool recommender system in Galaxy using extra trees classifier. 2020;. https://github.com/anuprulez/galaxy_tool_recommendation/tree/sklearn_rf. Accessed 22 July 2020.
33. Smith T, Heger A, Sudbery I. UMI-tools: modeling sequencing errors in Unique Molecular Identifiers to improve quantification accuracy. Genome Research 2017;27(3):491–499. doi:10.1101/gr.209601.116.

34. Butler A, Hoffman P, Smibert P, Papalexi E, Satija R. Integrating single-cell transcriptomic data across different conditions, technologies, and species. Nature Biotechnology 2018;36(5):411–420. doi:10.1038/nbt.4096.
35. D G, A L, L K, K W, O B, N S, et al. Single-cell messenger RNA sequencing reveals rare intestinal cell types. Nature 2015;525(7568):251–255. doi:10.1038/nature14966.
36. A D, CA D, F S, et al. STAR: ultrafast universal RNA-seq aligner. Bioinformatics 2013;29(1):15–21. doi:10.1093/bioinformatics/bts635.
37. Liao Y, Smyth GK, Shi W. featureCounts: an efficient general purpose program for assigning sequence reads to genomic features. Bioinformatics 2013;30 (7):923–930. doi:10.1093/bioinformatics/btt656.
38. P E, M M, S L, M K. MultiQC: summarize analysis results for multiple tools and samples in a single report. Bioinformatics 2016;32(19):3047–3048. doi:10.1093/bioinformatics/btw354.
39. Wang L, Wang S, Li W. RSeQC: quality control of RNA-seq experiments. Bioinformatics 2012;28(16):2184–2185. doi:10.1093/bioinformatics/bts356.
40. Bolger AM, Lohse M, Usadel B. Trimmomatic: a flexible trimmer for Illumina sequence data. Bioinformatics 2014;30:2114–2120.
41. Li H. Aligning sequence reads, clone sequences and assembly contigs with BWA-MEM. ArXiv 2013 03;1303.
42. Garrison E, Marth G, Haplotype-based variant detection from short-read sequencing; 2012. https://arxiv.org/abs/1207.3907.
43. Langmead B, Trapnell C, Pop M, et al. Ultrafast and memory-efficient alignment of short DNA sequences to the human genome. Genome Biol 2009;10. https://doi.org/10.1186/gb-2009-10-3-r25.
44. Li H, Handsaker B, Wysoker A, Fennell T, Ruan J, Homer N, et al. The Sequence Alignment/Map format and SAMtools. Bioinformatics 2009;25 (16):2078–2079. doi:10.1093/bioinformatics/btp352.
45. Garrison E, A C++ library for parsing and manipulating VCF files.;. https://github.com/vcflib/vcflib. 2015. Accessed 02 August 2020.
46. Ramirez F, Ryan DP, Gruening B, Bhardwaj V, Kilpert F, Richter AS, et al. deepTools2: a next generation web server for deep-sequencing data analysis. Nucleic Acids Research 2016;44 (W1:W160–W165. doi:10.1093/nar/gkw257.
47. O'Boyle NM, Banck M, James CA, Morley C, Vandermeersch T, Hutchison GR. Open Babel: An open chemical toolbox. Journal of Cheminformatics 2011;3 (1). doi:10.1186/1758-2946-3-33.
48. Bray S, Protein-ligand docking (Galaxy Training Materials); 2020. https://training.galaxyproject.org/training-material/topics/computational-chemistry/tutorials/cheminformatics/tutorial.html, [Online; accessed 22 July 2020].
49. Ramirez F, Wolff J, Gruening B, Bhardwaj V, Ryan D, Duendar F, Deeptools/Hicexplorer: Winter Release;. https://zenodo.org/record/1133705. 2017. Accessed 02 August 2020.
50. Wolff J, Ramirez F, Bhardwaj V, Polkh E, Hi-C analysis of Drosophila melanogaster cells using HiCExplorer (Galaxy Training Materials); 2020. https://training.galaxyproject.org/training-material/topics/epigenetics/tutorials/hicexplorer/tutorial.html, [Online; accessed 22 July 2020].
51. Dündar F, Erxleben A, Batut B, Bhardwaj V, Ramirez F, Rabbani L, Formation of the Super-Structures on the Inactive X (Galaxy Training Materials); 2019. https://training.galaxyproject.org/training-material/topics/epigenetics/tutorials/formation_of_super-structures_on_xi/tutorial.html, [Online; accessed 22 July 2020].
52. Kumar A, Khanteymoori A, Introduction to deep learning (Galaxy Training Materials); 2020. https://training.galaxyproject.org/training-material/topics/statistics/tutorials/intro_deep_learning/tutorial.html, [Online; accessed 22 July 2020].
53. Kessner D, Chambers M, Burke R, Agus D, Mallick P. ProteoWizard: open source software for rapid proteomics tools development. Bioinformatics 2008;24 (21):2534–2536. doi:10.1093/bioinformatics/btn323.
54. Sigloch FC, Grüning B, Peptide and Protein ID using OpenMS tools (Galaxy Training Materials); 2020. https://training.galaxyproject.org/training-material/topics/proteomics/tutorials/protein-id-oms/tutorial.html, [Online; accessed 22 July 2020].
55. Marcel M. Cutadapt removes adapter sequences from high-throughput sequencing reads. EMBnetjournal 2011;17 (1). doi:10.14806/ej.17.1.200.
56. Batut B, Freeberg M, Heydarian M, Erxleben A, Videm P, Blank C, et al., Reference-based RNA-Seq data analysis (Galaxy Training Materials); 2020. https://training.galaxyproject.org/training-material/topics/transcriptomics/tutorials/ref-based/tutorial.html, [Online; accessed 22 July 2020].
57. Tekman M, Batut B, Erxleben A, Maier W, Pre-processing of Single-Cell RNA Data (Galaxy Training Materials); 2020. https://training.galaxyproject.org/training-material/topics/transcriptomics/tutorials/scrna-preprocessing/tutorial.html, [Online; accessed 22 July 2020].
58. Tekman M, Ostrovsky A, Downstream Single-cell RNA analysis with RaceID (Galaxy Training Materials); 2020. https://training.galaxyproject.org/training-material/topics/transcriptomics/tutorials/scrna-raceid/tutorial.html, [Online; accessed 22 July 2020].
59. Nekrutenko A, Soranzo N, Calling variants in diploid systems (Galaxy Training Materials); 2019. https://training.galaxyproject.org/training-material/topics/variant-analysis/tutorials/dip/tutorial.html, [Online; accessed 22 July 2020].
60. Batut B, et al. Community-Driven Data Analysis Training for Biology. Cell Systems 2018;6:752–758.E1. https://training.galaxyproject.org/, doi:10.1016/j.cels.2018.05.012.
61. Tsoumakas G, Katakis I. Multi-label classification: An overview. International Journal of Data Warehousing and Mining 2009;3:1–13. doi:10.4018/jdwm.2007070101.
62. Ruiz-Carmona S, Alvarez-Garcia D, Foloppe N, Garmendia-Doval AB, Juhos S, Schmidtke P, et al. rDock: A Fast, Versatile and Open Source Program for Docking Ligands to Proteins and Nucleic Acids. PLoS Computational Biology 2014;10 (4). doi:10.1371/journal.pcbi.1003571.
63. Pascanu R, Mikolov T, Bengio Y. Understanding the exploding gradient problem. ArXiv 2012;abs/1211.5063.
64. Zaremba W, Sutskever I, Vinyals O. Recurrent Neural Network Regularization. ArXiv 2014;abs/1409.2329.
65. Gal Y, Ghahramani Z. A Theoretically Grounded Application of Dropout in Recurrent Neural Networks. In: Proceedings of the 30th International Conference on Neural Information Processing Systems; 2016. p. 1027–1035.
66. Clevert D, Unterthiner T, Hochreiter S. Fast and Accurate Deep Network Learning by Exponential Linear Units (ELUs). CoRR 2015;abs/1511.07289.
67. Nair V, Hinton GE. Rectified Linear Units Improve Restricted Boltzmann Machines. In: ICML'10: Proceedings of the 27th International Conference on International Conference on Machine Learning, June 2010; 2010. p. 807–814.
68. Pedregosa F, Varoquaux G, Gramfort A, et al. Scikit-learn: Machine Learning in Python. Journal of

Machine Learning Research 2011;12:2825–2830. doi:10.5555/1953048.2078195.

69. Janocha K, Czarnecki W. On Loss Functions for Deep Neural Networks in Classification. ArXiv 2017;abs/1702.05659. doi:10.4467/20838476SI.16.004.6185.
70. Sadowski P, Notes on Backpropagation. https://www.ics.uci.edu/~pjsadows/notes.pdf (2016). Accessed 22 July 2020;.
71. Ruder S. An overview of gradient descent optimization algorithms. ArXiv 2016;abs/1609.04747.
72. Bergstra J, Yamins D, Cox DD. Hyperopt: A Python Library for Optimizing the Hyperparameters of Machine Learning Algorithms. 12th Python in science conf (SCIPY 2013) 2013;.
73. BwCluster. https://portal.bw-cloud.org/project/ (2020);. Accessed 22 July 2020.
74. Kumar A, Tool Recommender model;. https://github.com/galaxyproject/galaxy-test-data/blob/master/tool_recommendation_model.hdf5. 2020. Accessed 29 July 2020.
75. Kumar A, Get tool predictions. 2020;. https://github.com/usegalaxy-eu/galaxy/blob/release_20.05_europe/lib/galaxy/webapps/galaxy/api/workflows.py#L638. Accessed 22 July 2020.
76. Chollet F, et al. Keras 2015;.
77. Kumar A, Tool Recommender in Galaxy using GRU neural network. 2020;. https://github.com/anuprulez/galaxy_tool_recommendation. Accessed 22 July 2020.
78. Kumar A, Tool Recommender in Galaxy using CNN neural network. 2020;. https://github.com/anuprulez/galaxy_tool_recommendation/tree/cnn_wc. Accessed 22 July 2020.
79. Kumar A, Tool Recommender in Galaxy using DNN neural network. 2020;. https://github.com/anuprulez/galaxy_tool_recommendation/tree/dnn_wc. Accessed 22 July 2020.
80. Kumar A, Output results files. 2020;. https://github.com/anuprulez/galaxy_tool_recommendation/tree/master/output_files/data_20_05. Accessed 22 July 2020.
81. Kumar A, Tool Recommender model creator;. https://usegalaxy.eu/root?tool_id=toolshed.g2.bx.psu.edu/repos/bgruening/create_tool_recommendation_model/create_tool_recommendation_model/0.0.3. 2020. Accessed 22 July 2020.
82. Kumar A, Tool Recommender overwrite;. https://github.com/usegalaxy-eu/galaxy/blob/release_20.05_europe/config/tool_recommendations_overwrite.yml.sample. 2020. Accessed 30 July 2020.
83. Kumar A, Rasche H, Gruening B, Backofen R, Supporting data for "Tool recommender system in Galaxy using deep learning" GigaScience Database; 2020. http://dx.doi.org/10.5524/100838.

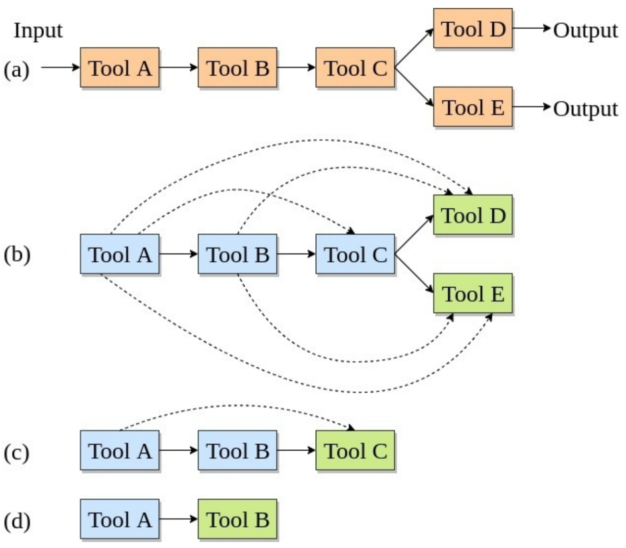

Figure 1. An example workflow (a) is shown consisting of 5 different tools which is decomposed into multiple tool sequences shown in (b), (c) and (d). Each tool sequence shows higher-order dependencies where a tool is dependent on all of its prior tools. These dependencies are shown by the dashed arrows.

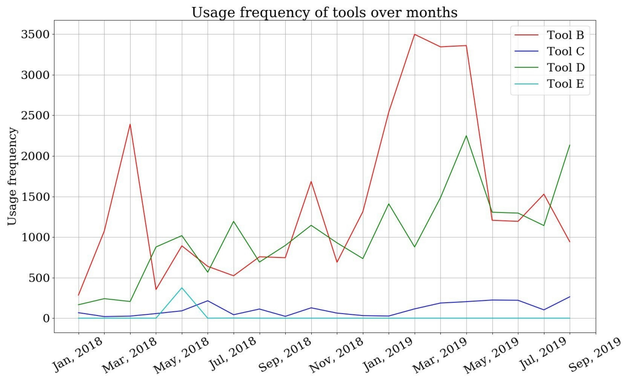

Figure 2. The plot shows the usage frequencies of 4 tools collected over the past one year. The Tools B and D have high usage frequencies almost every month while the Tools C and E have much lower usage frequencies compared to Tools B and D. The tool A is absent from the plot because it is not the label of any tool for the workflow shown in Figure 1.

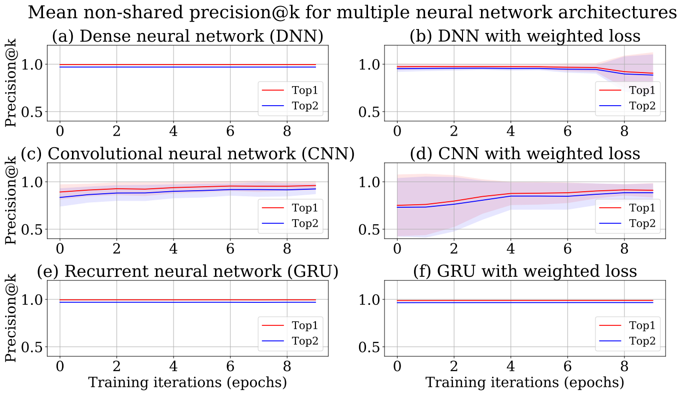

Figure 3. The subplots (a), (c) and (e) show top-k (precision@k) non-shared precision for DNN, CNN and GRU neural networks with cross-entropy loss function, respectively. The subplots (b), (d) and (f) show top-k (precision@k) non-shared precision for DNN, CNN and GRU neural networks with weighted cross-entropy loss function, respectively.

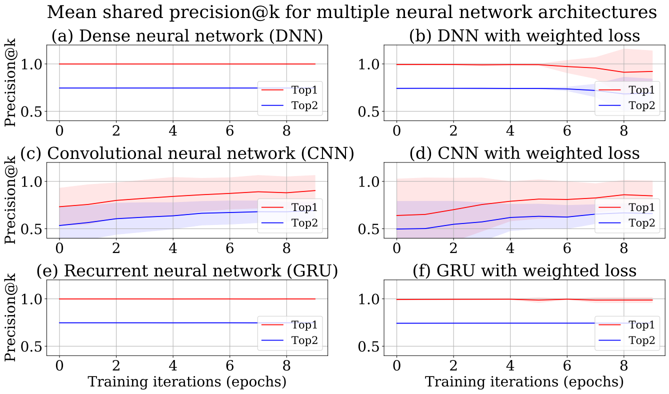

Figure 4. The subplots (a), (c) and (e) show top-k (precision@k) shared precision for DNN, CNN and GRU neural networks with cross-entropy loss function, respectively. The subplots (b), (d) and (f) show top-k (precision@k) shared precision for DNN, CNN and GRU neural networks with weighted cross-entropy loss function, respectively.

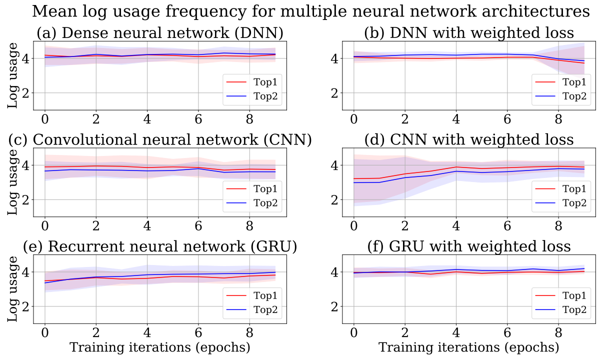

.

Figure 5. The subplots (a), (c) and (e) show usage frequencies of (top-k) predicted tools for DNN, CNN and GRU neural networks with cross-entropy loss function, respectively. The subplots (b), (d) and (f) show usage frequencies of (top-k) predicted tools for DNN, CNN and GRU neural networks with weighted cross-entropy loss function, respectively.

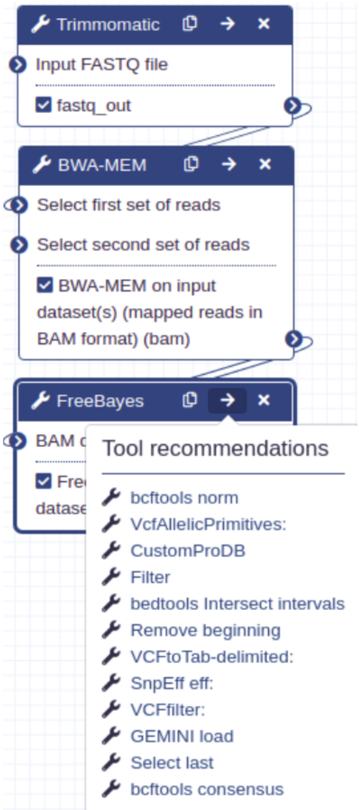

Figure 6. The figure shows recommended tools, listed in a dropdown with "Tool recommendations" as its header, in the workflow editor of the European Galaxy server for "Trimmomatic → BWA-MEM → FreeBayes" tool sequence. The recommended tools for the tool sequence can be seen in a dropdown while hovering on the right arrow button placed in the top-right corner of the "FreeBayes" tool. Clicking on any recommended tool such as "bcftools norm" in the dropdown opens a new block for the chosen tool which can be connected to the tool sequence.

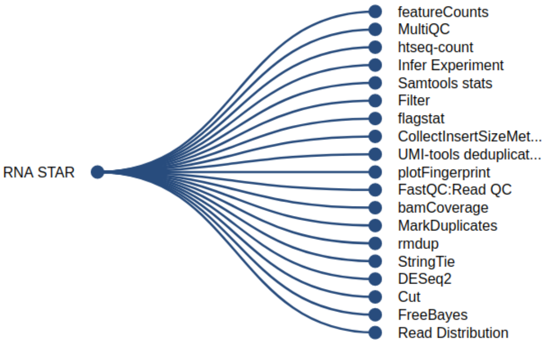

Figure 7. The figure shows recommended tools as leaves (on the right) of the tree after executing RNA-STAR tool. Clicking on any recommended tool opens its definition in Galaxy and can be used for further analysis with the data files produced by the previous tool (RNA-STAR).

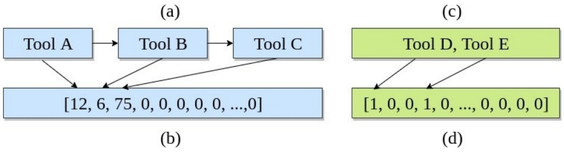

Figure 8. The figure shows how a tool sequence and its labels are transformed into vectors.

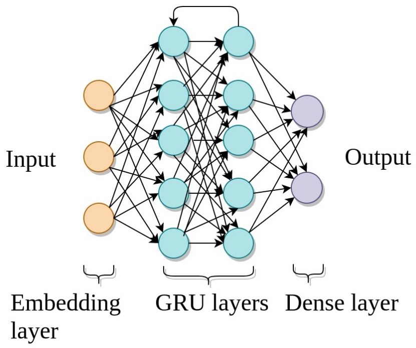

Figure 9. The figure shows the architecture of the GRU neural network. It has four components as layers. The first layer is the input layer (yellow), two stacked layers of GRU (cyan) and the last layer is the output layer (violet). The dropout layers are added between embedding and GRU layers, between two GRU layers and between the second GRU and dense layers.

Manuscript
Click here to access/download;Manuscript;GigaScience_Tool_Recommend

GigaScience, 0000, 1–12

doi: xx.xxxx/xxxx
Manuscript in Preparation
Technical Note

TECHNICAL NOTE

Tool recommender system in Galaxy using deep learning

Anup Kumar[1,*,†], Helena Rasche[1, ‡, †], Björn Grüning[1, §, †] and Rolf Backofen[1,2, ¶, †]

[1]Bioinformatics Group, Department of Computer Science, University of Freiburg, Georges-Koehler-Allee 106, 79110 Freiburg, Germany and [2]Signalling Research Centres BIOSS and CIBSS, University of Freiburg, Schaenzlestr. 18, 79104 Freiburg, Germany

*kumara@informatik.uni-freiburg.de https://orcid.org/0000-0002-2068-4695
‡helena.rasche@gmail.com https://orcid.org/0000-0001-9760-8992
§gruening@informatik.uni-freiburg.de https://orcid.org/0000-0002-3079-6586
¶backofen@informatik.uni-freiburg.de https://orcid.org/0000-0001-8231-3323
†Contributions follow the order of the names of authors

Abstract

Background Galaxy is a web-based and open-source scientific data-processing platform. Researchers compose pipelines in Galaxy to analyse scientific data. These pipelines, also known as workflows, can be complex and difficult to create from thousands of tools, especially for researchers new to Galaxy. To help researchers with creating workflows, a system is developed to recommend tools that can facilitate further data analysis. **Findings** A model is developed to recommend tools using a deep learning approach by analysing workflows composed by researchers on the European Galaxy server. The higher-order dependencies in workflows, represented as directed acyclic graphs, are learned by training a gated recurrent units (GRU) neural network, a variant of a recurrent neural network (RNN). In the neural network training, the weights of tools used are derived from their usage frequencies over time and the sequences of tools are uniformly sampled from training data. Hyperparameters of the neural network are optimised using Bayesian optimisation. Mean accuracy of 98% in recommending tools is achieved for the top-1 metric. **Conclusions** The model is accessed by a Galaxy API to provide researchers with recommended tools in an interactive manner using multiple user interface (UI) integrations on the European Galaxy server. Good quality and highly-used tools are shown at the top of the recommendations. The scripts and data to create the recommendation system are available under MIT license at https://github.com/anuprulez/galaxy_tool_recommendation.

Key words: Recommender system; Galaxy; Workflows; Deep learning; Neural networks; Gated recurrent units

Findings

Background

Life sciences depend increasingly on high-throughput data, turning them into data science to a large extent. However, raw high-throughput data does not have much value on its own without proper analysis and interpretation of the data. To simplify the data analysis process and to ensure a reproducible analysis, several workflow systems such as Bcbio-nextgen, Omics Pipe, Nextflow, Luigi, Toil and many others have emerged [1, 2, 3]. The main idea for workflow systems is based on the observation that any computational analysis of high-throughput data encompasses multiple steps such as quality control, preprocessing, quantification and statistical analysis to transform raw data into scientific results. Collec-

Compiled on: November 25, 2020.
Draft manuscript prepared by the author.

tively, these steps form a workflow where each step performs a definite transformation of the data, which can be performed using standardised tools. Using workflow for the analysis is simple and convenient and has several advantages. First, it is easy to replace individual tools by a newer version or to assess the influence of the associated step on the final result. Second, a workflow can be saved, shared and reused, which ensures reproducible research. Therefore, workflows are becoming essential in the analysis of scientific data and there are multiple platforms where researchers can create workflows for their analyses. However, a critical question is how to assess whether a generated workflow is state-of-art or even valid at all. To give a concrete example, one can use several real-valued input vectors (such as fluorescence-based measurement stemming from arrays), transform them into integer-based values in the first step and combine it with a tool that uses a count-based statistics (such as negative binomial distribution as used in DESeq2 [4]) to determine values that show high differential behaviour. While this workflow would run on a workflow system without problems and even produce some results, the generated results are not valid because of the wrong statistical model. Therefore, it is important to use a tool for each step in a workflow which can bring desired results. To make it possible, a system is needed which can recommend useful tools at each step while creating a workflow.

Galaxy and workflows

Galaxy is an open-source data processing platform which enables researchers to create and store their workflows for multiple scientific analyses [5]. A workflow in Galaxy is a directed acyclic graph and consists of one or many tool sequences to analyse scientific data such as DNA and RNA sequences. A tool consumes one or more data files as input and produces one or more data files as output and supports a number of formats of these input and output files. In workflows, the tools are connected one after another following a constraint that the adjacent tools must have compatible data types. In other words, the data types of output files of a tool should match the data types of input files of the following tool. Galaxy has thousands of accessible tools and acquiring familiarity and constructing workflows with these tools can be a complex and time-consuming task, especially for researchers new to Galaxy. To assist them in creating workflows and making them aware of the possible tools for further analyses, a recommender system is devised. The benefits of having such a system are manifold. First, it will avoid the loss of time spent in creating erroneous or less optimal workflows by choosing tools which may produce undesired results and thereby making researchers more efficient. Second, it will help researchers bypass the step of searching for tools separately, which will further reduce the time spent in creating workflows and at the same time increase the accessibility of tools. Third, it will promote good quality tools having higher usage frequencies in the past (last one year) to the top of the recommendations and downgrade those having lower usage frequencies to the bottom of the recommendations. It is achieved by assigning weights to tools which are derived from their usage frequencies over a period of time. Finally, it can be extended to promote the newly added tools in Galaxy by showing them alongside the recommended tools predicted using the neural network approach.

Recommender systems

The objective of having recommender systems in fields such as scientific literature search, online shopping, travel bookings, media-service providers and many other fields is to help people discover suitable, interesting and newly-released products. These recommended products are recognised based on the usage and purchasing patterns of people in the past. In the field of scientific literature search, the exponential increase in the number of published papers necessitates having a recommender system to help scientists explore relevant and recent papers quickly [6, 7, 8]. Recommender systems are significant in the world of commercial applications too. Companies such as Amazon and Netflix have appropriately used them to learn preferences of their respective customers in selecting products such as their favourite books or movies and to propose a few products out of a large catalogue. By enabling users and customers to discover reasonable and customised products, recommender systems have helped them grow as organisations [9, 10]. In short, recommender systems make it faster for users and customers to look through a few recommended products to find the most suitable ones. These successful implementations of recommender systems by organisations across the world working in diverse areas to assess the needs of their respective users in proposing relevant products motivated us to create a tool recommender system in Galaxy.

Related work

To simplify creating workflows for scientific analyses, a few approaches have been proposed which suggest alternative tools and workflows. EDAM (EMBRACE Data And Methods) and semantic annotations of tools are used to compose workflows automatically for mass-spectrometry based proteomics [11]. The annotations include the names, functionalities, input and output data types of tools. The PROPHETS (Process Realisation and Optimisation Platform using Human-readable Expression of Temporal-logic Synthesis) program generates suitable candidates of workflows which match the goal of the proposed workflow and its annotations [12]. WINGS (Workflow INstance Generation and Specialization) offers multiple variations of a workflow created using different tools. It makes use of the input parameters, types of datasets and functions of tools to build the variations [13, 14]. The approach used in [15] utilises data types to facilitate the automatic creation of workflows. All these approaches depend either on annotations or matching input and output data types of adjacent tools in workflows and they pose challenges such as the addition and maintenance of the meaningful annotations of tools and extracting input and output data types of adjacent tools. Moreover, these approaches have their workflow generation restricted to a few specific bioinformatics analyses such as proteomics or proteogenomics. In addition, they do not discuss the presence of higher-order relationships [16] in tool sequences of workflows. Our approach to recommend tools in workflows aims to overcome these challenges in the following manner. First, it does not require collecting and storing information about tools. Second, it takes into account the higher-order relationships among tools (Figure 1) in tool sequences. Finally, it incorporates workflows from multiple scientific analyses to produce the recommender system.

Sequential learning on workflows

Workflows, created by many researchers in Galaxy for different scientific analyses, are decomposed into numerous tool sequences (Figure 1). The sequential nature of these tool sequences where tools are connected one after another inspires us to apply similar learning techniques used for other sequential data such as text and speech. There are multiple studies in the fields of natural language processing, clinical research and speech recognition which apply deep learning techniques on se-

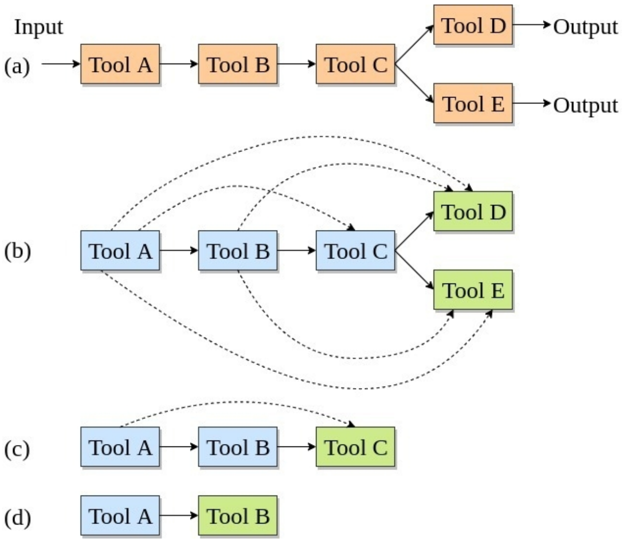

Figure 1. An example workflow (a) is shown consisting of 5 different tools which is decomposed into multiple tool sequences shown in (b), (c) and (d). Each tool sequence shows higher-order dependencies where a tool is dependent on all of its prior tools. These dependencies are shown by the dashed arrows.

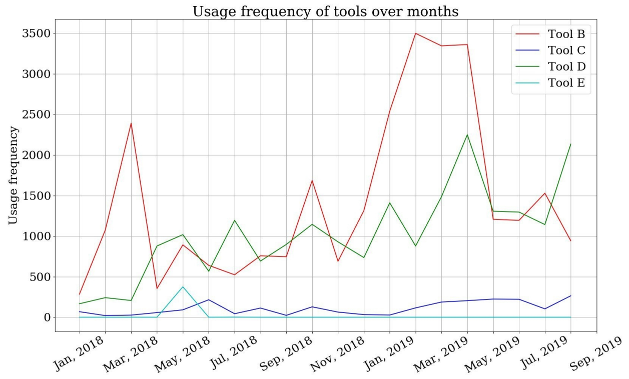

Figure 2. The plot shows the usage frequencies of 4 tools collected over the past one year. The Tools B and D have high usage frequencies almost every month while the Tools C and E have much lower usage frequencies compared to Tools B and D. The tool A is absent from the plot because it is not the label of any tool for the workflow shown in Figure 1.

quential data to obtain good accuracy in predicting future items. The approach used in [17] finds context in long sequences of words for sentiment analysis and part-of-speech tagging using recurrent neural network (RNN) and achieves 85% and 93% accuracy, respectively. For clinical data, learning on long sequences of health states proves to be beneficial [18]. The health states of patients recorded at different time points are analysed by accessing their electronic health records. The future health states of patients could be predicted by training RNN on the sequences of their past health states to achieve 85% accuracy. Moreover, the variants of RNN are used to model speech and music signals [19, 20]. These successful studies benefit from sequential learning techniques using different variants of RNN. Therefore, in our work, a variant of RNN - gated recurrent units (GRU) - is used to create the tool recommender system in Galaxy.

A Bayesian network can also be used for modelling directed acyclic graphs (workflows) [21, 22]. It requires computing joint and conditional probabilities of nodes in graphs and an increase in the number of nodes can lead to a higher cost to compute these probabilities. In addition, making predictions by learning a probabilistic network is a hard problem [23, 24, 25]. Because of these drawbacks of using a Bayesian network, it is not used in our approach to create the recommender system in Galaxy.

Data description

Over 18,000 workflows from different scientific analyses such as RNA-seq, Variant-calling, Hi-C, Assembly, Single-cell, Proteomics and so on in the European Galaxy server [26] have been used to create the recommender system. A workflow consisting of 5 tools is shown in Figure 1a. It is divided into smaller tool sequences as shown in Figure 1b, 1c and 1d. The last tool, shown in green, of each tool sequence (of length n) is assigned as the label of the sub-sequence (of length n-1) shown in blue in Figure 1. A label is an output which is learned and predicted by the recommender system. In the neural network learning, a tool is a label. For example, in Figure 1b, Tools D and E are the labels of the sub-sequence Tool A $\rightarrow$ Tool B $\rightarrow$ Tool C. They show higher-order dependencies in their connections which implies that a tool is not only dependent on its immediate predecessor but also on all prior tools in the tool sequence. For example, in Figure 1c, the Tool C is dependent on Tools B and A. By analysing multiple workflow fragments in this way, the neural network learns that the label of a tool sequence Tool A $\rightarrow$ Tool B is Tool C. It is expected that dividing a tool sequence into fragments with a minimum length of two tools, as shown in Figure 1c and 1d, will improve the generalisation performance of the neural network because it gets more tool sequences with a variety of lengths to learn from. The dependencies shown in Figure 1b, 1c and 1d present in tool sequences are learned using the GRU neural network by modelling the conditional probability given by Equation 1 [27]. Using the approach explained above, more than 229,000 tool sequences are extracted.

Usage pattern of tools

Tools in Galaxy have different usage patterns. Some tools are used more often than other tools for multiple reasons such as differences in their functions and availability of similar but better tools. It is essential to analyse the usage patterns of tools because the recommender system proposes tools for researchers and these tools should have high relevance to their analyses. One of the key indicators of the relevance of tools can be their high usage frequencies. If a tool has been used often in the recent past, it implies that the tool is relevant. However, if a tool was used often a few years ago but has been used less often in the last six months then the relevance of that tool has certainly declined. The usage frequencies of tools (shown as labels in Figure 1) over the past year are shown in Figure 2.

Shared and non-shared workflows

The set of workflows may have poor quality workflows which are non-published or deleted or may have errors in their tool connections. To distinguish between good and poor quality tool connections, the labels for each tool sequence are divided into two parts - shared and non-shared labels. The shared labels come from the published, non-deleted and non-erroneous workflows while the non-published labels come from other workflows. While recommending tools for a tool or tool sequence, the shared labels are promoted to the top of the recommendations if available followed by the non-shared labels. This enables good quality tools to be shown as the top recommendations.

Imbalance in workflows

Tool sequences from these workflows may vary in number - some may occur more frequently and others may not. There-

fore, the complete set of tool sequences may not be equally representative of all workflows coming from different scientific analyses. Learning on the imbalanced set of tool sequences can induce bias which may have an undesired outcome - good accuracy in recommending tools coming from highly frequent tool sequences and poor accuracy for tools coming from less frequent ones. To mitigate this bias, all tool sequences are chosen with uniform frequency while training the neural network which allows it to have a comparable accuracy in recommending tools in different scientific analyses. This uniform sampling strategy is discussed in the "Implementation" section in detail.

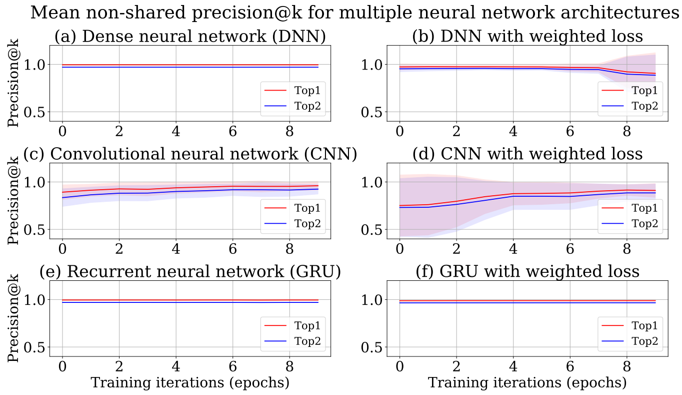

Figure 3. The subplots (a), (c) and (e) show top-k (precision@k) non-shared precision for DNN, CNN and GRU neural networks with cross-entropy loss function, respectively. The subplots (b), (d) and (f) show top-k (precision@k) non-shared precision for DNN, CNN and GRU neural networks with weighted cross-entropy loss function, respectively.

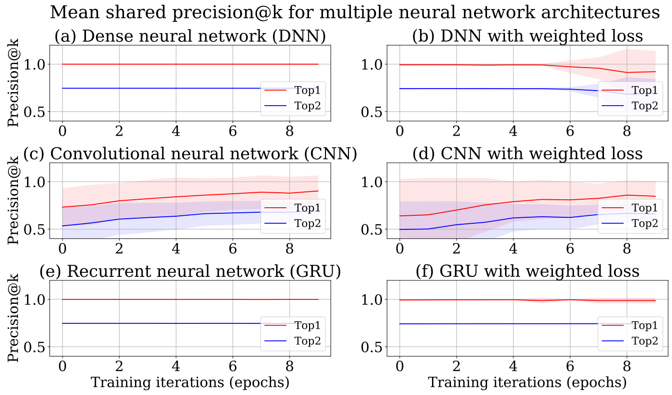

Figure 4. The subplots (a), (c) and (e) show top-k (precision@k) shared precision for DNN, CNN and GRU neural networks with cross-entropy loss function, respectively. The subplots (b), (d) and (f) show top-k (precision@k) shared precision for DNN, CNN and GRU neural networks with weighted cross-entropy loss function, respectively.

Results

Three different neural network architectures - dense neural network (DNN), convolutional neural network (CNN) and gated recurrent units neural network (GRU) - are compared on their performances in predicting tools (Figures 3 and 4). The models obtained after training all the neural network architectures are used to predict tools for the tool sequences in the test data after every training iteration. Top-k precision (precision@k) is a popular metric for evaluating a recommender system [28, 29, 30]. Precision@k implies how many in the k predicted tools are correct. The correctness here refers to the compatibility of the predicted tools with the tool for which predictions have been made. For example, $k = 2$ implies that the number of predicted tools are 2 with the highest predicted scores. If only 1 of them is correct, then the precision@2 is $\frac{1}{2} = 0.5$. In this way, precision@1 and precision@2 are computed for all the tool sequences in the test data and then averaged. Precision@1 and precision@2 metrics are used in this approach to evaluate the quality of the tool recommender system. The precision of recommended tools are computed separately for the non-shared and shared recommendations and shown in different plots (Figures 3 and 4) but for usage frequencies, they are combined into one plot (Figure 5). The precision and usage frequencies of the predicted tools for the precision@1 and precision@2 (top-1 and top-2) metrics are computed over 10 training iterations for each experiment run. They are averaged and their respective standard deviations are computed over 10 experiment runs. Mean precision and usage frequencies are shown by the respective line plots and the shaded regions span the area between one standard deviation above and below the mean (Figures 3, 4 and 5).

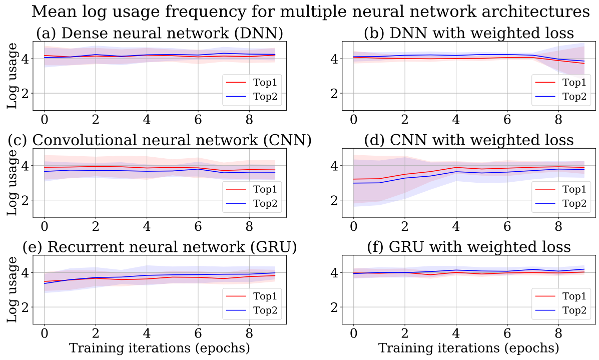

.

Figure 5. The subplots (a), (c) and (e) show usage frequencies of (top-k) predicted tools for DNN, CNN and GRU neural networks with cross-entropy loss function, respectively. The subplots (b), (d) and (f) show usage frequencies of (top-k) predicted tools for DNN, CNN and GRU neural networks with weighted cross-entropy loss function, respectively.

Comparison of GRU neural network with other approaches

The GRU neural network with the weighted cross-entropy loss function shows superior performance to CNN (Figures 3d, 3f, 4d and 4f) by achieving 98% top-1 non-shared and shared precision which proves that the GRU layers in a neural network are better for learning on tool sequences than the convolutional layer. Moreover, it shows a lower divergence in non-shared and shared precision and usage frequencies (Figures 3d, 3f, 4d, 4f, 5d and 5f) establishing that its predictive strength is more stable than CNN over multiple experiment runs. Surprisingly, the weighted cross-entropy loss function does not have any beneficial effect on the CNN architecture as its non-shared and shared precision and usage frequencies show higher divergence over multiple experiment runs (Figures 3c, 3d, 4c, 4d, 5c and 5d). Therefore, CNN is not used in our approach. In contrast to CNN, DNN achieves a similar non-shared and shared precision to the GRU neural network with a small divergence (Figures 3a, 3b, 3f, 4a, 4b and 4f). However, due to higher divergence in accumulated usage frequencies, it is not used in our approach (Figures 5a, 5b and 5f). Weighted cross-entropy loss function

in the GRU neural network (Figure 5f) drives it to classify tools more robustly with higher usage frequencies. In other words, it predicts tools with higher precision than CNN and lower divergence in usage frequencies than DNN. Therefore, it is used in our approach to learn on tool sequences and recommend tools.

To compare the performance of the GRU neural network with approaches which do not use any neural network, two ideas are explored. The first approach simply stores all the sequences of tools [31] formed using the technique shown in Figure 1 to create a model. To recommend tools using this model, all the tool sequences are searched for a given tool or a sequence of tools. The second approach uses ExtraTrees classifier [32] to recommend tools. These approaches are discussed in section S1 of the supplementary document in detail.

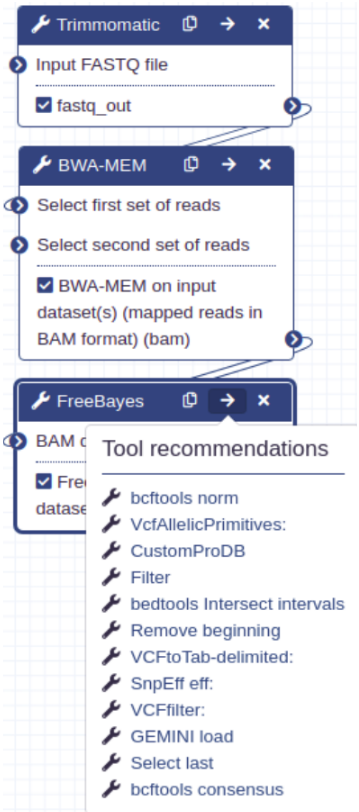

Figure 6. The figure shows recommended tools, listed in a dropdown with "Tool recommendations" as its header, in the workflow editor of the European Galaxy server for "Trimmomatic → BWA-MEM → FreeBayes" tool sequence. The recommended tools for the tool sequence can be seen in a dropdown while hovering on the right arrow button placed in the top-right corner of the "FreeBayes" tool. Clicking on any recommended tool such as "bcftools norm" in the dropdown opens a new block for the chosen tool which can be connected to the tool sequence.

Benefit of regularisation

Using regularisation minimises overfitting by assisting the GRU neural network to make better recommendations by predicting tools having low usage frequencies but useful in addition to tools with high usage frequencies. For example, the recommendations of "UMI-tools count" [33] tool with the regularised model include "Seurat" [34] tool which is absent from recommendations by the non-regularised model. Another example is for "RaceID, Lineage computation using StemID" tool sequence which gets "Lineage Branch Analysis using StemID" [35] tool as one of the recommendations by the regularised model while there is no recommendation at all by the non-regularised model. The recommendations for a popular mapper, RNA-STAR [36], are "featureCounts" [37], "MultiQC" [38], "Infer Experiment" [39] and few others by both the models. But, in addition to these recommendations, the regularised model recommends the "Read Distribution" [39] tool which is not predicted by the non-regularised model. More details have been provided in section S4 of the supplementary document.

Examples of tool recommendations

To illustrate the real time usage of the recommender system in the European Galaxy server, two examples have been provided. The first one shows recommended tools for a tool sequence with 3 tools, Trimmomatic [40] → BWA-MEM [41] → FreeBayes [42], in the workflow editor of the European Galaxy server (Figure 6). Trimmomatic is used to trim the sequencing data such as DNA and RNA sequences. One of the useful analyses after trimming the sequences is to map them on a reference genome using a mapper. Several mappers such as BWA-MEM [41], Bowtie2 [43] and RNA-STAR [36] are predicted. BWA-MEM is chosen from the predicted mappers and connected to Trimmomatic. After mapping, for further analysis of mapped sequences, many tools are predicted such as MultiQC for summarising the quality of mapping, featureCounts for counting the reads mapped to different regions on the genome or FreeBayes for detecting variants and a few others. FreeBayes is chosen and a list of recommendations is shown as a dropdown containing tools such as "bcftools norm" [44], VcfAllelicPrimitives [45] and many others for Trimmomatic → BWA-MEM → FreeBayes tool sequence. Another example of tool recommendations after using RNA-STAR is shown in Figure 7. It shows follow-up tools such as bamCoverage [46] for calculating read coverage, MultiQC, featureCounts and a few others. In summary, the tool recommendations provide useful knowledge about tools to Galaxy users and researchers to continue multiple scientific analyses.

Table 1 lists a few shared and non-shared recommended tools for multiple tool sequences in different scientific analyses such as Computational chemistry, Epigenetics, Machine learning, Proteomics, RNA sequencing and a few others. The recommended tools shown in this table are highly used for standard scientific analyses as highlighted in multiple GTN training materials [60].

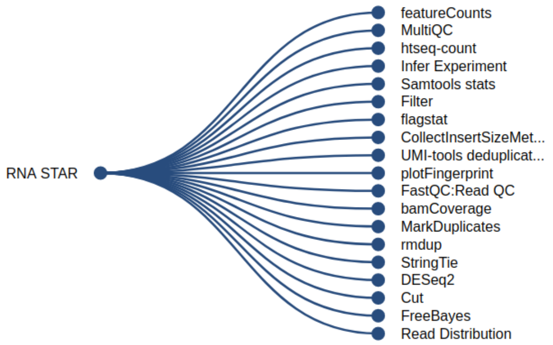

Figure 7. The figure shows recommended tools as leaves (on the right) of the tree after executing RNA-STAR tool. Clicking on any recommended tool opens its definition in Galaxy and can be used for further analysis with the data files produced by the previous tool (RNA-STAR).

Implementation

To create a tool recommender system in Galaxy, workflows are collected from the European Galaxy server. A workflow may

Table 1. Shared and non-shared recommendations for tool sequences in different scientific analyses

Scientific analyses	Tool/Tool sequences	Recommended tools (shared)	Recommended tools (non-shared)
Computational chemistry	Molecule to fingerprint [47]	Taylor-Butina clustering, NxN Clustering [48], Similarity Search	-
Epigenetics	hicBuildMatrix [49]	hicSumMatrices	hicMergeMatrixBins, hicPlotMatrix [50], hicPCA, hicTransform
Epigenetics	multiBamSummary [46]	plotCorrelation [51]	plotPCA
Epigenetics	Bowtie2	MASC2 CallPeak [51]	bamCoverage, FreeBayes
Machine learning	Create a deep learning model architecture	-	Create deep learning model [52], Build Deep learning Batch Training Models
Proteomics	Msconvert [53]	Search GUI, FlashLFQ	PeakPickerHiRes [54]
RNA Sequencing	Cutadapt [55]	FastQC, RNA-STAR, MultiQC [56]	Bowtie2, Hisat2, BWA-MEM
RNA Sequencing	Cutadapt [55], RNA-STAR	featureCounts, MultiQC, Infer Experiment [56]	bamCoverage, RmDup
Single-cell	UMI-tools extract [33]	RNA-STAR [57]	Bowtie2, Hisat2, BWA-MEM, UMI-tools group
Single-cell	Initial processing using RaceID [35]	Clustering using RaceID [58]	-
Single-cell	Initial processing using RaceID, Clustering using RaceID [35]	Cluster Inspection using RaceID [58], Lineage computation using StemID	-
Variant-calling	FreeBayes	VcfAllelicPrimitives [59]	Gemini load
Variant-calling	FreeBayes,VcfAllelicPrimitives	SnpSift Filter, VT normalize	SnpEff eff [59]

have one or many tool sequences where tools are connected one after another. Tool sequences are transformed into matrices and produced as input to a GRU neural network to learn patterns in the connections of tools.

$$p(x_T|x_1, x_2, ..., x_{T-1}) \quad (1)$$

The probability of a tool (x_T) is estimated given all other prior tools (x_1, ..., x_{T-1}) for a tool sequence (x_1, ..., x_{T-1}, x_T). Neural network learning is classification because there are labels for tool sequences which are learned and then predicted. Moreover, the classification is multi-class (multiple tools as labels) and multi-label (multiple tools as labels for a tool sequence) [61]. To ensure an unbiased learning and evaluation by the neural network, the set of tool sequences is divided into two parts - training and test. The training data is used for learning a model and the test data is used for evaluating the model.

Uniform sampling

Workflows in Galaxy come from different scientific analyses. It may happen that the number of workflows from these analyses are not comparable - some analyses may have a large number of workflows while some may have only a small number of workflows. This can cause some tools to be present very frequently in workflows while other tools are less frequent. Learning on these workflows and recommending tools may exhibit bias by showing better recommendations for the frequently occurring tools and poorer recommendations for the less frequent ones. To showcase this imbalance, the frequencies of the last tool in each tool sequence in training data are calculated and it is found that only a few tools have large frequencies and most of the tools are present in low frequencies (Supplementary Figure 3). For example, the tools with very high frequencies (> 10,000) are “Concatenate datasets”, “Cut”, “Grouping” and “Join” while the tools having very low frequencies (< 5) are “Cluster inspection using RaceID”, “rDock cavity definition” [62] and “ChiRA collapse”. Therefore, to overcome this drawback, the training data created after extracting tool sequences should be balanced to make the neural network learn on a similar number of tool sequences from different scientific analyses in each training iteration. To implement this strategy, a set of last tools in all tool sequences from the training data is collected. Further, for each tool in this set, a list of indices of tool sequences in the training data are stored for which it is the last tool (Supplementary Table 3). Only the last tools are considered for implementing this strategy because of two reasons. First, the smallest tool sequences contain only two tools and second, all tools become the last tool in at least one

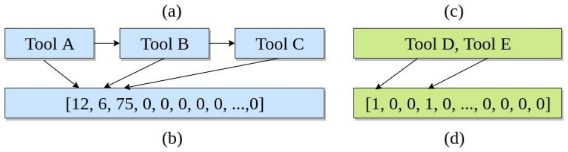

Figure 8. The figure shows how a tool sequence and its labels are transformed into vectors.

tool sequence and the computed frequencies of these last tools suggest their overall frequencies in the training data.

In the neural network training, for each iteration (which consumes all tool sequences in the training data), small batches containing an equal number of tool sequences are created. For example, if the batch size is 100 and the size of training data is 2000, then 20 (2000/100=20) batches are created, each containing 100 tool sequences. In each batch, 100 tools from the set of last tools are uniformly selected (Column 2 in Supplementary Table 3) and for each selected tool, a tool sequence is chosen uniformly from its respective list of tool indices (Column 3 in Supplementary Table 3). After selecting tool sequences for many batches for each iteration of training (epoch), it is expected that all the tools from the set of last tools and their respective tool sequences are chosen. Performing this uniform selection of different tool sequences for each iteration, the training data becomes balanced. Supplementary Figure 4 shows that each last tool is present approximately 1670 times on an average in each iteration (epoch). The order of tools in the Supplementary Figures 3 and 4 are same.

Data transformation

Tool sequences extracted from workflows are transformed into vectors because neural networks require input data to be represented as vectors and matrices. Each tool sequence has one or more labels (Figure 1) and they are transformed into different vectors - a tool sequence vector (Figure 8b) and a label vector (Figure 8d). To form these vectors, a dictionary of tools is needed which stores an index for each tool. Using the indices of tools, a tool sequence vector is created preserving the original order of tools as in the tool sequence. For example, Tool A has an index of "12" in the dictionary, therefore it is replaced by "12" in the vector (Figure 8b). The vector is padded with trailing zeros to keep the length of the vector same across the varying lengths of tool sequences. The size of this vector is 25 which means that a tool sequence can have a maximum of 25 tools. The tool sequences larger than this size are discarded. The labels (Figure 8c) are transformed into a bit vector (Figure 8d) in which the positions, stored as indices in the dictionary of tools, of the labels (tools) are turned "on" (set to 1) specifying that these tools are the labels of the tool sequence and others are not (set to 0). It has the same size as the dictionary of tools. In the machine learning field, it is also known as a multi hot-encoded vector. Together, these two vectors form a training sample for the neural network. A pair of vectors are created in this manner for each tool sequence and for all the tool sequences, they are combined to form two matrices - one for tool sequences and another for their respective labels. Internally, the label vectors have two subsets, one for shared labels (from the published, non-deleted and non-erroneous workflows) and another for non-shared labels (from the rest of the workflows). These matrices form input data to the neural network.

Neural network architecture

GRU neural network, a variant of RNN, is used for creating a model to recommend tools. The neural network architecture has multiple components such as different layers (Figure 9),

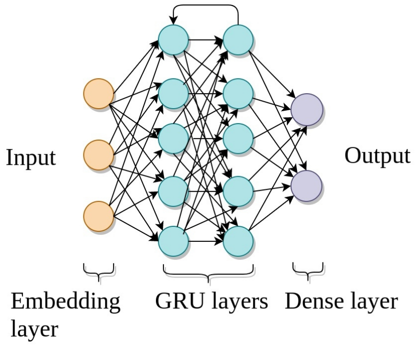

Figure 9. The figure shows the architecture of the GRU neural network. It has four components as layers. The first layer is the input layer (yellow), two stacked layers of GRU (cyan) and the last layer is the output layer (violet). The dropout layers are added between embedding and GRU layers, between two GRU layers and between the second GRU and dense layers.

activation functions, class weights, loss function, hyperparameter tuning technique which are discussed in detail in the following paragraphs.

Embedding layer. The first component of the neural network architecture is an input layer (Figure 9) which learns an embedding, a fixed-size vector, for each tool. This vector is used by the neural network as an internal representation of a tool. The embedding vector replaces the indices of tools in each tool sequence. The size of the embedding vector is fixed for all tools. For example, the vector of a tool sequence [12, 6, 75, 0, 0, ..., 0] is transformed into [[0.3, 0.01, 0.003, ..., 0.23], [0.5, 0.1, 0.005, ..., 0.9], [...], 0, 0, ...,0] by the embedding layer. The same embedding vector represents a tool in all tool sequences in which the tool is present.

GRU layer. The stacked layers of GRU learn deeper structures in the tool sequences by modelling the conditional probabilities of tools (labels) given all other prior tools (Figure 9). GRU has certain advantages which help it to learn on sequential data. First, it avoids the problems of vanishing and exploding gradients which commonly occur in traditional RNN [63]. It is important because learning higher-order dependencies depends on the gradients of errors concerning the parameters (recurrent and input weight matrices) of GRU layers. Second, GRU has slightly fewer parameters than the long short-term memory network (LSTM), another variant of RNN, which makes using GRU simpler than LSTM. Finally, it achieves similar accuracy as the LSTM [19].

Output layer. The last component of the neural network architecture is a dense layer which computes predictions (Figure 9). The dimension of this layer is equal to the number of unique tools because it predicts a score for each tool (label). The predicted score of each tool is considered as its probability of being the label of an input tool sequence. The closer the predicted score of a tool is to 1, the more probable it is to be the recommended tool and the closer it is to 0, the less probable it is to be the recommended tool.

Dropout layer. Overfitting happens when a neural network performs exceptionally well on the training data but its performance on test (unseen) data remains poor. To minimise the effect of overfitting, a dropout layer is used between two layers of the neural network. It sets a few randomly chosen connections to 0 in the neural network to introduce some randomness to minimise overfitting [64, 65]. 3 dropout layers are used in our approach - one between the embedding and the first GRU

layers, one between 2 GRU layers and the last one between the second GRU and dense layers.

Activations. These are mathematical functions which are used in neural networks to transform inputs to a layer into its outputs. Two activations are used in our approach - one is exponential linear units (ELU) [66] and another is sigmoid (Equation 2). ELU is used for both the GRU layers and has a special feature of being negative when the input is negative which allows mean activation (output) to get closer to 0 compared to other activation functions such as ReLU [67] which is always positive. As mean activations get closer to 0, the approximated and actual gradients get closer to each other. Therefore, using ELU in our neural network as an activation can be useful to achieve faster training, an increased drop in loss and better accuracy. Sigmoid is used in the output layer which normalises any real number to lie between 0 and 1 and it is considered as a probability of each tool.

$$f(x) = \frac{1}{1 + e^{-x}} \quad (2)$$

Usage frequencies of tools as weights. To incorporate the usage frequency of tools in the recommender system, the usage frequencies of all the tools used in the last one year have been collected and are used in the neural network training as the weights of tools. A tool which has been used often (for example Tool B in Figure 2) in the past one year is assigned a higher weight than a tool (for example Tool C in Figure 2) which has been used less often in the past one year. When tools are recommended a score is assigned to each tool by the neural network. It is expected that a tool with a higher weight gets a higher score and a tool with a lower weight gets a lower score. To summarise, the relevance of a tool to be used in a workflow decays if its usage drops in Galaxy over time. This weighing scheme filters out tools from the list of recommendations which have not been used in the last one year irrespective of their origin, either shared or non-shared workflows.

Alternatively, the relevance of tools can also be ascertained by counting the occurrence of each tool in all workflows and these occurrences can be used as their weights in the neural network training. But, it may happen that some tools which were used often in the past to create workflows are not used anymore. Therefore, assigning weights to these tools in the neural network training based on their occurrences in workflows may not be a good indicator of their relevance and overall, may not be optimal.

A curve is fit through the usage frequencies of each tool using support vector regression (SVR) to display a trend of the usage of tool over time. Using this trend, the usage of the tool for the next month is predicted and its logarithm is used as the weight for this tool in the neural network training. The logarithm of usage frequencies is computed to normalise them because only a few tools have a significantly large magnitude of usage compared to that of the remaining tools which may lead the neural network to learn and predict only tools with a very large magnitude of usage and ignore other tools. Learning a trend for each tool involves 5-fold cross-validation and optimising two hyperparameters of SVR, kernel and degree, using grid search. The values used for the kernel are - "rbf", "poly" and "linear" and the values of degree used are 2 and 3. By following the grid search, there are 3 (kernels) x 2 (degrees) = 6 different combinations of hyperparameters to be verified to find the best curve for each tool [68].

Loss function. A neural network learns patterns from data by minimising a loss function. Cross-entropy is a popular choice for a loss function in classification problems [69]. In our approach, cross-entropy function is used in the GRU neural network to compute the loss between the true and predicted label and is weighted by the label's weight. The loss is summed up over all labels of a tool sequence and then averaged (Equation 3). The term T is the total number of labels (size of the label bit vector). The term w_i is the weight of the ith label. The terms p^a and p^b refer to the true and predicted label vectors for a tool sequence, respectively. In general, the loss is large when p^a and p^b are far away from each other which means that the learning by the neural network is not good. If they are close, the loss is low and the predictions are better. When an unweighted cross-entropy is used as the loss function for any classification problem [70] then it is assumed that all the predictions have the same weight and it does not differentiate between the more and less dominant labels. In our approach when it is used as a loss function in the neural network, then even though the predicted labels are correct they may not necessarily have large weights and thereby may be less relevant. Therefore, to reduce the possibility of less relevant labels appearing in recommendations, loss is weighted by the weights of labels. It means that if a label with a larger weight is misclassified, which means that the true and predicted values are different, then the overall loss is higher. In this way, the wrong classification of a label with a larger weight is penalised more than the wrong classification of a label with a smaller weight.

$$loss = -\frac{1}{T}\sum_{i=1}^{T}(p_i^a \cdot \log(p_i^b) + (1 - p_i^a) \cdot \log(1 - p_i^b)) * w_i \quad (3)$$

The loss in Equation 3 is computed for all tool sequences in training data and is minimised using a root mean square propagation (RMSProp) optimiser. It follows an adaptive approach to estimate the learning rate by keeping knowledge of gradients in prior iterations. The learning rate is updated by dividing it with an average of the square of the prior gradients [71].

Hyperparameter tuning. The hyperparameters in our approach are optimised using Bayesian (sequential model-based) optimisation [72]. It learns from the previously evaluated configurations which ensure faster convergence. Reasonable ranges of all the hyperparameters to be optimised are given and the best configuration is found after 20 evaluations. More details are given in section S7 of the supplementary document.

Learning and predictions

Over 229,000 tool sequences collected from more than 18,000 workflows are divided into training and test data. A neural network learns patterns in the tool sequences from the training data and creates a model. The ability of the model to recommend tools is evaluated on the test data which is unseen by the neural network during training. The training data forms 80% (approximately 185,000) of all tool sequences and it is iterated over 10 epochs of neural network training. The remaining 20% (approximately 45,000) is used as the test data. The running time of the training is approximately 50 hours on a high performance compute cluster provided by bwCloud [73] with multiple cores. After learning on the training data, the model is used to predict tools. Each predicted tool gets a probability score of being the recommended tool of a tool or tool sequence. Two sets of predictions are made - shared and non-shared. Each set is sorted in the descending order of their probabilities and the top ones in both sets are combined to show them as recommendations.

Summary and future work

A system to recommend tools in Galaxy is built by analysing workflows using a variant of RNN (GRU) and a weighted cross-entropy loss function. The recommended tools are relevant for multiple scientific analyses with high accuracy as shown by the high similarities between the tools used in GTN tutorials and the recommended tools for similar analyses (Table 1). Moreover, they are easily accessible through simple UI integrations in Galaxy (Figures 6 and 7). Collectively, they improve user experience by helping researchers to easily create correct workflows. In addition, the approach does not store any information about tools and the recommendations are made by learning only the patterns of tool connections in workflows. The model [74] created using this approach and an API [75] are integrated into the European Galaxy server. The API resides with other Galaxy APIs and accesses a tool or a tool sequence specified by researchers to show its recommendations in real time using the model. The API is used at two different user interfaces in Galaxy - one shows recommendations in the workflow editor (Figure 6) and another shows them after each tool execution (Figure 7). The recommendation system should be potentially helpful for those researchers who are new to the Galaxy platform. It shows them a few follow-up tools from a big collection of more than 3,000 tools and enables them to perform multiple exploratory data analyses.

Different Galaxy servers maintain different sets of tools and workflows. The current approach can be used to create different recommendation models for different Galaxy servers. Alternatively, all the workflows can be collected from multiple Galaxy servers and using the current approach, one recommendation model can be created by learning on the complete set of workflows and the model can be distributed to different Galaxy servers. To improve the quality of recommendations, the annotations of tools can be incorporated in the learning mechanism by assigning higher weights to the annotated tools in comparison to tools which are not annotated. Tools containing similar annotations may have similar functionalities and using these similarities, tool recommendations can be further enhanced by showing similar tools for each recommended tool. In addition to learning tool connections to recommend tools, the knowledge of tools connecting to different tools based on their respective parameters can also be incorporated.

Methods

Library, model and code repositories

The Keras deep learning library is used for producing the neural network architectures [76]. The trained model [74] is saved as an H5 file to simplify its distribution to different Galaxy instances (RRID:SCR_006281). The file is an HDF5 store containing the weights of different layers of the neural network and their configurations, a dictionary of tools and their indices and the weights of tools. The weights and configuration of the neural network are needed to recreate the trained model. The dictionary is used to replace IDs of the predicted tools by their indices in a tool sequence. All data and python scripts used in our approach are stored at Github for all approaches - GRU [77], CNN [78] and DNN [79]. In each of these repositories, the process to create a tool recommendation model is explained. All these repositories are provided with a script ("extract_data.sh") for collecting raw input datasets from a Galaxy instance. These datasets are workflows and usage frequencies of tools and are also provided in each repository. The values of multiple hyperparameters of neural networks, number of training iterations and sizes of training and test data can be altered using a bash script ("train.sh"). To execute the scripts on a GPU enabled machine, the "tensorflow-gpu" package should be installed instead of "tensorflow" as mentioned in the conda package dependencies file ("environment.yml"). To see recommended tools, an ipython script ("tool_recommendation_gru_wc.ipynb" for GRU repository) is also provided which loads and recreates a trained model to predict tools for a tool or a tool sequence. The result files storing precision, training and validation losses and usage frequencies, which are used for generating line plots (Figures 3, 4 and 5), for all approaches are also available at Github [80]. The code repositories of two other approaches which do not use neural networks are available at - simple approach [31] and ExtraTrees [32].

New recommendation model

On a usual Galaxy server, tools and workflows are dynamic as they are added and updated regularly. Therefore, it is important to train the GRU neural network on the complete set of workflows periodically to keep the tool recommendation model updated with the latest tools and workflows. Using a Galaxy tool [81], a new recommendation model can be created after collecting workflows and tool usage data from a Galaxy server. The tool runs for several hours (> 24 hours) and creates a model which is pushed to an online repository [74]. From this repository, Galaxy downloads it using an API [75] to recommend tools. The recommendation model is created periodically every 3-4 months to accommodate new workflows and tools. Galaxy admins can decide upon the frequency of creating a new model. It can be created every month or every 6 months

New tools as recommendations

Galaxy admins can overwrite the recommended tools predicted using the trained model by a different set of tools using the configuration option described in [82]. In addition, to highlight the newly added tools, which are not part of the model, they can be appended to the recommendations using this additional configuration option.

Availability of supporting source code and requirements

Project name: Tool recommender in Galaxy using deep learning
Project home page: https://github.com/anuprulez/galaxy_tool_recommendation
Operating system: Linux
Programming languages: Python, XML, JavaScript
Other requirements: Tensorflow, Keras, Scikit-learn, Numpy, H5py, Csvkit, Hyperopt
License: MIT License
RRID: SCR_018491
Biotools ID: tool_recommender_system_in_galaxy

Availability of supporting data

A snapshot of the source code is available in the GigaScience GigaDB repository [83].

Additional files

File name: Tool_recommendations_supplementary_file.pdf Title: Supplementary Material: Tool recommender system in Galaxy using deep learning Description: There 3 tables - Table 1 shows a comparison of recommendations between the GRU neural network, a simple model and ExtraTrees classifier, Table 2 shows a comparison of recommendations between the regularised and non-regularised GRU neural network and Table 3 shows the strategy of uniform sampling of training data. There are 7 figures - Figures 1 and 2 show architectures of convolutional neural network (CNN) and dense neural network (DNN) used in the paper, Figure 3 shows original frequencies of last tools in training data, Figure 4 shows the frequencies of last tools in training data after uniform sampling, Figures 5 and 6 show top-1 non-shared and shared precision respectively for less frequent tools and Figure 7 shows the top-1 and top-2 precision (non-shared and shared recommendations) of the ExtraTrees classifier.

Declarations

List of abbreviations

API: Application programming interface; CNN: Convolutional neural network; DNN: Dense neural network; ELU: Exponential linear units; GRU: Gated recurrent units; PROPHETS: Process realisation and optimisation platform using human-readable expression of temporal-logic synthesis; RNN: Recurrent neural network; SVR: Support vector regression; UI: User interface; WINGS: Workflow instance generation and specialization;

Ethics approval and consent to participate

Not applicable

Consent for publication

Not applicable

Competing interests

The authors declare that they have no competing interests.

Funding

This work was supported by the German Research Foundation (DFG) under Germany's Excellence Strategy (CIBSS - EXC-2189 - Project ID 390939984) and German Federal Ministry of Education and Research (BMBF grant 031A538A de.NBI).

Authors' contributions

First author (A.K.) implemented the project and wrote the manuscript. The second author (H.R.) wrote scripts for data collection, contributed to the manuscript and deployed the project on European Galaxy server. The third author (B.G.) provided the idea of the project, validated results, and contributed to the manuscript. The last author (R.B.) contributed to the manuscript. All authors approved the manuscript.

Acknowledgements

We thank Simon Bray and Joachim Wolff for proofreading the manuscript, Dr. Wolfgang Maier for providing feedback and Gianmauro Cuccuru for deploying it on the European Galaxy server.

References

1. Ewels P, Krueger F, Käller M, Andrews S. Cluster Flow: A user-friendly bioinformatics workflow tool. F1000Research 2017;5:2824, doi:10.12688/f1000research.10335.2.
2. Leipzig J. A review of bioinformatic pipeline frameworks. Brief Bioinform 2017;1;18(3):530-536, doi:10.1093/bib/bbw020.
3. Baichoo S, Souilmi Y, Panji S, Botha G, et al. Developing reproducible bioinformatics analysis workflows for heterogeneous computing environments to support African genomics. BMC Bioinformatics 2018;19, 457 (2018), doi:10.1186/s12859-018-2446-1.
4. Love MI, Huber W, Anders S. Moderated estimation of fold change and dispersion for RNA-seq data with DESeq2. Genome Biol 2014;550. doi:10.1186/s13059-014-0550-8.
5. Afgan E, Baker D, Batut B, et al. The Galaxy platform for accessible, reproducible and collaborative biomedical analyses: 2018 update. Nucleic Acids Research 2018;46(W1):W537-W544. doi:10.1093/nar/gky379.
6. Bela G, Beel J, Hentschel C. Scienstein : A Research Paper Recommender System. In: Proceedings of the International Conference on Emerging Trends in Computing; 2009. p. 309-315. http://www.sciplore.org/wp-content/papercite-data/pdf/gipp09.pdf.
7. Achakulvisut T, Acuna DE, Ruangrong T, Kording K. Science Concierge: A Fast Content-Based Recommendation System for Scientific Publications. PLoS ONE 2016;11(7): e0158423, doi:10.1371/journal.pone.0158423.
8. Wang D, Liang Y, Xu D, et al. A content-based recommender system for computer science publications. Knowledge-Based Systems 2018;157:1-9. doi:10.1016/j.knosys.2018.05.001.
9. SGomez-Uribe CA, Hunt N. The Netflix Recommender System: Algorithms, Business Value, and Innovation. ACM Transactions on Management Information Systems TMIS 2016;6(4).
10. Smith B, Linden G. Two Decades of Recommender Systems at Amazon.com. IEEE Internet Computing 2017;21(3):12-18. doi:10.1109/MIC.2017.72.
11. Palmblad M, L LA, Ison J, Schwämmle V. Automated workflow composition in mass spectrometry-based proteomics. Bioinformatics 2019;35(4):656-664. doi:10.1093/bioinformatics/bty646.
12. Naujokat S, Lamprecht AL, Steffen B. Loose Programming with PROPHETS, Fundamental Approaches to Software Engineering, vol. 7212. J L, A Z, editors, Springer, Berlin, Heidelberg; 1996. Online ISBN 978-3-642-28872-2, doi: 10.1007/978-3-642-28872-2_7.
13. Gil Y, Ratnakar V, Kim J, et al. Wings Intelligent Workflow-Based Design of Computational Experiments. IEEE Intelligent Systems 2011;26(1):62-72. doi:10.1109/MIS.2010.9.
14. Srivastava A, Adusumilli R, Boyce H, et al. Semantic workflows for benchmark challenges: Enhancing comparability, reusability and reproducibility. PSB 2018;doi:10.1142/9789813279827_0019.
15. DiBernardo M, Pottinger R, Wilkinson M. Semi-automatic web service composition for the life sciences using the biomoby semantic web framework.

Journal of Biomedical Informatics 2008;41(5):837–847. doi:10.1016/j.jbi.2008.02.005.
16. Michalski V, Memisevic R, Konda KR. Modeling sequential data using higher-order relational features and predictive training. CoRR 2014;abs/1402.2333. http://arxiv.org/abs/1402.2333.
17. Yin W, Kann K, Yu M, Schütze H. Comparative Study of CNN and RNN for Natural Language Processing. ArXiv 2017;abs/1702.01923. http://arxiv.org/abs/1702.01923.
18. Lipton ZC, Kale DC, Elkan C, Wetzel R. Learning to diagnose with LSTM recurrent neural networks. CoRR 2015;abs/1511.03677.
19. Chung J, Gulcehre C, Cho K, Bengio Y. Empirical evaluation of gated recurrent neural networks on sequence modeling. In: NIPS 2014 Workshop on Deep Learning, December 2014; 2014. .
20. Boulanger-Lewandowski N, Bengio Y, Vincent P. Modeling Temporal Dependencies in High-Dimensional Sequences: Application to Polyphonic Music Generation and Transcription. Proceedings of the 29th International Conference on Machine Learning, ICML 2012;2.
21. Karan S, Zola J. Exact structure learning of Bayesian networks by optimal path extension. IEEE International Conference on Big Data 2016;p. 48–55. doi:10.1109/BigData.2016.7840588.
22. Spirtes P, Glymour C, Scheines R, et al. Constructing Bayesian Network Models of Gene Expression Networks from Microarray Data, Research Showcase @ CMU. Semantic Scholar 2018;doi:10.1184/R1/6491291.v1.
23. Chickering DM. Learning Bayesian Networks is NP-Complete, vol. 112. D F, HJ L, editors, New York, NY: Springer; 1996. ISBN: 978-1-4612-2404-4, doi:10.1007/978-1-4612-2404-4_12.
24. Chickering DM, Heckerman D, Meek C. Large-Sample Learning of Bayesian Networks is NP-Hard. Journal of Machine Learning Research 2004;5:1287–1330.
25. Cooper GF. The computational complexity of probabilistic inference using bayesian belief networks. Artificial Intelligence 1990;42:393–405. doi:10.1016/0004-3702(90)90060-D.
26. European Galaxy Server. https://usegalaxy.eu/ (2020);. Accessed 22 July 2020.
27. Jian X, Wickramarathne TL, Chawla NV. Representing higher-order dependencies in networks. Science Advances 2016;2(5). doi:10.1126/sciadv.1600028.
28. Said A, Bellogín Kouki A, de Vries AP. A Top-N Recommender System Evaluation Protocol Inspired by Deployed Systems. In: In Proceedings of the 2013 ACM RecSys Workshop on Large-Scale Recommender Systems. LSRS; 2013. .
29. Kang Z, Peng C, Cheng Q. Top-N Recommender System via Matrix Completion. In: Proceedings of the Thirtieth AAAI Conference on Artificial Intelligence (AAAI-16); 2016. .
30. Deshpande M, Karypis G. Item-Based Top-N recommender Algorithms. ACM Transactions on Information Systems 2004;22(1):143–177. doi:10.1145/963770.963776.
31. Kumar A, Tool Recommender in Galaxy using stored tool sequences. 2020;. https://github.com/anuprulez/galaxy_tool_recommendation/tree/statistical_model. Accessed 22 July 2020.
32. Kumar A, Tool recommender system in Galaxy using extra trees classifier. 2020;. https://github.com/anuprulez/galaxy_tool_recommendation/tree/sklearn_rf. Accessed 22 July 2020.
33. Smith T, Heger A, Sudbery I. UMI-tools: modeling sequencing errors in Unique Molecular Identifiers to improve quantification accuracy. Genome Research 2017;27(3):491–499. doi:10.1101/gr.209601.116.
34. Butler A, Hoffman P, Smibert P, Papalexi E, Satija R. Integrating single-cell transcriptomic data across different conditions, technologies, and species. Nature Biotechnology 2018;36(5):411–420. doi:10.1038/nbt.4096.
35. D G, A L, L K, K W, O B, N S, et al. Single-cell messenger RNA sequencing reveals rare intestinal cell types. Nature 2015;525(7568):251–255. doi:10.1038/nature14966.
36. A D, CA D, F S, et al. STAR: ultrafast universal RNA-seq aligner. Bioinformatics 2013;29(1):15–21. doi:10.1093/bioinformatics/bts635.
37. Liao Y, Smyth GK, Shi W. featureCounts: an efficient general purpose program for assigning sequence reads to genomic features. Bioinformatics 2013;30 (7):923–930. doi:10.1093/bioinformatics/btt656.
38. P E, M M, S L, M K. MultiQC: summarize analysis results for multiple tools and samples in a single report. Bioinformatics 2016;32(19):3047–3048. doi:10.1093/bioinformatics/btw354.
39. Wang L, Wang S, Li W. RSeQC: quality control of RNA-seq experiments. Bioinformatics 2012;28(16):2184–2185. doi:10.1093/bioinformatics/bts356.
40. Bolger AM, Lohse M, Usadel B. Trimmomatic: a flexible trimmer for Illumina sequence data. Bioinformatics 2014;30:2114–2120.
41. Li H. Aligning sequence reads, clone sequences and assembly contigs with BWA-MEM. ArXiv 2013 03;1303.
42. Garrison E, Marth G, Haplotype-based variant detection from short-read sequencing; 2012. https://arxiv.org/abs/1207.3907.
43. Langmead B, Trapnell C, Pop M, et al. Ultrafast and memory-efficient alignment of short DNA sequences to the human genome. Genome Biol 2009;10. https://doi.org/10.1186/gb-2009-10-3-r25.
44. Li H, Handsaker B, Wysoker A, Fennell T, Ruan J, Homer N, et al. The Sequence Alignment/Map format and SAMtools. Bioinformatics 2009;25 (16):2078–2079. doi:10.1093/bioinformatics/btp352.
45. Garrison E, A C++ library for parsing and manipulating VCF files.;. https://github.com/vcflib/vcflib. 2015. Accessed 02 August 2020.
46. Ramirez F, Ryan DP, Gruening B, Bhardwaj V, Kilpert F, Richter AS, et al. deepTools2: a next generation web server for deep-sequencing data analysis. Nucleic Acids Research 2016;44 (W1:W160–W165. doi:10.1093/nar/gkw257.
47. O'Boyle NM, Banck M, James CA, Morley C, Vandermeersch T, Hutchison GR. Open Babel: An open chemical toolbox. Journal of Cheminformatics 2011;3 (1). doi:10.1186/1758-2946-3-33.
48. Bray S, Protein-ligand docking (Galaxy Training Materials); 2020. https://training.galaxyproject.org/training-material/topics/computational-chemistry/tutorials/cheminformatics/tutorial.html, [Online; accessed 22 July 2020].
49. Ramirez F, Wolff J, Gruening B, Bhardwaj V, Ryan D, Duendar F, Deeptools/Hicexplorer: Winter Release;. https://zenodo.org/record/1133705. 2017. Accessed 02 August 2020.
50. Wolff J, Ramirez F, Bhardwaj V, Polkh E, Hi-C analysis of Drosophila melanogaster cells using HiCExplorer (Galaxy Training Materials); 2020. https://training.galaxyproject.org/training-material/topics/epigenetics/tutorials/hicexplorer/tutorial.html, [Online; accessed 22 July 2020].
51. Dündar F, Erxleben A, Batut B, Bhardwaj V, Ramirez F, Rabbani L, Formation of the Super-Structures on the Inactive X (Galaxy Training Materials); 2019. https://training.galaxyproject.org/training-material/topics/epigenetics/tutorials/formation_of_super-structures_

on_xi/tutorial.html, [Online; accessed 22 July 2020].
52. Kumar A, Khanteymoori A, Introduction to deep learning (Galaxy Training Materials); 2020. https://training.galaxyproject.org/training-material/topics/statistics/tutorials/intro_deep_learning/tutorial.html, [Online; accessed 22 July 2020].
53. Kessner D, Chambers M, Burke R, Agus D, Mallick P. ProteoWizard: open source software for rapid proteomics tools development. Bioinformatics 2008;24 (21):2534–2536. doi:10.1093/bioinformatics/btn323.
54. Sigloch FC, Grüning B, Peptide and Protein ID using OpenMS tools (Galaxy Training Materials); 2020. https://training.galaxyproject.org/training-material/topics/proteomics/tutorials/protein-id-oms/tutorial.html, [Online; accessed 22 July 2020].
55. Marcel M. Cutadapt removes adapter sequences from high-throughput sequencing reads. EMBnetjournal 2011;17 (1). doi:10.14806/ej.17.1.200.
56. Batut B, Freeberg M, Heydarian M, Erxleben A, Videm P, Blank C, et al., Reference-based RNA-Seq data analysis (Galaxy Training Materials); 2020. https://training.galaxyproject.org/training-material/topics/transcriptomics/tutorials/ref-based/tutorial.html, [Online; accessed 22 July 2020].
57. Tekman M, Batut B, Erxleben A, Maier W, Pre-processing of Single-Cell RNA Data (Galaxy Training Materials); 2020. https://training.galaxyproject.org/training-material/topics/transcriptomics/tutorials/scrna-preprocessing/tutorial.html, [Online; accessed 22 July 2020].
58. Tekman M, Ostrovsky A, Downstream Single-cell RNA analysis with RaceID (Galaxy Training Materials); 2020. https://training.galaxyproject.org/training-material/topics/transcriptomics/tutorials/scrna-raceid/tutorial.html, [Online; accessed 22 July 2020].
59. Nekrutenko A, Soranzo N, Calling variants in diploid systems (Galaxy Training Materials); 2019. https://training.galaxyproject.org/training-material/topics/variant-analysis/tutorials/dip/tutorial.html, [Online; accessed 22 July 2020].
60. Batut B, et al. Community-Driven Data Analysis Training for Biology. Cell Systems 2018;6:752–758.E1. https://training.galaxyproject.org/, doi:10.1016/j.cels.2018.05.012.
61. Tsoumakas G, Katakis I. Multi-label classification: An overview. International Journal of Data Warehousing and Mining 2009;3:1–13. doi:10.4018/jdwm.2007070101.
62. Ruiz-Carmona S, Alvarez-Garcia D, Foloppe N, Garmendia-Doval AB, Juhos S, Schmidtke P, et al. rDock: A Fast, Versatile and Open Source Program for Docking Ligands to Proteins and Nucleic Acids. PLoS Computational Biology 2014;10 (4). doi:10.1371/journal.pcbi.1003571.
63. Pascanu R, Mikolov T, Bengio Y. Understanding the exploding gradient problem. ArXiv 2012;abs/1211.5063.
64. Zaremba W, Sutskever I, Vinyals O. Recurrent Neural Network Regularization. ArXiv 2014;abs/1409.2329.
65. Gal Y, Ghahramani Z. A Theoretically Grounded Application of Dropout in Recurrent Neural Networks. In: Proceedings of the 30th International Conference on Neural Information Processing Systems; 2016. p. 1027–1035.
66. Clevert D, Unterthiner T, Hochreiter S. Fast and Accurate Deep Network Learning by Exponential Linear Units (ELUs). CoRR 2015;abs/1511.07289.
67. Nair V, Hinton GE. Rectified Linear Units Improve Restricted Boltzmann Machines. In: ICML'10: Proceedings of the 27th International Conference on International Conference on Machine Learning, June 2010; 2010. p. 807–814.
68. Pedregosa F, Varoquaux G, Gramfort A, et al. Scikit-learn: Machine Learning in Python. Journal of Machine Learning Research 2011;12:2825–2830. doi:10.5555/1953048.2078195.
69. Janocha K, Czarnecki W. On Loss Functions for Deep Neural Networks in Classification. ArXiv 2017;abs/1702.05659. doi:10.4467/20838476SI.16.004.6185.
70. Sadowski P, Notes on Backpropagation. https://www.ics.uci.edu/~pjsadows/notes.pdf (2016). Accessed 22 July 2020;.
71. Ruder S. An overview of gradient descent optimization algorithms. ArXiv 2016;abs/1609.04747.
72. Bergstra J, Yamins D, Cox DD. Hyperopt: A Python Library for Optimizing the Hyperparameters of Machine Learning Algorithms. 12th Python in science conf (SCIPY 2013) 2013;.
73. BwCluster. https://portal.bw-cloud.org/project/ (2020);. Accessed 22 July 2020.
74. Kumar A, Tool Recommender model;. https://github.com/galaxyproject/galaxy-test-data/blob/master/tool_recommendation_model.hdf5. 2020. Accessed 29 July 2020.
75. Kumar A, Get tool predictions. 2020;. https://github.com/usegalaxy-eu/galaxy/blob/release_20.05_europe/lib/galaxy/webapps/galaxy/api/workflows.py#L638. Accessed 22 July 2020.
76. Chollet F, et al. Keras 2015;.
77. Kumar A, Tool Recommender in Galaxy using GRU neural network. 2020;. https://github.com/anuprulez/galaxy_tool_recommendation. Accessed 22 July 2020.
78. Kumar A, Tool Recommender in Galaxy using CNN neural network. 2020;. https://github.com/anuprulez/galaxy_tool_recommendation/tree/cnn_wc. Accessed 22 July 2020.
79. Kumar A, Tool Recommender in Galaxy using DNN neural network. 2020;. https://github.com/anuprulez/galaxy_tool_recommendation/tree/dnn_wc. Accessed 22 July 2020.
80. Kumar A, Output results files. 2020;. https://github.com/anuprulez/galaxy_tool_recommendation/tree/master/output_files/data_20_05. Accessed 22 July 2020.
81. Kumar A, Tool Recommender model creator;. https://usegalaxy.eu/root?tool_id=toolshed.g2.bx.psu.edu/repos/bgruening/create_tool_recommendation_model/create_tool_recommendation_model/0.0.3. 2020. Accessed 22 July 2020.
82. Kumar A, Tool Recommender overwrite;. https://github.com/usegalaxy-eu/galaxy/blob/release_20.05_europe/config/tool_recommendations_overwrite.yml.sample. 2020. Accessed 30 July 2020.
83. Kumar A, Rasche H, Gruening B, Backofen R, Supporting data for "Tool recommender system in Galaxy using deep learning" GigaScience Database; 2020. http://dx.doi.org/10.5524/100838.

Supplementary figure 1 Click here to access/download;Figure;supplementary_fi

Architecture of Convolutional neural network (CNN)

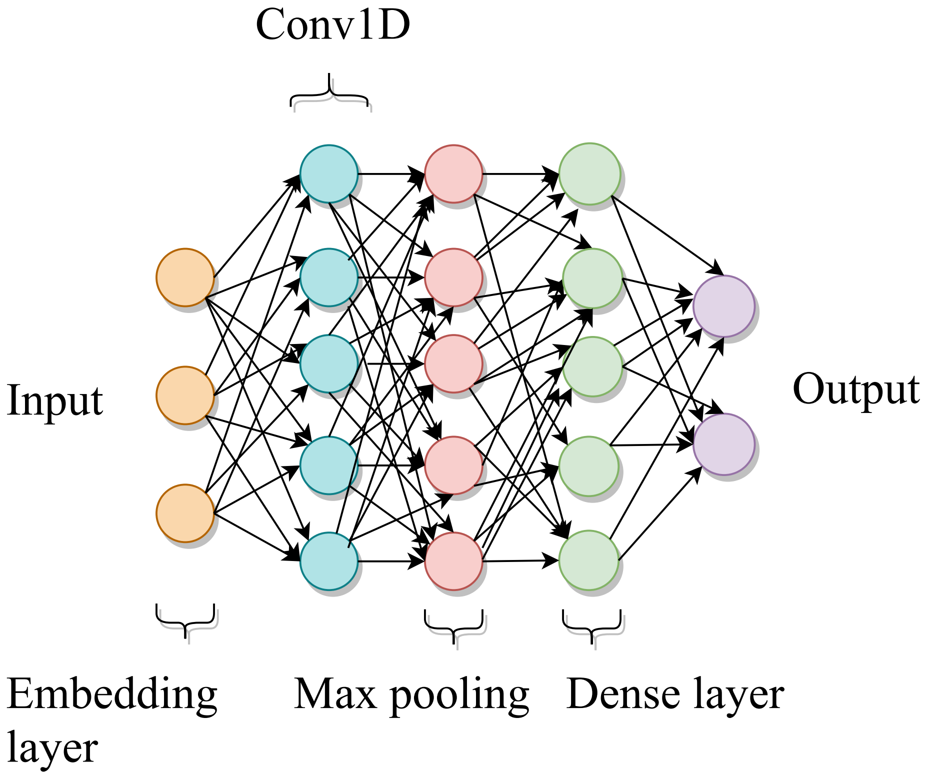

Supplementary figure 2

Click here to access/download;Figure;suppleme

Architecture of Dense neural network (DNN)

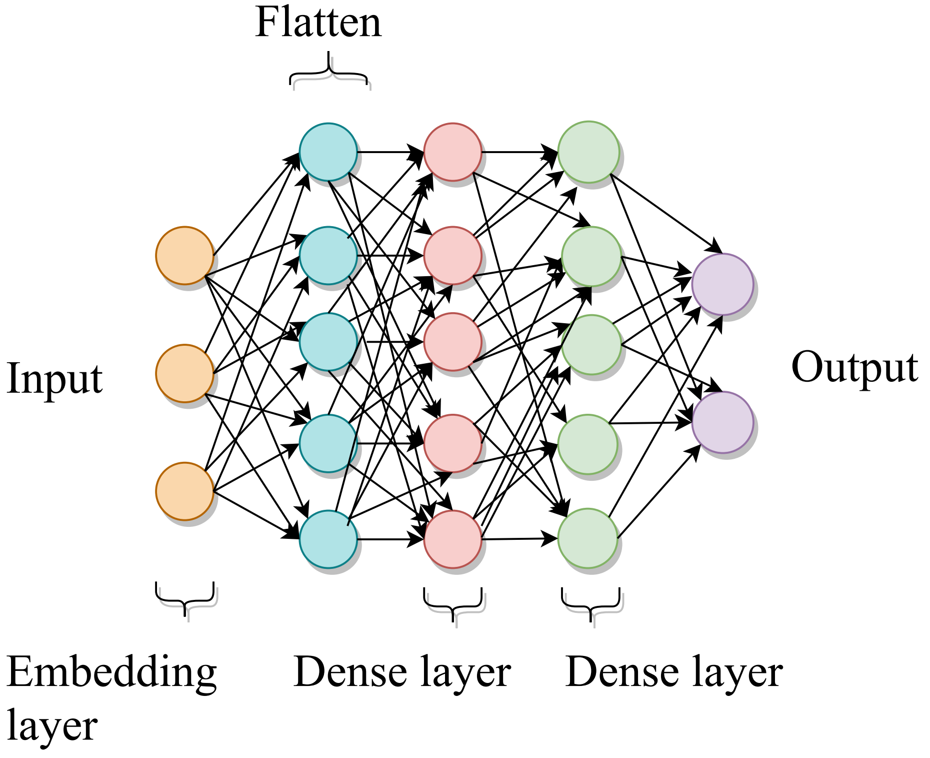

Click here to access/download;Figure;supplementary_figure_3.pdf

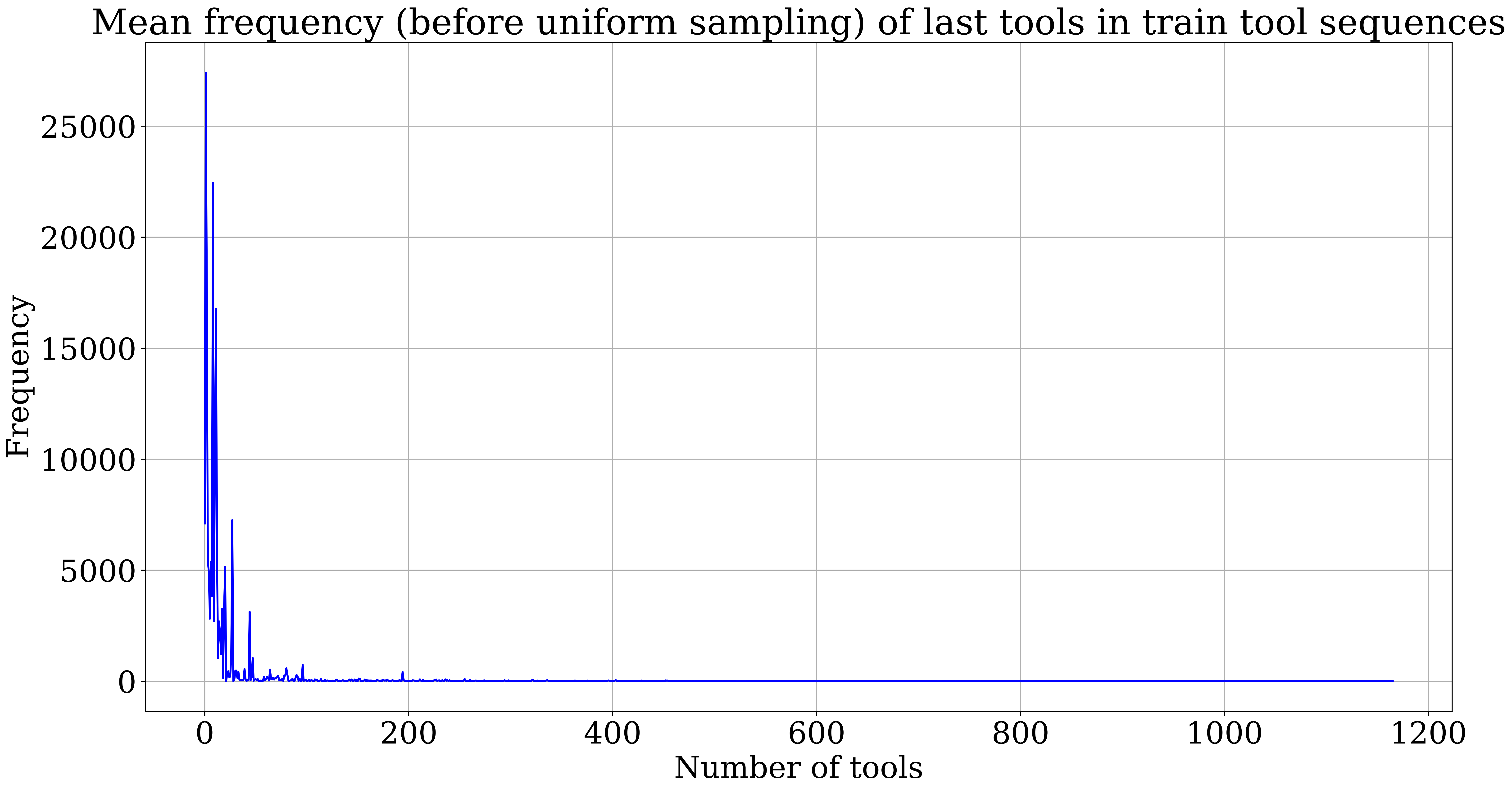

Click here to access/download;Figure;supplementary_figure_4.pdf

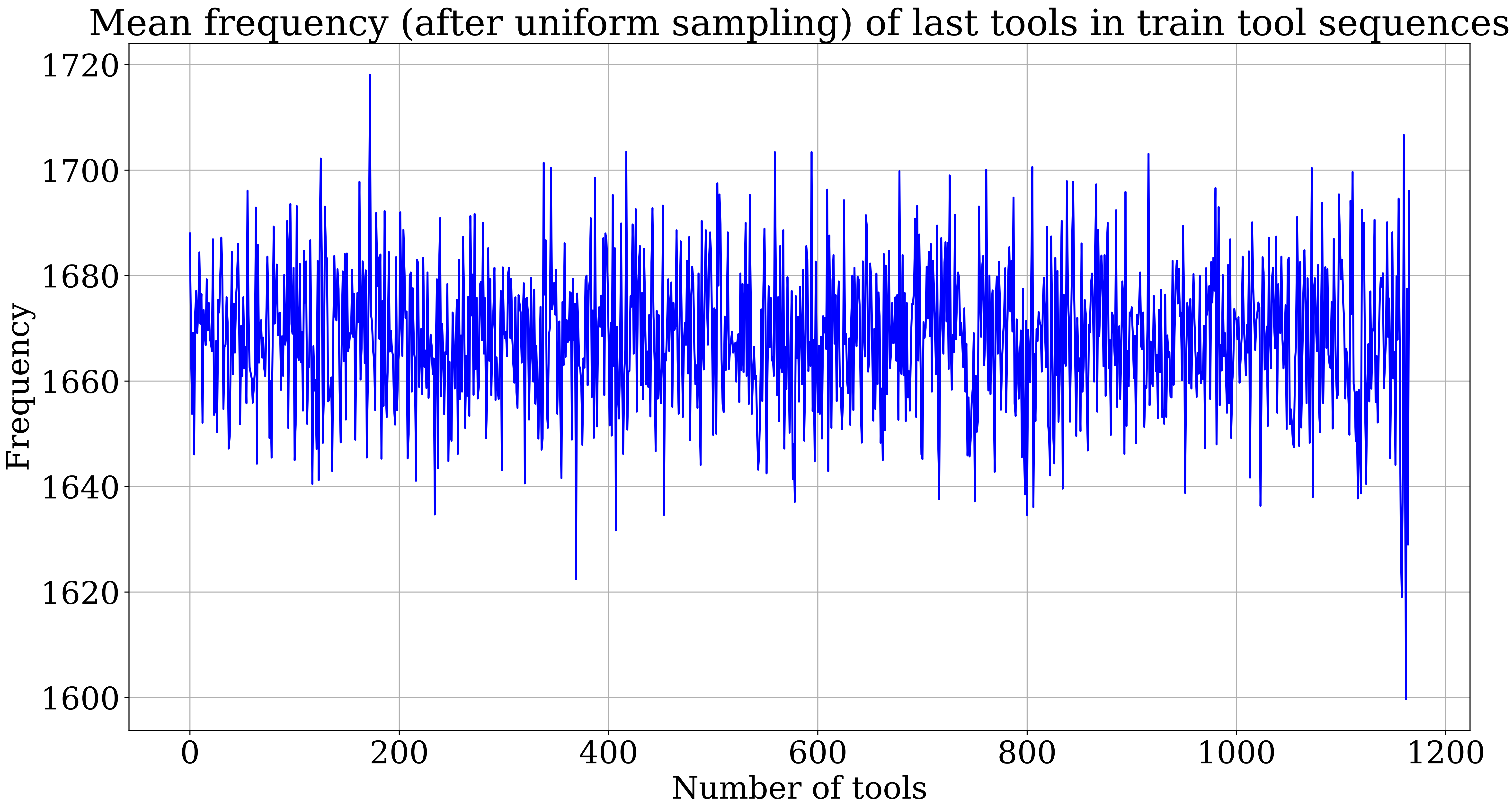

Click here to access/download;Figure;supplementary_figure_5.pdf

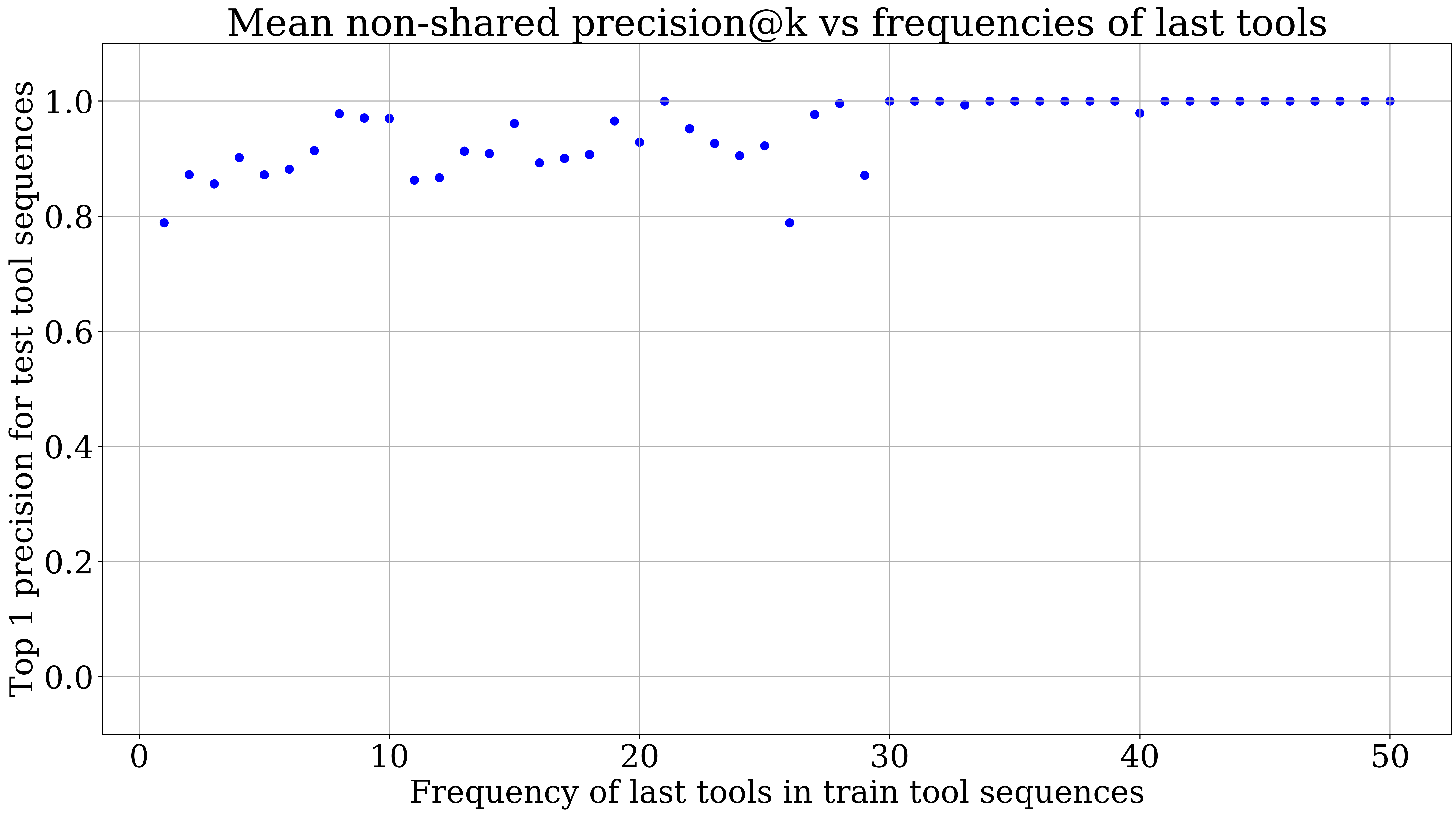

Click here to access/download;Figure;supplementary_figure_6.pdf

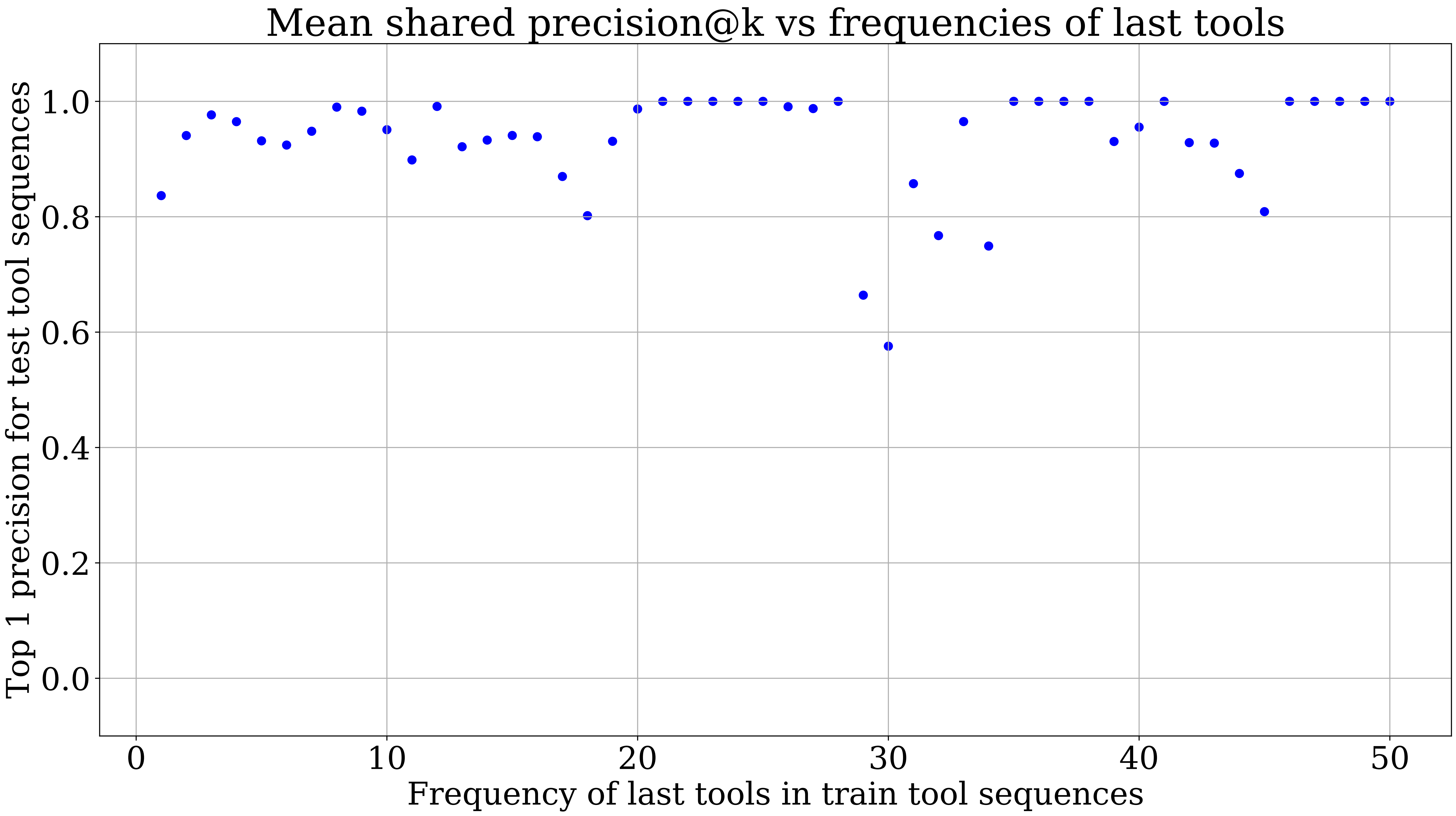

Click here to access/download;Figure;supplementary_figure_7.pdf

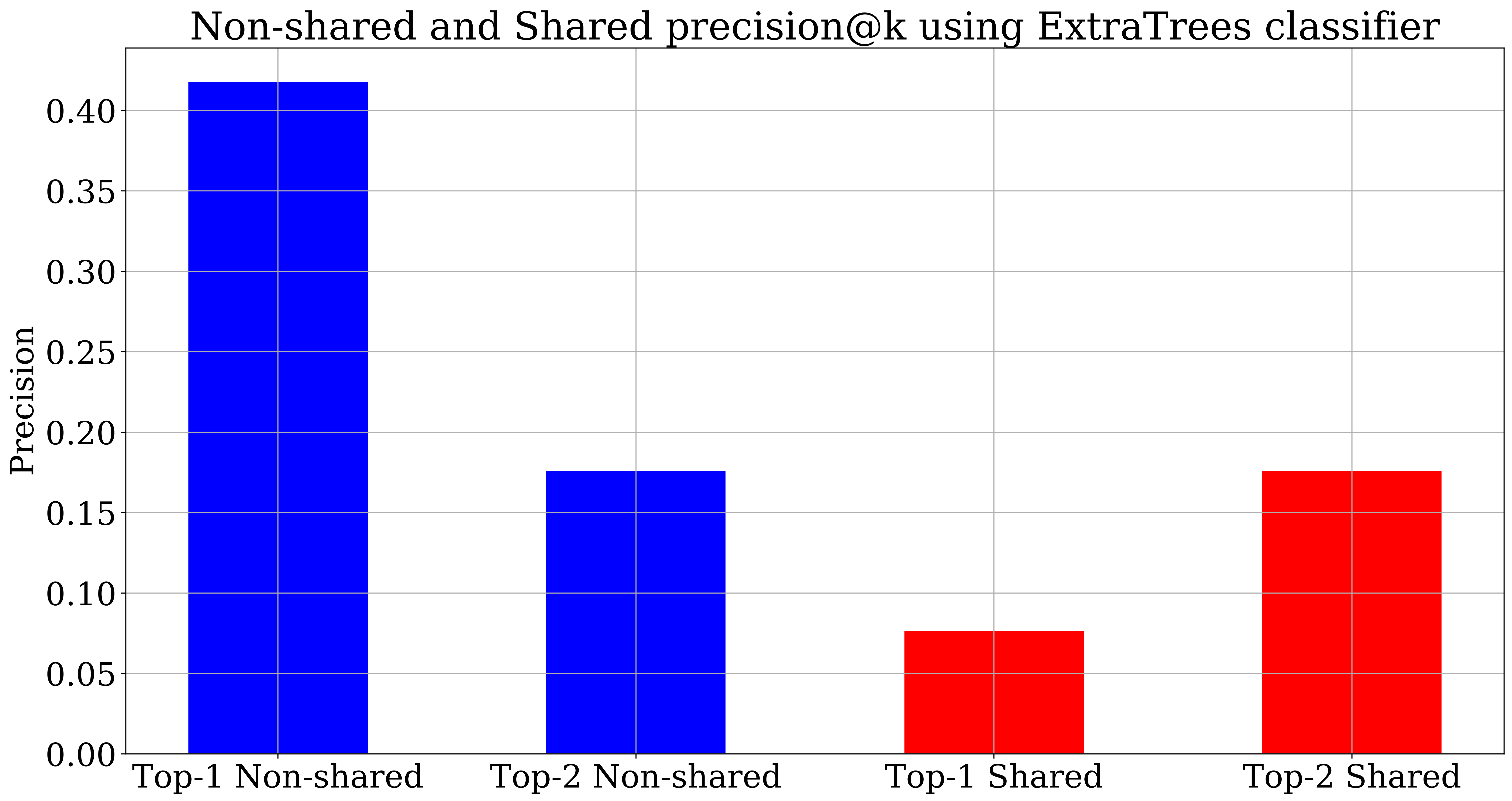

Click here to access/download
Supplementary Material
Tool_recommendations_supplementary_file.pdf
