# Supplementary material for: Tool recommender system in Galaxy using deep learning
Source: Gigascience. 2021 Jan 6;10(1):giaa152. doi: 10.1093/gigascience/giaa152 (PMC7786169; doi:10.1093/gigascience/giaa152)
Supplement: giaa152_Supplemental_Files [file giaa152_supplemental_files.zip › Tool_recommendations_supplementary_file.pdf]

Supplementary Material: Tool recommender system in Galaxy using deep learning

Anup Kumar[1,*] (kumara@informatik.uni-freiburg.de)
Helena Rasche[2,*] (helena.rasche@gmail.com)
Bjoern Gruening[3,*] (gruening@informatik.uni-freiburg.de)
Rolf Backofen[4,*,^] (backofen@informatik.uni-freiburg.de)

* Bioinformatics Group, Department of Computer Science, University of Freiburg, Georges-Koehler-Allee 106, 79110 Freiburg, Germany
^ Signalling Research Centres BIOSS and CIBSS, University of Freiburg, Schaenzlestr. 18, 79104 Freiburg, Germany

Contributions follow the order of the names of authors.
All correspondence should be made to: Anup Kumar (kumara@informatik.uni-freiburg.de).

S1. Tool recommendations using simpler approaches other than neural networks

To compare the performance of the GRU neural network with approaches without any use of a neural network, two ideas are explored - a simple approach to store all indices of sequences of tools or to use an ExtraTrees classifier. The simple approach to recommend tools is implemented by storing the indices of tool sequences extracted from over 18,000 workflows on the European Galaxy server. The size of the resulting model (simple model) is 46 MB while its size created using the GRU neural network is only 6 MB. As the number of workflows grows in future, the model size will grow too, posing limitations in storing and sharing it over online platforms such as Galaxy. Therefore, to limit the size of the model, machine learning and deep learning methods are more suitable where it is not required to store any data. Instead, it is sufficient only to store the weights of features in data. Moreover, the GRU neural network recommends tools similar to the ones recommended by the simple model (Table 1 in supplementary section S2) for multiple scientific analyses.

In addition, ExtraTrees, an ensemble-based classifier, is also trained on tool sequences to recommend tools. The optimisation of hyperparameters such as the number of estimators, depth of trees and the uniform sampling of the training data was done following the same approach as used for training the GRU neural network. The model trained with the ExtraTrees classifier achieves a precision of less than 0.5 for the top-1 metric (Supplementary Figure 7) which is very low compared to the accuracy of the GRU neural network (0.98 top-1 precision). It shows that the ExtraTrees classifier is unsuitable for learning on sequential data as used in this project. Moreover, the peak memory usage for the ExtraTrees classifier is approximately 75 GB while for the GRU neural network it is approximately 8 GB.

In summary, large model size of the simple model and low precision and high memory usage for the ExtraTrees classifier do not qualify them as good approaches to create the tool recommendation system. On the other hand, the GRU neural network has a smaller model size and memory usage and

higher precision which collectively make it a better approach to create the tool recommendation system.

S2. Comparison of recommendations for different bioinformatic analysis: GRU neural network with the simple and ExtraTrees models

Analysis	Tool	GRU model	Simple model	ExtraTrees	Reference
**RNA-seq**	FastQC	MultiQC	MultiQC	None	[GTN link]
**RNA-seq**	Featurecounts	DESeq2	DESeq2	None	[GTN link]
**Proteomics**	MSI preprocessing	MSI mz images, MSI Qualitycontrol	MSI mz images, MSI Qualitycontrol	None	
**Hi-C**	hicBuildMatrix	hicCorrectmatrix	hicCorrectmatrix	None	[GTN link]
**Single-cell**	UMI-tools extract	RNA-STAR, Je-Demultiplex, FastQC	RNA-STAR, Je-Demultiplex, FastQC	None	[GTN link]
**Cheminformatics**	Remove counterions and fragments	Molecule to fingerprint, Compound Convert	Molecule to fingerprint, Compound Convert	None	
**Imaging**	Filter Image	Auto Threshold, Count Objects, Histogram equalization	Auto Threshold, Count Objects, Histogram equalization	None	
**Variant-calling**	BamLeftAlign	Filter	Filter	None	
**Assembly**	Create assemblies with Unicycler	Prokka, Quast	Prokka, Quast	None	[GTN link]

Table 1: Comparison of recommendations by the GRU, simple and ExtraTrees models. From column 3 and 4 it can be concluded that the GRU and simple models have similar recommendations for the same tools. The ExtraTrees model is not able to recommend any tool for the tools mentioned in the second column.

S3. Architectures of other neural networks

Multiple neural network architectures

Multiple neural network architectures such as a convolutional neural network (CNN) and dense neural network (DNN) with only dense layers, are used to compare their respective predictive strengths with the GRU neural network (Figures 3, 4 and 5 in the paper). In these architectures too, the embedding layer is used as the first (input) layer and a dense layer is used as an output layer having the same dimensions as the number of tools. Additionally, in CNN, convolutional and max-pooling layers are used to learn spatial patterns in tool sequences and downsample the dimensionality of input, respectively. Moreover, two dense layers are also used and the last one serves as an output layer. DNN uses two dense layers as hidden layers. The cross-entropy, with and without weights, is used as the loss function and RMSProp is used as an optimiser. Bayesian optimization is used to optimise the hyperparameters of these architectures.

S3.1 In the Convolutional neural network (CNN) architecture (Figure 1), one convolution (1D), one max-pooling and one dense layer collectively form the hidden structure and together they map the sequences of tools to their respective labels. The input layer is an embedding layer which learns a fixed-size vector for each tool. The output layer is a dense layer and the number of units for this layer is the number of tools.

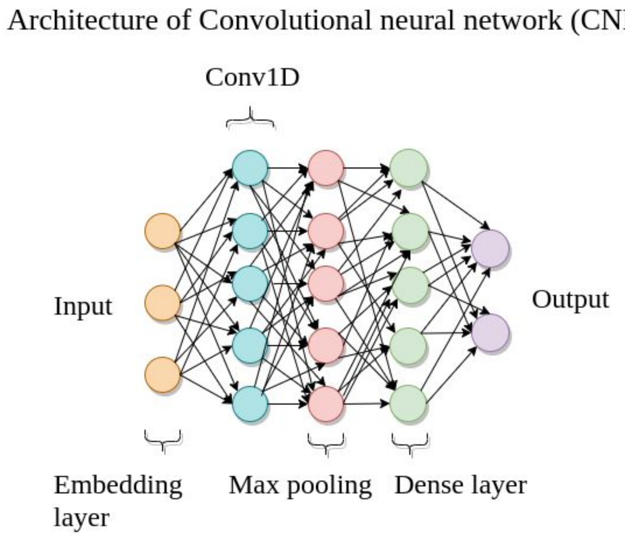

Figure 1: Neural network architecture for CNN

S3.2 In the dense neural network (DNN) architecture (Figure 2), only dense layers are used to map the sequences of tools to their respective labels. Similar to the other architectures, the input layer is an embedding layer and the output layer is a dense layer.

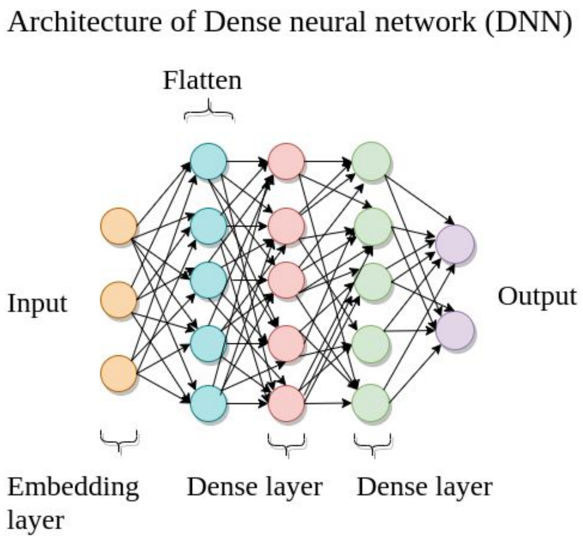

Figure 2: Neural network architecture for DNN

S4: Comparison of recommendations made by the regularised GRU vs non-regularised GRU models

Analysis	Tool Sequences	Regularised model	Non-regularised model
**Single-cell**	RaceID, Lineage computation using StemID	Lineage Branch Analysis using StemID	None
**Single cell**	Scanpy RegressOut, Scanpy ScaleData, Scanpy RunPCA, Scanpy RunTSNE, Scanpy FindMarkers	Scanpy RunUMAP, Plot with scanpy, Scanpy run TSNE	Scanpy run TSNE
**Single cell**	UMI-tools count	Text transformation with sed, Column Join on Collections, Initial processing using RaceID, Transpose rows/columns, Seurat	Text transformation with sed, Column Join on Collections, Initial processing using RaceID, Transpose rows/columns
**Machine learning**	Hyperparameter Search	Ensemble methods for classification and regression	Scatterplot with ggplot2
**RNA**	Cutadapt, ChiRA collapse,	ChiRA quantify	None

	ChiRA map, ChiRA merge		
**RNA**	Cutadapt, ChiRA collapse, ChiRA map, ChiRA merge, ChiRA qauntify	ChiRA extract	None
**RNA**	RNA-STAR	Read Distribution ...	Other recommendations are same and Read distribution is absent

Table 2: Comparison of recommendations for the regularised and non-regularised GRU models. The tools shaded in green are recommended only by the regularised GRU model and are absent in the recommendations by the non-regularised GRU model for the respective tool sequences. The tools shaded in green are not recommended by the non-regularised GRU model.

S4.1 Learned weights of regularised and non-regularised models

The use of dropout in the GRU neural network for regularisation allowed the model to have smaller weights compared to the non-regularised GRU neural network model. Overall mean of weights (weights of all layers) for the regularised model is 0.075 (See "Unpack trained model for prediction" section at https://github.com/anuprulez/galaxy_tool_recommendation/blob/master/ipython_script/tool_recommendation_gru_wc.ipynb) while for the non-regularised model, it is 0.096 (See "Unpack trained model for prediction" section at https://github.com/anuprulez/galaxy_tool_recommendation/blob/no_regularisation/ipython_script/tool_recommendation_gru_wc_no_reg.ipynb). Having larger weights in a neural network model may lead to overfitting as it is an indication of a more complex network. In an overfit model, the neurons (neural network units) in a layer try to fix the errors made in the previous layers to make the model robust (on the training data) and may not learn more general features. One of the advantages of using regularisation in a recommendation engine can be to avoid the following situation - an overfit model may try to learn the most common tools with higher usage frequency to minimise the error (due to the weighted cross-entropy loss function) and may ignore tools with lower usage, but important ones. For example, the regularised model recommends the following tools for the "UMI-tools count" tool. The (log) usage frequency has been shown beside each tool name.

- Text transformation with sed (3.98) [Shared recommendation]
- Column Join on Collections (4.78) [Non-shared recommendation]
- Initial processing using RaceID (3.68) [Non-shared recommendation]
- Transpose rows/columns (3.57) [Non-shared recommendation]
- Seurat (2.67) [Non-shared recommendation]

The non-regularised model also recommends the same tools except "Seurat" tool which has the lowest usage frequency (log usage frequency) out of all recommended ones.

S5. Effect of uniform sampling

S5.1 Uniform sampling vs precision for less frequent tool sequences

Precision computed after training on uniformly sampled training data is shown in figures 5 and 6. The frequency of the last tools in tool sequences was calculated only in training tool sequences. The test tool sequences were not used. After learning on the training tool sequences, the trained model was used to predict tools for test tool sequences and precision was calculated based on the frequency of the last tools in training tool sequences. In Figure 5, the y-axis shows the precision for test tool sequences and the x-axis shows the frequencies of their respective last tools in the original training tool sequences. The non-shared precision of tool sequences whose last tools have a frequency < 30 is shown in the top-left section of Figure 5. The precision achieved for these tool sequences is approximately 0.9 and for the tool sequences which have higher frequencies (frequency > 30) of their last tools achieve 1.0 precision. The less frequent tool sequences achieve shared precision of approximately 0.9 (Figure 6). It becomes possible because while training, the GRU neural network learns features from uniformly distributed tool sequences.

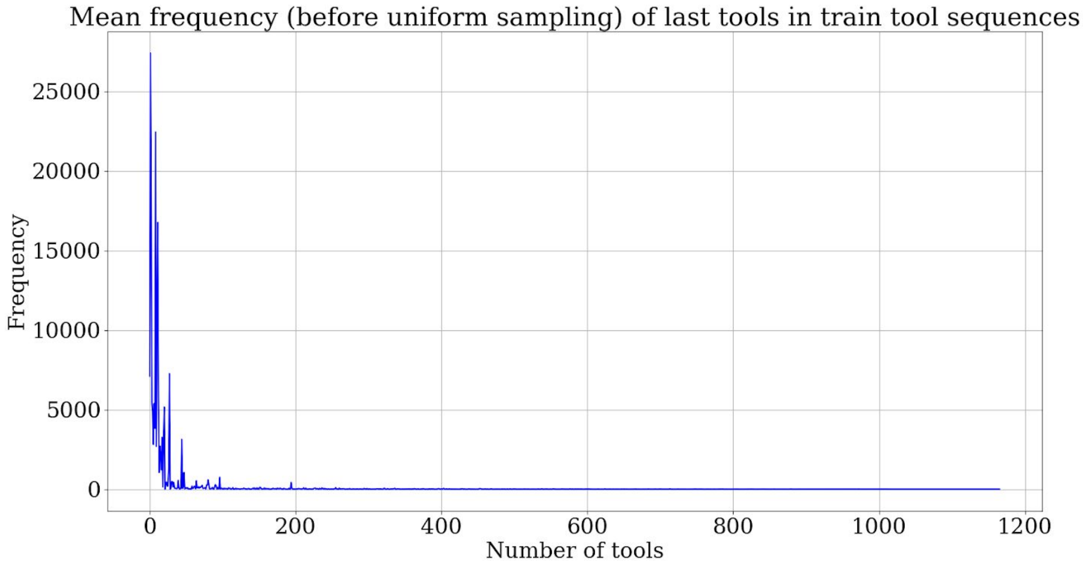

Figure 3: Frequencies of last tools in train tool sequences. It can be seen that only a few tools have large frequencies (some of the leftmost tools are - ‘Concatenate datasets’, ‘Cut’ and ‘Join’) and most of the tools have very low frequencies (some of those tools are - “Cluster inspection using RaceID”, “rDock cavity definition” and “ChiRA collapse”).

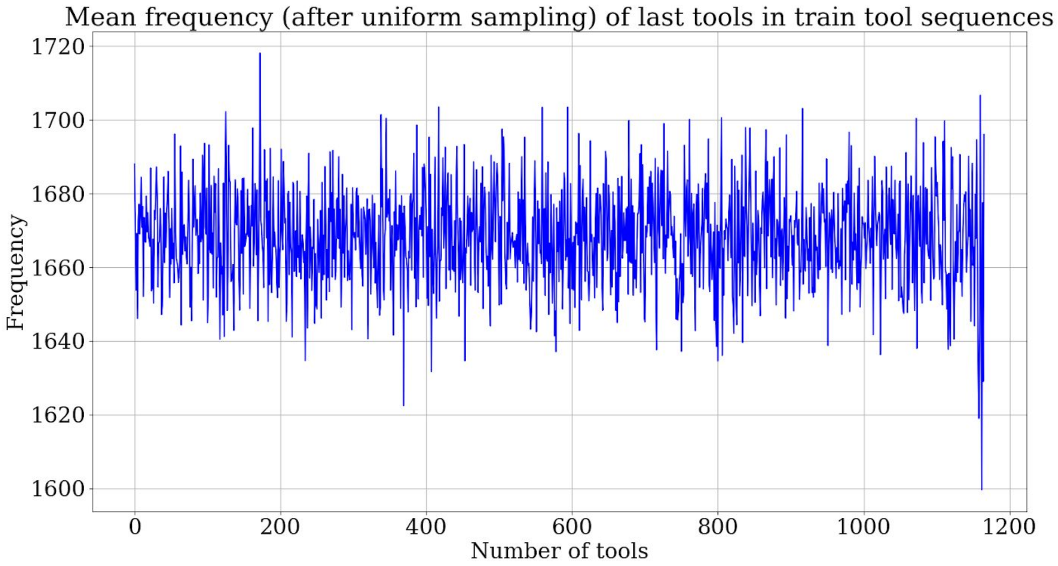

Figure 4: Frequencies of last tools in train tool sequences after uniform sampling. In the plot, all the tools have similar frequencies (1670).

S5.2 Strategy for uniform sampling

**Tool sequence**	**Last tool**	**List of indices of tool seqs for which tool in the second column is the last tool**
Tool C, Tool A	Tool A	[5, 1, 9, 453]
Tool E, Tool D, Tool B	Tool B	[4, 90, 76, 3]
Tool Z, Tool C	Tool C	[7, 6]
...	...	...

Table 3: List of last tools with their respective indices of tool sequences in the training data. While creating training data, a fixed number of tools from the second column are selected uniformly and for each tool, an index is selected from its list of indices (column 3) again uniformly which is the index of a sample in the original training data.

S5.3 Precision for less frequent tool sequences in test data

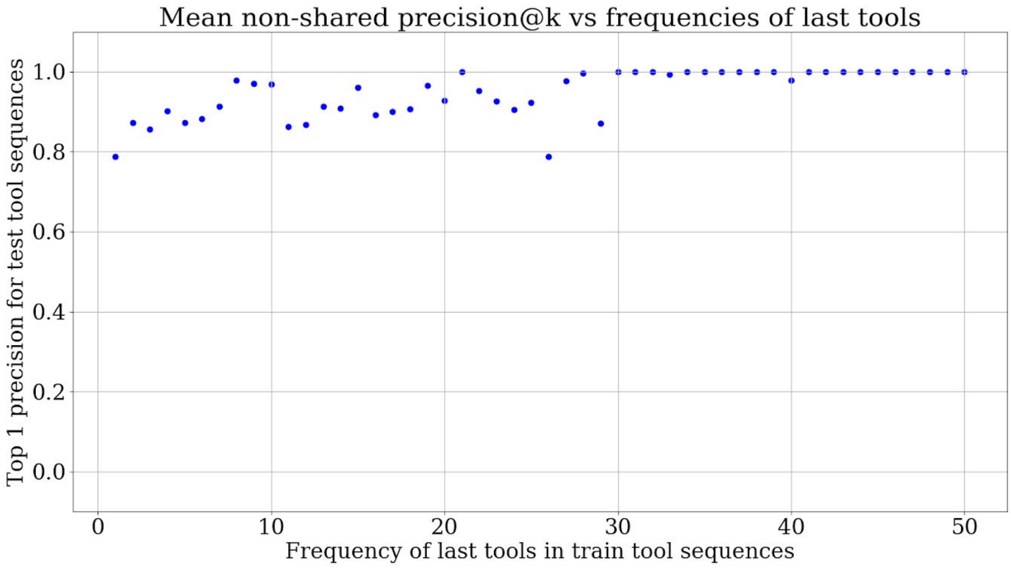

Figure 5: Top 1 non-shared precision in test tool sequences vs frequency of last tools in training tool sequences. It can be seen that even for very less frequent tools (< 30), the top 1 non-shared precision is approximately 0.9 (90%).

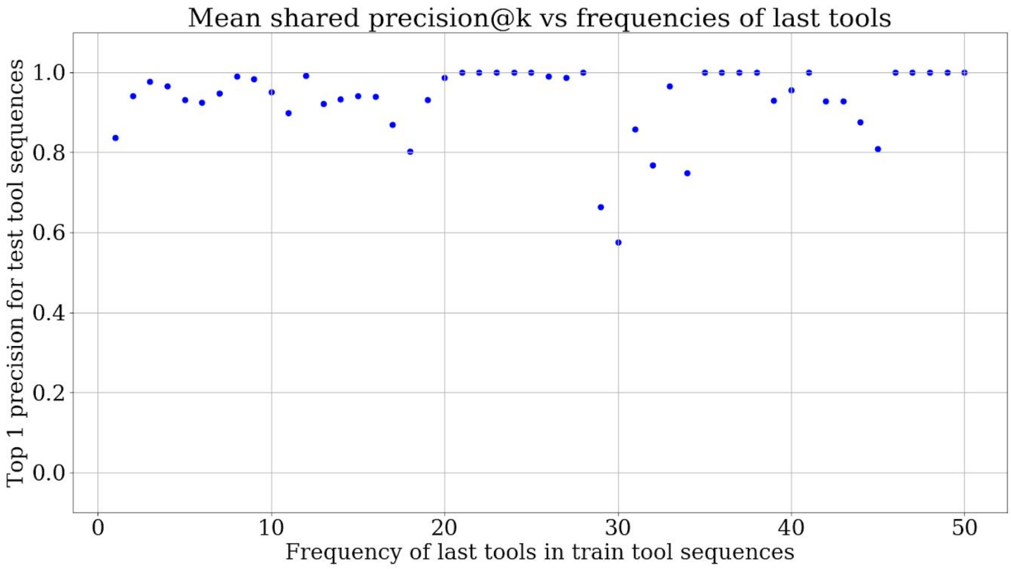

Figure 6: Top 1 shared precision in test tool sequences vs frequency of last tools in training tool sequences. It can be seen that for most tools with very low frequencies (< 30), the top 1 shared precision is approximately 0.9 (90%).

S6: ExtraTrees classifier

To showcase another example of a classifier used in addition to the GRU neural network to recommend tools, ExtraTrees, an ensemble-based classifier, is used to learn on tool sequences. For the ExtraTrees classifier, 95% data is used for training and only 5% is used for testing. Hyperparameters of the ExtraTrees classifier such as the number of estimators, depth of trees and a few others are optimised using the same approach as used for the GRU neural network (Bayesian optimisation). The precision achieved is less than 0.5 (Figure 7). When 80% of data is used for training and 20% for testing as used in the other compared approaches such as the GRU neural network, the precision becomes worse. Moreover, the memory usage of this approach is also high as the peak memory usage is approximately 75 GB. Due to the low precision (< 0.5) for non-shared and shared recommendations and high memory requirements, ExtraTrees classifier is not suitable for learning on the sequences of tools.

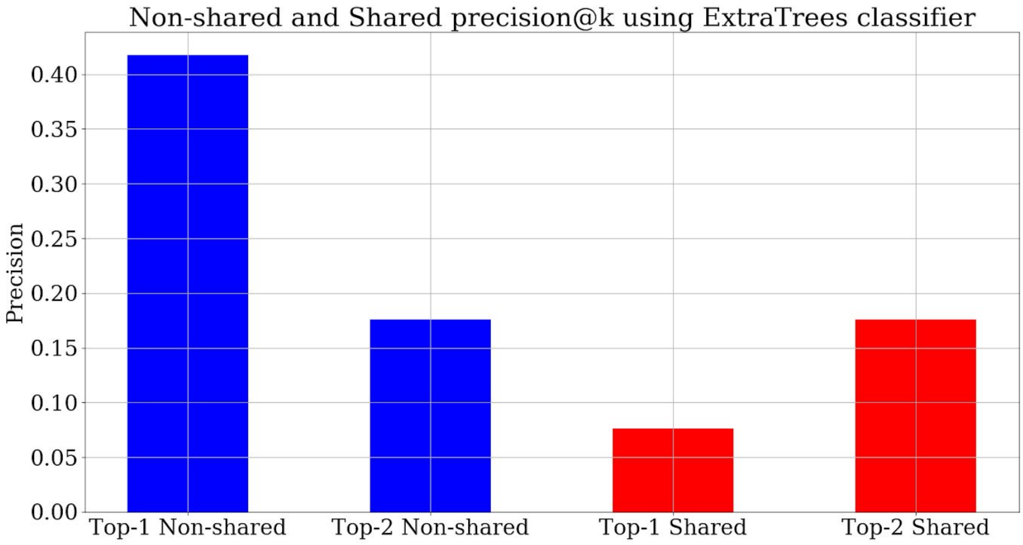

Figure 7: Non-shared and shared precision for top-1 and top-2 metrics using ExtraTrees classifier are shown. Both precision are less than 0.5 (50%) which is much lower than achieved by compared approaches such as GRU neural network (0.98 top-1 non-shared and shared precision). Therefore, it cannot be used for recommending tools. Shared recommendations are learned from tool connections in shared and non-deleted workflows while non-shared recommendations are from other workflows.

S7: Hyperparameter tuning

In our approach, the neural network has multiple hyperparameters such as the number of dimensions of the embedding layer, learning and dropout rates, number of units for the GRU layer and size of batches. They should be optimised to find the best configuration (a combination of hyperparameters) for training on tool sequences as a different configuration may give different performance on the same training data. The grid and random searches are popular techniques to optimise hyperparameters. One limitation of these approaches is that they evaluate each configuration independently and have a high time complexity to find the best configuration. Therefore, the hyperparameters in our approach are optimised using a Bayesian (sequential model-based) optimisation [1]. It learns from the previously evaluated configurations which ensure faster convergence. Reasonable ranges of all the hyperparameters to be optimised are given and the best configuration is found after 20 evaluations.

S8: Tool parameters and individual tool recommendations

In Galaxy, a tool has many parameters and when these parameters are configured differently, the tool may connect to multiple tools. In our approach, tool connections in workflows are used to recommend tools. If there are such patterns, a tool connecting to multiple tools, available in the workflows, our recommendation model captures them. For example, if tool A, with a different configuration of parameters, connects to tool B and with another configuration, connects to tool C, then our model learns both these connections for tool A -> tool B and tool A -> tool C. While recommending tools for tool A, both these connections (tool B and tool C) are predicted. However, if there are no patterns available showing the use of multiple connections of tools based on a different configuration of parameters, our model does not know about it as we can learn and predict only what we see.

In addition, only one tool is recommended at a time instead of recommending a chain of tools. Recommending one tool at a time is beneficial for a few reasons. First, it enables users to diversify the usage of tools by showing tools from multiple scientific analyses at each step of their analyses. Second, if chains of tools which are always used together are recommended and if one or more of them get deprecated (maybe because they are old and not used anymore), it would make little sense to show them together and at the same time, excluding them would introduce a gap in that particular analysis. But, if we predict only one tool at a time and the tool gets deprecated, we simply exclude it and show only relevant tools. Lastly, it is not the aim of the project currently to recommend chains of tools.

References

[1] Bergstra J, Yamins D, Cox DD. Hyperopt: A Python Library for Optimizing the Hyperparameters of Machine LearningAlgorithms. 12th Python in science conf (SCIPY 2013) 2013
