# Supplementary figures and images for: Tool recommender system in Galaxy using deep learning
Source: Gigascience. 2021 Jan 6;10(1):giaa152. doi: 10.1093/gigascience/giaa152 (PMC7786169; doi:10.1093/gigascience/giaa152)

# Architecture of Convolutional neural network (CNN)

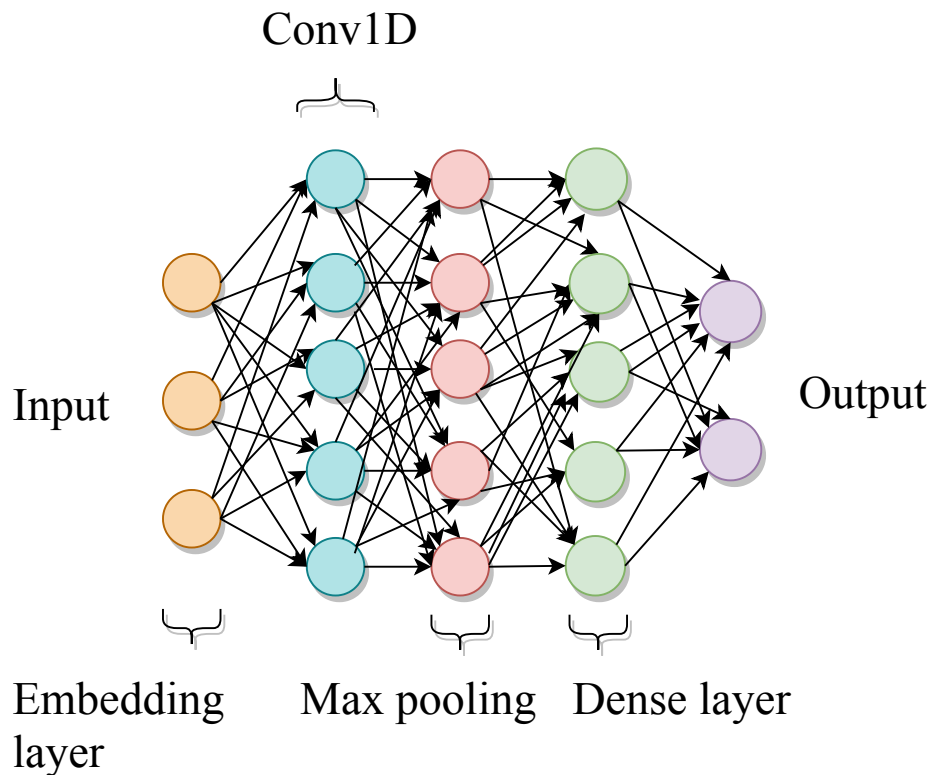

Supplement: giaa152_Supplemental_Files [file giaa152_supplemental_files.zip › supplementary_figure_1.pdf]

# Architecture of Dense neural network (DNN)

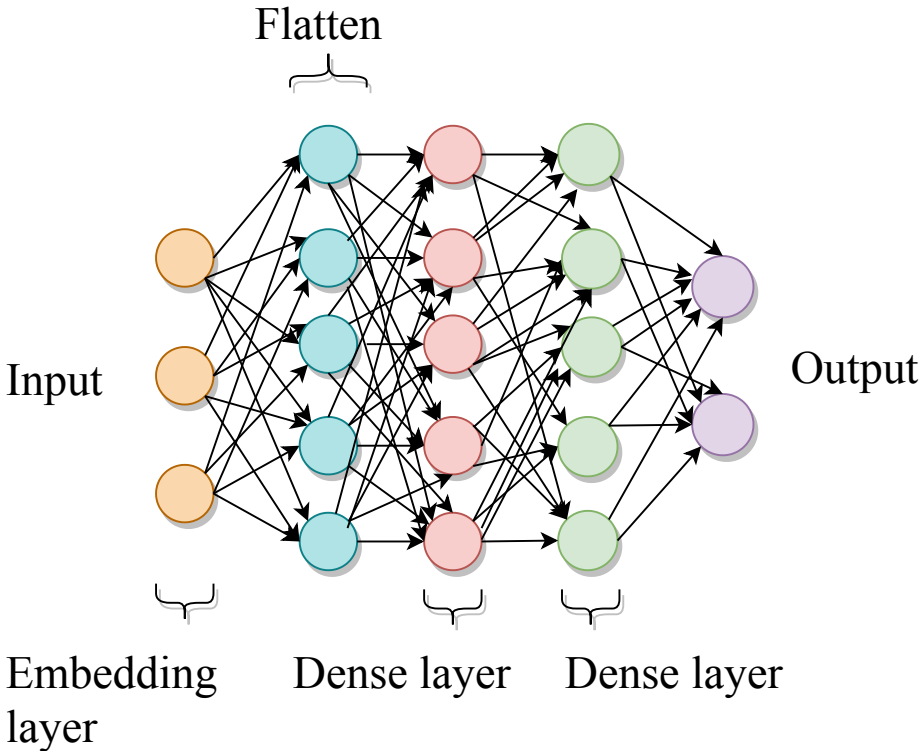

Supplement: giaa152_Supplemental_Files [file giaa152_supplemental_files.zip › supplementary_figure_2.pdf]

Mean frequency (before uniform sampling) of last tools in train tool sequences

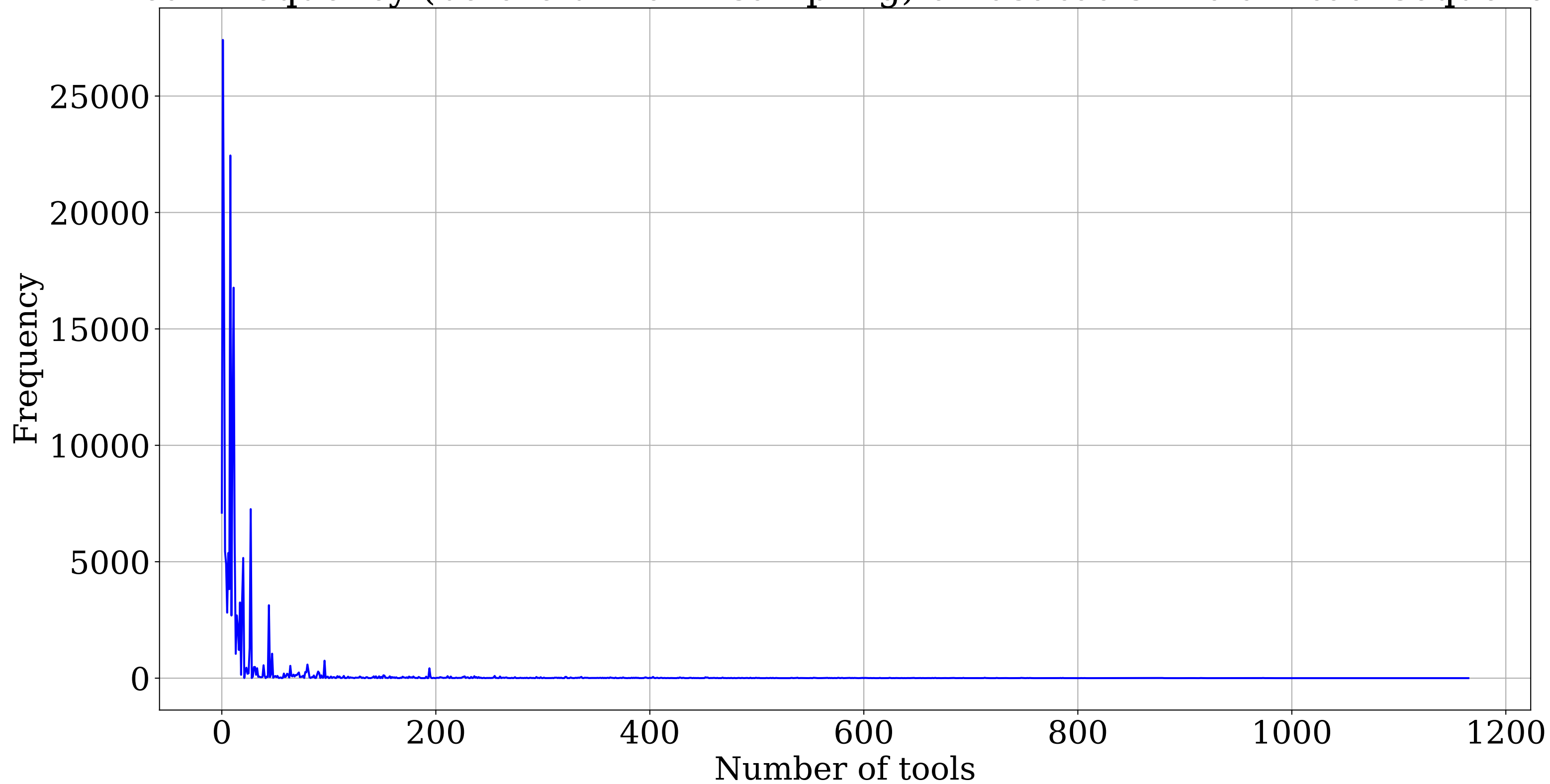

Supplement: giaa152_Supplemental_Files [file giaa152_supplemental_files.zip › supplementary_figure_3.pdf]

Mean frequency (after uniform sampling) of last tools in train tool sequences

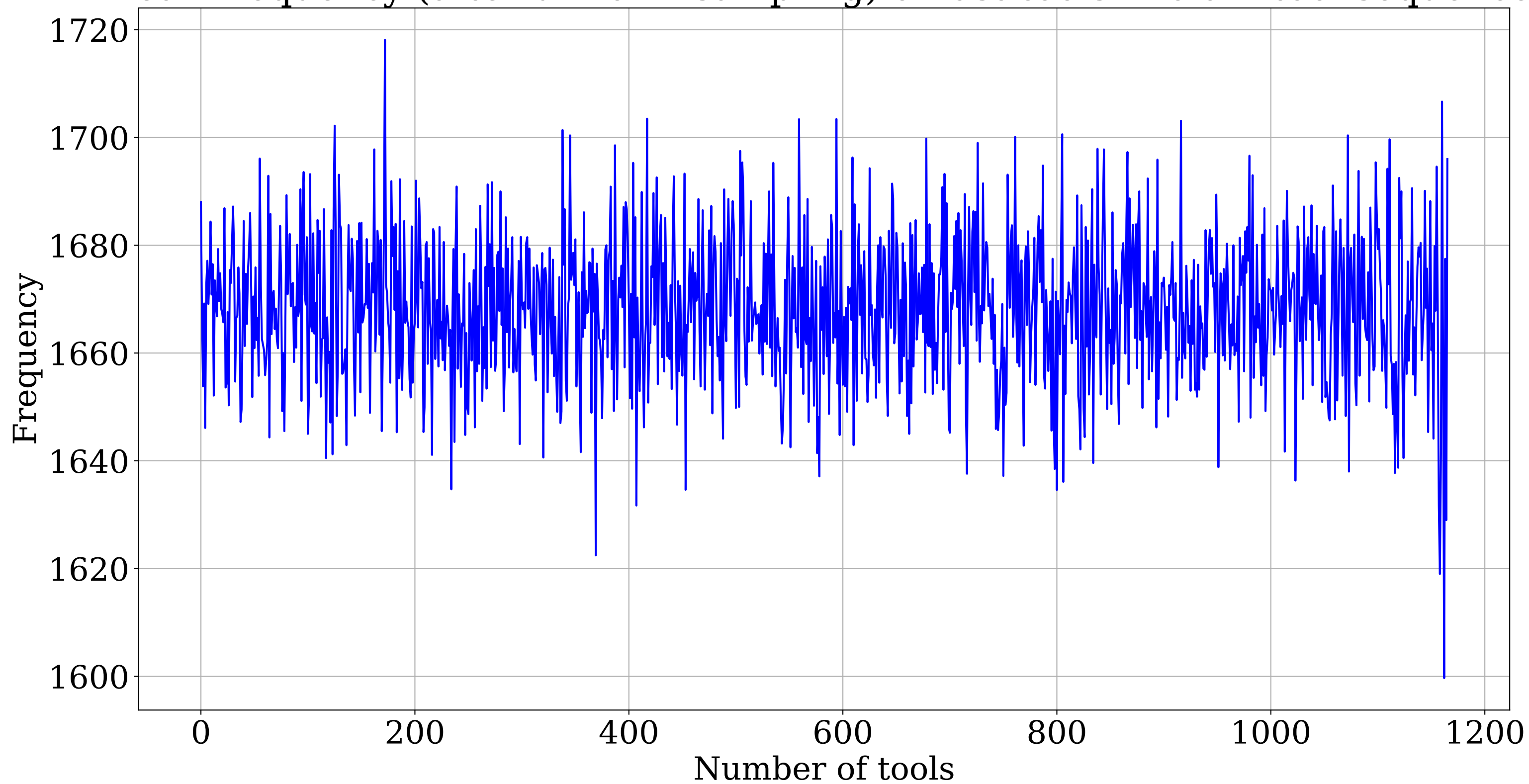

Supplement: giaa152_Supplemental_Files [file giaa152_supplemental_files.zip › supplementary_figure_4.pdf]

Mean non-shared precision@k vs frequencies of last tools

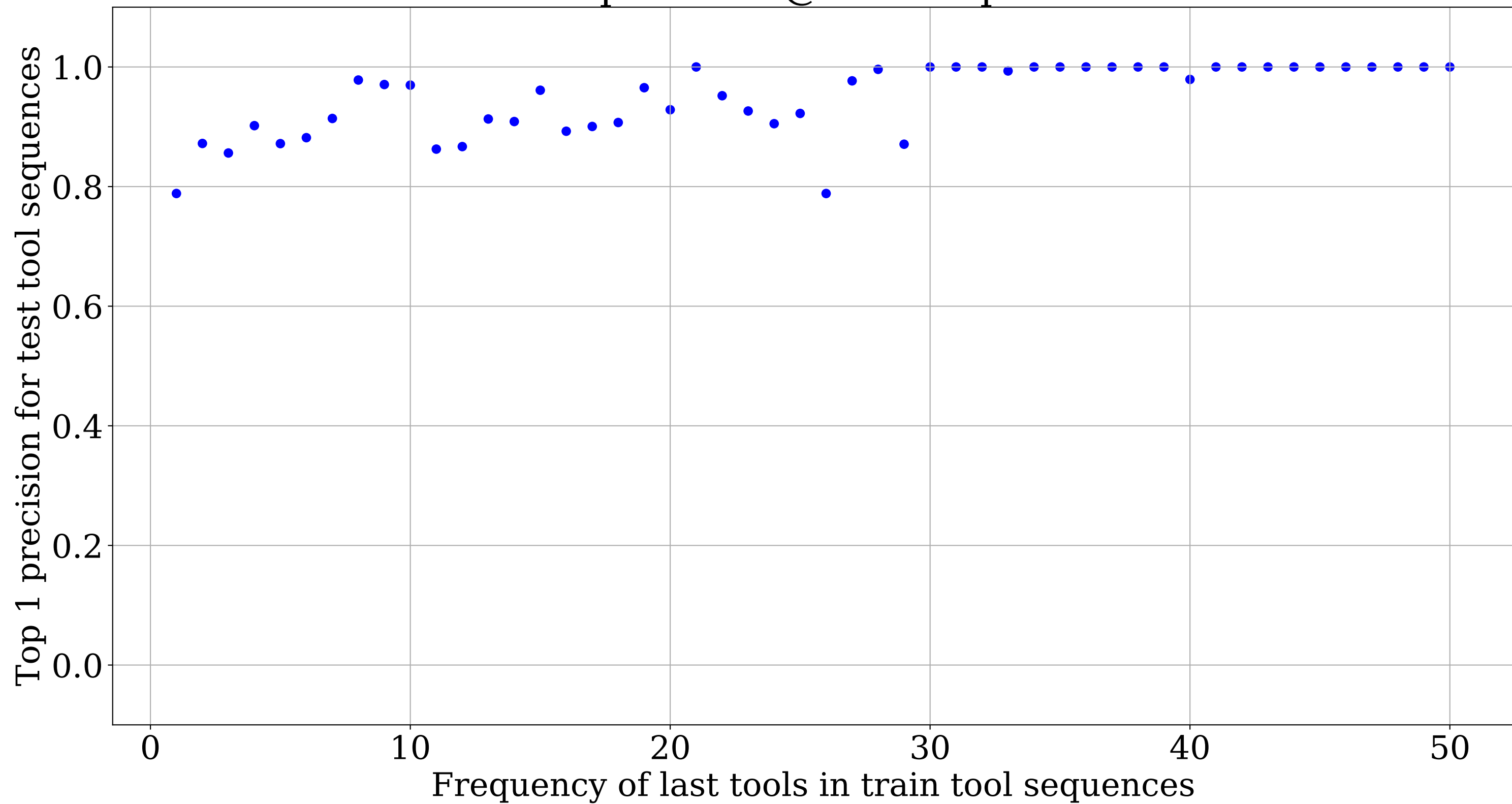

Supplement: giaa152_Supplemental_Files [file giaa152_supplemental_files.zip › supplementary_figure_5.pdf]

Mean shared precision@k vs frequencies of last tools

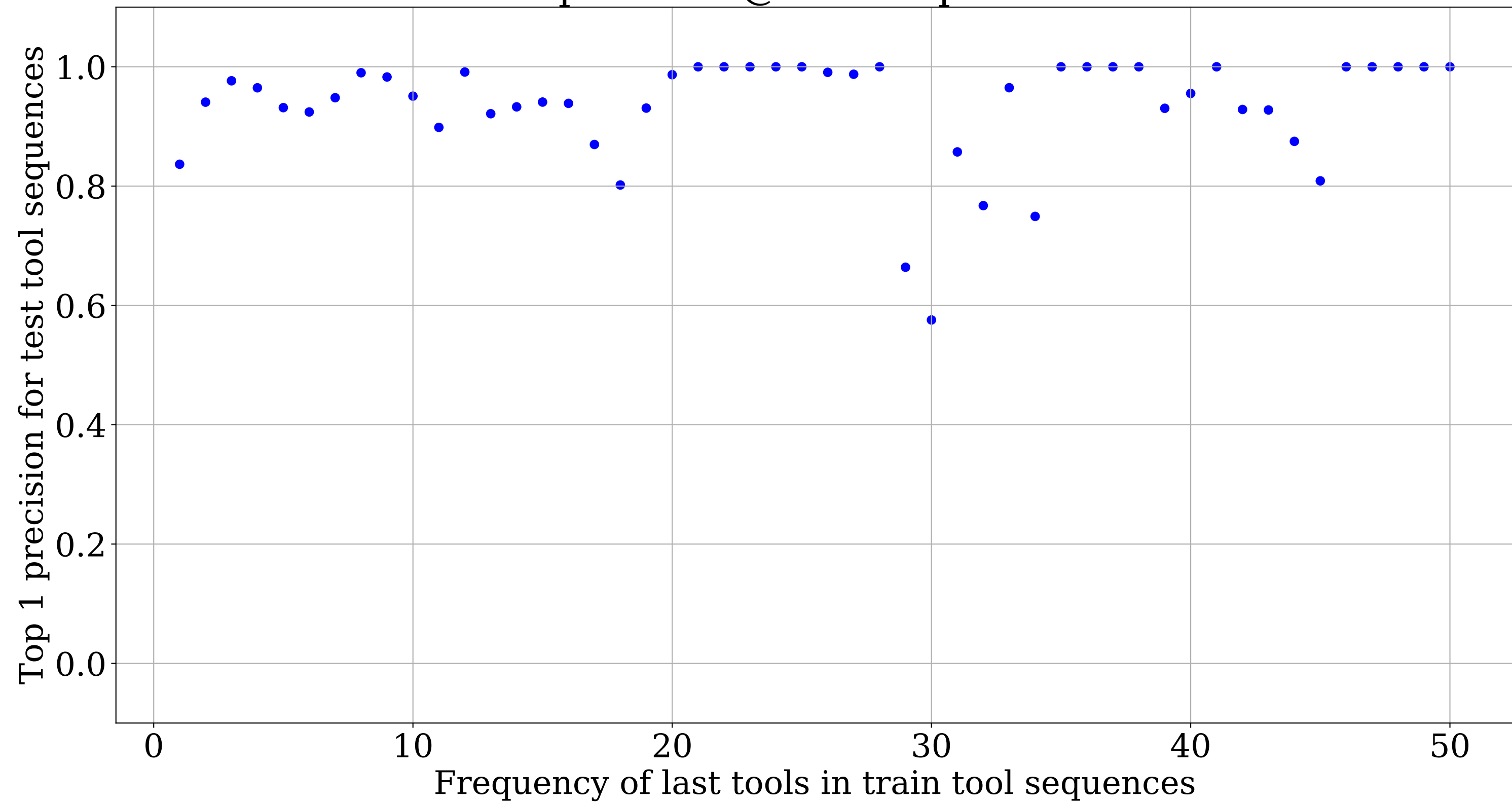

Supplement: giaa152_Supplemental_Files [file giaa152_supplemental_files.zip › supplementary_figure_6.pdf]

Non-shared and Shared precision@k using ExtraTrees classifier

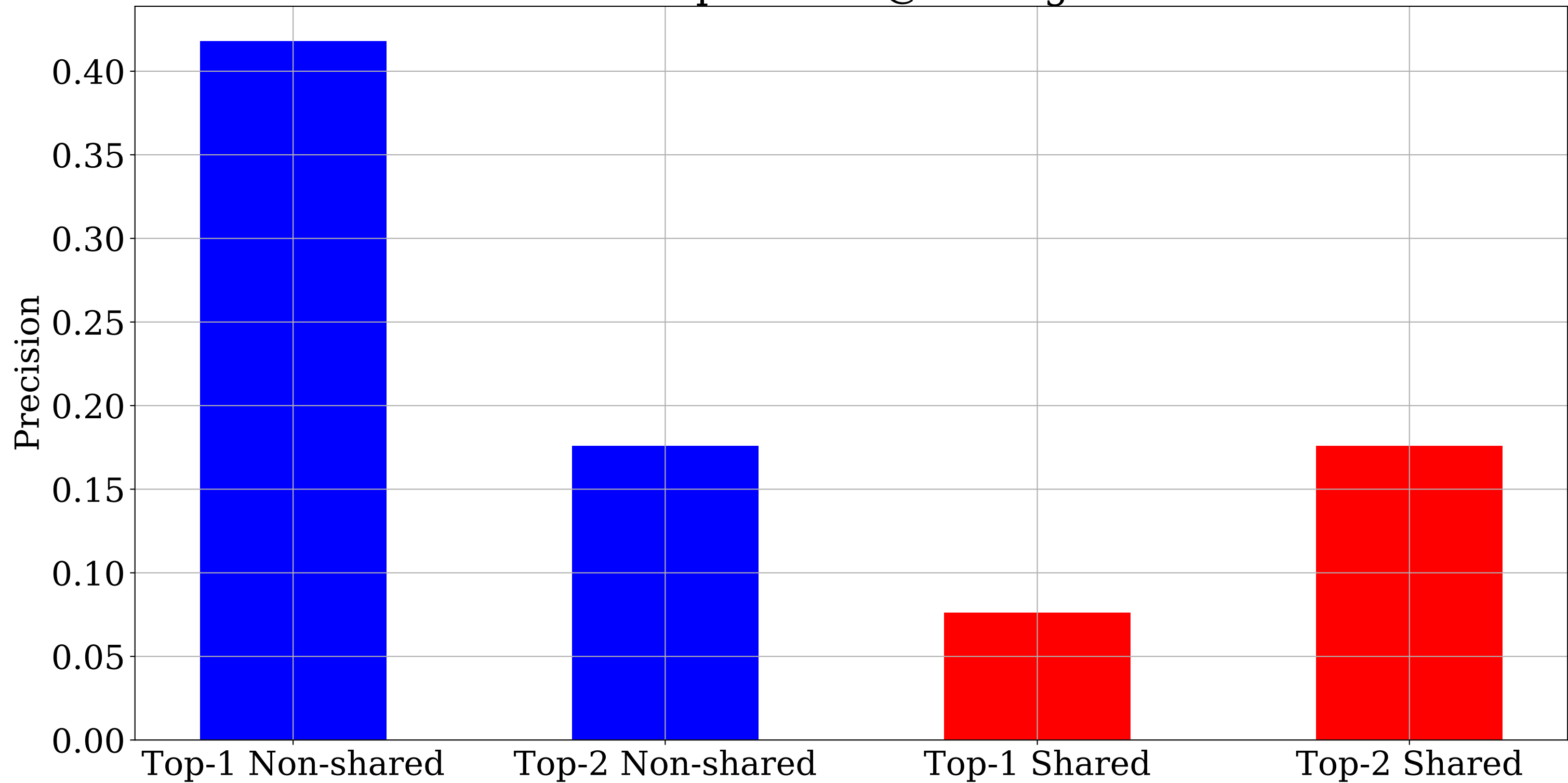

Supplement: giaa152_Supplemental_Files [file giaa152_supplemental_files.zip › supplementary_figure_7.pdf]
