# Supplementary material for: Tool recommender system in Galaxy using deep learning
Source: Gigascience. 2021 Jan 6;10(1):giaa152. doi: 10.1093/gigascience/giaa152 (PMC7786169; doi:10.1093/gigascience/giaa152)
Supplement: giaa152_Response_to_Reviewer_Comments_Original_Submission [file giaa152_response_to_reviewer_comments_original_submission.pdf]

Author's Response To Reviewer Comments

Close

GIGA-D-20-00053
Tool recommender system in Galaxy using deep learning
Anup Kumar; Helena Rasche; Björn Grüning; Rolf Backofen
GigaScience

Dear Anup,

Your manuscript "Tool recommender system in Galaxy using deep learning" (GIGA-D-20-00053) has been assessed by our reviewers. While it is of interest, we are unable to consider it for publication without some additional work. The reviewers have raised a number of points which we believe would improve the manuscript and would allow a revised version to be published in GigaScience. Particularly regarding the need for some real-world examples.

Author response:

We highly appreciate the suggestions provided by the editor and all the reviewers and have addressed all the review comments. A few sections have been added and modified in the paper and they are accordingly referenced in the responses. Moreover, we have provided real-world examples of tool recommendations for many tools and tool sequences from multiple scientific analyses in the paper (Table 1) and in the supplementary document (Supplementary table 1 and 2) in addition to figures 6 and 7.

Their reports, together with any other comments, are below. Please also take a moment to check our website at https://www.editorialmanager.com/giga/ for any additional comments that were saved as attachments.

In addition, please register any new software application in the bio.tools and SciCrunch.org databases to receive RRID (Research Resource Identification Initiative ID) and biotoolsID identifiers, and include these in your manuscript. This will facilitate tracking, reproducibility and re-use of your tool.

Author response:

We have received RRID (SCR_018491) for the project and also registered it on bio.tools (URL: https://bio.tools/tool_recommender_system_in_galaxy).

If you are able to fully address these points, we would encourage you to submit a revised manuscript to GigaScience. Once you have made the necessary corrections, please submit online at:

https://www.editorialmanager.com/giga/

If you have forgotten your username or password please use the "Send Login Details" link to get your login information. For security reasons, your password will be reset.

Please include a point-by-point within the 'Response to Reviewers' box in the submission system. Please ensure you describe additional experiments that were carried out and include a detailed rebuttal of any criticisms or requested revisions that you disagreed with. Please also ensure that your revised manuscript conforms to the journal style, which can be found in the Instructions for Authors on the journal homepage. If the data and code has been modified in the revision process please be sure to update the public versions of this too.

The due date for submitting the revised version of your article is 20 Jul 2020.

I look forward to receiving your revised manuscript soon.

Best wishes,

Scott Edmunds, Ph.D.
GigaScience
www.gigasciencejournal.com

Reviewer reports:

Reviewer #1: This paper describes a tool recommendation system for Galaxy workflows that employs a GRU neural network to train a classifier system. The classifier is trained on existing workflows that are recorded in the Galaxy system, and usage frequency of tools is additionally used to weight their relevance to the result.

Author response:

Thank you for your review comments. Please find their responses below.

This paper was submitted under the "Research" manuscript type, but I wonder whether it would have been better as a Technical note, given that the paper describes an implemented system, but there is no tested hypotheses or research question being answered.

Author response:

The manuscript has been reformatted as a technical note.

There are some interesting ideas explored in this paper but I have some major concerns about its current presentation:
- The solution seems like it might be overkill for the problem that is being solved. The authors have not implemented or compared to any alternative approaches to solving the same problem, so we have no basis to understand whether a NN is needed for this problem, or whether a simpler statistical model would suffice.

Author response:

We have compared the proposed approach with a simple model and ExtraTrees classifier and added the following text to the section S1 of the supplementary document which compares the performance of the proposed approach with these two other approaches.

“To compare the performance of the GRU neural network with approaches without any use of the neural networks, two ideas are explored - a simple approach to store all the indices of sequences of tools (https://github.com/anuprulez/galaxy_tool_recommendation/tree/statistical_model)
and the other one uses ExtraTrees classifier
(https://github.com/anuprulez/galaxy_tool_recommendation/tree/sklearn_rf).
The simple approach to recommend tools is implemented by storing the indices of tool sequences extracted from over 18,000 workflows on the European Galaxy server. The size of the resulting model (simple model) is 46 MB while its size created using the GRU neural network is only 6 MB. As the number of workflows grows in future, the model size will grow too, posing limitations in storing and sharing it over online platforms such as Galaxy. Therefore, to limit the size of the model, machine learning and deep learning methods are more suitable where it is not required to store any data. Instead, it is sufficient only to store the weights of features in data. Moreover, the GRU neural network recommends tools similar to the ones recommended by the simple model (Table 1 in supplementary section S2) for multiple scientific analyses. In addition, ExtraTrees, an ensemble based classifier, is also

trained on tool sequences to recommend tools. The optimisation of hyperparameters such as the number of estimators, depth of trees and so on and the uniform sampling of the training data were done following the same approach as used for training the GRU neural network. The model trained with the ExtraTrees classifier achieves a precision of less than 0.5 for the top-1 metric (Supplementary Figure 7) which is very low compared to the accuracy of the GRU neural network (0.98 top-1 precision). It shows that the ExtraTrees classifier is unsuitable for learning on sequential data as used in this project. Moreover, the peak memory usage for the ExtraTrees classifier is approximately 75 GB while for the GRU neural network it is approximately 8 GB. In summary, large model size of the simple model and low precision and high memory usage for the ExtraTrees classifier do not qualify them as good approaches to create the tool recommendation system. On the other hand, the GRU neural network has a smaller model size and memory usage and higher precision which collectively make it a better approach to create the tool recommendation system."

- The paper does not address the issue of whether the tool is really useful in practice. For example it says "they [recommendations] improve user experience by helping researchers to easily create correct workflows". This claim is not tested in this paper. You would need to test user behaviour to find out, or in the very least, survey user experience with the tool.

Author response:

Table 1 in the paper lists a few standard (tool connections in published and non-deleted are marked as standard and they are of good quality) and normal (tool connections from other workflows) recommended tools for multiple tool sequences in different scientific analyses such as Computational chemistry, Epigenetics, Machine learning, Proteomics, RNA sequencing analysis and a few others. The recommended tools shown in this table are highly used for standard scientific analyses as highlighted in multiple GTN training materials (https://training.galaxyproject.org/) which shows that the recommended tools are of good quality and highly used. Moreover, this project has been deployed on the European Galaxy server for the last 12 months and has received very positive user-feedback since then. It was presented as a live demonstration at the Galaxy community conference (GCC) 2019 and at the Automatic workflow composition workshop (March 2020). At both events, positive feedback was received. In addition, the code has been accepted upstream by the Galaxy community and is already part of the main Galaxy codebase (release 20.05) because it was considered to be an important addition for the Galaxy users. A couple of reviewers of this paper seem to have tested it and found it to be good and intuitive. However, there is no easy way to monitor user's behavior regarding the usefulness of recommendations. We are in contact with the cognitive science department of our university, who are supporting us with the user experience (UX) questions. In summary, we are confident that such a recommendation system can assist Galaxy users in choosing good quality tools for different analyses.

- Such a recommendation system us potentially dangerous if it is giving poor/biased/incorrect suggestions to users, and is highly sensitive to the training data. It is not clear from the paper how biases in the training data are dealt with. For example, suppose the Galaxy server is used by students undergoing training. It could be the case that for training purposes students are taught initially to use older, out of date, tools and techniques, for the sake of simplicity. There could be very many of these students on the system. A large number of students using out-of-date techniques could seriously bias the results. The paper suggests re-training the system periodically, which is reasonable, however, how does the system protect against circular dependencies in the data, where it starts training on workflows that have used the recommendation system themselves?

Author response:

We have divided the tool connections into standard and normal connections. Standard connections are tool connections coming from the published and non-deleted workflows which are of good quality while the normal ones are from other workflows. Recommendations are also divided into standard and normal ones and the standard ones are shown at the top of the list of recommendations. We achieve high accuracy (top-1 precision = 0.98) for the standard and normal recommendations. Table 1 in the paper suggests that the system predicts useful tools which appear in diverse standard scientific pipelines mentioned in Galaxy training materials. These materials use the latest tools which are used for training purposes around the world and they are regularly maintained. To avoid old tools appearing in the list of

recommendations, we have implemented a whitelist (overwrite.yml) where Galaxy admins can overwrite deprecated tools. Training data (workflows) can get biased, for example, when lots of students are using old tools to create workflows for multiple scientific analyses as asked in the review. Therefore, to minimise the problem occuring due to the biases, we performed uniform sampling of the data before feeding it to the neural network. "Uniform sampling" section has been added to the paper. Please find the corresponding text from the paper below.

"Workflows in Galaxy come from different scientific analyses. It may happen that the number of workflows from these analyses are not comparable - some analyses may have a large number of workflows while some may have only a small number of workflows. This can cause some tools to be present very frequently in workflows while other tools are less frequent. Learning on these workflows and recommending tools may exhibit bias by showing better recommendations for the frequently occurring tools and poorer recommendations for the less frequent ones. To showcase this imbalance, the frequencies of the last tool in each tool sequence in training data are calculated and it is found that only a few tools have large frequencies and most of the tools are present in low frequencies (Supplementary Figure 3). For example, the tools with very high frequencies (> 10,000) are "Concatenate datasets", "Cut", "Grouping" and "Join" while the tools having very low frequencies (< 5) are "Cluster inspection using RaceID", "rDock cavity definition" and "ChiRA collapse". Therefore, to overcome this drawback, the training data created after extracting tool sequences should be balanced in order to make the neural network learn on a similar number of tool sequences from different scientific analyses in each training iteration. To implement this strategy, a set of last tools in all tool sequences from the training data is collected. Further, for each tool in this set, a list of indices of tool sequences in the training data are stored for which it is the last tool (Supplementary Table 3). Only the last tools are considered for implementing this strategy because of two reasons. First, the smallest tool sequences contain only two tools and second, all tools become the last tool in at least one tool sequence and the computed frequencies of these last tools suggest their overall frequencies in the training data. In the neural network training, for each iteration (which consumes all tool sequences in the training data), small batches containing equal number of tool sequences are created. For example if batch size is 100 and the size of training data is 2000, then 20 (2000/100=20) batches are created, each containing 100 tool sequences. In each batch, 100 tools from the set of last tools are uniformly selected (Column 2 in Supplementary Table 3) and for each selected tool, a tool sequence is chosen uniformly from its respective list of tool indices (Column 3 in Supplementary Table 3). After selecting tool sequences for many batches for each iteration of training (epoch), it is expected that all the tools from the set of last tools and their respective tool sequences are chosen. Performing this uniform selection of different tool sequences for each iteration, the training data becomes balanced. Supplementary Figure 4 shows that each last tool is present approximately 1670 times on an average in each iteration (epoch). The order of tools in the Supplementary Figures 3 and 4 are same."

Due to the uniform sampling, good precision has been achieved for the tool sequences with low frequencies too. In summary, the recommendation of good quality and highly used tools should mitigate the problem of circular dependency. Please see section S5 of the supplementary document for more details.

- The system considers tools, but apparently not data types. In the example discussed on page 4, how would the system know to recommend RNA-STAR compared to BWA or Bowtie if it doesn't know whether the inputs are DNA or RNA?

Author response:

Currently, Galaxy does not differentiate between RNA and DNA as both are FASTA files. This behaviour is in Galaxy and does not arise from the tool recommendation system. If there are tools such as RNA-FASTA or DNA-FASTA in Galaxy, this issue would be solved. However, the recommendation system uses the knowledge of data types of tools to filter out the incompatible recommendations as a post-processing step (after recommending tools using the trained model). The recommendations shown in the Galaxy UI for a tool or a sequence of tools have compatible data types with the last tool it connects to. The recommendations with incompatible data types are not shown.

Beyond those concerns above, I feel that the paper is imbalanced in its structure. Too much space is used to discuss general features of NNs, whereas too little attention is paid to the actual methods used

in the project. For example, the actual size and nature of the training data is only very briefly mentioned near the end of the paper "The number of tool sequences extracted from workflows is approximately 200,000..." This is a very important detail that deserves more attention. We don't know the proportions of the training workflows for different kinds of analyses, and therefore it is hard to say anything about selection bias in the data. What if 90% of the training data is for DNA sequencing, how would this affect the ability of the system to recommend proteomics tools for example?

Author response:

The “Data description” section in the paper has been extended to include information about workflows. In addition, we have added a “Uniform sampling” section in the paper that explains how the imbalance in the data is solved. More details about uniform sampling are given in section S5 of the supplementary document. Uniform sampling makes it possible that the neural network trains on tool sequences from workflows in multiple scientific analyses in roughly equal number of times. Therefore, even if 90% of training data is for DNA sequencing and other analyses form only 10% of all data, the neural network will train on different sequences of tools almost equally. Supplementary Figures 5 and 6 show that the precision (standard and normal) achieved for the less frequent tool sequences are also good (90%). In addition, every Galaxy instance will have its own set of tools and workflows. Therefore, it has been made possible to create different recommendation models ideal for different Galaxy instances by using a Galaxy tool - “Create a model to recommend tools”.

Reviewer #2: The authors present a Galaxy tool recommendation system based on a training set of over 200,000 tool sequences from existing workflows. The final version of this system uses a GRU neural network. Although extensive testing was done to compare and benchmark this model with other neural networks, it should be noted there is no existing conventional approach (Markov Model/market basket/Bayesian) but I don't feel that it is necessary to create a straw man for this purpose. The neural network offers a high degree of accuracy without the need to manually annotate each tool.

The description of the GRU implementation, testing, and optimization is very good and will inform readers about how to implement similar solutions for other applications. The Github repository is also well organized. This paper is well suited to this journal and should be accepted with minor revisions.

Author response::

Thank you for the appreciation and for your review comments. Please find their responses below.

I have three minor revisions to suggest:
1. I think most readers would understand what overfitting would look like in a typical machine learning, but maybe not in a recommendation engine for tools. What noise or error that is propagated by an overfit recommendation engine? For instance, would it resemble obscure tools that some edge case user chose? More importantly, what does the regularization step actually do in this case - recommend a repertoire of more common tools, or simply remain agnostic?

Author response:

The following text has been added to section S4 of the supplementary document.

The use of dropout in the GRU neural network for regularisation allowed the model to have smaller weights compared to the non-regularised GRU neural network model. Overall mean of weights (weights of all layers) for the regularised model is 0.0755 while for the non-regularised model, it is 0.0962. Having larger weights in a neural network model may lead to overfitting as it is an indication of a more complex network. In an overfit model, the neurons (neural network units) in a layer try to fix the errors made in the previous layers to make the model robust (on the training data) and may not learn more general features. One of the advantages of using regularisation in a recommendation engine can be to avoid the following situation - an overfit model may try to learn the most common tools with higher usage frequency to minimise the error (due to the weighted cross-entropy loss function) and may ignore tools with lower usage, but important ones. For example, the regularised model recommends the

following tools for the “UMI-tools count” tool. The (log) usage frequency has been shown beside each tool name.

-Text transformation with sed (3.98) [Standard recommendation]
-Column Join on Collections (4.78) [Normal recommendation]
-Initial processing using RaceID (3.68) [Normal recommendation]
-Transpose rows/columns (3.57) [Normal recommendation]
-Seurat (2.67) [Normal recommendation]

The non-regularised model also recommends the same tools except “Seurat” tool which has the lowest usage frequency out of all recommended ones.

Provide a real-world example, something like…

"An example of overfitting in our recommendation system may involve tools with few use cases (e.g. tools for dealing with organisms with no reference assembly). The GRU would learn only from a limited training set and which could recommend tools that would not be appropriate for most users."

Author response:

We have added a table comparing the recommendations made by the regularised and non-regularised models to the section S4 of the supplementary document. Please find the relevant text from section “Benefit of regularisation” in the paper below.

“Using regularisation minimises overfitting by assisting the GRU neural network to make better recommendations by predicting tools having low usage frequencies but useful in addition to tools with high usage frequencies. For example, the recommendations of "UMI-tools count" tool with the regularised model include "Seurat" tool which is absent from recommendations by the non-regularised model. Another example is for "RaceID, Lineage computation using StemID" tool sequence which gets "Lineage Branch Analysis using StemID" tool as one of the recommendations by the regularised model while there is no recommendation at all by the non-regularised model. The recommendations for a popular mapper, RNA-STAR, are "featureCounts", "MultiQC", "Infer Experiment" and few others by both the models. But, in addition to these recommendations, the regularised model recommends the "Read Distribution" tool which is not predicted by the non-regularised model. More details have been provided in section S4 of the supplementary document.”

2. It appears the recommendation engine cannot recommend entirely new tools without completely rebuilding the model. Please explain how often the model is rebuilt in practice. Also explain how new tools would ever be recommended if they don't appear in existing workflows. Is the design that entirely new tools would be adopted into workflows by "power users" and therefore trickle down to more casual Galaxy users?

Author response:

The recommendation model for usegalaxy.eu is currently created periodically every 3-4 months to accommodate new workflows and tools. Galaxy admins can decide upon the frequency of creating a new model. It can be created every month or every 6 months. In addition, there is a provision to add and overwrite tool recommendations in the Galaxy API by Galaxy admins to promote newly added tools and deprecate old tools, respectively for all Galaxy users including power and casual ones. We created a tool to make it easier to recreate the training model with the latest tools and workflows by creating a Galaxy tool - “Create a model to recommend tools”. Using this tool, a new recommendation model can be created after collecting workflows and tool usage data from any Galaxy server. The tool runs for several hours (> 24 hours) and creates the recommendation model. It can be downloaded from Galaxy and uploaded to Galaxy’s test data repository (https://github.com/galaxyproject/galaxy-test-data) by Galaxy admins. From this repository, Galaxy downloads it using the API to recommend tools.

3. DESeq2 is miscapitalized as DeSeq2 on pg2

Author response:

Thank you for spotting this mistake. We have corrected it in the paper.

Reviewer #3: This paper describes an interesting and novel approach for learning from the large numbers of existing workflows in Galaxy. Using a neural network approach to learn which tools are used most frequently, and which tools are used in sequence, is a good basis for building a recommender system. The number of available Galaxy workflows provides a large test set to work with and spans a wide range of bioinformatics analyses. The recommender has already been embedded into the Galaxy.eu server. This increases the significance of the work as it means that a large user community can already benefit from the recommendations.

Author response:

We appreciate your positive feedback and thank you for your review comments. Please find their responses below.

Major Points

1. The paper would benefit from a deeper discussion about the quality and validity of the workflows in the test set. Are all workflow used, or only those that run without generating an error? There are many workflows created during exploration that are never used for an eventual analysis, either because they are considered unsuitable by their creator, or because they simply did not run. How are these 'test' workflows separated from 'production' workflows that are used to generate actual results?

Author response:

We have collected over 18,000 workflows from the European Galaxy server and extracted over 229,000 paths (sequences of tools) from them which are used for training a neural network (Gated recurrent units). All these paths are divided into training and test sets. The training set is used for training and the trained recommendation model is evaluated on the test set. To address the quality of the workflows, the connection of tools within workflows are separated based on their quality - the connections coming from the non-deleted and published workflows are marked as “standard” or “good” connections and the rest of all other tool connections are marked as “normal” connections. The recommendations are also separated as standard and normal. Standard recommendations (good quality) are promoted to the top of the list of recommendations followed by normal recommendations. In addition, the recommendation model can be created ideal for different Galaxy instances by using respective tools and workflows by Galaxy admins. Moreover, a whitelist of tools can be provided by Galaxy admins using the config option to overwrite the recommendations made by the neural network if needed.

2. Related to the point above - is there an attempt to collect and learn from tool parameter information in addition to the tool function? Some tools perform multiple functions and therefore behave very differently depending upon their configuration.

Author response:

Currently, we are recommending tools based on tool connections in workflows. In addition to learning from tool connections, recommending tools based on the configurations of tools would be in our future enhancement of the tool recommendation system and it has been mentioned in the “Summary and future work” section of the paper too.

3. The recommender system is based on suggesting the next tool, but could the recommender system

also be used to suggest whole configured workflows as the next step? If a collection of tools are always used together, suggesting one at a time (without parameter information) would be less accurate overall than suggesting a fully configured workflow for all the subsequent steps.

Author response:

Recommending a set of tools at each step provides more flexibility to users to diverge into multiple analyses using different tools enabling users to explore many different tools. However, predicting complete workflows may lose many different variations in which a tool can be used. Moreover, there is already a provision for sharing workflows in Galaxy. Also, many workflows are already available for multiple analyses over Galaxy Training Network (https://training.galaxyproject.org/). It may be possible to recommend complete workflows, but currently we do not aim to predict complete workflows in this work.

4. Recommendations are based on tool use frequency data and previous workflows. If a new tool is developed, there will be a lag before this tool is recommended to users, even if it is 'better' than similar tools. How could this problem be addressed? Similarly, using recommendations may lead to less innovation in workflow creation. Could the authors discuss the implications of this (i.e. who is the recommender for? Is it for the workflow expert, or for users who need more bioinformatics support?)

Author response:

Galaxy admins can overwrite the recommended tools predicted using the trained model by a different set of tools using a configuration option by adding different tools. In addition, newly added tools, which are not part of the model, can be appended to the recommendations using this additional configuration option. Moreover, every 3-4 months, a new recommendation model trained on the latest data will be made available on the EU Galaxy server. Galaxy admins can decide upon the frequency of creating a new model. It can be created every month or every 6 months. Admins of each Galaxy instance can create a recommendation model built using the tools and workflows on that instance. The frequency of model creation can also be managed by Galaxy admins. The question of implication of the recommendation system is briefly discussed in the “Summary and future work” section of the paper. Please find the relevant text from the paper below.

“The recommendation system should be potentially helpful for those researchers who are new to the Galaxy platform. It shows them a few follow-up tools from a big collection of more than 3,000 tools and enables them to perform multiple exploratory data analyses.”

6. Have the authors performed a user evaluation on their recommender system? It is already part of Galaxy.eu and the manuscript claims it "improves user experience by helping researchers to easily create correct workflows". However, there are no details of current usage or of how this has been assessed to date. As it is available, I have used it, and it is very intuitive, but the paper would be strengthened by adding a more formal evaluation of usage to date.

Author response:

This project has been deployed on the European Galaxy server for the last 12 months and has received very positive user-feedback since then. It was presented as a live demonstration at the Galaxy community conference (GCC) 2019 and at the Automatic workflow composition workshop (March 2020). At both events, positive feedback was received. In addition, the code has been accepted upstream by the Galaxy community and is already part of the main Galaxy codebase (https://github.com/galaxyproject/galaxy/tree/release_20.05) because it was considered to be an important addition for the Galaxy users. Table 1 in the paper suggests that the recommendation system predicts useful tools which appear in diverse standard scientific pipelines mentioned in Galaxy training materials (https://training.galaxyproject.org/). These materials use the latest tools which are used for training purposes around the world and they are regularly maintained. However, there is no easy way to monitor user’s behavior regarding the usefulness of recommendations. We are in contact with the cognitive science department of our university, who are supporting us with user experience (UX)

questions. In summary, we are confident that such a recommendation system can assist Galaxy users in choosing good quality tools for different analyses.

Minor point

In the results section, the example workflow is based on NGS sequence analysis (Trimmomatic --> Bowtie2 --> FreeBayes). This is a good example, however, in the text, the authors suggest that after bowtie2, FastQC might be recommended. Usually, FastQC would be used before Trimmomatic.

Author response:

Thank you for spotting it. We have updated the text in the paper.

Reviewer #4: Dear authors and editor(s),

After seeing a conference presentation and the tool recommender in usegalaxy.eu last summer, and noting the bioRxiv preprint of this work last November (https://doi.org/10.1101/838599), I am happy seeing that authors submitted the manuscript to GigaScience. Compared to the preprint, the text has been polished and better structured into sections.

I find the manuscript very nicely written and relatively easy to read (with only minor opportunities for editorial polishing, some mentioned below). As a reader, I highly appreciate the effort of the authors towards making the text reader-friendly. I find it also clear from the text, how the data was collected and curated, and where it is openly published. The manuscript also points to the used source code (scripts), and all is available together under a permissive open source software license.

I did not have the capacity to test the various scripts - for extracting data and all of the models training - but I repeatedly tested the user interface on the usegalaxy.eu server, useful from the user's point of view.

I'm very happy with the content and the results of this work, and the manuscript convinces me about the choices taken in the authors' approach. I don't recommend a revision of the work. My following suggestions are only about the code repository and some minute, mostly editorial improvements of the text, plus a couple of brief comments.

Author response:

We appreciate your positive review and thank you for your review comments.

Repositories and branches:

This manuscript links to a repository containing all the used code and data (https://github.com/anuprulez/galaxy_tool_recommendation), created in February 2020 by a mass upload of most of the code and data. I am really happy to see that some updates have been pushed to this repo in the recent weeks of April 2020.

The history of the work is recorded in another repo (https://github.com/anuprulez/similar_galaxy_workflow), with various branches active last year, a separate master branch subsequently active in Sep and Oct, and a 'tool_recommendation_release_19_09' branch active aftewards between Oct 2019 and Feb 2020. It would be nice to note the history and the relations between these repos and branches somewhere in the beginning of all the involved README.md files, especially so that the community sees where to look for the active work and where not (and eventually fork, experiment, contribute, start issue threads, etc.).

Author response:

We have added a note in the README.md file explaining the old and new repositories. Initial work to

create a tool recommendation model is stored at https://github.com/anuprulez/similar_galaxy_workflow. This repository storing the history of work until October 2019 will not be used in future. The current repository (https://github.com/anuprulez/galaxy_tool_recommendation) will be used for current and future developments. Further, the information about different branches has been added to the readme file.

It would be a nice bonus to have the version used in the article tagged as a release on GitHub, ideally with a Zenodo archive DOI (https://zenodo.org/oauth/login/github/?next=%2Fdeposit followed by https://zenodo.org/account/settings/github/), which could be permanently linked from the published article.

Author response:

We have added the zenodo link (https://zenodo.org/record/3885595#.Xt5uuzczY5k) to the code repository
(https://github.com/anuprulez/galaxy_tool_recommendation).

Notably, the README.md currently contains useful information on how to reproduce the work, and use it on another Galaxy server. I would also welcome in the near future some additional brief information about this project, and explicitly separate "how to-s" for various types of usage/users: Galaxy end users, Galaxy admins, researchers who want to reproduce the work, existing contributors to the project, and potential new contributors. CONTRIBUTING.md might be a good option for the latter or latter 2; and/or README.md can include the overview of all the information, with further links to potential other files with details.

Author response:

We have added a CONTRIBUTING.md (https://github.com/anuprulez/galaxy_tool_recommendation) file explaining how to contribute to this repository. We have also updated the README.md file which contains information about how to reproduce the work, how to enable this feature in Galaxy for admins and how to use/see recommendations for Galaxy end users.

Abstract:

The abstract of the manuscript is nicely concise and gives a good summary of the work. I suggest it could possibly be made even easier to read and understand (especially for unacquainted readers), with a couple of small changes.

"To make creating workflows easier, faster and less error-prone, a predictive system is developed to recommend tools facilitating further analysis." could be simplified into something in the sense of "To help (users) with creating workflows, we developed a system to recommend tools that (would) facilitate further analysis."

Author response:

We have changed the sentence to:
“To help researchers with creating workflows, a system is developed to recommend tools that can facilitate further data analysis.”

"A model is developed to recommend tools by analysing workflows, composed by researchers" - without comma, or comma after 'tools' would read smoother.

Author response:

We have changed the sentence to:
“A model is developed to recommend tools using a deep learning approach by analysing workflows

composed by researchers on the European Galaxy server.”

"precision@1, precision@2 and precision@3 metrics" - would it be clearer with e.g. "top-one, top-two, and top-three metrics", or not?

Author response:

We have updated the names of metrics to top-1 and top-2. Now, the sentence reads as:
“Mean accuracy of 98% in recommending tools is achieved for the top-1 metric.”

The Conclusion(s) part is hard to read. The preprint included a sentence similar to "The model is accessed by the Galaxy API to recommend tools in real time.", which could perhaps fit in. Subsequent "Multiple user interface (UI) integrations on the European Galaxy server communicate with an API, which accesses the model, to apprise researchers of recommended tools in an interactive manner." could be simplified - especially if the previous sentence from the preprint is included in some form - into e.g.: "Multiple user-interface integrations on the European Galaxy server communicate with the(?) API, which accesses the model, to provide researchers with recommended (next?) tools, in an interactive manner." The last sentence would be clearer with something like "The scripts to create the recommendation model, and the (sample) (training) data (we used), are available under MIT License at ..."

Author response:

We have updated the text as follows.

“The model is accessed by a Galaxy API to provide researchers with recommended tools in an interactive manner using multiple user interface (UI) integrations on the European Galaxy server. Good quality and highly-used tools are shown at the top of the recommendations. The scripts and data to create the recommendation system are available under MIT license at https://github.com/anuprulez/galaxy_tool_recommendation”

Main text:

The manuscript is excellently written, however, including some opportunities for minor improvements to smoothen some of the sentences. These include unusual or ambiguous words ('stemming', 'appropriately', the latter can be omitted); some punctuation (I find some missing commas, but appreciate nicely and correctly used hyphenation); and minor grammar nuances, e.g. "Galaxy is a(n) open-source data-processing platform...", or "time spent (in)".

Author response:

Thank you for the appreciation.

I also included a couple of positive comments among the following notes.

[Introduction]

"... to ensure it, a system is needed which can recommend correct tools while creating a workflow." - both 'ensure' and 'correct' are too strong (restrictive) words in this context, claiming way too ambitious goals.

Author response:

We have updated the text as follows:
“To make it possible, a system is needed which can recommend useful tools at each step while creating a workflow.”

"has a defined number of data types for these input and output files" - clearer would be something like "supports a number of formats of these input and output files", in case it is only the format that is the point here. (The use of 'data types' in what follows after is fine, unless the authors would want to be more precise (but verbose) with types of data and data formats. However, I don't suggest that it is necessary here. Still, one may want to take into account that outside of Galaxy, 'data type' relates to types in (typed) programming languages for programmers, and to acquisition technologies and techniques in the jargon of many biologists and medical researchers.)

Author response:

We have updated the text as follows:
“A tool consumes one or more data files as input and produces one or more data files as output and supports a number of formats of these input and output files.”

"Second, it will help them bypass the step of searching for tools separately which shows potential to further reduce the time spent in creating workflows and increase the accessibility of tools." - For me personally, this is where I see the biggest benefit of the recommender system (bigger than the "First", 'correctness' use case). Maybe some smoothening of the sentence, e.g. "Second, it will help users bypass the step of searching for tools separately, what will further reduce the time spent in creating workflows and (at the same time) increase the accessibility of tools."

Author response:

We have updated the text as follows:
“Second, it will help researchers bypass the step of searching for tools separately, which will further reduce the time spent in creating workflows and at the same time increase the accessibility of tools.”

"Finally, it can also be used to promote the newly added tools in Galaxy by showing them alongside the recommended tools predicted using the deep learning approach." - This is really important and great!

Author response:

Thank you for your appreciation.

"metadata of tools" might be clearer with "information about tools". Also in Discussion.

Author response:

We have updated the text as follows:
“First, it does not require collecting and storing information about tools.”

"higher-order relationships among tools" are explained in Figure 1. The explanation is sufficient, and aided by the figure, but perhaps it would then be nice to add a link to the figure where they are mentioned the first time (except for the abstract).

Author response:

We have updated the text as follows:
“Second, it takes into account the higher-order relationships among tools (Figure 1) in tool sequences.”

"A Bayesian network can also be used..." perhaps a new paragraph?

Author response:

We have addressed it in the paper.

[Results]

"the next generation sequencing (NGS) data" could just be "the sequencing data".

Author response:

We have addressed it in the paper.

The recommended tools mentioned in the text following right after do not match with the recommended tools in Figure 5. It would be nice to explain what those in Figure 5 are, or match them with the text. Perhaps an easy UX fix: Display which step the recommended tools are for.

Author response:

We have updated the caption of the image to describe its contents. Moreover, we have updated the text in the "Examples of tool recommendations" section describing this image.

Figure 6 description: RNA-STAR is usually spelled with all capitals, as in the main text.

Author response:

We have addressed it in the paper.

[Discussion] (Reading the content, it could also be titled "Summary and final remarks".)

Author response:

We have addressed it in the paper and updated the name of the section to "Summary and future work".

"A recommender system to predict tools in Galaxy..." - Isn't it a "prediction system to recommend tools in Galaxy" instead? What about simply "A system to recommend tools in Galaxy..."?

Author response:

We have addressed it in the paper.

"in real time."

Author response:

We have addressed it in the paper.

The last 2 sentences could be part of a separate paragraph on "future work"/"future options", perhaps slightly more elaborate. For example, any UI ideas on how to suggest both highly-used and new tools transparently, and how to administer these (automatically?)? Or would it be worth experimenting with the inclusion of tools similarity, especially if these are in the future better annotated with semantic information?

Author response:

We have addressed it in the “Summary and future work” section. Please find the relevant text from the paper below.

“Different Galaxy servers maintain different sets of tools and workflows. The current approach can be used to create different recommendation models for different Galaxy servers. Alternatively, all the workflows can be collected from multiple Galaxy servers and using the current approach, one recommendation model can be created by learning on the complete set of workflows and the model can be distributed to different Galaxy servers. To improve the quality of recommendations, the annotations of tools can be incorporated in the learning mechanism by assigning higher weights to the annotated tools in comparison to tools which are not annotated. Tools containing similar annotations may have similar functionalities and using these similarities, tool recommendations can be further enhanced by showing similar tools for each recommended tool.”

The inclusion of new tools is discussed in the “New tools as recommendations” subsection of “Methods” section.

[Methods]

"usage based relevance" could be "usage-based relevance" or just "usage statistics" or even just "usage frequency" itself.

Author response:

We have addressed it in the paper.

GRU layer: I like the concise explanation! Similarly for the activation functions, and the prediction of usage frequency.

Author response:

Thank you for the appreciation.

"time complexity", no hyphen.

Author response:

We have addressed it in the paper.

"parameters (of) these architectures"

Author response:

We have addressed it in the paper.

Space permitting, it would be a nice extra to have figures of the used CNN and "DNN" architectures included as well, like the Figure 8 for the GRU RNN, near the "Multiple neural network architectures" subsection.

Author response:

We have added different neural network architecture images to the section S3 of the supplementary document.

This was all from my side, I hope to see this article published in GigaScience!

Sincerely yours,
Matúš Kalaš

Close
