# Supplementary material for: Tool recommender system in Galaxy using deep learning
Source: Gigascience. 2021 Jan 6;10(1):giaa152. doi: 10.1093/gigascience/giaa152 (PMC7786169; doi:10.1093/gigascience/giaa152)
Supplement: giaa152_Response_to_Reviewer_Comments_Revision_1 [file giaa152_response_to_reviewer_comments_revision_1.pdf]

Author's Response To Reviewer Comments

Close

GIGA-D-20-00053R1
Tool recommender system in Galaxy using deep learning
Anup Kumar; Helena Rasche; Björn Grüning; Rolf Backofen
GigaScience

Dear Anup,

Your manuscript "Tool recommender system in Galaxy using deep learning" (GIGA-D-20-00053R1) has been re-reviewed by our reviewers. Based on these reports, and my own assessment as Editor, I am pleased to inform you that it is potentially acceptable for publication in GigaScience, once you have carried out some final mnior revisions suggested by our reviewers.

Author comment:
We thank the reviewers and the editor for the feedback.

Their reports, together with any other comments, are below. Please also take a moment to check our website at https://www.editorialmanager.com/giga/ for any additional comments that were saved as attachments.

Once you have made the necessary corrections, please submit a revised manuscript online at:

https://www.editorialmanager.com/giga/

If you have forgotten your username or password please use the "Send Login Details" link to get your login information. For security reasons, your password will be reset.

Please include a point-by-point within the 'Response to Reviewers' box in the submission system. Please ensure you describe additional experiments that were carried out and include a detailed rebuttal of any criticisms or requested revisions that you disagreed with. Please also ensure that your revised manuscript conforms to the journal style, which can be found in the Instructions for Authors on the journal homepage. If the data and code has been modified in the revision process please be sure to update the public versions of this too.

The due date for submitting the revised version of your article is 07 Dec 2020.

We look forward to receiving your revised manuscript soon.

Best wishes,

Scott Edmunds, Ph.D.
GigaScience
www.gigasciencejournal.com

Reviewer reports:
Reviewer #1: This paper has been previously reviewed for the Journal and therefore I will not provide an overview in this review.

I am satisfied that all of the major concerns about the previous version of the paper have been addressed by the authors, and I am happy to recommend the paper to be accepted for publication.

Author comment:
Thank you for your feedback and making the paper better.

I have made some small observations below for consideration in the final revision:

This sentence is imprecise and non-committal for a scientific paper: "This should allow good quality tools to be shown as the top recommendations." Likewise "which should allow it ..." Saying "should" sounds like you are not sure if the statement is true or not, but are merely hypothesising.

Author comment:
Thank you for pointing it out. We have made the necessary corrections in the paper.

"First, using a simple approach to store all the indices of sequences of tools" it is unclear what this means: one has to read the supplementary materials to find out. Is there another way to describe this technique?

Author comment:
Thank you for your comment. We have added more detailed information to the paper. The relevant text from the paper is given below:

“The first approach simply stores all the sequences of tools formed using the technique shown in Figure 1 to create a model. To recommend tools using this model, all the tool sequences are searched for a given tool or a sequence of tools.”

The "standard" and "normal" categories for tools don't strike me as the most informative of terms.

Author comment:
Thank you for your comment. We have renamed these categories as “shared” (instead of standard) and “non-shared” (instead of normal) tools.

The paper is rather long, and I would encourage the authors to seek opportunities to be more concise.

Author comment:
Thank you for your comment. We have moved the section “Multiple neural network architectures” from the paper to the supplementary document (section S3) and shortened the “Hyperparameter tuning” section in the paper by moving some details to the supplementary document (section S7). In addition, we have also unified “Relevance of tools” and “Usage frequencies of tools as weights” sections in the paper.

Reviewer #3: This paper describes an interesting and novel approach to building a tool recommender system for workflow construction.

This version of the paper shows some good improvements and the authors have gone a good way towards addressing reviewer comments.

Author comment:
We thank the reviewer for providing feedback.

However, there are some parts of the manuscript that would still benefit from further elaboration.

The issue of workflow quality and the bearing this has on the quality of training and test data requires further justification. The authors state "To distinguish between good and poor quality tool connections, the labels for each tool sequence are divided into two parts - standard and normal labels. The standard labels come from the published, non-deleted and non-erroneous workflows, while the normal labels come from other workflows"
Clearly, it is important to distinguish between good and poor quality workflows, but accepting that published workflows are "good" is simplistic. Workflows are dynamic and decay over time. Published workflows may no longer work and should NOT be used as training data for good recommendations. If the workflow are tested more extensively than this, the authors should provide details of the testing. If they are not, the authors should add a section to the manuscript about the limitations of their method.

Author comment:
Thank you for your comment. We have divided the workflows into shared and non-shared considering that the shared workflows (published, non-deleted and non-erroneous) have more relevant tool connections than the non-shared ones. In addition, we have also implemented a constraint of usage frequencies of tools over the last one year. It has been applied as weights of tools in the neural network training to filter out tools which have not been used at all in the last one year irrespective of their origin, either shared or non-shared workflows. The tools which get old or deprecated and are not being used get automatically filtered out of the list of recommendations and the tools which are from shared workflows and have been used often in the last one year are shown at the top of recommendations. On the other hand, if we exclude those workflows which no longer work from the training data, then we risk missing one or more relevant tool connections in these workflows which are actually being used by users. The neural network will have no chance to learn from these connections which may produce sub-optimal recommendations. The section "Usage frequencies of tools as weights" in the paper provides further detail.

To conclude, it is always very challenging to learn on a meaningful but powerful dataset. The system is highly configurable for a Galaxy admin, who can create their own models if needed. Moreover, we are working currently with projects like Dockstore and WorkflowHub.eu to share high quality workflows which will also be used as learning datasets in the future.

In this section, they should also address the effects of recommending connections without any details of parameter selection and also how recommending individual tools compares to recommending chains of tools that are always used together.

Author comment:
Thank you for your comment. We have added a section (S8: Tool parameters and individual tool recommendations) to the supplementary document. Please find the relevant text from the section below:

"In Galaxy, a tool has many parameters and when these parameters are configured differently, the tool may connect to multiple tools. In our approach, tool connections in workflows are used to recommend tools. If there are such patterns, a tool connecting to multiple tools, available in the workflows, our recommendation model captures them. For example if tool A, with a different configuration of parameters, connects to tool B and with another configuration, connects to tool C, then our model learns both these connections for tool A -> tool B and tool A -> tool C. While recommending tools for tool A, both these connections (tool B and tool C) are predicted. However, if there are no patterns available showing the use of multiple connections of tools based on different configuration of parameters, our model does not know about it as we can learn and predict only what we see.

In addition, only one tool is recommended at a time instead of recommending a chain of tools. Recommending one tool at a time is beneficial for a few reasons. First, it enables users to diversify the usage of tools by showing tools from multiple scientific analyses at each step of their analyses. Second, if chains of tools which are always used together are recommended and if one or more of them get deprecated (maybe because they are old and not used anymore), it would make little sense to show them together and at the same time, excluding them would introduce a gap in that particular analysis. But, if we predict only one tool at a time and the tool gets deprecated, we simply exclude it and show only relevant tools. Lastly, it is not the aim of the project currently to recommend chains of tools."

I would also like a clearer description of the training and testing set of workflows. Some workflows exist as multiple published "copies" because they are used in Galaxy training courses. How do these workflows bias the recommendation results?

Author comment:
Thank you for your comment. We collect all workflows from the EU Galaxy server and extract paths (tool sequences) from these workflows. All duplicate paths are removed as part of the pre-processing step. The unique set of tool sequences are divided into training and test sets. The training set is used for training the neural network and comprises 80% of all tool sequences and the rest 20% is used as the test set for evaluation. The section “Learning and predictions” in the paper provides further details.

In addition, the tool sequences are fed uniformly to the neural network which means that the number of tool sequences from different scientific analyses is kept similar. Therefore, even if there are multiple “copies” of shared or non-shared workflows, it will not affect the recommendations as the neural network in each iteration trains on tool sequences coming from multiple scientific analyses uniformly. The section “Uniform sampling” in the paper provides further details.

A minor point related to that above - the semantics of "standard" and "normal" is not a very clear distinction.

Author comment:
Thank you for your comment. We have renamed these categories as “shared” (instead of standard) and “non-shared” (instead of normal) tools.

The authors describe the implementation of their system in Galaxy Europe and good feedback from the user community, but how many times have the recommendations actually been used? Can the authors provide further information on this? If not, can they start collecting this information now and maybe adding recommender use into Galaxy training programmes to explore this in a more systematic way?

Author comment:
Thank you for your comment. We can easily count the API usage of the tool recommender system. But, one of the UI (user interface) elements in Galaxy uses it automatically, without user intervention. Therefore, just counting the API usage is not accurate, but a number that we can provide: ~2000/24h (usage count/time). We have added URL components to both the UI elements (Figures 6 and 7 in the paper) showing tool recommendations which will enable us to count the usage of the webhook. Recently, this feature has been made available with the new release of Galaxy 20.09 on the EU server. On a similar note, we would like to point out that recommendations can also be consumed purely in a “visual” manner without countable interactions. The user feedback we received overtime (personal conversations) indicates that the “visual” indications are educative and that they are used to explore tools more often. This hits in the direction that the usefulness of such recommendations is much more than what can be tracked technically.

In general, we are hesitant to track user usage, as we try to be GDPR (general data protection regulation) compliant and we need to be cautious about what we track and where we store it.

Close
