# Supplementary material for: Tool recommender system in Galaxy using deep learning
Source: Gigascience. 2021 Jan 6;10(1):giaa152. doi: 10.1093/gigascience/giaa152 (PMC7786169; doi:10.1093/gigascience/giaa152)
Supplement: giaa152_Response_to_Reviewer_Comments_Revision_2 [file giaa152_response_to_reviewer_comments_revision_2.pdf]

Author's Response To Reviewer Comments

Close

Dear Anup

Your manuscript "Tool recommender system in Galaxy using deep learning" (GIGA-D-20-00053R2) has been assessed by our curators and editors for a final time. Based on the changes can you cite the GigaDB citation in the Available of Supporting Data Section at the end of the paper. The reference you should use is:

Kumar A; Rasche H; Gruening B; Backofen R (2020): Supporting data for "Tool recommender system in Galaxy using deep learning" GigaScience Database. http://dx.doi.org/10.5524/100838

I've sent you an email with an annotated paper explaining the changes in more detail.

Author comment:

Thank you for your feedback. We have made the following changes to the manuscript according to the feedback received as annotations in the manuscript.

1. We have added ORCIDs of all the authors beside their respective email ids.

2. We have included the Galaxy's RRID (RRID:SCR_006281) in "Library, model and code repositories" section of the manuscript.

3. We have added a short section "Availability of supporting data" just below the section "Availability of supporting source code and requirements" in the manuscript and cited the GigaScience GigaDB DOI.

Thank you!

Close
