# Supplementary material for: Tool recommender system in Galaxy using deep learning
Source: Gigascience. 2021 Jan 6;10(1):giaa152. doi: 10.1093/gigascience/giaa152 (PMC7786169; doi:10.1093/gigascience/giaa152)
Supplement: giaa152_Reviewer_1_Report_Original_Submission — Bernie Pope, Ph.D. -- 3/9/2020 Reviewed [file giaa152_reviewer_1_report_original_submission.pdf]

Reviewer Report

Title: Tool recommender system in Galaxy using deep learning

Version: Original Submission **Date:** 3/9/2020

Reviewer name: Bernie Pope, Ph.D.

Reviewer Comments to Author:

This paper describes a tool recommendation system for Galaxy workflows that employs a GRU neural network to train a classifier system. The classifier is trained on existing workflows that are recorded in the Galaxy system, and usage frequency of tools is additionally used to weight their relevance to the result.

This paper was submitted under the "Research" manuscript type, but I wonder whether it would have been better as a Technical note, given that the paper describes an implemented system, but there is no tested hypotheses or research question being answered.

There are some interesting ideas explored in this paper but I have some major concerns about its current presentation:

- The solution seems like it might be overkill for the problem that is being solved. The authors have not implemented or compared to any alternative approaches to solving the same problem, so we have no basis to understand whether a NN is needed for this problem, or whether a simpler statistical model would suffice.
- The paper does not address the issue of whether the tool is really useful in practice. For example it says "they [recommendations] improve user experience by helping researchers to easily create correct workflows". This claim is not tested in this paper. You would need to test user behaviour to find out, or in the very least, survey user experience with the tool.
- Such a recommendation system us potentially dangerous if it is giving poor/biased/incorrect suggestions to users, and is highly sensitive to the training data. It is not clear from the paper how biases in the training data are dealt with. For example, suppose the Galaxy server is used by students undergoing training. It could be the case that for training purposes students are taught initially to use older, out of date, tools and techniques, for the sake of simplicity. There could be very many of these students on the system. A large number of students using out-of-date techniques could seriously bias the results. The paper suggests re-training the system periodically, which is reasonable, however, how does the system protect against circular dependencies in the data, where it starts training on workflows that have used the recommendation system themselves?
- The system considers tools, but apparently not data types. In the example discussed on page 4, how would the system know to recommend RNA-STAR compared to BWA or Bowtie if it doesn't know whether the inputs are DNA or RNA?

Beyond those concerns above, I feel that the paper is imbalanced in its structure. Too much space is used to discuss general features of NNs, whereas too little attention is paid to the actual methods used in the project. For example, the actual size and nature of the training data is only very briefly mentioned near the end of the paper "The number of tool sequences extracted from workflows is approximately

200,000..." This is a very important detail that deserves more attention. We don't know the proportions of the training workflows for different kinds of analyses, and therefore it is hard to say anything about selection bias in the data. What if 90% of the training data is for DNA sequencing, how would this affect the ability of the system to recommend proteomics tools for example?

Level of Interest

Please indicate how interesting you found the manuscript: Choose an item.

Quality of Written English

Please indicate the quality of language in the manuscript: Choose an item.

Declaration of Competing Interests

Please complete a declaration of competing interests, considering the following questions:

- Have you in the past five years received reimbursements, fees, funding, or salary from an organisation that may in any way gain or lose financially from the publication of this manuscript, either now or in the future?
- Do you hold any stocks or shares in an organisation that may in any way gain or lose financially from the publication of this manuscript, either now or in the future?
- Do you hold or are you currently applying for any patents relating to the content of the manuscript?
- Have you received reimbursements, fees, funding, or salary from an organization that holds or has applied for patents relating to the content of the manuscript?
- Do you have any other financial competing interests?
- Do you have any non-financial competing interests in relation to this paper?

If you can answer no to all of the above, write 'I declare that I have no competing interests' below. If your reply is yes to any, please give details below.

I declare that I have no competing interests

I agree to the open peer review policy of the journal. I understand that my name will be included on my report to the authors and, if the manuscript is accepted for publication, my named report including any attachments I upload will be posted on the website along with the authors' responses. I agree for my report to be made available under an Open Access Creative Commons CC-BY license (http://creativecommons.org/licenses/by/4.0/). I understand that any comments which I do not wish to be included in my named report can be included as confidential comments to the editors, which will not be published.

Choose an item.

To further support our reviewers, we have joined with Publons, where you can gain additional credit to further highlight your hard work (see: https://publons.com/journal/530/gigascience). On publication of this paper, your review will be automatically added to Publons, you can then choose whether or not to claim your Publons credit. I understand this statement.

Yes Choose an item.
