# Supplementary material for: Tool recommender system in Galaxy using deep learning
Source: Gigascience. 2021 Jan 6;10(1):giaa152. doi: 10.1093/gigascience/giaa152 (PMC7786169; doi:10.1093/gigascience/giaa152)
Supplement: giaa152_Reviewer_2_Report_Original_Submission — Jeremy Leipzig -- 3/31/2020 Reviewed [file giaa152_reviewer_2_report_original_submission.pdf]

Reviewer Report

Title: Tool recommender system in Galaxy using deep learning

Version: Original Submission **Date:** 3/31/2020

Reviewer name: Jeremy Leipzig

Reviewer Comments to Author:

The authors present a Galaxy tool recommendation system based on a training set of over 200,000 tool sequences from existing workflows. The final version of this system uses a GRU neural network. Although extensive testing was done to compare and benchmark this model with other neural networks, it should be noted there is no existing conventional approach (Markov Model/market basket/Bayesian) but I don't feel that it is necessary to create a straw man for this purpose. The neural network offers a high degree of accuracy without the need to manually annotate each tool.
The description of the GRU implementation, testing, and optimization is very good and will inform readers about how to implement similar solutions for other applications. The Github repository is also well organized. This paper is well suited to this journal and should be accepted with minor revisions.
I have three minor revisions to suggest:
1. I think most readers would understand what overfitting would look like in a typical machine learning, but maybe not in a recommendation engine for tools. What noise or error that is propagated by an overfit recommendation engine? For instance, would it resemble obscure tools that some edge case user chose? More importantly, what does the regularization step actually do in this case - recommend a repertoire of more common tools, or simply remain agnostic?
Provide a real-world example, something like…
"An example of overfitting in our recommendation system may involve tools with few use cases (e.g. tools for dealing with organisms with no reference assembly). The GRU would learn only from a limited training set and which could recommend tools that would not be appropriate for most users."
2. It appears the recommendation engine cannot recommend entirely new tools without completely rebuilding the model. Please explain how often the model is rebuilt in practice. Also explain how new tools would ever be recommended if they don't appear in existing workflows. Is the design that entirely new tools would be adopted into workflows by "power users" and therefore trickle down to more casual Galaxy users?
3. DESeq2 is miscapitalized as DeSeq2 on pg2

Level of Interest

Please indicate how interesting you found the manuscript: Choose an item.

Quality of Written English

Please indicate the quality of language in the manuscript: Choose an item.

Declaration of Competing Interests

Please complete a declaration of competing interests, considering the following questions:

- Have you in the past five years received reimbursements, fees, funding, or salary from an organisation that may in any way gain or lose financially from the publication of this manuscript, either now or in the future?
- Do you hold any stocks or shares in an organisation that may in any way gain or lose financially from the publication of this manuscript, either now or in the future?
- Do you hold or are you currently applying for any patents relating to the content of the manuscript?
- Have you received reimbursements, fees, funding, or salary from an organization that holds or has applied for patents relating to the content of the manuscript?
- Do you have any other financial competing interests?
- Do you have any non-financial competing interests in relation to this paper?

If you can answer no to all of the above, write 'I declare that I have no competing interests' below. If your reply is yes to any, please give details below.

I declare that I have no competing interests

I agree to the open peer review policy of the journal. I understand that my name will be included on my report to the authors and, if the manuscript is accepted for publication, my named report including any attachments I upload will be posted on the website along with the authors' responses. I agree for my report to be made available under an Open Access Creative Commons CC-BY license (http://creativecommons.org/licenses/by/4.0/). I understand that any comments which I do not wish to be included in my named report can be included as confidential comments to the editors, which will not be published.

Choose an item.

To further support our reviewers, we have joined with Publons, where you can gain additional credit to further highlight your hard work (see: https://publons.com/journal/530/gigascience). On publication of this paper, your review will be automatically added to Publons, you can then choose whether or not to claim your Publons credit. I understand this statement.

Yes Choose an item.
