# Supplementary material for: Tool recommender system in Galaxy using deep learning
Source: Gigascience. 2021 Jan 6;10(1):giaa152. doi: 10.1093/gigascience/giaa152 (PMC7786169; doi:10.1093/gigascience/giaa152)
Supplement: giaa152_Reviewer_3_Report_Original_Submission — Katy Wolstencroft -- 4/2/2020 Reviewed [file giaa152_reviewer_3_report_original_submission.pdf]

Reviewer Report

Title: Tool recommender system in Galaxy using deep learning

Version: Original Submission **Date:** 4/2/2020

Reviewer name: Katy Wolstencroft

Reviewer Comments to Author:

This paper describes an interesting and novel approach for learning from the large numbers of existing workflows in Galaxy. Using a neural network approach to learn which tools are used most frequently, and which tools are used in sequence, is a good basis for building a recommender system. The number of available Galaxy workflows provides a large test set to work with and spans a wide range of bioinformatics analyses. The recommender has already been embedded into the Galaxy.eu server. This increases the significance of the work as it means that a large user community can already benefit from the recommendations.

Major Points

1. The paper would benefit from a deeper discussion about the quality and validity of the workflows in the test set. Are all workflow used, or only those that run without generating an error? There are many workflows created during exploration that are never used for an eventual analysis, either because they are considered unsuitable by their creator, or because they simply did not run. How are these 'test' workflows separated from 'production' workflows that are used to generate actual results?

2. Related to the point above - is there an attempt to collect and learn from tool parameter information in addition to the tool function? Some tools perform multiple functions and therefore behave very differently depending upon their configuration.

3. The recommender system is based on suggesting the next tool, but could the recommender system also be used to suggest whole configured workflows as the next step? If a collection of tools are always used together, suggesting one at a time (without parameter information) would be less accurate overall than suggesting a fully configured workflow for all the subsequent steps.

4. Recommendations are based on tool use frequency data and previous workflows. If a new tool is developed, there will be a lag before this tool is recommended to users, even if it is 'better' than similar tools. How could this problem be addressed? Similarly, using recommendations may lead to less innovation in workflow creation. Could the authors discuss the implications of this (i.e. who is the recommender for? Is it for the workflow expert, or for users who need more bioinformatics support?)

6. Have the authors performed a user evaluation on their recommender system? It is already part of Galaxy.eu and the manuscript claims it "improves user experience by helping researchers to easily create correct workflows". However, there are no details of current usage or of how this has been assessed to date. As it is available, I have used it, and it is very intuitive, but the paper would be strengthened by adding a more formal evaluation of usage to date.

Minor point

In the results section, the example workflow is based on NGS sequence analysis (Trimmomatic -->

Bowtie2 --> FreeBayes). This is a good example, however, in the text, the authors suggest that after bowtie2, FastQC might be recommended. Usually, FastQC would be used before Trimmomatic.

Level of Interest

Please indicate how interesting you found the manuscript: Choose an item.

Quality of Written English

Please indicate the quality of language in the manuscript: Choose an item.

Declaration of Competing Interests

Please complete a declaration of competing interests, considering the following questions:

- Have you in the past five years received reimbursements, fees, funding, or salary from an organisation that may in any way gain or lose financially from the publication of this manuscript, either now or in the future?
- Do you hold any stocks or shares in an organisation that may in any way gain or lose financially from the publication of this manuscript, either now or in the future?
- Do you hold or are you currently applying for any patents relating to the content of the manuscript?
- Have you received reimbursements, fees, funding, or salary from an organization that holds or has applied for patents relating to the content of the manuscript?
- Do you have any other financial competing interests?
- Do you have any non-financial competing interests in relation to this paper?

If you can answer no to all of the above, write 'I declare that I have no competing interests' below. If your reply is yes to any, please give details below.

I declare that I have no competing interests

I agree to the open peer review policy of the journal. I understand that my name will be included on my report to the authors and, if the manuscript is accepted for publication, my named report including any attachments I upload will be posted on the website along with the authors' responses. I agree for my report to be made available under an Open Access Creative Commons CC-BY license (http://creativecommons.org/licenses/by/4.0/). I understand that any comments which I do not wish to be included in my named report can be included as confidential comments to the editors, which will not be published.

Choose an item.

To further support our reviewers, we have joined with Publons, where you can gain additional credit to further highlight your hard work (see: https://publons.com/journal/530/gigascience). On publication of this paper, your review will be automatically added to Publons, you can then choose whether or not to claim your Publons credit. I understand this statement.

Yes Choose an item.
