# Supplementary material for: Tool recommender system in Galaxy using deep learning
Source: Gigascience. 2021 Jan 6;10(1):giaa152. doi: 10.1093/gigascience/giaa152 (PMC7786169; doi:10.1093/gigascience/giaa152)
Supplement: giaa152_Reviewer_3_Report_Revision_1 — Katy Wolstencroft -- 9/7/2020 Reviewed [file giaa152_reviewer_3_report_revision_1.pdf]

Reviewer Report

Title: Tool recommender system in Galaxy using deep learning

Version: Revision 1 **Date:** 9/7/2020

Reviewer name: Katy Wolstencroft

Reviewer Comments to Author:

This paper describes an interesting and novel approach to building a tool recommender system for workflow construction.
This version of the paper shows some good improvements and the authors have gone a good way towards addressing reviewer comments. However, there are some parts of the manuscript that would still benefit from further elaboration.
The issue of workflow quality and the bearing this has on the quality of training and test data requires further justification. The authors state "To distinguish between good and poor quality tool connections, the labels for each tool sequence are divided into two parts - standard and normal labels. The standard labels come from the published, non-deleted and non-erroneous workflows, while the normal labels come from other workflows"
Clearly, it is important to distinguish between good and poor quality workflows, but accepting that published workflows are "good" is simplistic. Workflows are dynamic and decay over time. Published workflows may no longer work and should NOT be used as training data for good recommendations. If the workflow are tested more extensively than this, the authors should provide details of the testing. If they are not, the authors should add a section to the manuscript about the limitations of their method. In this section, they should also address the effects of recommending connections without any details of parameter selection and also how recommending individual tools compares to recommending chains of tools that are always used together. I would also like a clearer description of the training and testing set of workflows. Some workflows exist as multiple published "copies" because they are used in Galaxy training courses. How do these workflows bias the recommendation results?
A minor point related to that above - the semantics of "standard" and "normal" is not a very clear distinction.
The authors describe the implementation of their system in Galaxy Europe and good feedback from the user community, but how many times have the recommendations actually been used? Can the authors provide further information on this? If not, can they start collecting this information now and maybe adding recommender use into Galaxy training programmes to explore this in a more systematic way?

Level of Interest

Please indicate how interesting you found the manuscript: Choose an item.

Quality of Written English

Please indicate the quality of language in the manuscript: Choose an item.

Declaration of Competing Interests

Please complete a declaration of competing interests, considering the following questions:

- Have you in the past five years received reimbursements, fees, funding, or salary from an organisation that may in any way gain or lose financially from the publication of this manuscript, either now or in the future?
- Do you hold any stocks or shares in an organisation that may in any way gain or lose financially from the publication of this manuscript, either now or in the future?
- Do you hold or are you currently applying for any patents relating to the content of the manuscript?
- Have you received reimbursements, fees, funding, or salary from an organization that holds or has applied for patents relating to the content of the manuscript?
- Do you have any other financial competing interests?
- Do you have any non-financial competing interests in relation to this paper?

If you can answer no to all of the above, write 'I declare that I have no competing interests' below. If your reply is yes to any, please give details below.

I declare that I have no competing interests

I agree to the open peer review policy of the journal. I understand that my name will be included on my report to the authors and, if the manuscript is accepted for publication, my named report including any attachments I upload will be posted on the website along with the authors' responses. I agree for my report to be made available under an Open Access Creative Commons CC-BY license (http://creativecommons.org/licenses/by/4.0/). I understand that any comments which I do not wish to be included in my named report can be included as confidential comments to the editors, which will not be published.

Choose an item.

To further support our reviewers, we have joined with Publons, where you can gain additional credit to further highlight your hard work (see: https://publons.com/journal/530/gigascience). On publication of this paper, your review will be automatically added to Publons, you can then choose whether or not to claim your Publons credit. I understand this statement.

Yes Choose an item.
