# Supplementary material for: Tool recommender system in Galaxy using deep learning
Source: Gigascience. 2021 Jan 6;10(1):giaa152. doi: 10.1093/gigascience/giaa152 (PMC7786169; doi:10.1093/gigascience/giaa152)
Supplement: giaa152_Reviewer_4_Report_Original_Submission — MatÃoÅ¡ KalaÅ¡ -- 4/20/2020 Reviewed [file giaa152_reviewer_4_report_original_submission.pdf]

Reviewer Report

Title: Tool recommender system in Galaxy using deep learning

Version: Original Submission **Date:** 4/20/2020

Reviewer name: MatÃºÅ¡ KalaÅ¡

Reviewer Comments to Author:

Dear authors and editor(s),
After seeing a conference presentation and the tool recommender in usegalaxy.eu last summer, and noting the bioRxiv preprint of this work last November (https://doi.org/10.1101/838599), I am happy seeing that authors submitted the manuscript to GigaScience. Compared to the preprint, the text has been polished and better structured into sections.
I find the manuscript very nicely written and relatively easy to read (with only minor opportunities for editorial polishing, some mentioned below). As a reader, I highly appreciate the effort of the authors towards making the text reader-friendly. I find it also clear from the text, how the data was collected and curated, and where it is openly published. The manuscript also points to the used source code (scripts), and all is available together under a permissive open source software license.
I did not have the capacity to test the various scripts - for extracting data and all of the models training - but I repeatedly tested the user interface on the usegalaxy.eu server, useful from the user's point of view.
I'm very happy with the content and the results of this work, and the manuscript convinces me about the choices taken in the authors' approach. I don't recommend a revision of the work. My following suggestions are only about the code repository and some minute, mostly editorial improvements of the text, plus a couple of brief comments.
Repositories and branches:
This manuscript links to a repository containing all the used code and data (https://github.com/anuprulez/galaxy_tool_recommendation), created in February 2020 by a mass upload of most of the code and data. I am really happy to see that some updates have been pushed to this repo in the recent weeks of April 2020.
The history of the work is recorded in another repo (https://github.com/anuprulez/similar_galaxy_workflow), with various branches active last year, a separate master branch subsequently active in Sep and Oct, and a 'tool_recommendation_release_19_09' branch active aftewards between Oct 2019 and Feb 2020. It would be nice to note the history and the relations between these repos and branches somewhere in the beginning of all the involved README.md files, especially so that the community sees where to look for the active work and where not (and eventually fork, experiment, contribute, start issue threads, etc.).
It would be a nice bonus to have the version used in the article tagged as a release on GitHub, ideally with a Zenodo archive DOI (https://zenodo.org/oauth/login/github/?next=%2Fdeposit followed by https://zenodo.org/account/settings/github/), which could be permanently linked from the published

article.
Notably, the README.md currently contains useful information on how to reproduce the work, and use it on another Galaxy server. I would also welcome in the near future some additional brief information about this project, and explicitly separate "how to-s" for various types of usage/users: Galaxy end users, Galaxy admins, researchers who want to reproduce the work, existing contributors to the project, and potential new contributors. CONTRIBUTING.md might be a good option for the latter or latter 2; and/or README.md can include the overview of all the information, with further links to potential other files with details.
Abstract:
The abstract of the manuscript is nicely concise and gives a good summary of the work. I suggest it could possibly be made even easier to read and understand (especially for unacquainted readers), with a couple of small changes.
"To make creating workflows easier, faster and less error-prone, a predictive system is developed to recommend tools facilitating further analysis." could be simplified into something in the sense of "To help (users) with creating workflows, we developed a system to recommend tools that (would) facilitate further analysis."
"A model is developed to recommend tools by analysing workflows, composed by researchers" - without comma, or comma after 'tools' would read smoother.
"precision@1, precision@2 and precision@3 metrics" - would it be clearer with e.g. "top-one, top-two, and top-three metrics", or not?
The Conclusion(s) part is hard to read. The preprint included a sentence similar to "The model is accessed by the Galaxy API to recommend tools in real time.", which could perhaps fit in. Subsequent "Multiple user interface (UI) integrations on the European Galaxy server communicate with an API, which accesses the model, to apprise researchers of recommended tools in an interactive manner." could be simplified - especially if the previous sentence from the preprint is included in some form - into e.g.: "Multiple user-interface integrations on the European Galaxy server communicate with the(?) API, which accesses the model, to provide researchers with recommended (next?) tools, in an interactive manner." The last sentence would be clearer with something like "The scripts to create the recommendation model, and the (sample) (training) data (we used), are available under MIT License at ..."
Main text:
The manuscript is excellently written, however, including some opportunities for minor improvements to smoothen some of the sentences. These include unusual or ambiguous words ('stemming', 'appropriately', the latter can be omitted); some punctuation (I find some missing commas, but appreciate nicely and correctly used hyphenation); and minor grammar nuances, e.g. "Galaxy is a(n) open-source data-processing platform...", or "time spent (in)".
I also included a couple of positive comments among the following notes.
[Introduction]
"... to ensure it, a system is needed which can recommend correct tools while creating a workflow." - both 'ensure' and 'correct' are too strong (restrictive) words in this context, claiming way too ambitious goals.
"has a defined number of data types for these input and output files" - clearer would be something like

"supports a number of formats of these input and output files", in case it is only the format that is the point here. (The use of 'data types' in what follows after is fine, unless the authors would want to be more precise (but verbose) with types of data and data formats. However, I don't suggest that it is necessary here. Still, one may want to take into account that outside of Galaxy, 'data type' relates to types in (typed) programming languages for programmers, and to acquisition technologies and techniques in the jargon of many biologists and medical researchers.)
"Second, it will help them bypass the step of searching for tools separately which shows potential to further reduce the time spent in creating workflows and increase the accessibility of tools." - For me personally, this is where I see the biggest benefit of the recommender system (bigger than the "First", 'correctness' use case). Maybe some smoothening of the sentence, e.g. "Second, it will help users bypass the step of searching for tools separately, what will further reduce the time spent in creating workflows and (at the same time) increase the accessibility of tools."
"Finally, it can also be used to promote the newly added tools in Galaxy by showing them alongside the recommended tools predicted using the deep learning approach." - This is really important and great!
"metadata of tools" might be clearer with "information about tools". Also in Discussion.
"higher-order relationships among tools" are explained in Figure 1. The explanation is sufficient, and aided by the figure, but perhaps it would then be nice to add a link to the figure where they are mentioned the first time (except for the abstract).
"A Bayesian network can also be used..." perhaps a new paragraph?
[Results]
"the next generation sequencing (NGS) data" could just be "the sequencing data".
The recommended tools mentioned in the text following right after do not match with the recommended tools in Figure 5. It would be nice to explain what those in Figure 5 are, or match them with the text. Perhaps an easy UX fix: Display which step the recommended tools are for.
Figure 6 description: RNA-STAR is usually spelled with all capitals, as in the main text.
[Discussion] (Reading the content, it could also be titled "Summary and final remarks".)
"A recommender system to predict tools in Galaxy..." - Isn't it a "prediction system to recommend tools in Galaxy" instead? What about simply "A system to recommend tools in Galaxy..."?
"in real time."
The last 2 sentences could be part of a separate paragraph on "future work"/"future options", perhaps slightly more elaborate. For example, any UI ideas on how to suggest both highly-used and new tools transparently, and how to administer these (automatically?)? Or would it be worth experimenting with the inclusion of tools similarity, especially if these are in the future better annotated with semantic information?
[Methods]
"usage based relevance" could be "usage-based relevance" or just "usage statistics" or even just "usage frequency" itself.
GRU layer: I like the concise explanation! Similarly for the activation functions, and the prediction of usage frequency.
"time complexity", no hyphen.
"parameters (of) these architectures"
Space permitting, it would be a nice extra to have figures of the used CNN and "DNN" architectures

included as well, like the Figure 8 for the GRU RNN, near the "Multiple neural network architectures" subsection.
This was all from my side, I hope to see this article published in GigaScience!
Sincerely yours,
MatÃºÅ¡ KalaÅ¡

Level of Interest

Please indicate how interesting you found the manuscript: Choose an item.

Quality of Written English

Please indicate the quality of language in the manuscript: Choose an item.

Declaration of Competing Interests

Please complete a declaration of competing interests, considering the following questions:

- Have you in the past five years received reimbursements, fees, funding, or salary from an organisation that may in any way gain or lose financially from the publication of this manuscript, either now or in the future?
- Do you hold any stocks or shares in an organisation that may in any way gain or lose financially from the publication of this manuscript, either now or in the future?
- Do you hold or are you currently applying for any patents relating to the content of the manuscript?
- Have you received reimbursements, fees, funding, or salary from an organization that holds or has applied for patents relating to the content of the manuscript?
- Do you have any other financial competing interests?
- Do you have any non-financial competing interests in relation to this paper?

If you can answer no to all of the above, write 'I declare that I have no competing interests' below. If your reply is yes to any, please give details below.

I declare that I have no competing interests.

I agree to the open peer review policy of the journal. I understand that my name will be included on my report to the authors and, if the manuscript is accepted for publication, my named report including any attachments I upload will be posted on the website along with the authors' responses. I agree for my report to be made available under an Open Access Creative Commons CC-BY license (http://creativecommons.org/licenses/by/4.0/). I understand that any comments which I do not wish to

be included in my named report can be included as confidential comments to the editors, which will not be published.

Choose an item.

To further support our reviewers, we have joined with Publons, where you can gain additional credit to further highlight your hard work (see: https://publons.com/journal/530/gigascience). On publication of this paper, your review will be automatically added to Publons, you can then choose whether or not to claim your Publons credit. I understand this statement.

Yes Choose an item.
